# Bimetallic Uranium Complexes with 2,6-Dipicolinoylbis(*N*,*N*-Dialkylthioureas)

**DOI:** 10.3390/molecules29215001

**Published:** 2024-10-22

**Authors:** Christelle Njiki Noufele, Dennis Schulze, Maximilian Roca Jungfer, Adelheid Hagenbach, Ulrich Abram

**Affiliations:** 1 Institute of Chemistry and Biochemistry, Freie Universität Berlin, Fabeckstr. 34/36, 14195 Berlin, Germany; chrisnjiki@gmail.com (C.N.N.); dennis.schulze1@gmail.com (D.S.);; 2 Ruprecht-Karls Universität Heidelberg, Im Neuenheimer Feld 271, 69120 Heidelberg, Germany; maximilian.roca.jungfer@uni-heidelberg.de

**Keywords:** uranium, transition metals, mixed-metal complexes, 2,6-Dipicolinoylbis(*N*,*N*-dialkylthioureas)

## Abstract

2,6-Dipicolinoylbis(*N,N*-dialkylthioureas), H_2_L^R^, readily react with uranyl salts under formation of monomeric or dimeric complexes of the compositions [UO_2_(L^R^)(solv)] (solv = donor solvents such as H_2_O, MeOH or DMF) or [{UO_2_(L^R^)(µ-OMe)}_2_]^2−^ (**1**). In such complexes, the uranyl ions are exclusively coordinated by the “hard” *O*,*N*,*O* or *N,N,N* donor atom sets of the central ligand unit and the lateral sulfur donor atoms do not participate in the coordination. Different conformations have been found for the dimeric anions. The bridging methanolato ligands and the four uncoordinated sulfur atoms can adopt different orientations with respect to the equatorial coordination spheres of the uranyl units. The presence of non-coordinated sulfur atoms offers the opportunity for the coordination of additional, preferably “soft” metal ions. Thus, reactions with [AuCl(PPh_3_)], lead acetate or acetates of transition metal ions such as Ni^2+^, Co^2+^, Fe^2+^, Mn^2+^, Zn^2+^, or Cd^2+^, were considered for the syntheses of bimetallic complexes. Various oligometallic complexes with uranyl units were prepared: [{UO_2_(L^R^)(μ-OMe)(Au(PPh_3_)}_2_] (**2**), [(UO_2_)_3_Pb_2_(L^R^)_4_(MeOH)_2_(μ-OMe)_2_] (**3**), [M{UO_2_(L^R^)(OAc)}_2_] (M= Zn, Ni, Co, Fe, Mn or Cd) (R = Et: **5**, RR = morph: **6**), or [(UO_2_)(NiI)_2_(L^R^)_2_] (**7**). The products were extensively studied spectroscopically and by X-ray diffraction.

## 1. Introduction

The coordination chemistry of uranium is of interest from different points of view: (i) there is a permanent demand for the development and improvement of separation techniques for this actinide element, (ii) the optimization of the fuel cycle chemistry from the production of nuclear fuel elements until the reprocessing of spent nuclear fuel element requires more input particularly for the treatment of multi-metal solutions and (iii) a deeper insight is required into the biological chemistry of the radioactive element [1,2,3,4,5,6,7,8,9,10,11,12,13,14,15,16,17,18,19,20,21,22,23,24,25]. Particularly the latter point, which is related to the increasing bioavailability of soluble uranium compounds (mainly containing uranyl units) in mining regions also involves hitherto somewhat underestimated uranium complexes, For example, the coordination chemistry with “soft” donor atoms. {UO_2_}^2+^ units are commonly regarded as “hard” acids according to Pearson’s concept [26]; thus, the vast majority of structurally studied uranyl compounds comprise complexes with oxygen or other “hard” donors. Compounds with “soft” donor atoms are frequently regarded as instable and, thus, are not in the main focus for technological processes. For the evaluation of potential uptake and distribution patterns in biological systems, however, particularly interactions with sulfur-containing binding sites such as thioethers, thiolates or thiocarbonyl units are of interest, having in mind their important roles in the accumulation, transport and functional activity of metal ions in biological systems in general.

**Scheme 1:**
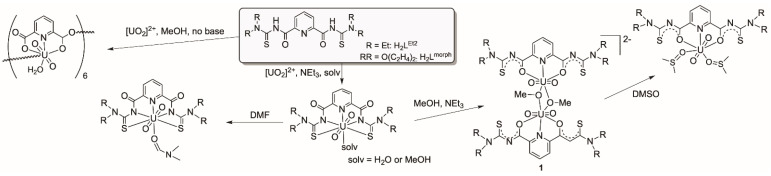
Reaction patterns of H_2_L^R^ ligands with {UO_2_}^2+^ ions [41].

The available structural information about uranium(VI) complexes with sulfur-containing ligands is relatively restricted, and the Cambridge Structural Database contains less than 70 uranyl complexes with uranium–sulfur bonds [27], while some more is known about corresponding compounds with uranium in lower oxidation states [28,29,30]. The ligands involved in the related uranyl studies mainly belong to chalcogenocarbamates, thiosemicarbazones and related compounds, bis(thiophosphinoyl)methanediides or imidodiphosphinochalcogenides [28,29,30,31,32,33,34,35,36,37,38,39,40]. In a recent paper, uranyl complexes with bifunctional aroylbis(dialkylthioureas), H_2_L^R^ (Scheme 1), have been studied [41]. Such ligands possess “hard” (carbonyl units), “medium soft” (pyridine rings), and “soft” (thiourea moieties) donor atoms and allow a flexible coordination behavior depending on the respective metal ions [42,43,44,45,46,47]. Only weak uranium–sulfur bonds are established with {UO_2_}^2+^ ions, which are readily cleaved when alternative bonding modes. For example, coordination to (hard) donor solvents becomes available (Scheme 1).

The reactions summarized in Scheme 1 confirm the structural flexibility of the H_2_L^R^ ligands and the obvious pH dependence of reactions including the fact that the bis(thio–ureas) decompose under strongly acidic conditions as in methanolic solutions of uranyl nitrate or uranyl acetate under formation of hexameric complexes with dipicolinato ligands, when no supporting base is added. Corresponding reactions in the presence of NEt_3_ give chelate complexes with intact, doubly deprotonated {L^R^}^2−^ ligands. The organic ligands coordinate pentadentate, when only small amounts of NEt_3_ is added, while solvolysis under formation of dimers with bridging methanolato ligands is observed at higher pH. In the products of the methanolysis, the uranium–sulfur bonds are cleaved and the picolinoylbis(thioureato) ligands act as tridentate *O,N,O* chelates. The sulfur atoms remain uncoordinated even after cleavage of the dimers by reaction with DMSO [41].

The unexpected pH dependence of such reactions and the presence of uncoordinated “soft” sulfur donors in some of the products stimulated us to conduct ongoing experiments with variable amounts of NEt_3_ and with the addition of “soft” metal ions, which might be able to coordinate to the vacant thioureato units of the coordinated {L^R^}^2−^ ligands.

## 2. Results and Discussion

### 2.1. Structural Isomerism of Dimeric Uranyl Complexes with H_2_L^R^ Ligands

An ongoing study of the reactions of uranyl compounds with H_2_L^R^ ligands described in ref. [41] shows that the dimeric [{UO_2_(L^Et2^)(µ-OMe)}_2_]^2−^ anion (compound **1** of Scheme 1) can be isolated in different conformations depending on the solvents and the cations used. Since such a behavior may have an influence on the attempted reactions with other metal ions, some attention should be devoted to this point.

**Figure 1:**
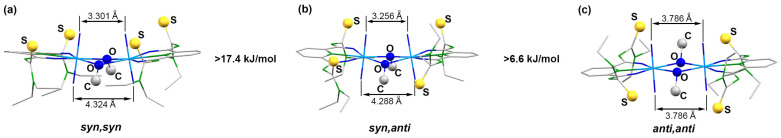
Conformations of the [{UO_2_(L^Et2^)(µ-OMe)}_2_]^2−^ (**1**) anions observed in the solid-state of the compound together with the small energy differences between the *syn,syn* (**a**), the, *syn anti* (**b**) and the *anti,anti* isomers (**c**) according to DFT calculations.

The previously structurally characterized anion **1** has been crystallized as a triclinic bis(triethylammonium) salt from a CH_2_Cl_2_ solution and may be described as a *syn,syn*-[{UO_2_(L^Et2^)(µ-OMe)}_2_]^2−^ isomer. This means that the two methyl groups of the bridging methanolato ligands as well as the four (uncoordinated) sulfur atoms each point to one side of the equatorial plane of the complex anion. This plane is formed by the uranium atoms, the O,N,O donor sets of the deprotonated aroylthioureato ligands and the oxygen atoms of the bridging methanolates as depicted in Figure 1a. During the recrystallization of this compound from CHCl_3_, an isomerization was observed and the isolated orange-yellow crystals were monoclinic and contained the compound in an *anti,anti*-conformation, which is shown in Figure 1b. Interestingly, similar ligand rearrangements have also been found during simple salt metatheses, such as the production of ethyltriphenylphosphonium salts of the uranium complexes. Several isomers could be isolated from such procedures including the *syn,anti* isomer shown in Figure 1c. DFT calculations on B3LYP/LANL2DZ(uranium)+6-311++G**(others) and PBE0-GD3BJ/StuttgartRLC(uranium)+def2-TZVPPD(others) level were performed to assess the conformational stability of the complexes. While a neglectable preference for the *anti,anti* conformation and the *syn,anti* conformation was found for the *N*,*N*-diethyl and morpholine derivatives on the B3LYP level respectively, the *syn,syn* structure is the least favored conformer in both cases. The *syn,anti* conformer of the morpholine derivative is the most stable isomer by nearly 20 kJ/mol on the higher PBE0-GD3BJ level of calculation, which includes dispersion effects. Structural differences resulting in energetic differences ranging around 20 kJ/mol as identified in the present case can readily be overcome by solvent effects in solution and packing effects in the solid state that become relevant during the crystallization processes. The individual B3LYP energy values for the corresponding experimentally observed geometries are displayed in Figure 1.

**Figure 2:**
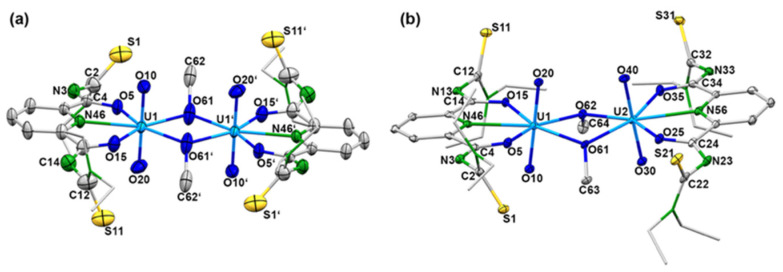
Structures of the complex anions of (**a**) (HNEt_3_)_2_ *anti,anti*-[{UO_2_(L^Et2^)(µ-OMe)}_2_] (symmetry operation: 0.5–x, 1.5–y,–z) and (**b**) (EtPPh_3_)_2_*syn,anti*-[{UO_2_(L^Et2^)(µ-OMe)}_2_]. Thermal ellipsoids represent 30% probability. For color code see atom labelling scheme.

While details of the structure of the *syn,syn*-[{UO_2_(L^Et2^)(µ-OMe)}_2_]^2−^ anion have already been reported [41], ellipsoid representations of the *anti,anti* and *syn,anti* isomers are shown in Figure 2. The different orientations of the methanolato ligands and the thiourea sulfur atoms relative to the central coordination plane are clearly seen. The *anti,anti* orientation of these units in the (HNEt_3_)_2_[{UO_2_(L^Et2^)(µ-OMe)}_2_] isomer shown in Figure 2a is enforced by the inversion center of the molecule being located in the central U_2_O_2_ unit. Selected bond lengths and angles are given in Table 1 and are compared with the values of the *syn,syn* isomer. A closer inspection of the values in this table reveals only a marginal difference between the three isomers. This is not surprising with regard to their close structural relationship and the fact that the isomerism found is characterized by only minor energy differences between the species. Thus, the main structural features can be shortly discussed for the three complexes together. 

It is evident that the chelating ligands are doubly deprotonated by the absence of the corresponding NH stretches in the infrared spectra of the compounds, but also by simple charge considerations. Consequently, the carbonyl bonds in the complexes are parts of the chelate rings. They range between 1.285 and 1.307 Å, which is in accord with their considerable bathochromic shifts of the IR frequency by almost 100 cm^−1^ compared with the value in the non-coordinated ligand (1680 cm^−1^ [44]). Similar shifts, which indicate a large extent of electron delocalization within the chelate rings, have been observed for other metal complexes after chelate formation with ligands of the type {L^R^}^2−^ [42,43,44,45,46]. 

Expectedly, the uranyl groups are practically linear and their bond lengths are in the normal range. More interesting are the orientations of these linear groups on the binuclear complexes to each other. We find a perfectly parallel arrangement in the *anti,anti*-isomer, while a marked tilting is visible for the *syn,syn* and *syn,anti*-complexes. This situation can be quantified by an analysis of the bonding situation in the central U_2_O_2_ diamonds. An evaluation of the angles established between the planes formed by the uranium atoms with the two bridging oxygen atoms gives values of 0° for the *anti,anti*-isomer of Figure 1, 15.4° for the *syn,syn* structure and 16.8° for the *syn,anti* compounds. The planar central unit in the first compound is of course the consequence of the crystallographic inversion center being located inside this unit, but this becomes only possible by the *anti*-conformation for the two bridging methanolato ligands, which does not produce unilateral steric stress above or below this plane. This, however, is clearly the case for the two isomers having the bridging ligands in *syn* arrangement. Consequently the uranyl units are slightly bent bringing the uranyl oxygen atoms of the sterically unencumbered side of the molecules closer together. The resulting O…O distances differ for up to 2 Å. The individual values for the three isomers are indicated in Figure 1. The uranium–uranium distances are not concerned by these structural differences (see Table 1).

The observed structural isomerization is not restricted to representatives with {L^Et2^}^2−^ ligands but has also been found for complexes with the morpholinoyl ligand {L^morph^}^2−^. The variation of the alkyl moiety of the thiourea group may influence the stability of the different conformations of the ligand and steric effects due to the substituents might obstructing the formation of the desired coordination compound. In this work, the chosen substituents are restricted to diethylamine and morpholinoyl groups. A morpholinoyl unit is a less flexible substituent than the NEt_2_ one and potentially provides an additional “hard” oxygen donor atom in its periphery. It has already been demonstrated that such a unit is suitable for the formation of intermolecular interactions in solid-state structures with the coordination of “hard” Ba^2+^ ions [42]. Thus, we found it interesting to see if such a behavior is also observed with the “hard” uranyl ions and conducted similar reactions between different uranyl starting materials, H_2_L^morph^, and different amounts of NEt_3_. Generally, the same behavior was observed as for H_2_L^Et2^, including different isomers of the dimeric complexes. Details and the structures of some representative morpholinoyl compounds are given in the Supplementary Materials. 

More interesting is the question, which species are present in solution? The behavior of both ligands strongly suggests a dissociative mechanism of the observed isomerization. Evidence for this assumption is given by the NMR spectra recorded for such solutions. The ^1^H-NMR spectra of (HNEt_3_)_2_[{UO_2_(L^Et2^)(µ-OMe)}_2_] in CDCl_3_ and (CD_3_)_2_SO indicate an interesting behavior of the complex in these two solvents. Only the signals of the deprotonated ligand {L^2a^}^2−^ and the counter ions {HNEt_3_}^+^ were observed, while those of the methanolato ligands could not be detected. This indicates a ready deconstruction of the dimeric complex in solution. The labile U-O(Me)-U bonds are broken and most probably, monomeric {UO_2_(L^2a^)(L)_n_}^0,–,2−^ complexes with L = solvent or deprotonated solvents, are formed. The missing signal for the coordinated methanolato ligands indicates a fast exchange, possibly also with other donor ligands such as OH¯ or H_2_O. Unlike the spectrum recorded in CDCl_3_, that measured in (CD_3_)_2_SO shows a remaining signal at 3.13 ppm, which can be assigned to methanol released from the dimeric starting complex. The isolation of a complex of the composition [UO_2_(L^Et2^)(DMSO)_2_] from such solutions [41] supports the conclusion that in this solvent a stable complex is formed, which also dominates in the solution. 

**Figure 3:**
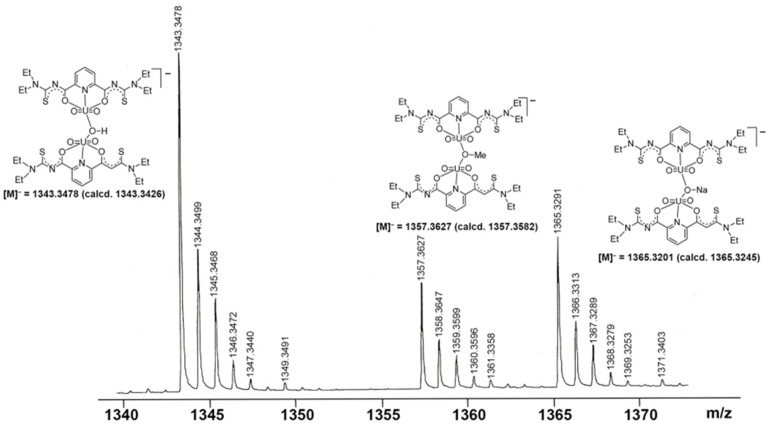
Fraction of the [M]^−^ region of the ESI(–) mass spectrum of (HNEt_3_)_2_[{UO_2_(L^Et2^)}_2_(µ-OMe)_2_] in MeOH showing signals assigned to dimeric species with different bridging ligands.

Further evidence for a dynamic equilibrium in solutions of the dimeric compounds is given by mass spectrometry. Irrespective of the isomers or counter ions used, ESI(–) mass spectra of the of [{UO_2_(L^Et2^)(µ-OMe)}_2_]^2−^ salts show fragmentation patterns containing fragments, which confirm the presence of a variety of monomeric and dimeric uranium-containing species. One or two bridging units are formed by the used solvents (e.g., methanol or methanolate), but also species with bridging hydroxido units have been detected. Figure 3 depicts a fraction of the [M]^−^ peaks of such a spectrum, in which peaks belonging to species such as [{UO_2_(L^Et2^)}_2_(µ-OH)]^−^, [{UO_2_(L^Et2^)}_2_(µ-OMe)]^−^, and [{UO_2_(L^Et2^)}_2_(µ-ONa)]^−^ are clearly resolved with the correct isotopic patterns. 

Summarizing the information, it must be concluded that solutions containing uranyl ions and H_2_L^R^ ligands can contain a variety of monomeric and dimeric species. The formation and potential reformation of the species depend on the solvents and pH of such solutions and the presence of potential counter ions for the precipitation of ionic complex species. Thus, the conformation of the ligands in solution as well as the nature of the bridging ligands are subjects of various equilibria, which should not at least also have consequences for reaction with additional metal ions as can be seen from Section 2.2 and Section 2.3.

### 2.2. Mixed-Metal Complexes with Gold and Lead 

The binuclear uranium complexes introduced in Section 2.1 possess uncoordinated thiourea sulfur atoms. Obviously, the “hard” uranyl ions prefer tridentate coordination via the central *O,N,O* donor set of the {L^R^}^2−^ ligands. A few examples of the general formula [UO_2_(L^R^)(solv)] (solv = H_2_O, MeOH, DMF) have been reported previously, in which the 2,6-dipicolinoylbis(*N,N*-dialkylthioureas) coordinate pentadentate [41]. In these products, however, they adopt another conformation and bind via their *S,N,N,N,S* donor atoms.

**Scheme 2:**
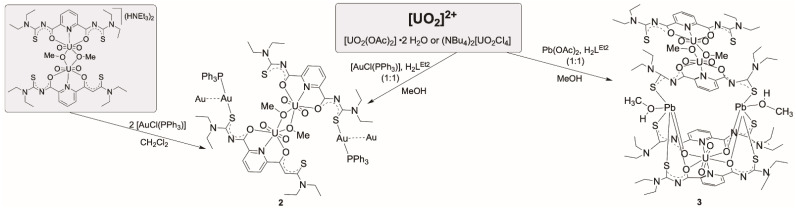
Reactions of [AuCl(PPh_3_)] and Pb(OAc)_2_ with H_2_L^Et2^ in the presence of uranyl compounds.

For potential mixed-metal complexes, the combination of “hard” uranyl ions with “soft” metal ions should be interesting. Typical representatives of the latter are Au^+^ or Pb^2+^ ions. From reactions of these ions with H_2_L^R^ ligands, hitherto no defined products could be isolated and structurally characterized, but there are several reports with related aroylthioureas including the parent, potentially *O,S*-bidentate benzoylthiourea ligand system, where exclusively S-coordination has been observed with gold ions [48,49,50,51], while chelate formation has been observed for Pb^2+^ ions [51,52,53]. Interesting, reactions patterns were observed when Au(I) complexes were exposed to H_2_L^R^ ligands together with other metal ions such as lanthanide or other M^3+^ ions, alkaline earth M^2+^ ions or transition metal M^2+^ ions. Large metallacoronands consisting of {(AuL^R^)*_n_*}*^n^*^−^ ring systems (*n* = 2–4) are formed, which accommodate the mentioned M^2+^ or M^3+^ metal ions in their center [46,54,55,56]. Recently, such compounds even found consideration for nuclear medical procedures using the radioactive isotope ^198^Au [57]. The reactions of common gold or lead starting materials with H_2_L^Et2^ conducted in the presence of uranyl compounds followed the previously observed reaction patterns and gave the mixed-metal complexes summarized in Scheme 2. 

**Figure 4:**
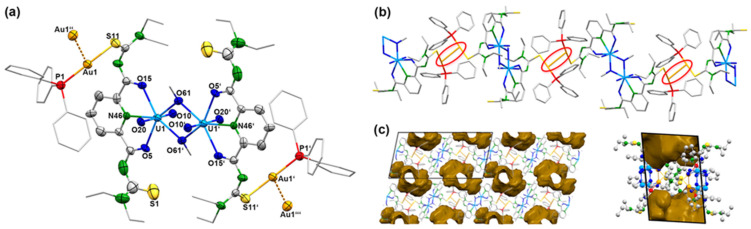
(**a**) Structure of the [{UO_2_(L^Et2^)(µ-OMe)}_2_{Au(PPh_3_)}_2_] monomer, (**b**) visualization of the linear polymer formed by Au…Au interactions (red circles), and (**c**) voids between the linear chains shown along the crystallographic *x*-axis (**left**) and *y*-axis (**right**). For color code see atomic labelling scheme.

Yellow needles of the composition [{UO_2_(L^Et2^)(µ-OMe)}_2_{Au(PPh_3_)}_2_] were isolated directly from a reaction mixture between the dimeric complex (HNEt_3_)_2_[{UO_2_(L^Et2^)(μ-OMe)}_2_] and two equivalents of [Au(PPh_3_)Cl] in CH_2_Cl_2_. The same compound can be obtained by a one-pot synthesis of equimolar amounts of H_2_L^Et2^, (NBu_4_)_2_[UO_2_Cl_4_], and [Au(PPh_3_)Cl] in MeOH. The use of an excess of [Au(PPh_3_)Cl] did not yield products with more than two sulfur atoms being coordinated to {Au(PPh_3_)}^+^ fragments. The IR spectrum of [{UO_2_(L^Et2^)(µ-OMe)}_2_{Au(PPh_3_)}_2_] shows the ν_C=O_ vibration at 1587 cm^−1^ and the ν_U=O_ band at 914 cm^−1^. The frequencies correspond to the values found in the dimeric complex (HNEt_3_)_2_[{UO_2_(L^Et2^)(μ-OMe)}_2_]. The presence of the triphenylphosphine ligands is confirmed by ^31^P-NMR spectroscopy showing one broad signal at 36.1 ppm. This corresponds to the chemical shift which is observed for the {Au(PPh_3_)}^+^ units in other triphenylphosphine gold(I) complexes [58,59,60]. The compound crystallizes in the triclinic space group P$\bar{1}$. The centrosymmetric molecule contains two *O,N,O* coordinated {UO_2_(L^2a^)} units, which are connected by two methanolato ligands establishing the conformation with inversion symmetry, which has been assigned in Section 2.1 as *anti,anti*. {Au(PPh_3_)}^+^ units coordinate to two opposite thiourea sulfur atoms. A structural representation of the resulting molecule is shown in Figure 4a. Details about the bond lengths shall not be discussed here since it is not justified by the quality of the crystallographic data set. A summary of the values is given as Supplementary Materials.

The bonding situation inside the central [{UO_2_(L^Et2^)(µ-OMe)}_2_]^2−^ unit of compound **2** shows only minor deviations from that in the parent anion **1c**. Each Au(I) ion is coordinated with one sulfur atom and the triphenylphosphine ligand. The S–Au–P bond angles are 178.5(3)°. In the solid-state structure of [{UO_2_(L^Et2^)(µ-OMe)}_2_{Au(PPh_3_)}_2_], one-dimensional polymers are formed by weak Au…Au contacts. Such attractive “aurophilic interactions” with Au...Au distances shorter than the sum of two van der Waals radii (≈3.80 Å) play a role in almost all areas of gold chemistry [61,62,63,64,65,66,67]. A comprehensive overview with a classification of such closed-shell interactions can be found in a review of Schmidbaur and Schier [68]. According to the classification used in the latter, the Au…Au interactions in compound **2** are “unsupported,” which means that the involved gold atoms are not additionally connected by bridging ligands or hydrogen bonding. Au…Au distances in such compounds are frequently larger than 3 Å and differ depending on the steric demands of the involved ligands. Figure 4b illustrates that such repulsing forces cannot be excluded for the compound under study; thus, the observed value of 3.21(1) Å for compound **2** is remarkably short. In the structurally related complex with tetraethyl-*N,N*-isophthaloylbis(thiourea), no aurophilic interactions could be found [51], while short Au…Au distances of 3.08 Å have recently been found for some [Au(PPh_3_)(thiazolidine thiourea)] derivatives [69]. The formation of polymeric chains with the sterically demanding ligands of compound **2** produced relatively large voids between the chains, which comprise almost 20% of the unit space. This is illustrated in Figure 4c. 

**Figure 5:**
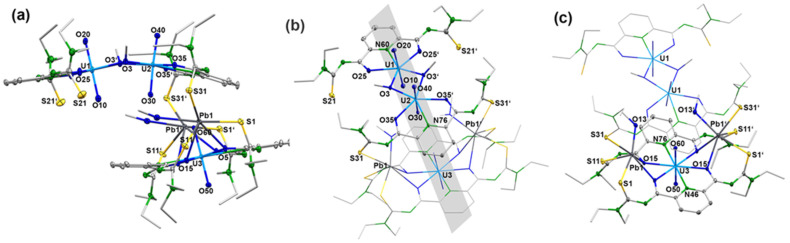
Structure of [Pb_2_(UO_2_)_3_(L^Et2^)_3_(µ-OMe)_2_(MeOH)_2_] (**3**): (**a**) a general view illustrating the orientation of the two molecular parts relative to each other, (**b**) a view highlighting the dimeric *syn,syn*-[{UO_2_(L^Et2^)(µ-OMe)}_2_]^2−^ unit together with the position of the crystallographic mirror plane and (**c**) a view highlighting the monomeric [UO_2_(L^Et2^)_2_]^2−^ unit together with the coordinated Pb^2−^ ions. For color code see atomic labelling scheme.

Reactions of Pb(OAc)_2_ × 2 H_2_O with each one equivalent of H_2_L^Et2^ and uranyl acetate in methanol give a yellow precipitate. The addition of a supporting base such as NEt_3_ is not required. As for the other uranyl complexes of this study, deprotonation of the chelating ligand is also indicated by the infrared spectrum of this product by the absence of the NH vibrations of H_2_L^Et2^. A significant bathochromic shift of the ν_CO_ band of the ligand strongly chelate formation with an extended delocalization of electron density. It appears in the complex at 1597 cm^−1^. Crystals suitable for X-ray analysis were obtained from a saturated CH_2_Cl_2_ solution of the yellow precipitate overlayered with MeOH. Figure 5 depicts the molecular structure of the complex. It possesses the unusual composition [Pb_2_(UO_2_)_3_(L^Et2^)_3_(µ-OMe)_2_(MeOH)_2_] (**3**) with two different coordination environments for the three uranium atoms contained. The molecular structure is composed of four deprotonated ligands {L^Et2^}^2−^, three uranyl ions, two Pb^2+^ ions, two methanolato ligands and two coordinated methanol molecules. This results in a neutral complex. All {L^Et2^}^2−^ ligands in compound **3** are coordinated to the uranyl ions tridentate with their *O,N,O* donor atoms. Interestingly, the molecule is built up of two different uranyl-containing subunits, which are connected by the Pb^2+^ ions as is shown in Figure 5. One subunit has the composition *syn,syn*-[{UO_2_(L^Et2^)(µ-OMe)}_2_]^2−^ (see Figure 1a). The second subunit has the composition {UO_2_(L^Et2^)_2_}^2−^. A crystallographic mirror plane divides the molecule into two identical parts. It is visualized in Figure 5b. Some selected bond lengths and angles are summarized in Table 2.

The two lead ions are coordinated monodentate with the sulfur atoms of the same {L^Et2^}^2−^ ligand of the dimeric [{UO_2_(L^Et2^)(µ-OMe)}_2_]^2−^ subunit, while with the monomeric subunit [{UO_2_(L^Et2^)_2_]^2−^ an *S,O* chelate formation is established, sharing the oxygen atoms with uranium. In contrast to the seven-coordinate uranium ions of the *syn,syn*-[{UO_2_(L^Et2^)(µ-OMe)}_2_]^2−^ subunit, in the monomeric subunit [UO_2_(L^Et2^)_2_]^2−^ a hexagonal-bipyramidal geometry around the uranium atom is found. The formation of *S,O* chelates of Pb^2+^ ions with aroylthioureas aligns with previous findings [52,53]. 

**Figure 6:**
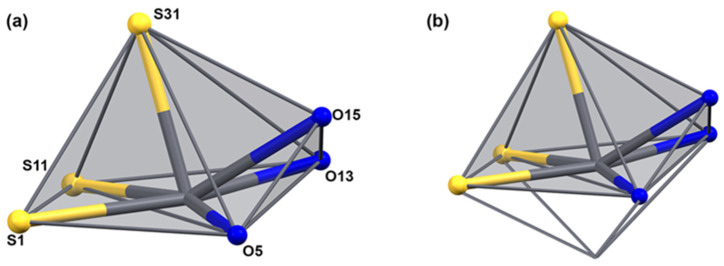
The coordination sphere of lead in [Pb_2_(UO_2_)_3_(L^Et2^)_3_(µ-OMe)_2_(MeOH)_2_] (**a**) without and (**b**) with consideration of the lone-pair of the Pb^2+^ ion. For color scheme see atomic labelling scheme in (**a**).

The uranyl bond lengths are unexceptional. The distances between the uranyl ions and the equatorial donor atoms are in the range between 2.294(3) and 2.471(3) Å for the oxygen atoms and between 2.529(6) and 2.656(5) Å for the nitrogen atoms. The coordination environment of lead is occupied by three sulfur atoms and three oxygen atoms and can be described as monocapped square-pyramidal. Under consideration of the stereoactive *6s* lone-pair electrons, which is indicated by the large void around the metal ions, the donor atoms are hemidirected and the description of the coordination sphere (including this lone pair) around the Pb^2+^ ion would correspond to a monocapped octahedron as has been observed previously for a number of lead(II) complexes [51,70,71,72,73] (see Figure 6). The Pb–S bond length is in the range of 2.720(6)–2.882(5) Å, and the Pb–O bond length is in the range between 2.685(6)–2.736(5) Å. As generally observed in hemidirected lead(II) compounds, the bond lengths of the donor atoms converging to the lone pair are somewhat elongated. In the present case, this concerns the Pb–S21, Pb–S11, and Pb–O25 bonds. The two intra-ligand bond angles around Pb are similar with approximately 77°. The inter-ligand bond angles, however, are very variable with values between 53.9(1)° and 166.8(1)°. This indicates a highly distorted geometry. The *S,O* chelate rings of {L^Et2^}^2−^ in the lead-containing subunit show an unprecedented deviation from the planarity in aroylbenzoylthioureato complexes. This is obviously due to the strongly bonded *O,N,O* chelate with uranium in the central parts of the corresponding subunits.

While the NMR spectra of compound **3** do not give a clear picture of the situation in solutions, some information can be derived from the respective ESI(+) mass spectra. There is no evidence for the molecular ion of the pentanuclear complex, but metal-containing fragments with considerable intensity are displayed in mass range *m*/*z* > 2100. They indicate a fragmentation pattern as has been observed for compound **1** by a ready cleavage of the methanolato bridges of the dimers, while the {UO_2_(L^Et2^)_2_}^2−^ unit in compound **3** seems to be more stable. Thus, the intense signals observed in the ESI(+) spectra of complex **3** at *m*/*z* = 2166.4376 and *m*/*z* = 2152.4218 can be assigned to fragments of the compositions [Pb_2_{UO_2_(L^Et2^)_2_}{UO_2_(L^Et2^)(OMe)}]^+^ (calcd. 2166.4408) and [Pb_2_{UO_2_(L^Et2^)_2_}{UO_2_(L^Et2^)(OH)}]^+^ (calcd. 2152.4252). Negative mode ESI(–) mass spectra support the assumption of the ready cleavage of the dimeric, µ-MeO-bridged building block by the detection of a signal at *m*/*z* = 2829.6021, which can be assigned to a singly bridged fragment of the composition [Pb_2_{UO_2_(L^Et2^)_2_}{UO_2_(L^Et2^)}_2_(OMe)]^+^ (calcd. 2829.6108). 

Summarizing the experiences with the structural variety found in the bimetallic gold/uranium and lead/uranium complexes supports the observations made for the dimeric uranyl complexes of Section 2.1 that minor modifications in the reaction conditions (stoichiometry, solvents, pH etc.) may have significant influences on the products obtained and that the structures determined for the isolated solids do not necessarily completely describe the situation in solution. This brought us to similar reactions with M^2+^ metal ions from the *3d* transition metal family. There exists some experience with mixed-metal complexes formed by such relatively “soft” metal ions in combinations with “hard” metal ions coming from the groups of lanthanides, alkali metals or alkaline earth metals [42,43,45,74,75,76,77]. For uranium, such experiments are not yet known.

### 2.3. Mixed-Metal Complexes with Various 3D Metal Ions

**Scheme 3:**
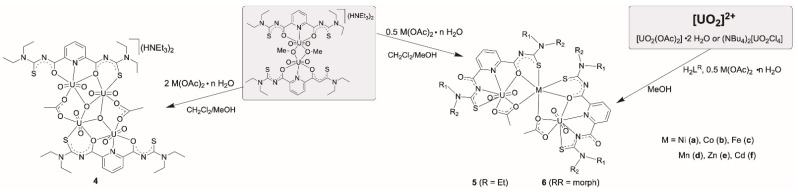
Reactions of various uranyl starting materials with H_2_L^R^ ligands and transition metal acetates.

The nature of the products obtained from mixtures of H_2_L^R^ ligands, uranyl compounds and M^2+^ transition metal acetates is dependent on the ratio of the reactants, particularly on the amount of the metal acetates (Scheme 3). The addition of a two-fold excess of M(II) acetates (M = Ni, Co, Fe, Mn, Zn or Cd) dissolved in methanol to a dichloromethane solution of (HNEt_3_)_2_[{UO_2_(L^2a^)(μ_2_-OMe)}_2_] gave an orange-red precipitate. The product precipitates immediately from the reaction mixture and is almost insoluble in common solvents, which restricts the analytical methods. The IR and elemental analysis data reveal a compound with a composition, which is different from that of the dimeric starting complex.

**Figure 7:**
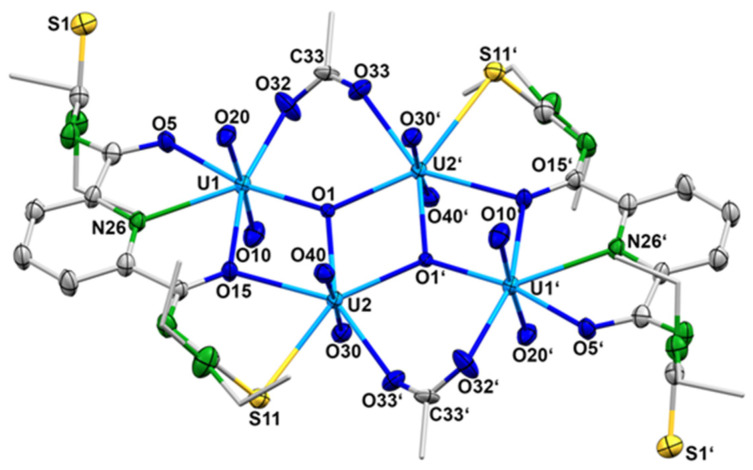
Structure of the complex anion **4**. For color codes see atomic labelling scheme.

The exact composition of the product was determined by X-ray diffraction on single crystals obtained by slow evaporation of the reaction mixture after filtering off the immediately formed solid. IR spectra and elemental analysis confirmed the identity of the crystals with the separated orange-red powder. Surprisingly, no mixed-metal complex was formed under the conditions applied. Obviously, the excess of metal acetate increased the pH of the reaction mixture to the level that the formation of oligonuclear uranyl aggregates is preferred and a sparingly soluble complex of the composition (HNEt_3_)_2_[{(UO_2_)_2_(L^Et2^)(µ_2_-OAc)(µ_3_-O)}_2_] ({HNEt_3_}2[**4**]) precipitated. Figure 7 shows the molecular structure of the [{(UO_2_)_2_(L^2a^)(µ_2_-OAc)(µ_3_-O)}_2_]^2−^ anion. Selected bond lengths and angles are summarized in Table 3. 

The compound crystallizes in the centrosymmetric, triclinic space group P$\bar{1}$ with one- half of the molecule in the asymmetric unit. The [{(UO_2_)_2_(L^Et2^)(µ_2_-OAc)(µ_3_-O)}_2_]^2−^ anion consists of four uranyl cations and two {L^Et2^}^2−^ ligands, which are coordinated in a tetradentate *O,N,O,S* fashion. Each ligand coordinates two uranyl ions and the resulting sub-units are bridged by two µ_3_-oxido and two bidentate-coordinated acetato ligands. This results in a diamond-like arrangement of the four uranium atoms. The inversion center is located in the center of the rhombus formed by the atoms U2, U2′, O1 and O1′ (Figure 7). Each uranium atom establishes a pentagonal-bipyramidal geometry. The uranium atoms U1 and U1′ are coordinated by two oxygen atoms and the pyridine nitrogen atoms of {L^Et2^}^2−^, one oxygen atom of the acetato ligand and the *µ*_3_-oxido ligand. The uranium atoms U2 and U2′ are coordinated by the *S,O* chelate ring of the {L^Et2^}^2−^ ligands, one oxygen atom of the acetato ligand and two *µ*_3_-oxido ligands. It should be mentioned that the coordinated sulfur atom S11 is subject of a partial hydrolysis and the structure is best refined with a S/O disorder of 90/10. The hydrolysis of thiourea functionalities in {L^R^}^2−^ ligands is not without precedence and has been found for other uranium complexes, where a complete S/O exchange obviously facilitates the coordination of such ligands to the “hard” {UO_2_}^2+^ ions [78]. Since in the present example such an exchange is observed to a minor degree, it should not be subject of an extended discussion. Nevertheless, the formation of the tetranuclear uranium complex nicely illustrates the structural flexibility of the products, which can be isolated from {UO_2_}^2+^/H_2_L^R^/metal acetate reaction mixtures and the strong dependence of the preferred products on the pH value in such solutions. Further support for this assumption is given by slight variations in the reaction conditions in such mixtures.

Finally, the formation of heterometallic uranyl compounds of the composition [M{UO_2_(L^R^)(OAc)}_2_] (R = Et: **5**, RR = morph: **6**) (M = Ni^2+^, Co^2+^, Fe^2+^, Mn^2+^, Zn^2+^ or Cd^2+^) was achieved by decreasing the amount of metal acetate to 0.5 equivalent per equivalent of the uranyl source (Scheme 3). This is most probably due to a well-balanced lowering of the pH in such solutions. Interestingly, the addition of a supporting base such as NEt_3_ to such reaction mixtures induces the formation of the previously described dimeric complexes (HNEt_3_)_2_[{UO_2_(L^R^)(µ-OMe)}_2_] (**1**).

The neutral mixed-metal complexes **5** or **6** can be obtained from CH_2_Cl_2_/MeOH reaction mixtures of the dimeric complexes (HNEt_3_)_2_[{UO_2_(L^R^)(µ-OMe)}_2_] (**1**) with the transition metal acetates or from one-pot reactions starting directly from the transition metal acetates, H_2_L^R^ and uranyl acetate in methanol. They precipitate from the reaction solutions and can be crystallized from CH_2_Cl_2_/MeOH mixtures. The formation of the heterometallic complexes is easily verified by IR spectroscopy. In contrast to the dimeric complexes **1**, the heterometallic complexes show four carbonyl absorption bands, clearly indicating the presence of differently arranged carbonyl units (belonging to picolinoylthioureato and/or acetato ligands) in the products. The asymmetric uranyl stretches appear in the spectra of the products between 916–924 cm^−1^, which corresponds to a moderate shift compared to the position in compound **1** (910 cm^−1^). 

**Figure 8:**
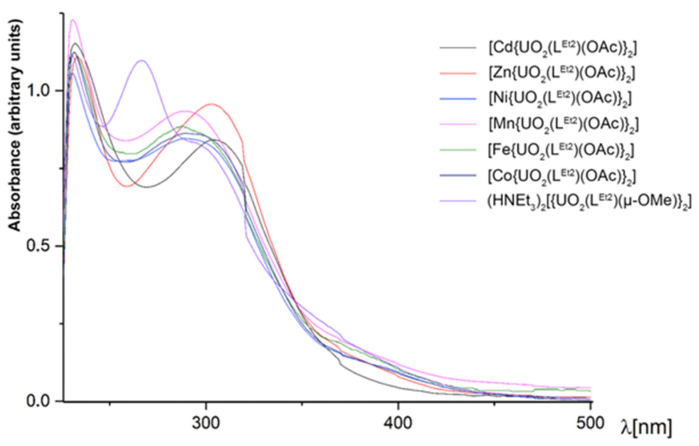
UV/vis spectra of some [M{UO_2_(L^Et2^)(OAc)}_2_] complexes in CH_2_Cl_2_. The spectrum of (HNEt_3_)_2_[{UO_2_(L^Et2^)(µ-OMe)}_2_] is included for comparison.

The colors of the heterometallic compounds are slightly different depending on the transition metal contained. The complexes with Zn^2+^ and Cd^2+^ are pale yellow; the Ni^2+^ and Mn^2+^ complexes are deep yellow or light orange, while the complexes with Fe^2+^ and Co^2+^ are brown. The UV/vis spectra of some [M{UO_2_(L^Et2^)(OAc)}_2_] complexes are shown in Figure 8. For comparison, the electronic spectrum of the dimeric complex (HNEt_3_)_2_[{UO_2_(L^Et2^)(µ-OMe)}_2_] is included in the figure. The spectra of the heterometallic complexes with the transition metal ions Ni^2+^, Mn^2+^, Fe^2+^, and Co^2+^ are similar with three main absorptions around 230, 290, and 370 nm. The complexes with the closed-shell metal ions Cd^2+^ and Zn^2+^ are slightly different with a red shift of the absorption at 290 nm, which appears around 305 nm in both complexes. The presence of the transition metal in the complexes is indicated by the absence of absorption at 265 nm.

NMR spectra of the diamagnetic representatives and ESI mass spectra of the complexes **6** and **7** are helpful tools to obtain information about the molecular dynamic in solution. Representative spectra are shown as Supporting Material. The detection of molecular ions in the ESI(+) mass spectra of the heterobimetallic complexes indicates a relatively high stability of the compounds, which allows a transfer to the gas without decomposition. Such a behavior is in contrast to that of the bi- and oligonuclear uranyl complexes with the same ligands discussed vide supra.

**Figure 9:**
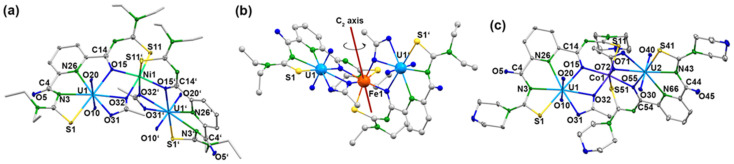
Structures of (**a**) [Ni{UO_2_(L^Et2^)(OAc)}_2_] with the atomic labeling scheme for the complexes having a rotational axis, (**b**) [Fe{UO_2_(L^Et2^)(OAc)}_2_] with the orientation of the C_2_ axis and (**c**) [Co{UO_2_(L^morph^)(OAc)}_2_] with the atomic labeling scheme for the complexes without rotational axis. For color code see atomic labelling scheme.

Single crystals suitable for X-ray diffraction were obtained for the trinuclear complexes of Ni^2+^ (compound **5a**), Co^2+^ (compound **5b**), Fe^2+^ (compound **5c**), and Mn^2+^ (compound **5d**) with {L^Et2^}^2−^_._ Additionally, the solid-state structures of such compounds with Ni^2+^ (compound **6a**), Co^2+^ (compound **6b**), and Zn^2+^ (compound **6e**) containing H_2_L^morph^ have been studied. Figure 9 depicts representative structures of the complexes, and selected bond lengths are summarized in Table 4. The corresponding values of complexes **6a** and **6e** are not contained in the table since the quality of the X-ray data does not justify a detailed discussion of individual bond lengths and angles. But they clearly support a general structural evaluation of general bonding features. Corresponding figures and tables are contained in the Supporting Material for inspection.

Although their molecular structures are very similar, two different modes of crystallization are observed for the [M{UO_2_(L^R^)(OAc)}_2_] complexes. Some of the compounds (**5a**, **5b**, and **5c**) crystallize in the C-centered space group C2/c with similar unit cell parameters (Figure 9a). Their asymmetric units each contain half of the complex molecule, and the second half is generated by a twofold axis (Figure 9b), on which the respective transition metal ion is located. For another group of complexes (**5d**, **6a**, **6b**, and **6e**), obviously, the high local symmetry could not be established, and they crystallize with complete [M{UO_2_(L^R^)(OAc)}_2_] molecules in their asymmetric unit. An example containing the atomic labeling scheme for this group of compounds is shown in Figure 9c with the structure of [Co{UO_2_(L^morph^)(OAc)}_2_]. 

Independent of their mode of crystallization and their peripheral residues (NEt_2_ or morpholinyl groups), the [M{UO_2_(L^R^)(OAc)}_2_] molecules are composed of two {UO_2_(L^R^)(OAc)}^−^ units, which are linked to each other by the transition metal M^2+^ ion. Each uranium atom is coordinated in a tetradentate manner with the *S,N,N,O* donor sets of a {L^R^}^2−^ ligand. The hexagonal equatorial coordination spheres of the uranyl ions are completed by acetato ligands. The axial U=O bond lengths are unexceptional. The equatorial U–O bond lengths are in the range between 2.449(3) and 2.535(4) Å. The U–N distances span from 2.460(4) to 2.562(5) Å, while the uranium–sulfur bond lengths are between 2.854(2) and 2.886(1) Å. The equatorial coordination spheres of the two uranium atoms are almost perfectly planar (with minor deviations due to the acetate ligands) and are arranged perpendicular to each other.

The central M^2+^ ions are coordinated with two *S,O* chelating units of the {L^R^}^2−^ ligands and two oxygen donor atoms of the acetato ligands. This results in distorted octahedral coordination spheres. The M–O and M–S bond lengths are in the range of the values observed in other octahedral M^2+^ chelates with aroylbisthioureato ligands [42,45]. The uranium and transition metal ions are connected by the carbonyl groups of one *S,O* chelating unit of the {L^R^}^2−^ ligands and the acetato ligands. This brings them relatively close together with uranium–M^2+^ distances between 3.702(1) and 3.926(1) Å. The uranium–uranium distances are between 6.1 and 6.8 Å. Interestingly, the longest metal–metal distances are found for the representatives without symmetrical restraints (**5d**, **6b**), (see Table 4). 

**Figure 10:**
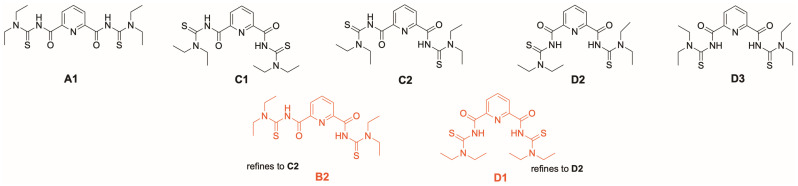
Stable molecular conformations of the free ligand H_2_L^Et2^. Two unstable configurations of the free ligand that have been observed in transition metal complexes of the ligand are additionally provided in red. Further details are given as Supporting Information.

An interesting structural feature of complexes **5** and **6** is the coordination mode established by the {L^R^}^2−^ ligands. Unlike the situations in complexes **1**, **2**, **3** and **4**, an *S,N,N,O* donor atom set is used for the coordination of the uranium atoms and one of the carbonyl oxygen atoms remains uncoordinated. Such a behavior may underline the structural flexibility of the potentially pentadentate ligands and their ability to adopt the sterical and electronic demands of the metal ions as well as their preferred coordination modes, but also arises the question for the inherently preferred conformation. To determine the most stable conformers, the geometries were optimized at the B3LYP/6-311G level of theory and the energies of the final geometries were calculated at the PBE0-GD3BJ/def2-TZVPPD. The results indicate that the conformation suitable for potential *N,N,N* or *S,N,N,N,S* chelate formation is the thermodynamically most stable one within the specified energy differences between the conformers. Stable molecular conformations of H_2_L^Et2^ and those observed in the solid-state structures of its transition metal complexes are shown in Figure 10.

This information is somewhat misleading with regard to the coordination chemistry with deprotonated {L^Et2^}^2−^ ligands. Four out of the five conformers shown in Figure 10 can establish one (conformers **B2** and **C2**) or two (conformers **D1** and **D3**) hydrogen bonds between the amide units and the central pyridine nitrogen atom. As stated for the conformers of the uranium complexes above, the relatively small energetic differences between conformers could easily be overcome by solvation, packing effects or, as in this case, hydrogen-bonding, all of which may play a key role in the evaluation of their relative thermodynamic stability. Indeed, such hydrogen bonding has been found in the crystal structure of the compound [44]. Thus, it might be more instructive to have a look at the situation of the related isophthaloylbis(*N,N*-diethylthiourea), which has a central phenyl ring instead of pyridine and, consequently, for which such NH…N interactions cannot be formed. Analog DFT calculations for the related conformers effectively confirm the analogous structure to **A1** as the most stable isomer [41]. A similar sequence should also be expected for the deprotonated ligand {L^Et2^}^2−^ and explains that most of the hitherto structurally studied complexes with this ligand and “hard” metal ions establish a neutral *O,N,O* coordination. Metal complexes with the conformations **B2**, **C2** and **D3** have also been reported [42,43]. Examples for the pentadentate coordination with conformer **D3** are known for rhenium and technetium complexes [79,80]. The same coordination mode has recently also been found for monomeric uranyl complexes [UO_2_(L^Et2^)(solv)] (solv = MeOH, DMF) [41].

**Scheme 4:**
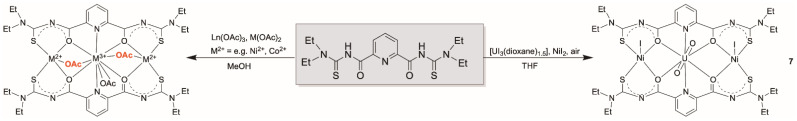
Formation of [LnM^II^(L^Et2^)_2_(OAc)_3_] complexes [45] and [(UO_2_)(NiI)_2_(L^Et2^)_2_].

Most of the hitherto studied bimetallic complexes with {L^R^}^2−^ ligands and M^2+^ and M^3+^ metal ions have been prepared from metal acetates and a screening of their structures confirms that almost all of them contain acetato co-ligands. With the exceptions of the [MM’(L^R^)_3_]^0,+^ complexes [42,43,44,76,81] and a few examples, in which the coordination spheres of the transition metal ions are completed by solvent molecules [42], the acetato ligands act as bridges between the different metal ions. A typical example is shown in Scheme 4 with the structure established with lanthanide and M^2+^ ions [45]. 

**Figure 11:**
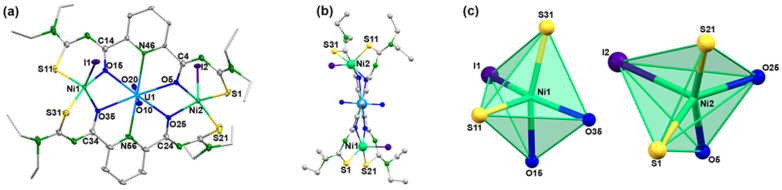
Molecular structure of [(UO_2_)(NiI)_2_(L^Et2^)_2_] (**7**): (**a**) general view with atomic labeling scheme, (**b**) view along the N46-U1-N56 axis, (**c**) coordination polyhedral of the Ni atoms. For color code see atomic labelling scheme in (**a**).

Acetato bridges also play a role in the mixed-metal complexes **5** and **6** of the present study (Scheme 3). They fill the coordination spheres of the *S,N,N,O* coordinated {UO_2_(L^R^} fragments and connect them with the central M^2+^ ions. It would be interesting to see, if similar reactions also proceed with acetate-free starting materials and which structures are stabilized. Thus, we conducted a first, preliminary reaction between H_2_L^Et2^, the low-valent uranium compound [UI_3_(dioxane)_1.5_] and nickel iodide in methanol. Since the reaction was conducted in air, uranium was oxidized and the resulting uranyl product, [(UO_2_)(NiI)_2_(L^Et2^)_2_] (**7**), precipitated directly from the reaction mixture. It contains one central uranyl unit, which is coordinated by the *O,N,O* donor atom sets of two {L^Et2^}^2−^ ligands, and two five-coordinate Ni(II) ions. The equatorial coordination spheres of the transition metal ions are occupied by two *S,O* chelating units of the picolinoylbis(thioureas). An ellipsoid representation of the resulting complex structure is shown in Figure 11 and some bond lengths are summarized in Table 5.

The bond lengths in compound **7** are unexceptional and similar to those found in the other uranium complexes with {L^Et2^}^2−^ ligands. An interesting feature, however, is given with the distortions inside the chelating ligands. In contrast to the situation in the previously discussed complexes with essentially planar ligands, the sulfur atoms in compound **7** show marked deviations from the planes of the central pyridine rings, as illustrated in Figure 11b. The lower symmetry in [(UO_2_)(NiI)_2_(L^Et2^)_2_] is also expressed by two different uranium-nickel differences and by clear differences in the coordination environments of the nickel atoms. Both Ni^2+^ ions are five-coordinate, but the established coordination polyhedra are clearly different as can be seen in Figure 11c. A more quantitative evaluation becomes available by an analysis by means of the Continuous Shape algorithm [82,83,84,85], which allows the evaluation of the bonding situations, even in the case of simple visualization giving no unambiguous results. Five polyhedra are relevant for the coordination number five, but most coordination compounds establish a trigonal bipyramid or square pyramid as the two Ni^2+^ ions in compound **7**. While the environment of Ni1 is intermediate between these two shapes with continuous shape measures of 3.394 for a trigonal bipyramid and 3.169 for a square pyramid, a clear preference for a square pyramid with the iodine atom as the apex (continuous shape measure 1.892) is found for the nickel atom Ni2 (value for a trigonal bipyramid: 3.394). 

It should be interesting to extend this work to other acetate-free precursors in order to obtain a more complete overview about the structural chemistry of such uranium-containing mixed-metal complexes.

## 3. Materials and Methods

Unless otherwise stated, reagent-grade solvents and starting materials were used. Solvents were dried and distilled prior to use. H_2_L^Et2^ and H_2_L^morph^ were prepared according to a literature procedure [86].

### 3.1. Radiation Precaution

All synthetic work with uranium was performed in a laboratory approved for the handling of radioactive material. All personel working on this project were permanently monitored for potential contaminations.

### 3.2. Physical Measurements

IR spectra were measured as KBr pellets on a Shimadzu IR Affinity-1 spectrometer (Shimadzu, Kyoto, Japan). The NMR spectra were recorded on JEOL 400 MHz spectrometers (JEOL, Kyoto, Japan). ESI mass spectra were measured with an Agilent 6210 ESI-TOF (Agilent Technology, Santa Clara, CA, USA) spectrometer. All MS results are given in the form of *m*/*z* assignment. Elemental analysis of carbon, hydrogen, nitrogen, and sulfur was determined using a Heraeus vario EL elemental analyzer (Elementar, Langensebold, Germany). 

### 3.3. Syntheses

*(HNEt_3_)_2_[{UO_2_(L^Et2^)(µ-OMe)}_2_]* ((HNEt_3_)_2_[**1**]). H_2_L^Et2^ (20 mg, 0.05 mmol) was dissolved in MeOH (3 mL) at room temperature and added to a stirred solution of (NBu_4_)_2_[UO_2_Cl_4_] (90 mg, 0.1 mmol) or UO_2_(CH_3_COOH)_2_ × 2 H_2_O (42 mg, 0.1 mmol) in MeOH (3 mL). After 10 min, 2 drops of NEt_3_ were added and the reaction mixture was stirred for 1 h. The obtained precipitate was filtered off, washed with MeOH and dried under vacuum. Single crystals for X-ray diffraction containing the *syn,syn* conformer of the anion were obtained after evaporation of a CH_2_Cl_2_/MeOH (1:2, *v*/*v*) solution at room temperature. Yield: 74% (59 mg). Elemental analysis: Calcd. for C_48_H_84_N_12_O_10_S_4_U_2_: C, 35.8; H, 5.2; N, 10.7; S, 8.1%. Found: C, 35.6; H, 5.23; N, 10.4; S, 8.1%. IR (KBr, cm^−1^): 3429(m), 3070(w), 2974(m), 2933(w), 2874(w), 2681(w), 1589 (vs), 1497(m), 1462(w), 1425(m), 1382(s), 1309(m), 1278(m), 1247(s), 1201(w), 1145(m), 1120(m), 1070(m), 1016(m), 950(w), 912 (vs), 868(w), 766(s), 678(w), 638(m). ^1^H NMR (400 MHz, DMSO, ppm): 8.50 (d, 4H, *J* = 8.0 Hz, py); 8.41 (t, 2H, *J* = 8.0 Hz, py); 3.95 (q, 8H, *J* = 7.0 Hz, CH_2_); 3.53 (q, 8H, *J* = 7.0 Hz, CH_2_); 2.37 (q, 12H, *J* = 7.2 Hz, CH_2__HNEt3); 1.24 (t, 12H, *J* = 7.0 Hz, CH_3_); 1.02 (t, 12H, *J* = 7.0 Hz, CH_3_); 0.87 (t, 18H, J =7.2 Hz, CH_3__HNEt3). UV/Vis (CH_2_Cl_2_, nm): 230 (ε = 8.3 × 10^3^ L mol^−1^ cm^−1^), 265 (ε = 7.8 × 10^3^ L mol^−1^ cm^−1^), 304 (ε = 4.9 × 10^3^ L mol^−1^cm^−1^), 368 (ε = 1.2 × 10^3^ L mol^−1^ cm^−1^).

*(EtPPh_3_)_2_[{UO_2_(L^Et2^)(µ-OMe)}_2_]* ((EtPPh_3_)_2_[**1**]). A solution of (EtPPh_3_)Cl (33 mg, 0.1 mmol) in MeOH (2 mL) was added dropwise to a solution of (HNEt_3_)_2_[{UO_2_(L^Et2^)(µ-OMe)}_2_] (159 mg, 0.1 mmol) in CH_2_Cl_2_ (2 mL). The reaction mixture was evaporated slowly at room temperature. The obtained yellow crystals were collected, washed with MeOH and dried under vacuum. Yield: 12% (24 mg). Elemental analysis: Calcd. for C_76_H_92_N_10_O_10_P_2_S_4_U_2_: C, 46.3; H, 4.7; N, 7.1; S, 6.5%. Found: C, 45.9; H, 4.9; N, 7.1; S, 6.5%. IR (KBr, cm^−1^): 3425(m), 3057(w), 2974(m), 2929(m), 2855(w), 2810(w), 2601(w), 1591(vs), 1492(s), 1433(s), 1377(s), 1311(m), 1280(m), 1248(s), 1146(w), 1113(s), 1001(m), 949(w), 905(vs), 843(w), 758(m), 691(m), 631(w), 530(m). UV/Vis (CH_2_Cl_2_, nm): 232 (ε = 6.3 × 10^3^ L mol^−1^cm^−1^), 264 (ε = 5.3 × 10^3^ L mol^−1^cm^−1^), 304 (ε = 3.8 × 10^3^ L mol^−1^cm^−1^), 358 (ε = 0.9 × 10^3^ L mol^−1^cm^−1^).

[{UO_2_(L^Et2^)(µ-OMe)}{Au(PPh_3_}_2_] (**2**). A solution of [Au(PPh_3_)Cl] (50 mg, 0.1 mmol) in CH_2_Cl_2_ (2 mL) was added dropwise to a solution of (HNEt_3_)_2_[{UO_2_(L^Et2^)(µ-OMe)}_2_] (80 mg, 0.05 mmol) in CH_2_Cl_2_ (2 mL). The reaction mixture was stirred for 1 h at room temperature, MeOH (2 mL) was added and the mixture was allowed to evaporate slowly at room temperature. The obtained yellow crystals were collected, washed with MeOH and dried under vacuum. Yield: 85% (96 mg). Elemental analysis: Calcd. for C_72_H_82_Au_2_–N_10_O_10_P_2_S_4_U_2_: C, 37.5; H, 3.6; N, 6.1; S, 5.6%. Found: C, 36.9; H, 3.5; N, 5.9; S, 5.5%. IR (KBr, cm^−1^): 3445(m), 3051(w), 2974(m), 2932(m), 2872(w), 1587(vs), 1562(vs), 1431(vs), 1393(vs), 1358(w), 1310(m), 1281(m), 1248(s), 1202(m), 1146(m), 1121(m), 1099(s), 1072(w), 1020(m), 949(w), 914(vs), 845 (m), 756(vs), 692(vs), 635(m), 538(vs), 505(vs). ^1^H NMR (DMSO-D_6_, ppm): 8.23 (d, 4H, *J* = 7.6 Hz, py); 7.64 (t, 2H, *J* = 7.6 Hz, py); 7.41–7.07 (m, 30H, Ph); 4.21–3.72 (m, 16H, CH_2_); 3.09 (s, 3H, CH_3_O); 1.67–1.21 (m, 24H, *J* = 7.5 Hz, CH_3_). ^31^P NMR (DMSO-D_6_, ppm): 36.1. UV/Vis (CH_2_Cl_2_, nm): 230 (ε = 5.3 × 10^3^ L mol^−1^ cm^−1^), 251 (ε = 4.2 × 10^3^ L mol^−1^cm^−1^), 266 (ε = 3.6 × 10^3^ L mol^−1^cm^−1^), 305 (ε = 1.8 × 10^3^ L mol^−1^cm^−1^), 366 (ε = 0.5 × 10^3^ L mol^−1^cm^−1^).

[Pb_2_(UO_2_)_3_(L^Et2^)_4_(MeOH)_2_(µ-OMe)_2_] (**3**). A solution of Pb(OAc)_2_ ∙ 2 H_2_O (33 mg, 0.1 mmol) in MeOH (1 mL) was added to a solution of UO_2_(OAc)_2_ ∙ 2 H_2_O (43 mg, 0.1 mmol) in MeOH (2 mL). H_2_L^Et2^ (40 mg, 0.1 mmol) was added and the solution was stirred at room temperature for 1 h. The formed precipitate was filtered off, washed with MeOH and Et_2_O and dried in vacuum. Single crystals for X-ray diffraction were obtained after slow evaporation of a CH_2_Cl_2_/MeOH (1:1, *v*/*v*) solution at room temperature. Yield: 70% (56 mg). Elemental analysis: Calcd. for C_75_H_118_N_20_O_21_S_8_U_3_Pb_2_ ([Pb_2_(UO_2_)_3_(L^2a^)_4_(MeOH)_2_(µ-OMe)_2_] · 3 MeOH): C, 29.2; H, 3.6; N, 9.3; S, 8.5%. Found: C, 28.2; H, 3.7; N, 9.4; S, 8.5%. IR (KBr, cm^−1^): 3443(m), 3084(w), 2974(m), 2934(m), 2874(w), 1597(vs), 1566(vs), 1514(vs), 1429(vs), 1385(vs), 1362(w), 1310(m), 1285(m), 1252(s), 1206(m), 1148(m), 1099(m), 1074(m), 1015(m), 955(w), 916(vs), 845 (m), 758 (s), 696(m), 638(w). ^1^H NMR (CD_2_Cl_2_, ppm): 8.91–7.88 (m, 12H, py); 4.25–4.15 (m, 8H, CH_2_); 3.40–3.53 (m, 32H, CH_2_); 3.47 (m, 12H, MeOH); 1.59–0.93 (m, 48H, CH_3_). UV/Vis (CH_2_Cl_2_, nm): 231 (ε = 7.6 × 10^3^ L mol^−1^ cm^−1^), 258 (ε = 7.6 × 10^3^ L mol^−1^cm^−1^), 296 (ε = 5.5 × 10^3^ L mol^−1^ cm^−1^), 369 (ε = 1.1 × 10^3^ L mol^−1^ cm^−1^).

*(HNEt_3_)_2_[{(UO_2_)_2_(L^Et2^)(µ_2_-OAc)(µ_3_-O)}_2_]* (**4**). A solution of M(OAc)_2_ ∙ n H_2_O (0.2 mmol) in MeOH (1 mL) was added to a stirred solution of UO_2_(OAc)_2_ ∙ 2 H_2_O (42 mg, 0.1 mmol) in MeOH (2 mL). H_2_L^Et2^ (40 mg, 0.1 mmol) was added and the solution was stirred at room temperature for 1 h. The formed precipitate was filtered off, washed with MeOH and dried in vacuum. Single crystals were obtained from slow evaporation of the reaction mixture after removal of the precipitate. Yield (60%). Elemental analysis: Calcd. for C_50_H_84_N_1_2O_18_S_4_U_4_: C, 27.03 H, 3.81; N, 7.57; S, 5.77%. Found: C, 25.72; H, 3.75; N, 7.13; S, 5.28%. IR (KBr, cm^−1^): 3445(m), 3032(w), 2976(m), 2933(w), 2873(w), 2812(w), 2742(w), 1593(vs), 1552(vs), 1525(w), 1429(s), 1385(vs), 1355(w), 1307(w), 1280(m), 1250(s), 1151(m), 1120(m), 1072(m), 1016(m), 954(w), 907 (vs), 872(m), 843(w), 791(w), 759(m), 654(m), 503(m).

*[M{UO_2_(L^R^)(OAc)}_2_] complexes* (**5** and **6**), general procedure. A solution of M(OAc)_2_ ∙ n H_2_O (0.05 mmol) in MeOH (1 mL) was added to a stirred solution of UO_2_(OAc)_2_ ∙ 2 H_2_O (42 mg, 0.1 mmol) in MeOH (2 mL). H_2_L^Et2^ (40 mg, 0.1 mmol) or H_2_L^morph^ (42 mg, 0.1 mmol) was added and the solution was stirred at room temperature for 1 h. The formed precipitate was filtered off, washed with MeOH and Et_2_O and dried in vacuum. Single crystals for X-ray diffraction were obtained by slow evaporation of a CH_2_Cl_2_/MeOH (1:1, *v*/*v*) solution at room temperature. 

*[Ni{UO_2_(L^Et2^)(OAc)}_2_]* (**5a**). Yield: 70% (53 mg). Elemental analysis: Calcd. for C_40_H_58_Cl_2_N_11_O_12_S_4_U_2_Ni ([Ni{UO_2_(L^Et2^)(OAc)}_2_] · 2 CH_2_Cl_2_ · H_2_O): C, 28.7; H, 3.4; N, 8.4; S, 7.7%. Found: C, 28.7; H, 3.4; N, 7.3; S, 7.7%. IR (KBr, cm^−1^): 3445(m), 3084(w), 2976(m), 2936(m), 2874(w), 1653(s), 1597(vs), 1558(vs), 1518(vs), 1427(vs), 1385(vs), 1348(s), 1314(m), 1290(m), 1256(s), 1206(m), 1149(m), 1082(m), 1013(m), 955(m), 922(vs), 868 (m), 843(m), 762(s), 685(m), 662(w). UV/Vis (CH_2_Cl_2_, nm): 229 (ε = 13.1 × 10^3^ L mol^−1^ cm^−1^), 289 (ε = 10.4 × 10^3^ L mol^−1^cm^−1^), 369 (ε = 1.9 × 10^3^ L mol^−1^cm^−1^).

*[Co{UO_2_(L^Et2^)(OAc)}_2_]* (**5b**). Yield: 78% (59 mg). Elemental analysis: Calcd. for C_40_H_56_Cl_4_N_10_O_12_S_4_U_2_Co ([Co{UO_2_(L^Et2^)(OAc)}_2_] · 2 CH_2_Cl_2_): C, 28.7; H, 3.4; N, 8.4; S, 7.6%. Found: C, 28.9; H, 3.6; N, 8.2; S, 7.5%. IR (KBr, cm^−1^): 3443(m), 3078(w), 2978(m), 2936(m), 2874(w), 1651(s), 1595(vs), 1560(vs), 1516(vs), 1427(vs), 1387(vs), 1346(s), 1314(w), 1290(m), 1258(s), 1206(m), 1149(m), 1086(m), 1015(m), 955(m), 922(vs), 866 (m), 843(m), 760(s), 685(m), 662(w). UV/Vis (CH_2_Cl_2_, nm): 230 (ε = 12.3 × 10^3^ L mol^−1^cm^−1^), 292 (ε = 10.5 × 10^3^ L mol^−1^cm^−1^), 369 (ε = 1.9 × 10^3^ L mol^−1^cm^−1^).

*[Fe{UO_2_(L^Et2^)(OAc)}_2_]* (**5c**). Yield 56% (42 mg). Elemental analysis: Calcd. for C_38_H_58_N_10_O_15_S_4_U_2_Fe ([Fe{UO_2_(L^Et2^)(OAc)}_2_]·3 H_2_O): C, 29.4; H, 3.8; N, 9.0; S, 8.3%. Found: C, 29.7; H, 3.5; N, 8.95; S, 8.0%. IR (KBr, cm^−1^): 3429(m), 3076(w), 2976(m), 2936(m), 2874(w), 1647(s), 1599(vs), 1562(vs), 1518(vs), 1427(vs), 1385(vs), 1348(s), 1314(w), 1290(m), 1258(s), 1206(m), 1149(m), 1086(m), 1016(m), 955(m), 924(vs), 866 (m), 843(m), 762(s), 685(m), 662(w). ^1^H NMR (400 MHz, CD_2_Cl_2_, ppm): 8.64 (m, 4H, py); 8.34 (m, 2H, py); 4.19–4.07 (m, 8H, CH_2_); 3.97–3.85 (m, 8H, CH_2_); 2.54 (s, 6H, CH_3_, OAc); 1.40–1.32 (t, 12H, CH_3_), 1.26–1.22 (m, 12H, CH_3_). UV/Vis (CH_2_Cl_2_, nm): 229 (ε = 11.2 × 10^3^ L mol^−1^ cm^−1^), 288 (ε = 8.9 × 10^3^ L mol^−1^ cm^−1^), 370 (ε = 1.8 × 10^3^ L mol^−1^ cm^−1^).

*[Mn{UO_2_(L^Et2^)(OAc)}_2_]* (**5d**). Yield: 87% (62 mg). Elemental analysis: Calcd. for C_40_H_56_Cl_2_N_10_O_12_S_4_U_2_Mn ([Mn{UO_2_(L^Et2^)(OAc)}_2_] · 2 CH_2_Cl_2_): C, 28.7; H, 3.4; N, 8.4; S, 7.7%. Found: C, 28.7; H, 3.4; N, 8.3; S, 7.6%. IR (KBr, cm^−1^): 3445(w), 3075(w), 2974(m), 2936(m), 2874(w), 1649(s), 1600(vs), 1558(vs), 1518(vs), 1423(vs), 1387(vs), 1348(s), 1314(w), 1292(m), 1258(s), 1206(m), 1149(m), 1082(m), 1016(m), 956(m), 924(vs), 868 (m), 843(m), 760(s), 685(m), 662(w). UV/Vis (CH_2_Cl_2_, nm): 231 (ε = 10.3 × 10^3^ L mol^−1^ cm^−1^), 289 (ε = 8.5 × 10^3^ L mol^−1^cm^−1^), 369 (ε = 1.7 × 10^3^ L mol^−1^ cm^−1^).

*[Zn{UO_2_(L^Et2^)(OAc)}_2_]* (**5e**). Yield: 72% (54 mg). Elemental analysis: Calcd. For C_39_H_54_Cl_2_N_10_O_12_S_4_U_2_Zn ([Zn{UO_2_(L^Et2^)(OAc)}_2_] · (CH_2_Cl_2_): C, 29.4; H, 3.4; N, 8.8; S, 8.0%. Found: C, 29.4; H, 3.5; N, 8.8; S, 8.1%. IR (KBr, cm^−1^): 3445(m), 3080(w), 2976(m), 2936(m), 2874(w), 1647(s), 1597(vs), 1562(vs), 1516(vs), 1431(vs), 1395(vs), 1358(w), 1312(m), 1287(m), 1256(s), 1204(m), 1149(m), 1123(m), 1078(m), 1013(m), 962(w), 916(vs), 847 (m), 758(s), 680(m), 659(w). UV/Vis (CH_2_Cl_2_, nm): 234 (ε = 11.1 × 10^3^ L mol^−1^ cm^−1^), 309 (ε = 9.1 × 10^3^ L mol^−1^ cm^−1^), 369 (ε = 1.6 × 10^3^ L mol^−1^ cm^−1^).

*[Cd{UO_2_(L^Et2^)(OAc)}_2_]* (**5f**). Yield: 70% (54 mg). Elemental analysis: Calcd. for: C_40_H_60_N_10_O_16_S_4_U_2_Cd ([Cd{UO_2_(L^Et2^)(OAc)}_2_] 2 MeOH): C, 29.6; H, 3.7; N, 8.6; S, 7.9%. Found: C, 30.0; H, 3.7; N, 8.8; S, 7.9%. IR (KBr, cm^−1^): 3446(m), 3078(w), 2974(m), 2933(m), 2874(w), 1649(s), 1599(vs), 1566(vs), 1518(vs), 1431(vs), 1396(vs), 1360(w), 1310(m), 1287(m), 1254(s), 1204(m), 1149(m), 1123(m), 1076(m), 1013(m), 962(w), 916(vs), 847 (m), 758(s), 679(m), 654(w). ^1^H NMR (CD_2_Cl_2_, ppm): 8.67 (d, 4H, *J* = 7.6 Hz, py); 8.41 (t, 2H, *J* = 7.6 Hz, py); 4.25–4.15 (m, 8H, CH_2_); 3.98–3.88 (m, 8H, CH_2_); 2.29 (s, 6H, CH_3__OAc); 1.30 (t, 12H, *J* = 7.4 Hz, CH_3_), 1.09 (t, *J* = 7.4 Hz, 12H, CH_3_). ESI(+) MS (*m*/*z*): 1581.2603 (calcd. 1581.2596) [M+Na]^+^, 1559.2914 (calcd. 1559.2776) [M+H]^+^. UV/Vis (CH_2_Cl_2_, nm): 231 (ε = 13.1 × 10^3^ L mol^−1^ cm^−1^), 305 (ε = 9.5 × 10^3^ L mol^−1^ cm^−1^), 369 (ε = 1.5 × 10^3^ L mol^−1^ cm^−1^).

*[Ni{UO_2_(L^morph^)(OAc)}_2_]* (**6a**). Yield: 60% (47 mg). Elemental analysis: Calcd. for C_39_H_46_Cl_2_N_10_O_16_S_4_U_2_Ni ([Ni{UO_2_(L^morph^)(OAc)}_2_] · CH_2_Cl_2_): C, 29.0; H, 3.0; N, 8.0; S, 7.4%. Found: C, 29.0; H, 3.0; N, 8.0; S, 7.4%. IR (KBr, cm^−1^): 3445(s), 2963(w), 2922(w), 2856(w), 1647(s), 1601(s), 1560(s), 1504(vs), 1429(s), 1387(vs), 1341(m), 1290(m), 1269(w), 1232(m), 1113(m), 1032(m), 955(m), 924(vs), 845 (m), 764(m), 687(w), 607(m). UV/Vis (CH_2_Cl_2_, nm): 231 (ε = 12.8 × 10^3^ L mol^−1^ cm^−1^), 298 (ε = 11.7 × 10^3^ L mol^−1^ cm^−1^), 369 (ε = 2.6 × 10^3^ L mol^−1^ cm^−1^), 398 (ε = 1.9 × 10^3^ L mol^−1^cm^−1^).

*[Co{UO_2_(L^morph^)(OAc)}_2_]* (**6b**). Yield: 76% (59 mg). Elemental analysis: Calcd. for C_40_H_50_Cl_2_N_10_O_17_S_4_U_2_Co ([Co{UO_2_(L^morph^)(OAc)}_2_] · CH_2_Cl_2_ · CH_3_OH): C, 29.9; H, 3.2; N, 8.3; S, 7.3%. Found: C, 29.9; H, 3.2; N, 8.3; S, 7.3%. IR (KBr, cm^−1^): 3427(vs), 2966(w), 2922(w), 2858(w), 1647(s), 1600(s), 1562(s), 1504(vs), 1431(s), 1388(vs), 1342(m), 1292(m), 1232(m), 1113(m), 1032(m), 953(m), 924(vs), 844 (m), 764(m), 683(w), 607(m). UV/Vis (CH_2_Cl_2_, nm): 235 (ε = 12.2 × 10^3^ L mol^−1^ cm^−1^), 298 (ε = 12.3 × 10^3^ L mol^−1^ cm^−1^), 369 (ε = 2.5 × 10^3^ L mol^−1^ cm^−1^), 399 (ε = 1.5 × 10^3^ L mol^−1^ cm^−1^).

*[Fe{UO_2_(L^morph^)(OAc)}_2_]* (**6c**). Yield: 53% (42 mg). Elemental analysis: Calcd. for C_38_H_44_N_10_O_16_S_4_U_2_Fe: C, 29.3; H, 2.9; N, 9.0; S, 8.2%. Found: C, 29.3; H, 2.9; N, 9.0; S, 8.2%. IR (KBr, cm^−1^): 3433(vs), 2959(w), 2920(w), 2855(w), 1639(s), 1589(s), 1555(vs), 1506(s), 1431(vs), 1391(vs), 1346(m), 1285(m), 1227(m), 1111(s), 1067(w), 1026(m), 951(m), 916(vs), 841 (m), 758(m), 675(m), 637(w). ^1^H NMR (CD_2_Cl_2_, ppm): 8.72 (m, 4H, py); 8.38 (m, 2H, py); 4.35–3.70 (32H, CH_2_); 2.59 (s, 6H, CH_3_, OAc). UV/Vis (CH_2_Cl_2_, nm): 231 (ε = 12.2 × 10^3^ L mol^−1^ cm^−1^), 284 (ε = 10.8 × 10^3^ L mol^−1^ cm^−1^), 368 (ε = 2.2 × 10^3^ L mol^−1^ cm^−1^).

*[Mn{UO_2_(L^morph^)(OAc)}_2_]* (**6d**). Yield: 80% (62 mg). Elemental analysis: Calcd. for C_40_H_48_N_10_O_18_S_4_U_2_Mn ([Mn{UO_2_(L^morph^)(OAc)}_2_]·2 CH_3_OH): C, 30.9; H, 3.5; N, 8.6; S, 7.9%. Found: C, 30.9; H, 3.5; N, 8.6; S, 7.8%. IR (KBr, cm^−1^): 3429(m), 2965(w), 2922(w), 2857(w), 1647(s), 1598(s), 1564(vs), 1506(s), 1429(s), 1391(vs), 1344(m), 1288(m), 1234(m), 1115(m), 1069(w), 1032(m), 953(m), 922(vs), 843 (m), 764(m), 685(m), 607(w). UV/Vis (CH_2_Cl_2_, nm): 233 (ε = 12.1 × 10^3^ L mol^−1^ cm^−1^), 293 (ε = 11.4 × 10^3^ L mol^−1^cm^−1^), 369 (ε = 2.3 × 10^3^ L mol^−1^ cm^−1^), 399 (ε = 1.4 × 10^3^ L mol^−1^ cm^−1^).

*[Zn{UO_2_(L^morph^)(OAc)}_2_]* (**6e**). Yield: 74% (58 mg). Elemental analysis: Calcd. for C_39_H_46_Cl_2_N_10_O_16_S_4_U_2_Zn ([Zn{UO_2_(L^morph^)(OAc)}_2_]·CH_2_Cl_2_): C, 29.6; H, 3.0; N, 8.4; S, 7.7%. Found: C, 29.7; H, 3.0; N, 8.4; S, 7.7%. IR (KBr, cm^−1^): 3443(s), 2967(w), 2924(w), 2858(w), 1647(s), 1597(s), 1560(vs), 1506(vs), 1429(s), 1394(vs), 1344(m), 1292(m), 1234(m), 1115(m), 1069(w), 1032(m), 953(m), 922(vs), 843 (m), 764(m), 687(m), 607(w). ESI(+) MS (*m*/*z*): 1603.1741 (calcd. 1603.1758) [M+K]^+^; 1587.2003 (calcd. 1587.2019) [M+Na]^+^. UV/Vis (CH_2_Cl_2_, nm): 230 (ε = 12.8 × 10^3^ L mol^−1^ cm^−1^), 301 (ε = 11.5 × 10^3^ L mol^−1^ cm^−1^), 369 (ε = 2.5 × 10^3^ L mol^−1^ cm^−1^).

*[Cd{UO_2_(L^morph^)(OAc)}_2_]* (**6f**). Yield: 72% (58 mg). Elemental analysis: Calcd. for C_40_H_48_Cl_2_N_10_O_16_S_4_U_2_Cd ([Cd{UO_2_(L^morph^)(OAc)}_2_]·2 CH_2_Cl_2_): C, 29.3; H, 2.8; N, 8.7; S, 8.0%. Found: C, 29.3; H, 2.8; N, 8.7; S, 8.0%. IR (KBr, cm^−1^): 3441(s), 2964(w), 2922(w), 2857(w), 1647(s), 1595(s), 1562(vs), 1506(vs), 1429(s), 1394(vs), 1342(m), 1292(m), 1233(m), 1113(m), 1069(w), 1030(m), 951(m), 920(vs), 843 (m), 762(m), 685(m), 606(w). ^1^H NMR (CD_2_Cl_2_, ppm): 8.82 (d, *J* = 7,6 Hz, 4H, py); 8.46 (t, *J* = 7.6 Hz, 2H, py); 4.40–3.50 (m, 32H, CH_2_); 2.32 (s, 6H, CH_3__ OAc). ESI(+) MS (*m*/*z*): 1653.1428 (calcd. 1653.1500) [M+K]^+^; 1637.1689 (calcd. 1637.1761) [M+Na]^+^. UV/Vis (CH_2_Cl_2_, nm): 230 (ε = 12.4 × 10^3^ L mol^−1^ cm^−1^), 300 (ε = 10.1 × 10^3^ L mol^−1^ cm^−1^), 369 (ε = 2.2 × 10^3^ L mol^−1^ cm^−1^).

[(UO_2_)(NiI)_2_(L^Et2^)_2_]. [UI_3_(dioxane)1.5] (76 mg, 0.1 mmol) was suspended in THF (5 mL), and NiI_2_ (78 mg, 0.25 mmol) and H_2_L^Et2^ (80 mg, 0.2 mmol) were added as solids. The mixture was stirred at room temperature until a clear solution was formed (approximately 2 h). Brown crystals were deposited upon slow evaporation of the solvent. Yield: 45% (71 mg). Elemental analysis: Calcd. for C_42_H_62_I_2_N_10_Ni_2_O_8_S_4_U ([(UO_2_)(NiI)_2_(L^Et2^)_2_] × 2 THF); C, 32.0;H, 3.9; N, 8.9; S, 8.1%. Found: C, 31.5; H, 3.8; N, 8.6; S, 8.2%. IR (KBr, cm^−^1): 3450(m), 2966(m), 2933(w), 2870(w), 2813(w), 2745(w), 1591(vs), 1550(vs), 1422(s), 1389(vs), 1358(w), 1307(w), 1271(m), 1252(s), 1148(m), 1120(m), 1070(m), 1014(m), 906 (vs), 873(m), 839(w), 788(w), 757(m), 664(m). ESI(+) MS (*m*/*z*): 1427.3901 (calcd. 1426.9867) [M+H]^+^; 1301.0630 (calcd. 1301.0822) [M–I+H]^+^ 1174.1608 (calcd. 1174.1778) [M–2I+H]^+^.

### 3.4. X-Ray Crystallography

The intensities for the X-ray determinations were collected on STOE IPDS-2T (STOE, Darmstadt, Germany) or Bruker CCD instruments (BRUKER, Billerica, MA, USA) with Mo/Kα radiation. The various temperatures applied are due to the experimental setup of the different diffractometers. Semi-empirical or numerical absorption corrections were carried out by the SADABS or X-RED32 programs [87,88]. Structure solution and refinement were performed with the SHELX programs [89,90] included in the OLEX2 program package (version 1.5) [91]. Hydrogen atoms were calculated for idealized positions and treated with the “riding model” option of SHELXL. The solvent mask option of OLEX2 was applied to treat diffuse electron density due to disordered solvents. Details are given in the Supplementary Materials. The representation of molecular structures was conducted using the program Mercury [92].

### 3.5. Computational Chemistry

Density Functional Theory (DFT) calculations were performed with the high-performance computing system of the ZEDAT [93] using the program package GAUSSIAN 16 [94]. The gas phase geometry optimizations were performed using coordinates derived from the X-ray crystal structures. The calculations for the ligand molecules H_2_L^Et2^ and H_2_L^morph^ were performed without any restrictions on the structures by using the hybrid density functional B3LYP [95,96,97] together with the 6-311G basis set for all atoms as implemented in Gaussian [98,99]. For initial optimization of uranium-containing compounds, the LANL2DZ basis set and the corresponding effective core potential (ECP) was used for uranium [100], while 6-311G was initially kept for the other atoms. The bigger 6-311++G** basis set was used for the other atoms to obtain more reliable geometries [98,99,101,102,103]. Relativistic, dispersion corrected, all-electron calculations on the PBE0-DKH2-GD3BJ/SARC-DKH2(uranium)+aug-cc-pVTZ-DK(others) level as suggested in ref. [104] were attempted but the system size proved prohibitive. For comparable accuracy as outlined in ref. [105], calculations using the PBE0 hybrid functional ([106]) with Grimme dispersion and Becke-Johnson damping ([107]) using the Stuttgart relativistic large core ECP for uranium [108,109], and the def2-TZVPPD basis set for all other atoms [110,111] were attempted. Although some SCF calculations for the *N*,*N*-diethyl derivatives did not converge (see main text), the energetic trends are reasonably close to those obtained at the B3LYP level. The same level of theory (PBE0-GD3BJ/def2-TZVPPD) was applied in single point calculations of the ligand isomers with geometries obtained at the B3LYP/6-311G to obtain more reliable relative energy difference estimated between the different conformers. Further details are given in the Supporting Information. All basis sets were obtained from the EMSL database or the Basis Set Exchange repository [108,109]. Frequency calculations after the optimizations confirmed the convergence through the absence of imaginary frequencies.

## 4. Conclusions

2,6-Dipicolinoylbis(*N,N*-dialkylthioureas) are versatile ligands, which form a variety of uranium complexes. The structures of the dimeric compounds of the composition [{UO_2_(L^R^)(µ-OMe)}_2_]^2−^ are flexible, and different conformations are established depending on the solvents used for crystallization and the counter ions. The dimers readily dissociate in solution. [{UO_2_(L^R^)(µ-OMe)}_2_]^2−^ building blocks are also structural components of bimetallic complexes with gold and lead; thus, the corresponding products display a similar dissociation behavior in solution. More stable are bimetallic products with M^2+^ transition metal ions, in which a different bonding mode of the {L^R^}^2−^ ligands to the uranyl centers is established. Generally, a strong pH dependence was observed during the complex formation, which makes such compounds interesting for metal extraction processes in nuclear waste solutions. Additionally, information is delivered about potential distribution pathways and chemical species conversions in biological systems in which pH changes play a decisive role.

## Figures and Tables

**Table 1:** Selected Bond Lengths (Å) and Angles in the Different Isomers of [{UO_2_(L^Et2^)(µ-OMe)}_2_]^2−^.

	**U1–O10**	**U1–O20**	**U2–O30**	**U2–O40**	**U1–N46**	**U2–N56**	**U1–O5**	**U1–O15**	**U2–O25**
*syn,syn* ^(a)^	1.765(6)	1.770(6)	1.776(6)	1.783(6)	2.579(7)	2.564(7)	2.357(6)	2.376(5)	2.380(6)
*anti,anti* ^(b)^	1.768(7)	1.762(7)	-	-	2.518(6)	-	2.357(7)	2.348(8)	-
*syn,anti* ^(c)^	1.782(3)	1.786(3)	1.788(3)	1.791(3)	2.537(4)	2.531(4)	2.359(3)	2.362(3)	2.357(3)
	**U2–O35**	**U1–O61**	**U1–O62**	**U2–O61**	**U2–O62**	**C2–S1**	**C12–S11**	**C22–S21**	**C32–S31**
*syn,syn* ^(a)^	2.372(6)	2.348(6)	2.365(5)	2.336(6)	2.344(5)	1.686(7)	1.697(7)	1.674(7)	1.686(7)
*anti,anti* ^(b)^	-	2.357(7)	2.377(8)	-	-	1.67(1)	1.68(1)	-	-
*syn,anti* ^(c)^	2.365(3)	2.332(3)	2.350(3)	2.351(3)	2.341(3)	1.687(5)	1.690(5)	1.686(5)	1.695(5)
	**C4–O5**	**C14–O15**	**C24–C25**	**C34–O35**	**U1…U2**	**O5–U1–O15**		**O25–U2–O35**	
*syn,syn* ^(a)^	1.305(5)	1.305(5)	1.302(6)	1.307(5)	3.772(5)	126.7(3)		126.8(3)	
*anti,anti* ^(b)^	1.289(9)	1.280(12)	-	-	3.7825(7)	127.1(2)		-	
*syn,anti* ^(c)^	1.284(5)	1.291(5)	1.288(5)	1.291(5)	3.7343(3)	126.1(1)		127.1(1)	

^(a)^ see Figure 1a, values taken from ref. [41], atom numbers adopted from Figure 2; ^(b)^ see Figure 2a, ^(c)^ see Figure 2b.

**Table 2:** Selected Bond Lengths (Å) in [Pb_2_(UO_2_)_3_(L^Et2^)_3_(µ-OMe)_2_(MeOH)_2_] (**3**).

**U1–O10**	**U1–O20**	**U2–O30**	**U2–O40**	**U3–O50**	**U3–O60**	**U1–N60**	**U1–O25**	**U1–O3**	**U2–O35**
1.778(4)	1.773(4)	1.806(4)	1.780(4)	1.770(4)	1.763(4)	2.527(5)	2.346(3)	2.359(3)	2.355(2)
**U2–N76**	**U2-O3**	**Pb1–S31**	**Pb1–S11**	**Pb1–S1**	**Pb1-O13**	**Pb1–O15**	**Pb1–O5**	**U3–O15**	**U3–N46**
2.541(4)	2.292(3)	2.795(1)	2.720(1)	2.883(1)	2.686(3)	2.735(3)	2.710(3)	2.470(2)	2.654(4)
**U3–N76**	**U3–O5**	**U1…U2**							
2.647(4)	2.468(2)	3.7114(9)							

**Table 3:** Selected Bond Lengths (Å) and Angles in the [{(UO_2_)_2_(L^Et2^)(µ_2_-OAc)(µ_3_-O)}_2_]^2−^ anion (**4**).

**U1–O10**	**U1–O20**	**U2–O30**	**U2–O40**	**U1–O1**	**U2–O1**	**U1–O5**	**U1–N26**	**U1–O15**
1.768(7)	1.774(7)	1.801(6)	1.775(6)	2.240(5)	2.254(5)	2.385(6)	2.558(7)	2.500(5)
**U1–O32**	**U2–S11**	**U2–O15**	**U2–O33′**	**U1…U2**	**U1…U1′**	**U2…U2′**	**U1–O1–U2**	**U1–O31-U2′**
2.374(7)	2.911(4)	2.509(6)	2.370(6)	3.8721(5)	7.089(8)	3.6555(8)	119.0(2)	131.6(2)

**Table 4:** Selected Bond Lengths (Å) in the [M{UO_2_(L^Et2^)(OAc)}_2_] (**5**) and [M{UO_2_(L^morph^)(OAc)}_2_] (**6**) complexes.

	**U1–S1**	**U1–N3**	**U1–N26**	**U1–O15**	**U1–O31**	**U1–O32**	**U2–S41**	**U2–N33**
**5a** (Ni)	2.864(4)	2.47(1)	2.56(1)	2.49(1)	2.52(1)	2.45(1)	-	-
**5b** (Co)	2.855(1)	2.578(4)	2.565(4)	2.493(3)	2.525(3)	2.449(3)	-	-
**5c** (Fe)	2.859(1)	2.468(4)	2.561(4)	2.494(3)	2.535(4)	2.462(3)	-	-
**5d** (Mn)	2.871(1)	2.472(4)	2.549(4)	2.518(3)	2.511(3)	2.475(3)	2.886(1)	2.480(4)
**6b** (Co)	2.872(2)	2.460(4)	2.549(4)	2.487(4)	2.517(4)	2.462(4)	2.854(2)	2.465(5)
	**U2–N66**	**U2–O35**	**M1–S11**	**M1–S51**	**M1–O15**	**M1–O55**	**M1–O32**	**M1–O72**
**5a** (Ni)	-	-	2.350(5)	-	2.08(1)	-	2.07(1)	-
**5b** (Co)	-	-	2.362(2)	-	2.133(3)	-	2.093(4)	-
**5c** (Fe)	-	-	2.410(2)	-	2.155(3)	-	2.119(4)	-
**5d** (Mn)	2.557(4)	2.480(4)	2.540(1)	2.515(1)	2.216(3)	2.228(3)	2.177(3)	2.164(3)
**6b** (Co)	2.562(5)	2.490(4)	2.397(2)	2.441(2)	2.168(4)	2.139(4)	2.0754)	2.120(4)
	**U1…M1**	**U2…M1**	**U1…U2/U1′ ^(a)^**	**C4–O5**	**C14–O15**	**C44–O45**	**C54–O55**	
**5a** (Ni)	3.702(1)	-	6.115(1)	1.24(2)	1.27(2)	-	-	
**5b** (Co)	3.766(1)	-	6.376(1)	1.220(6)	1.286(6)	-	-	
**5c** (Fe)	3.779(1)	-	6.290(1)	1.228(6)	1.300(6)	-	-	
**5d** (Mn)	3.926(1)	3.922(1)	6.645(1)	1.228(6)	1.302(5)	1.223(6)	1.297(5)	
**6b** (Co)	3.817(1)	3.760(1)	6.759(1)	1.223(7)	1.301(6)	1.222(7)	1.257(7)	

^(a)^ Symmetry operation: −x,y,1.5–z.

**Table 5:** Selected Bond Lengths (Å) in [(UO_2_)(NiI)_2_(L^Et2^)_2_] (**7**).

**U1–O10**	**U1–O20**	**U1–O5**	**U1–O15**	**U1–O25**	**U1–O35**	**U1–N46**	**U1–N56**	**Ni1–I1**
1.775(7)	1.767(6)	2.485(6)	2.405(6)	2.476(6)	2.553(6)	2.600(7)	2.588(7)	2.642(3)
**Ni1–S11**	**Ni1–S31**	**Ni1–O15**	**Ni1–O35**	**Ni2–I2**	**Ni2–S1**	**Ni2–S21**	**Ni2–O5**	**Ni2–O25**
2.308(4)	2.282(3)	2.052(6)	2.040(6)	2.702(3)	2.288(3)	2.301(3)	2.055(6)	2.036(6)
**C14–O15**	**C34–O35**	**C4–O5**	**C24–O25**	**Ni1…U1**	**Ni2…U1**	**Ni1…Ni2**		
1.30(1)	1.29(1)	1.30(1)	1.30(1)	3.569(2)	3.708(2)	7.199(1)		

## Data Availability

Data are contained within the article and Supplementary Materials.

## Supplementary Materials

The following supporting information can be downloaded at: https://www.mdpi.com/article/10.3390/molecules29215001/s1: **Table S1:** Crystallographic data and data collection parameters. **Table S2:** Crystallographic data and data collection parameters for inspection only. **Figure S1:** Ellipsoid representation of the structure of (HNEt_3_)_2_*anti,anti*-[**1**], also illustrating the disordered parts of the molecule. The thermal ellipsoids are set at a 30% probability level. Hydrogen atoms are omitted for clarity. **Table S3:** Bond lengths (Å) in (HNEt_3_)_2_*anti,anti*-[**1**]. **Table S4:** Bond angles (°) in (HNEt_3_)_2_*anti,anti*-[**1**]. **Figure S2:** Ellipsoid representation of the structure of (EtPPh_3_)_2_*syn,anti*-[**1**], also illustrating the disordered parts of the molecule. The thermal ellipsoids are set at a 30% probability level. Hydrogen atoms are omitted for clarity. **Table S5:** Bond lengths (Å) (EtPPh_3_)_2_*syn,anti*-[**1**]. **Table S6:** Bond angles (°) (EtPPh_3_)_2_*syn,anti*-[**1**]. **Figure S3:** Ellipsoid representation of the structure of [Pb_2_(UO_2_)_3_(L^Et2^)_3_(µ-OMe)_2_(MeOH)_2_] (**3**), also illustrating the disordered parts of the molecule. The thermal ellipsoids are set at a 30% probability level. Hydrogen atoms are omitted for clarity. **Table S7:** Bond lengths (Å) [Pb_2_(UO_2_)_3_(L^Et2^)_3_(µ-OMe)_2_(MeOH)_2_] (**3**). **Table S8:** Bond angles (°) [Pb_2_(UO_2_)_3_(L^Et2^)_3_(µ-OMe)_2_(MeOH)_2_] (**3**). **Figure S4:** Ellipsoid representation of the structure of [{(UO_2_)_2_(L^Et2^)(µ_2_-OAc)(µ_3_-O)}_2_] (**4**), also illustrating the partial S/O (90/10) exchange. The thermal ellipsoids are set at a 30% probability level. Hydrogen atoms are omitted for clarity. **Table S9:** Bond lengths (Å) of [{(UO_2_)_2_(L^Et2^)(µ_2_-OAc)(µ_3_-O)}_2_] (**4**). **Table S10:** Bond angles (°) in of [{(UO_2_)_2_(L^Et2^)(µ_2_-OAc)(µ_3_-O)}_2_] (**4**). **Figure S5.** Ellipsoid representation of the structure of [Ni{UO_2_(L^Et2^)(OAc)}_2_] (**5a**) x MeOH. The thermal ellipsoids are set at a 30% probability level. Hydrogen atoms are omitted for clarity. **Table S11:** Bond lengths (Å) of [Ni{UO_2_(L^Et2^)(OAc)}_2_] (**5a**). **Table S12:** Bond angles (°) in [Ni{UO_2_(L^Et2^)(OAc)}_2_] (**5a**). **Figure S6:** Ellipsoid representation of the structure of [Co{UO_2_(L^Et2^)(OAc)}_2_] (5b) x CH_2_Cl_2_. The thermal ellipsoids are set at a 30% probability level. Hydrogen atoms are omitted for clarity. **Table S13:** Bond lengths (Å) of [Co{UO_2_(L^Et2^)(OAc)}_2_] (**5b**). **Table S14:** Bond angles (°) in [Co{UO_2_(L^Et2^)(OAc)}_2_] (**5b**). **Figure S7:** Ellipsoid representation of the structure of [Fe{UO_2_(L^Et2^)(OAc)}_2_] (**5c**). The thermal ellipsoids are set at a 30% probability level. Hydrogen atoms are omitted for clarity. **Table S15:** Bond lengths (Å) of [Fe{UO_2_(L^Et2^)(OAc)}_2_] (**5c**). **Table S16.** Bond angles (°) in [Fe{UO_2_(L^Et2^)(OAc)}_2_] (**5c**). **Figure S8:** Ellipsoid representation of the structure of [Mn{UO_2_(L^Et2^)(OAc)}_2_] (**5d**) x CH_2_Cl_2_. The thermal ellipsoids are set at a 30% probability level. Hydrogen atoms are omitted for clarity. **Table S17:** Bond lengths (Å) of [Mn{UO_2_(L^Et2^)(OAc)}_2_] (**5d**). **Table S18:** Bond angles (°) in [Mn{UO_2_(L^Et2^)(OAc)}_2_] (**5d**). **Figure S9:** Ellipsoid representation of the structure of [Co{UO_2_(L^morph^)(OAc)}_2_] (**6b**) x CH_2_Cl_2_. The thermal ellipsoids are set at a 30% probability level. Hydrogen atoms are omitted for clarity. **Table S19:** Bond lengths (Å) of [Co{UO_2_(L^morph^)(OAc)}_2_] (**6b**). **Table S20:** Bond angles (°) in [Co{UO_2_(L^morph^)(OAc)}_2_] (**6b**). **Figure S10:** Ellipsoid representation of the structure of [(UO_2_)(NiI)_2_(L^Et2^)_2_] (**7**) x THF, also illustrating the disordered parts of the molecule. The thermal ellipsoids are set at a 30% probability level. Hydrogen atoms are omitted for clarity. **Table S21:** Bond lengths (Å) of [(UO_2_)(NiI)_2_(L^Et2^)_2_] (**7**). **Table S22:** Bond angles (°) in [(UO_2_)(NiI)_2_(L^Et2^)_2_] (**7**). **Figure S11:** Ellipsoid representation of the structure of (EtPPh_3_)_2_*anti,anti*-[{UO_2_(L^morph^)(µ-OMe)}_2_], (EtPPh_3_)_2_*anti,anti*-[**8**], also illustrating the disordered parts of the molecule. The thermal ellipsoids are set at a 30% probability level. Hydrogen atoms are omitted for clarity. **Table S23:** Bond lengths (Å) of (EtPPh_3_)_2_*anti,anti*-[{UO_2_(Lmorph)(µ-OMe)}_2_], (EtPPh_3_)_2_*anti,anti*-[**8**]. **Table S24:** Bond angles (°) in (EtPPh_3_)_2_*anti,anti*-[{UO_2_(Lmorph)(µ-OMe)}_2_], (EtPPh_3_)_2_*anti,anti*-[**8**]. **Figure S12:** Representation of the structure of (HNEt_3_)_2_*anti,anti*-[{UO_2_(L^morph^)(µ-OMe)}_2_] x MeOH, (HNEt_3_)_2_*anti,anti*-[**8**]. The thermal ellipsoids are set at a 30% probability level. Hydrogen atoms are omitted for clarity. **Table S25:** Bond lengths (Å) of (HNEt_3_)_2_*anti,anti*-[{UO_2_(L^morph^)(µ-OMe)}_2_], (HNEt_3_)_2_*anti,anti*-[**8**]. **Table S26:** Bond angles (°) in (HNEt_3_)_2_*anti,anti*-[{UO_2_(L^morph^)(µ-OMe)}_2_], (HNEt_3_)_2_*anti,anti*-[**8**]. **Figure S13:** Representation of the structure of [{UO_2_(L^Et2^)(µ-OMe)}_2_{Au(PPh_3_)}_2_] (**2**). The thermal ellipsoids are set at a 30% probability level. Hydrogen atoms are omitted for clarity. **Table S27:** Bond lengths (Å) of [{UO_2_(L^Et2^)(µ-OMe)}_2_{Au(PPh_3_)}_2_] (**2**). **Table S28:** Bond angles (°) in [{UO_2_(L^Et2^)(µ-OMe)}_2_{Au(PPh_3_)}_2_] (**2**). **Figure S14:** Ellipsoid representation of the structure of [Ni{UO_2_(L^morph^)(OAc)}_2_] (**6a**), also illustrating the disordered parts of the molecule. The thermal ellipsoids are set at a 30% probability level. Hydrogen atoms are omitted for clarity. **Table S29:** Bond lengths (Å) of [Ni{UO_2_(L^morph^)(OAc)}_2_] (**6a**). **Table S30:** Bond angles (°) in [Ni{UO_2_(L^morph^)(OAc)}_2_] (**6a**). **Figure S15:** Ellipsoid representation of the structure of [Zn{UO_2_(L^morph^)(OAc)}_2_] (**6e**), also illustrating the disordered parts of the molecule. The thermal ellipsoids are set at a 30% probability level. Hydrogen atoms are omitted for clarity. **Table S31:** Bond lengths (Å) of [Zn{UO_2_(L^morph^)(OAc)}_2_] (**6e**). **Table S32:** Bond angles (°) in [Zn{UO_2_(L^morph^)(OAc)}_2_] (**6e**). **Figure S16:** ^1^H NMR spectra of (HNEt_3_)_2_[{UO_2_(L^Et2^)(µ-OMe)}_2_], (HNEt_3_)_2_[**1**], in CDCl_3_ and DMSO. **Figure S17:** ESI(–) mass spectrum of (HNEt_3_)_2_[{UO_2_(L^Et2^)(µ-OMe)}_2_]*,* (HNEt_3_)_2_[**1**]. **Figure S18:** ESI(+) mass spectrum of [Pb_2_(UO_2_)_3_(L^Et2^)_3_(µ-OMe)_2_(MeOH)_2_] (**3**). **Figure S19:** ESI(+) mass spectrum of [Zn{UO_2_(L^Et2^)(OAc)}_2_] (**5e**). **Figure S20:** ^1^H NMR spectrum of [Cd{UO_2_(L^Et2^)(OAc)}_2_] (**6f**) in CDCl_3_. **Figure S21:** ESI(+) mass spectrum of [Cd{UO_2_(L^Et2^)(OAc)}_2_] (**6f**). **Figure S22:** ESI(+) mass spectrum of [(UO_2_)(NiI)_2_(L^Et2^)_2_] (**7**). **Figure S23:** Considered (potential) conformers of H2LPhthal. **Table S33:** DFT calculations of the different conformations of H_2_L^Et2^ and H_2_L^morph^. Level of theory: B3LYP; basis sets: 6-311G. **Figure S24:** Considered (potential) conformers of H2LEt2. Stable conformers are black, while unstable conformers are given in red. Grey conformers have not been considered. **Table S34:** DFT calculations of the different conformations of H_2_L^Phthal^. Level of theory: B3LYP; basis sets: 6-311G. **Figure S25:** Considered (potential) conformers of H2Lmorph. Stable conformers are black, while unstable conformers are given in red. Grey conformers have not been considered. **Table S35:** DFT calculations of the different conformations of H2Lmorph. Optimization level B3LYP/6-311G. Single-point level: PBE0-GD3BJ/def2-TZVPPD. The most stable isomer is bold. **Table S36:** DFT calculations of the different conformations of H2Lmorph. Level: B3LYP/LANL2DZ(uranium)+6-311++G**(others). The most stable isomer is bold. **Table S37:** DFT calculations of the different conformations of H2Lmorph. Optimization level: B3LYP/LANL2DZ(uranium)+6-311++G**(others). Single-point level: PBE0-GD3BJ/StuttgartRLC(uranium)+def2-TZVPPD(others). The most stable isomer is bold.
