# Supplementary material for: Bimetallic Uranium Complexes with 2,6-Dipicolinoylbis(N,N-Dialkylthioureas)
Source: Molecules. 2024 Oct 22;29(21):5001. doi: 10.3390/molecules29215001 (PMC11548025; doi:10.3390/molecules29215001)

checkCIF/PLATON report

Structure factors have been supplied for datablock(s) Et2_antianti_1, Et2_synanti_1, et2_co_5b, et2_fe_5c, et2_mn_5d, et2_ni_5a, et2_ni_i_7, et2_pb_3, et2_tetramer_4, morph_antianti_8, morph_co_6b

THIS REPORT IS FOR GUIDANCE ONLY. IF USED AS PART OF A REVIEW PROCEDURE FOR PUBLICATION, IT SHOULD NOT REPLACE THE EXPERTISE OF AN EXPERIENCED CRYSTALLOGRAPHIC REFEREE.

No syntax errors found. CIF dictionary Interpreting this report

Datablock: Et2_antianti_1

Bond precision: C-C = 0.0212 A Wavelength=0.71073

Cell:	a=33.452(2)	b=9.702(2)	c=22.278(2)
	alpha=90	beta=119.96(2)	gamma=90
Temperature:	346 K		

	Calculated	Reported
Volume	6264.2(19)	6264.2(18)
Space group	C 2/c	C 1 2/c 1
Hall group	-C 2yc	-C 2yc
Moiety formula	C36 H52 N10 O10 S4 U2, 2(C6 H16 N)	C36 H52 N10 O10 S4 U2, 2(C6 H16 N)
Sum formula	C48 H84 N12 O10 S4 U2	C48 H84 N12 O10 S4 U2
Mr	1593.57	1593.57
Dx,g cm-3	1.690	1.690
Z	4	4
Mu (mm-1)	5.357	5.357
F000	3136.0	3136.0
F000'	3062.27	
h,k,lmax	38,11,25	38,11,25
Nref	5210	5164
Tmin,Tmax	0.320,0.807	0.236,0.647
Tmin'	0.296	

Correction method= # Reported T Limits: Tmin=0.236 Tmax=0.647
AbsCorr = INTEGRATION

Data completeness= 0.991 Theta(max)= 24.496

```
R(reflections)= 0.0496( 3765)          wR2(reflections)= 0.1241( 5164)
S = 1.051                  Npar= 320
```

The following ALERTS were generated. Each ALERT has the format
test-name_ALERT_alert-type_alert-level.
Click on the hyperlinks for more details of the test.

Alert level B

```
PLAT230_ALERT_2_B Hirshfeld Test Diff for    S1       --C2       .        8.1 s.u.
```

Author Response: Low crystal quality

```
PLAT342_ALERT_3_B Low Bond Precision on  C-C Bonds ...............    0.02118 Ang.
```

Author Response: Low crystal quality

```
PLAT910_ALERT_3_B Missing # of FCF Reflection(s) Below Theta(Min).         39 Note
                0  2  0,   1  1  0,   2  0  0,   2  2  0,   3  1  0,   4  0  0,
                5  1  0,   6  0  0,  -5  1  1,  -3  1  1,  -2  2  1,  -1  1  1,
                0  2  1,   1  1  1,   3  1  1,   5  1  1,  -6  0  2,  -5  1  2,
               -4  0  2,  -3  1  2,  -2  0  2,  -2  2  2,  -1  1  2,   0  0  2,
```

Author Response: Due to the automated data collection routine of the IPDS. Several ’beamstop reflections’

Alert level C

```
THETM01_ALERT_3_C  The value of sine(theta_max)/wavelength is less than 0.590
            Calculated sin(theta_max)/wavelength =    0.5834
PLAT230_ALERT_2_C Hirshfeld Test Diff for    N16      --C3       .        5.9 s.u.
```

Author Response: Low crystal quality

```
PLAT234_ALERT_4_C Large Hirshfeld Difference N6       --C7       .       0.20 Ang.
PLAT241_ALERT_2_C High   'MainMol' Ueq as Compared to Neighbors of        O51 Check
PLAT242_ALERT_2_C Low    'MainMol' Ueq as Compared to Neighbors of         U1 Check
PLAT242_ALERT_2_C Low    'MainMol' Ueq as Compared to Neighbors of         C2 Check
PLAT260_ALERT_2_C Large Average Ueq of Residue Including       N10      0.120 Check
PLAT360_ALERT_2_C Short  C(sp3)-C(sp3) Bond  C3       - C9       .       1.41 Ang.
PLAT906_ALERT_3_C Large K Value in the Analysis of Variance ......      8.005 Check
PLAT911_ALERT_3_C Missing FCF Refl Between Thmin & STh/L=    0.583          7 Report
                6  2  1,  -1  3  2,  -9  1  3, -28  2  5,  -2  0  6, -23  1  8,
              -18  2 12,
PLAT971_ALERT_2_C Check Calcd Resid. Dens.  0.19Ang From S1             2.20 eA-3
PLAT971_ALERT_2_C Check Calcd Resid. Dens.  0.90Ang From S11            1.67 eA-3
PLAT972_ALERT_2_C Check Calcd Resid. Dens.  0.18Ang From S11           -1.90 eA-3
```

```
PLAT972_ALERT_2_C Check Calcd Resid. Dens.  0.33Ang From S11          -1.88 eA-3
PLAT975_ALERT_2_C Check Calcd Resid. Dens.  0.71Ang From O51     .      0.76 eA-3
```

Alert level G

```
PLAT002_ALERT_2_G Number of Distance or Angle Restraints on AtSite          2 Note
PLAT007_ALERT_5_G Number of Unrefined Donor-H Atoms .............          1 Report
              H10
PLAT128_ALERT_4_G Alternate Setting for Input Space Group     C2/c       I2/a Note
PLAT153_ALERT_1_G The s.u.'s on the Cell Axes   are Equal ..(Note)      0.002 Ang.
PLAT171_ALERT_4_G The CIF-Embedded .res File Contains EADP Records          4 Report
PLAT172_ALERT_4_G The CIF-Embedded .res File Contains DFIX Records          1 Report
PLAT187_ALERT_4_G The CIF-Embedded .res File Contains RIGU Records          1 Report
PLAT301_ALERT_3_G Main Residue  Disorder .............(Resd    1)        13% Note
PLAT410_ALERT_2_G Short Intra H...H Contact  H7B      ..H9AB     .       2.02 Ang.
                                                      x,y,z  =      1_555 Check
PLAT413_ALERT_2_G Short Inter XH3 .. XHn     H18F     ..H91A     .       2.08 Ang.
                                                      x,y,z  =      1_555 Check
PLAT720_ALERT_4_G Number of Unusual/Non-Standard Labels ..........          4 Note
              H9AA    H9AB    H9BA    H9BB
PLAT722_ALERT_1_G Angle   Calc      107.00, Rep      108.30 Dev...       1.30 Degree
              N16     -C17A    -H17B       1_555  1_555  1_555     #  110 Check
PLAT794_ALERT_5_G Tentative Bond Valency for U1        (VI)      .       5.69 Info
PLAT860_ALERT_3_G Number of Least-Squares Restraints ............        311 Note
PLAT909_ALERT_3_G Percentage of I>2sig(I) Data at Theta(Max) Still        53% Note
PLAT913_ALERT_3_G Missing # of Very Strong Reflections in FCF ....          1 Note
               -2  0  6,
PLAT933_ALERT_2_G Number of HKL-OMIT Records in Embedded .res File          6 Note
              -28  2  5, -23  1  8, -18  2 12,  -9  1  3,  -1  3  2,   6  2  1,
PLAT941_ALERT_3_G Average HKL Measurement Multiplicity ..........        4.3 Low
PLAT967_ALERT_5_G Note: Two-Theta Cutoff Value in Embedded .res ..       49.0 Degree
PLAT969_ALERT_5_G The 'Henn et al.' R-Factor-gap value ..........      3.461 Note
              Predicted wR2: Based on SigI**2  3.59 or SHELX Weight 11.81
PLAT978_ALERT_2_G Number C-C Bonds with Positive Residual Density.          0 Info
```

---

0 **ALERT level A** = Most likely a serious problem - resolve or explain
3 **ALERT level B** = A potentially serious problem, consider carefully
15 **ALERT level C** = Check. Ensure it is not caused by an omission or oversight
21 **ALERT level G** = General information/check it is not something unexpected

2 ALERT type 1 CIF construction/syntax error, inconsistent or missing data
17 ALERT type 2 Indicator that the structure model may be wrong or deficient
10 ALERT type 3 Indicator that the structure quality may be low
6 ALERT type 4 Improvement, methodology, query or suggestion
4 ALERT type 5 Informative message, check

Datablock: Et2_synanti_1

Bond precision: C-C = 0.0069 A   Wavelength=0.71073

Cell:	a=14.277(1)	b=25.632(1)	c=22.738(1)
	alpha=90	beta=97.3900	gamma=90
Temperature:	100 K		

	Calculated	Reported
Volume	8251.8(8)	8251.8(8)
Space group	P 21/n	P 1 21/n 1
Hall group	-P 2yn	-P 2yn
Moiety formula	C36 H52 N10 O10 S4 U2, 2(C20 H20 P)	C36 H52 N10 O10 S4 U2, 2(C20 H20 P)
Sum formula	C76 H92 N10 O10 P2 S4 U2	C76 H92 N10 O10 P2 S4 U2
Mr	1971.84	1971.83
Dx,g cm-3	1.587	1.587
Z	4	4
Mu (mm-1)	4.120	4.120
F000	3904.0	3904.0
F000'	3831.17	
h,k,lmax	17,32,28	17,32,28
Nref	17005	16919
Tmin,Tmax	0.586,0.719	0.507,0.745
Tmin'	0.175	

Correction method= # Reported T Limits: Tmin=0.507 Tmax=0.745
AbsCorr = MULTI SCAN

Data completeness= 0.995 Theta(max)= 26.446

R(reflections)= 0.0314(14377) wR2(reflections)= 0.0739(16919)

S = 1.087 Npar= 919

The following ALERTS were generated. Each ALERT has the format
test-name_ALERT_alert-type_alert-level.
Click on the hyperlinks for more details of the test.

Alert level B

PLAT910_ALERT_3_B Missing # of FCF Reflection(s) Below Theta(Min). 11 Note
1 1 0, 0 2 0, 1 2 0, -1 0 1, 1 0 1, -1 1 1,
0 1 1, 1 1 1, 0 2 1, 0 0 2, 0 1 2,

Author Response: This is due to the automated data collection routine of the IPDS, 'beamstop reflections'

Alert level C

```
PLAT220_ALERT_2_C NonSolvent   Resd 1  C   Ueq(max)/Ueq(min) Range        3.1 Ratio
PLAT369_ALERT_2_C Long   C(sp2)-C(sp2) Bond  C4       - C41      .       1.53 Ang.
PLAT413_ALERT_2_C Short Inter XH3 .. XHn     H40B     ..H40B     .       2.11 Ang.
                                               2-x,1-y,1-z  =      3_766 Check
PLAT767_ALERT_4_C INS Embedded LIST 6 Instruction Should be LIST 4     Please Check
PLAT911_ALERT_3_C Missing FCF Refl Between Thmin & STh/L=    0.600          3 Report
                4 13  4,   1  0  5,   2 15  7,
PLAT971_ALERT_2_C Check Calcd Resid. Dens.  0.82Ang From U1            1.65 eA-3
PLAT971_ALERT_2_C Check Calcd Resid. Dens.  2.88Ang From C37           1.59 eA-3
PLAT975_ALERT_2_C Check Calcd Resid. Dens.  0.66Ang From O25     .     0.62 eA-3
```

Alert level G

```
PLAT083_ALERT_2_G SHELXL Second Parameter in WGHT  Unusually Large      50.54 Why ?
PLAT145_ALERT_4_G s.u. on beta     Small or Missing .............     0.0000 Degree
PLAT153_ALERT_1_G The s.u.'s on the Cell Axes   are Equal ..(Note)      0.001 Ang.
PLAT171_ALERT_4_G The CIF-Embedded .res File Contains EADP Records          1 Report
PLAT232_ALERT_2_G Hirshfeld Test Diff (M-X)  U1       --N46      .        5.3 s.u.
PLAT480_ALERT_4_G Long H...A H-Bond Reported H71B     ..O2       .       2.65 Ang.
PLAT480_ALERT_4_G Long H...A H-Bond Reported H91      ..O15      .       2.62 Ang.
PLAT480_ALERT_4_G Long H...A H-Bond Reported H121     ..S31      .       2.99 Ang.
PLAT480_ALERT_4_G Long H...A H-Bond Reported H81      ..S11      .       3.01 Ang.
PLAT794_ALERT_5_G Tentative Bond Valency for U1        (VI)      .       5.60 Info
PLAT794_ALERT_5_G Tentative Bond Valency for U2        (VI)      .       5.55 Info
PLAT912_ALERT_4_G Missing # of FCF Reflections Above STh/L=  0.600         72 Note
PLAT913_ALERT_3_G Missing # of Very Strong Reflections in FCF ....          1 Note
                1  0  5,
PLAT969_ALERT_5_G The 'Henn et al.' R-Factor-gap value ..........      3.372 Note
              Predicted wR2: Based on SigI**2  2.19 or SHELX Weight  6.80
PLAT978_ALERT_2_G Number C-C Bonds with Positive Residual Density.          0 Info
```

0 **ALERT level A** = Most likely a serious problem - resolve or explain
1 **ALERT level B** = A potentially serious problem, consider carefully
8 **ALERT level C** = Check. Ensure it is not caused by an omission or oversight
15 **ALERT level G** = General information/check it is not something unexpected

1 ALERT type 1 CIF construction/syntax error, inconsistent or missing data
9 ALERT type 2 Indicator that the structure model may be wrong or deficient
3 ALERT type 3 Indicator that the structure quality may be low
8 ALERT type 4 Improvement, methodology, query or suggestion
3 ALERT type 5 Informative message, check

Datablock: et2_pb_3

Bond precision: C-C = 0.0057 A   Wavelength=0.71073

Cell:	a=10.686(2)	b=29.664(6)	c=17.074(3)
	alpha=90	beta=100.81(3)	gamma=90
Temperature:	100 K		

	Calculated	Reported
Volume	5316.2(18)	5316.2(18)
Space group	P 21/m	P 1 21/m 1
Hall group	-P 2yb	-P 2yb
Moiety formula	C72 H106 N20 O18 Pb2 S8 U3 [+ solvent]	C72 H106 N20 O18 Pb2 S8 U3, 6[CH4O]
Sum formula	C72 H106 N20 O18 Pb2 S8 U3 [+ solvent]	C78 H130 N20 O24 Pb2 S8 U3
Mr	2924.74	3116.96
Dx,g cm-3	1.827	1.947
Z	2	2
Mu (mm-1)	7.931	7.942
F000	2780.0	2996.0
F000'	2712.58	
h,k,lmax	13,38,21	13,38,21
Nref	12076	11816
Tmin,Tmax	0.751,0.853	0.637,0.746
Tmin'	0.574	

Correction method= # Reported T Limits: Tmin=0.637 Tmax=0.746
AbsCorr = MULTI-SCAN

Data completeness= 0.978 Theta(max)= 27.202

R(reflections)= 0.0234(10628) wR2(reflections)= 0.0508(11816)

S = 1.079 Npar= 592

The following ALERTS were generated. Each ALERT has the format
test-name_ALERT_alert-type_alert-level.
Click on the hyperlinks for more details of the test.

Alert level C

```
PLAT220_ALERT_2_C NonSolvent   Resd 1  C   Ueq(max)/Ueq(min) Range        5.3 Ratio
PLAT222_ALERT_3_C NonSolvent Resd 1  H   Uiso(max)/Uiso(min) Range        4.6 Ratio
PLAT767_ALERT_4_C INS Embedded LIST 6 Instruction Should be LIST 4     Please Check
PLAT910_ALERT_3_C Missing # of FCF Reflection(s) Below Theta(Min).           8 Note
                1  0  0,   1  1  0,   0  2  0,  -1  0  1,   0  0  1,  -1  1  1,
                0  1  1,   0  2  1,
PLAT911_ALERT_3_C Missing FCF Refl Between Thmin & STh/L=    0.600          84 Report
               10  0  0,  11  0  0,  12  0  0,  10  1  0,  11  1  0,  12  1  0,
               10  2  0,  11  2  0,  12  2  0,  10  3  0,  11  3  0,  12  3  0,
```

```
              12  4  0, -12  0  1, -11  0  1,   8  0  1,  10  0  1, -12  1  1,
             -11  1  1, -12  2  1, -12  3  1, -11  3  1,   0  3  1, -12  4  1,
             -11  4  1,  12  4  1, -12  5  1, -11  5  1, -12  6  1, -11  6  1,
             -10  6  1, -12  7  1, -11  7  1, -12  8  1, -11  8  1, -12  9  1,
              -1  0  2, -12  4  2, -12  5  2,  -4  5  2, -12  6  2, -12  7  2,
             -12  8  2, -11  8  2, -12  9  2, -12 10  2, -12 11  2,   3  1  3,
              -3  8  3,  -1  2  4,  -2  3  5,  -2  7  5, -12 12  5, -11  0  6,
               1 28  6, -10  0  7,  -9  0  8,  -8  0  9,   0 27  9,  -7  0 10,
               4 22 10,   2 23 10,   4 20 11,   3 21 11,   6 16 12,   5 17 12,
               4 14 13,   7  3 14,   6  6 14,   5  8 14,   5  9 14,   5 10 14,
               4 11 14,   5  1 15,   3  7 15,   3  8 15,   3  9 15,  -7  0 16,
              -7  1 16,  -6  0 17,  -5  0 17,  -6  3 17,   1  9 17,   1 10 17,
PLAT972_ALERT_2_C Check Calcd Resid. Dens.  0.48Ang From U1            -1.77 eA-3
PLAT972_ALERT_2_C Check Calcd Resid. Dens.  0.40Ang From U1            -1.70 eA-3
PLAT972_ALERT_2_C Check Calcd Resid. Dens.  0.53Ang From U1            -1.67 eA-3
PLAT975_ALERT_2_C Check Calcd Resid. Dens.  0.77Ang From O22     .       0.60 eA-3
```

Alert level G

```
FORMU01_ALERT_2_G  There is a discrepancy between the atom counts in the
            _chemical_formula_sum and the formula from the _atom_site* data.
            Atom count from _chemical_formula_sum:C78 H130 N20 O24 Pb2 S8 U3
            Atom count from the _atom_site data:  C72 H106 N20 O18 Pb2 S8 U3
CELLZ01_ALERT_1_G Difference between formula and atom_site contents detected.
CELLZ01_ALERT_1_G ALERT: Large difference may be due to a
            symmetry error - see SYMMG tests
           From the CIF: _cell_formula_units_Z    2
           From the CIF: _chemical_formula_sum  C78 H130 N20 O24 Pb2 S8 U3
           TEST: Compare cell contents of formula and atom_site data

           atom    Z*formula  cif sites diff
           C        156.00    144.00   12.00
           H        260.00    212.00   48.00
           N         40.00     40.00    0.00
           O         48.00     36.00   12.00
           Pb         4.00      4.00    0.00
           S         16.00     16.00    0.00
           U          6.00      6.00    0.00
PLAT007_ALERT_5_G Number of Unrefined Donor-H Atoms .............          1 Report
              H13
PLAT041_ALERT_1_G Calc. and Reported SumFormula    Strings  Differ     Please Check
              Calc: C72 H106 N20 O18 Pb2 S8 U3
              Rep.: C78 H130 N20 O24 Pb2 S8 U3
PLAT042_ALERT_1_G Calc. and Reported MoietyFormula Strings  Differ     Please Check
              Calc: C72 H106 N20 O18 Pb2 S8 U3
              Rep.: C72 H106 N20 O18 Pb2 S8 U3, 6[CH4O]
PLAT083_ALERT_2_G SHELXL Second Parameter in WGHT  Unusually Large      25.62 Why ?
PLAT605_ALERT_4_G Largest Solvent Accessible VOID in the Structure        317 A**3
PLAT794_ALERT_5_G Tentative Bond Valency for U1        (VI)      .       5.65 Info
PLAT794_ALERT_5_G Tentative Bond Valency for U2        (VI)      .       5.67 Info
PLAT794_ALERT_5_G Tentative Bond Valency for U3        (VI)      .       5.25 Info
PLAT794_ALERT_5_G Tentative Bond Valency for Pb1       (II)      .       2.26 Info
PLAT868_ALERT_4_G ALERTS Due to the Use of _smtbx_masks Suppressed          ! Info
PLAT912_ALERT_4_G Missing # of FCF Reflections Above STh/L=  0.600        168 Note
PLAT913_ALERT_3_G Missing # of Very Strong Reflections in FCF ....          2 Note
               1  1  0,  -3  8  3,
PLAT933_ALERT_2_G Number of HKL-OMIT Records in Embedded .res File         20 Note
```

```
              -13  0  4, -13  1  3, -13  2  4, -13  3  4, -13  3  5, -13  6  4,
              -13  7  7, -12 10 12, -12 16  6, -12 17  5, -11  0  6, -11 10 14,
               -7  0 16,  -1  0  2,  -1  2  4,   0  3  1,   0  8 21,   1  1 21,
                6 16 12,   8  1 14,
PLAT941_ALERT_3_G Average HKL Measurement Multiplicity ..........        3.9 Low
PLAT969_ALERT_5_G The 'Henn et al.' R-Factor-gap value ..........      2.528 Note
              Predicted wR2: Based on SigI**2  2.01 or SHELX Weight  4.71
PLAT978_ALERT_2_G Number C-C Bonds with Positive Residual Density.          0 Info
```

```
   0 ALERT level A = Most likely a serious problem - resolve or explain
   0 ALERT level B = A potentially serious problem, consider carefully
   9 ALERT level C = Check. Ensure it is not caused by an omission or oversight
  19 ALERT level G = General information/check it is not something unexpected

   4 ALERT type 1 CIF construction/syntax error, inconsistent or missing data
   9 ALERT type 2 Indicator that the structure model may be wrong or deficient
   5 ALERT type 3 Indicator that the structure quality may be low
   4 ALERT type 4 Improvement, methodology, query or suggestion
   6 ALERT type 5 Informative message, check
```

Datablock: et2_tetramer_4

Bond precision: C-C = 0.0200 A   Wavelength=0.71073

Cell:	a=11.651(1)	b=11.736(1)	c=13.889(1)
	alpha=112.57(1)	beta=100.57(1)	gamma=92.68(1)
Temperature:	173 K		

	Calculated	Reported
Volume	1709.5(3)	1709.5(3)
Space group	P -1	P -1
Hall group	-P 1	-P 1
Moiety formula	C38 H52 N10 O18.24 S3.76 U4, 2(C5.50 H15 N)	C38 H52 N10 O18.245 S3.755 U4, 2(C5.5 H15 N)
Sum formula	C49 H82 N12 O18.24 S3.76 U4	C49 H82 N12 O18.25 S3.75 U4
Mr	2203.71	2203.61
Dx,g cm-3	2.141	2.140
Z	1	1
Mu (mm-1)	9.631	9.631
F000	1034.0	1034.0
F000'	996.77	
h,k,lmax	14,14,17	14,14,17
Nref	6722	6659
Tmin,Tmax	0.243,0.463	0.030,0.137
Tmin'	0.121	

```
Correction method= # Reported T Limits: Tmin=0.030 Tmax=0.137
AbsCorr = INTEGRATION

Data completeness= 0.991          Theta(max)= 26.000

                                                wR2(reflections)=
R(reflections)= 0.0445( 5564)                   0.1132( 6659)

S = 1.045                  Npar= 401
```

The following ALERTS were generated. Each ALERT has the format
test-name_ALERT_alert-type_alert-level.
Click on the hyperlinks for more details of the test.

Alert level B

```
PLAT910_ALERT_3_B Missing # of FCF Reflection(s) Below Theta(Min).        14 Note
                1  0  0,  -1  1  0,   0  1  0,   1  1  0,  -1 -1  1,   0 -1  1,
                1 -1  1,  -1  0  1,   0  0  1,   1  0  1,  -1  1  1,   0  1  1,
                0 -1  2,   0  0  2,
```

Author Response: Due to the autoated data collection routine of the IPDS

```
PLAT972_ALERT_2_B Check Calcd Resid. Dens.  1.10Ang From U1           -2.51 eA-3
```

Author Response: Checked. Some electron density close to the heavy uranium atom

---

Alert level C

```
PLAT041_ALERT_1_C Calc. and Reported SumFormula    Strings  Differ     Please Check
              Calc: C49 H82 N12 O18.24 S3.76 U4
              Rep.: C49 H82 N12 O18.25 S3.75 U4
PLAT042_ALERT_1_C Calc. and Reported MoietyFormula Strings  Differ     Please Check
              Calc: C38 H52 N10 O18.24 S3.76 U4, 2(C5.50 H15 N)
              Rep.: C38 H52 N10 O18.245 S3.755 U4, 2(C5.5 H15 N)
PLAT077_ALERT_4_C Unitcell Contains Non-integer Number of Atoms ..     Please Check
PLAT220_ALERT_2_C NonSolvent   Resd 1   C    Ueq(max)/Ueq(min) Range       4.7 Ratio
PLAT222_ALERT_3_C NonSolvent Resd 1   H   Uiso(max)/Uiso(min) Range       4.3 Ratio
PLAT223_ALERT_4_C Solv./Anion   Resd 2   H    Ueq(max)/Ueq(min) Range       4.9 Ratio
PLAT234_ALERT_4_C Large Hirshfeld Difference C33      --C34      .      0.18 Ang.
PLAT234_ALERT_4_C Large Hirshfeld Difference C42      --C43      .      0.16 Ang.
PLAT241_ALERT_2_C High   'MainMol' Ueq as Compared to Neighbors of       O32 Check
PLAT242_ALERT_2_C Low    'MainMol' Ueq as Compared to Neighbors of       C33 Check
PLAT250_ALERT_2_C Large U3/U1 Ratio for <U(i,j)> Tensor(Resd    2)       2.6 Note
PLAT342_ALERT_3_C Low Bond Precision on  C-C Bonds ..............       0.02 Ang.
PLAT360_ALERT_2_C Short  C(sp3)-C(sp3) Bond  C19      - C20      .      1.34 Ang.
PLAT413_ALERT_2_C Short Inter XH3 .. XHn     H20A     ..H20B     .      2.13 Ang.
                                           2-x,3-y,-z  =      2_785 Check
PLAT906_ALERT_3_C Large K Value in the Analysis of Variance ......      5.116 Check
PLAT911_ALERT_3_C Missing FCF Refl Between Thmin & STh/L=    0.600         40 Report
               -2  2  0,  -4  3  0,  -3  3  0,  -6  4  0,  -5  4  0,  -4  4  0,
```

```
                  6 -6  1,   7 -6  1,   8 -6  1,   5 -5  1,   3 -1  1,   2  0  1,
                 -7 11  1,  -6 12  1,   7 -8  2,   8 -8  2,   8 -7  2,   9 -7  2,
                 10 -7  2,   4 -1  2,   2  0  2,   3  0  2,  -7 11  2,   8 -9  3,
                  9 -9  3,  10 -9  3,   9 -8  3,  10 -8  3,  11 -8  3,   3  0  3,
                  4  0  3,   8-11  4,   9-10  4,  10-10  4,  10 -9  4,  -6 10  4,
                 -2 11  4,   9-11  5, -13  3  6, -12  3  8,
PLAT971_ALERT_2_C Check Calcd Resid. Dens.  1.01Ang From U1           2.22 eA-3
PLAT971_ALERT_2_C Check Calcd Resid. Dens.  1.05Ang From U2           2.08 eA-3
PLAT971_ALERT_2_C Check Calcd Resid. Dens.  1.05Ang From U2           1.71 eA-3
PLAT971_ALERT_2_C Check Calcd Resid. Dens.  1.22Ang From U1           1.69 eA-3
PLAT972_ALERT_2_C Check Calcd Resid. Dens.  1.08Ang From U2          -2.45 eA-3
```

Author Response: Checked. Some electron density close to the heavy uranium atom

```
PLAT972_ALERT_2_C Check Calcd Resid. Dens.  0.94Ang From U2          -2.39 eA-3
```

Author Response: Checked. Some electron density close to the heavy uranium atom

```
PLAT972_ALERT_2_C Check Calcd Resid. Dens.  0.89Ang From U1          -2.22 eA-3
```

Author Response: Checked. Some electron density close to the heavy uranium atom

```
PLAT972_ALERT_2_C Check Calcd Resid. Dens.  0.73Ang From U1          -2.19 eA-3
```

Author Response: Checked. Some electron density close to the heavy uranium atom

```
PLAT972_ALERT_2_C Check Calcd Resid. Dens.  1.08Ang From U1          -2.19 eA-3
```

Author Response: Checked. Some electron density close to the heavy uranium atom

```
PLAT972_ALERT_2_C Check Calcd Resid. Dens.  1.03Ang From U1          -2.18 eA-3
```

Author Response: Checked. Some electron density close to the heavy uranium atom

```
PLAT972_ALERT_2_C Check Calcd Resid. Dens.  0.98Ang From U2          -2.12 eA-3
```

Author Response: Checked. Some electron density close to the heavy uranium atom

```
PLAT972_ALERT_2_C Check Calcd Resid. Dens.  0.81Ang From U2          -2.09 eA-3
```

Author Response: Checked. Some electron density close to the heavy uranium atom

```
PLAT972_ALERT_2_C Check Calcd Resid. Dens.  0.79Ang From U2          -2.01 eA-3
```

Author Response: Checked. Some electron density close to the heavy uranium atom

```
PLAT972_ALERT_2_C Check Calcd Resid. Dens.  0.78Ang From U1            -1.88 eA-3
```

Author Response: Checked. Some electron density close to the heavy uranium atom

```
PLAT972_ALERT_2_C Check Calcd Resid. Dens.  1.11Ang From U2            -1.80 eA-3
```

Author Response: Checked. Some electron density close to the heavy uranium atom

```
PLAT972_ALERT_2_C Check Calcd Resid. Dens.  0.81Ang From U1            -1.60 eA-3
```

Author Response: Checked. Some electron density close to the heavy uranium atom

```
PLAT976_ALERT_2_C Check Calcd Resid. Dens.  1.03Ang From O12     .      -1.38 eA-3
```

Alert level G

```
PLAT007_ALERT_5_G Number of Unrefined Donor-H Atoms .............          1 Report
              H41
PLAT154_ALERT_1_G The s.u.'s on the Cell Angles are Equal ..(Note)       0.01 Degree
PLAT171_ALERT_4_G The CIF-Embedded .res File Contains EADP Records          1 Report
PLAT187_ALERT_4_G The CIF-Embedded .res File Contains RIGU Records          4 Report
PLAT299_ALERT_4_G Atom Site Occupancy Constrained at ............         0.5 Check
              C42     H42A    H42B
PLAT301_ALERT_3_G Main Residue  Disorder .............(Resd    1)          3% Note
PLAT302_ALERT_4_G Anion/Solvent/Minor-Residue Disorder (Resd    2)          8% Note
PLAT304_ALERT_4_G Non-Integer Number of Atoms in ..... (Resd    2)      21.50 Check
PLAT380_ALERT_4_G Incorrectly? Oriented X(sp2)-Methyl Moiety .....        C34 Check
PLAT412_ALERT_2_G Short Intra XH3 .. XHn     H42B     ..H47C     .       2.12 Ang.
                                                   x,y,z  =      1_555 Check
PLAT432_ALERT_2_G Short Inter X...Y Contact  C20      ..C20      .       2.74 Ang.
                                              2-x,3-y,-z  =      2_785 Check
PLAT779_ALERT_4_G Suspect or Irrelevant (Bond) Angle(s) in CIF ...      39.00 Deg.
              O11  -S11  -U2      1_555   1_555   1_555 ......     #   74 Check
PLAT779_ALERT_4_G Suspect or Irrelevant (Bond) Angle(s) in CIF ...      25.00 Deg.
              O11  -C12  -S11     1_555   1_555   1_555 ......     #  124 Check
PLAT794_ALERT_5_G Tentative Bond Valency for U1        (VI)      .       5.61 Info
PLAT860_ALERT_3_G Number of Least-Squares Restraints ............          44 Note
PLAT912_ALERT_4_G Missing # of FCF Reflections Above STh/L=  0.600          9 Note
PLAT913_ALERT_3_G Missing # of Very Strong Reflections in FCF ....          1 Note
                1 -1  1,
PLAT941_ALERT_3_G Average HKL Measurement Multiplicity ..........         2.2 Low
PLAT969_ALERT_5_G The 'Henn et al.' R-Factor-gap value ..........       3.224 Note
              Predicted wR2: Based on SigI**2  3.51 or SHELX Weight 10.82
PLAT978_ALERT_2_G Number C-C Bonds with Positive Residual Density.          0 Info
```

```
   0 ALERT level A = Most likely a serious problem - resolve or explain
   2 ALERT level B = A potentially serious problem, consider carefully
  33 ALERT level C = Check. Ensure it is not caused by an omission or oversight
  20 ALERT level G = General information/check it is not something unexpected

   3 ALERT type 1 CIF construction/syntax error, inconsistent or missing data
```

```
27 ALERT type 2 Indicator that the structure model may be wrong or deficient
 9 ALERT type 3 Indicator that the structure quality may be low
13 ALERT type 4 Improvement, methodology, query or suggestion
 3 ALERT type 5 Informative message, check
```

Datablock: et2_ni_5a

Bond precision: C-C = 0.0255 A   Wavelength=0.71073

Cell:	a=18.684(2)	b=10.5544(11)	c=27.845(3)
	alpha=90	beta=92.732(4)	gamma=90
Temperature:	100 K		

	Calculated	Reported
Volume	5484.8(10)	5484.7(10)
Space group	C 2/c	C 1 2/c 1
Hall group	-C 2yc	-C 2yc
Moiety formula	C38 H52 N10 Ni O12 S4 U2, 2(C H4 O)	C38 H52 N10 Ni O12 S4 U2, 2(C H4 O)
Sum formula	C40 H60 N10 Ni O14 S4 U2	C40 H60 N10 Ni O14 S4 U2
Mr	1567.97	1567.99
Dx,g cm-3	1.899	1.899
Z	4	4
Mu (mm-1)	6.450	6.450
F000	3032.0	3032.0
F000'	2959.82	
h,k,lmax	22,12,32	22,12,32
Nref	4702	4678
Tmin,Tmax	0.347,0.525	0.337,0.745
Tmin'	0.321	

Correction method= # Reported T Limits: Tmin=0.337 Tmax=0.745
AbsCorr = EMPIRICAL

Data completeness= 0.995   Theta(max)= 24.750

R(reflections)= 0.0893(4067)   wR2(reflections)= 0.2076(4678)

S = 1.183   Npar= 179

```
The following ALERTS were generated. Each ALERT has the format
       test-name_ALERT_alert-type_alert-level.
Click on the hyperlinks for more details of the test.
```

Alert level A

```
PLAT971_ALERT_2_A Check Calcd Resid. Dens.  1.03Ang From U1            4.36 eA-3
```

Author Response: Checked. Electron density could not be removed by appropriate absorption correction

```
PLAT971_ALERT_2_A Check Calcd Resid. Dens.  0.97Ang From O10           3.68 eA-3
```

Author Response: Checked. Electron density could not be removed by appropriate absorption correction

```
PLAT972_ALERT_2_A Check Calcd Resid. Dens.  1.86Ang From C20          -5.89 eA-3
```

Author Response: Checked. Electron density could not be removed by appropriate absorption correction

```
PLAT972_ALERT_2_A Check Calcd Resid. Dens.  1.11Ang From U1           -4.52 eA-3
```

Author Response: Checked. Electron density could not be removed by appropriate absorption correction

```
PLAT972_ALERT_2_A Check Calcd Resid. Dens.  1.03Ang From U1           -3.87 eA-3
```

Author Response: Checked. Electron density could not be removed by appropriate absorption correction

```
PLAT972_ALERT_2_A Check Calcd Resid. Dens.  1.27Ang From Ni1          -3.85 eA-3
```

Author Response: Checked. Electron density could not be removed by appropriate absorption correction

---

Alert level B

```
PLAT342_ALERT_3_B Low Bond Precision on  C-C Bonds ...............    0.02545 Ang.
```

Author Response: Low crystal quality

```
PLAT971_ALERT_2_B Check Calcd Resid. Dens.  1.12Ang From U1            3.40 eA-3
```

Author Response: Checked. Electron density could not be removed by appropriate absorption correction

```
PLAT971_ALERT_2_B Check Calcd Resid. Dens.  1.10Ang From U1            3.21 eA-3
```

Author Response: Checked. Electron density could not be removed by appropriate absorption correction

```
PLAT971_ALERT_2_B Check Calcd Resid. Dens.  2.44Ang From C8A           2.98 eA-3
```

Author Response: Checked. Electron density could not be removed by appropriate absorption correction

```
PLAT976_ALERT_2_B Check Calcd Resid. Dens.  0.96Ang From O20     .      -1.74 eA-3
```

Author Response: Checked. Electron density could not be removed by appropriate absorption correction

---

Alert level C

```
RINTA01_ALERT_3_C  The value of Rint is greater than 0.12
            Rint given   0.123
THETM01_ALERT_3_C  The value of sine(theta_max)/wavelength is less than 0.590
            Calculated sin(theta_max)/wavelength =    0.5891
PLAT020_ALERT_3_C The Value of Rint is Greater Than 0.12 .........      0.123 Report
PLAT906_ALERT_3_C Large K Value in the Analysis of Variance ......      8.021 Check
PLAT906_ALERT_3_C Large K Value in the Analysis of Variance ......      2.469 Check
PLAT911_ALERT_3_C Missing FCF Refl Between Thmin & STh/L=    0.589         22 Report
                0   4   0,  12   6   0,  14   6   0, -14   4   1,   0   4   1,   5 11  1,
              -14   6   2,  12   8   3,  14   4   3,  16   8   3,  12   2   4,  14   2   4,
               16   2   4, -14   8   5,  18   4   5, -20   2   6,  16   2   6,   2   0   8,
               18   6   8,  16   2 10, -18   2 14,  -7   1 27,
PLAT971_ALERT_2_C Check Calcd Resid. Dens.  1.32Ang From C20           2.42 eA-3
```

Author Response: Checked. Electron density could not be removed by appropriate absorption correction

```
PLAT971_ALERT_2_C Check Calcd Resid. Dens.  2.05Ang From C20           2.37 eA-3
```

Author Response: Checked. Electron density could not be removed by appropriate absorption correction

PLAT971_ALERT_2_C Check Calcd Resid. Dens. 1.98Ang From C17   2.33 eA-3

Author Response: Checked. Electron density could not be removed by appropriate absorption correction

PLAT971_ALERT_2_C Check Calcd Resid. Dens. 1.18Ang From U1   2.08 eA-3

Author Response: Checked. Electron density could not be removed by appropriate absorption correction

PLAT971_ALERT_2_C Check Calcd Resid. Dens. 0.80Ang From O20   1.95 eA-3

Author Response: Checked. Electron density could not be removed by appropriate absorption correction

PLAT971_ALERT_2_C Check Calcd Resid. Dens. 1.41Ang From U1   1.82 eA-3

Author Response: Checked. Electron density could not be removed by appropriate absorption correction

PLAT971_ALERT_2_C Check Calcd Resid. Dens. 0.94Ang From S1   1.79 eA-3

Author Response: Checked. Electron density could not be removed by appropriate absorption correction

PLAT971_ALERT_2_C Check Calcd Resid. Dens. 1.40Ang From Ni1   1.79 eA-3

Author Response: Checked. Electron density could not be removed by appropriate absorption correction

PLAT971_ALERT_2_C Check Calcd Resid. Dens. 0.99Ang From S11   1.79 eA-3

Author Response: Checked. Electron density could not be removed by appropriate absorption correction

PLAT971_ALERT_2_C Check Calcd Resid. Dens. 0.96Ang From O31   1.78 eA-3

Author Response: Checked. Electron density could not be removed by appropriate absorption correction

PLAT971_ALERT_2_C Check Calcd Resid. Dens. 1.29Ang From N26   1.70 eA-3

Author Response: Checked. Electron density could not be removed by appropriate absorption correction

```
PLAT971_ALERT_2_C Check Calcd Resid. Dens.  1.23Ang From U1            1.63 eA-3
```

Author Response: Checked. Electron density could not be removed by appropriate absorption correction

```
PLAT971_ALERT_2_C Check Calcd Resid. Dens.  1.16Ang From Ni1           1.60 eA-3
```

Author Response: Checked. Electron density could not be removed by appropriate absorption correction

```
PLAT971_ALERT_2_C Check Calcd Resid. Dens.  1.13Ang From O15           1.57 eA-3
```

Author Response: Checked. Electron density could not be removed by appropriate absorption correction

```
PLAT971_ALERT_2_C Check Calcd Resid. Dens.  0.93Ang From S1            1.56 eA-3
```

Author Response: Checked. Electron density could not be removed by appropriate absorption correction

```
PLAT971_ALERT_2_C Check Calcd Resid. Dens.  0.78Ang From O10           1.52 eA-3
```

Author Response: Checked. Electron density could not be removed by appropriate absorption correction

```
PLAT972_ALERT_2_C Check Calcd Resid. Dens.  1.26Ang From O20          -2.38 eA-3
```

Author Response: Checked. Electron density could not be removed by appropriate absorption correction

```
PLAT972_ALERT_2_C Check Calcd Resid. Dens.  1.40Ang From O10          -2.27 eA-3
```

Author Response: Checked. Electron density could not be removed by appropriate absorption correction

```
PLAT972_ALERT_2_C Check Calcd Resid. Dens.  1.95Ang From C20          -1.85 eA-3
```

Author Response: Checked. Electron density could not be removed by appropriate absorption correction

```
PLAT972_ALERT_2_C Check Calcd Resid. Dens.  1.57Ang From O20           -1.80 eA-3
```

Author Response: Checked. Electron density could not be removed by appropriate absorption correction

```
PLAT972_ALERT_2_C Check Calcd Resid. Dens.  0.96Ang From O20           -1.74 eA-3
```

Author Response: Checked. Electron density could not be removed by appropriate absorption correction

```
PLAT972_ALERT_2_C Check Calcd Resid. Dens.  0.67Ang From U1            -1.67 eA-3
```

Author Response: Checked. Electron density could not be removed by appropriate absorption correction

```
PLAT972_ALERT_2_C Check Calcd Resid. Dens.  1.65Ang From O20           -1.62 eA-3
```

Author Response: Checked. Electron density could not be removed by appropriate absorption correction

```
PLAT977_ALERT_2_C Check Negative Difference Density on H7A       .      -0.34 eA-3
PLAT977_ALERT_2_C Check Negative Difference Density on H34A      .      -1.05 eA-3
PLAT977_ALERT_2_C Check Negative Difference Density on H34B      .      -0.32 eA-3
PLAT977_ALERT_2_C Check Negative Difference Density on H41       .      -0.36 eA-3
```

Alert level G

```
PLAT007_ALERT_5_G Number of Unrefined Donor-H Atoms .............          1 Report
             H41
PLAT083_ALERT_2_G SHELXL Second Parameter in WGHT  Unusually Large     835.14 Why ?
PLAT171_ALERT_4_G The CIF-Embedded .res File Contains EADP Records          7 Report
PLAT380_ALERT_4_G Incorrectly? Oriented X(sp2)-Methyl Moiety .....        C34 Check
PLAT720_ALERT_4_G Number of Unusual/Non-Standard Labels ..........          3 Note
             H8AA    H8AB    H8AC
PLAT794_ALERT_5_G Tentative Bond Valency for Ni1       (II)      .       2.02 Info
PLAT909_ALERT_3_G Percentage of I>2sig(I) Data at Theta(Max) Still        75% Note
PLAT910_ALERT_3_G Missing # of FCF Reflection(s) Below Theta(Min).          2 Note
               2  0  0,   0  0  2,
PLAT913_ALERT_3_G Missing # of Very Strong Reflections in FCF ....          2 Note
               2  0  0,   2  0  8,
PLAT933_ALERT_2_G Number of HKL-OMIT Records in Embedded .res File         19 Note
             -20  2  6, -18  2 14, -14  4  1, -14  6  2, -14  8  5,  -7  1 27,
               2  0  8,  12  2  4,  12  6  0,  12  8  3,  14  2  4,  14  4  3,
              14  6  0,  16  2  4,  16  2  6,  16  2 10,  16  8  3,  18  4  5,
              18  6  8,
PLAT967_ALERT_5_G Note: Two-Theta Cutoff Value in Embedded .res ..       49.5 Degree
PLAT969_ALERT_5_G The 'Henn et al.' R-Factor-gap value ..........      3.882 Note
             Predicted wR2: Based on SigI**2  5.35 or SHELX Weight 17.55
PLAT978_ALERT_2_G Number C-C Bonds with Positive Residual Density.          0 Info
```

```
 6 ALERT level A = Most likely a serious problem - resolve or explain
 5 ALERT level B = A potentially serious problem, consider carefully
33 ALERT level C = Check. Ensure it is not caused by an omission or oversight
13 ALERT level G = General information/check it is not something unexpected

 0 ALERT type 1 CIF construction/syntax error, inconsistent or missing data
40 ALERT type 2 Indicator that the structure model may be wrong or deficient
10 ALERT type 3 Indicator that the structure quality may be low
 3 ALERT type 4 Improvement, methodology, query or suggestion
 4 ALERT type 5 Informative message, check
```

Datablock: et2_co_5b

Bond precision: C-C = 0.0079 A Wavelength=0.71073

Cell:	a=18.801(1)	b=10.699(1)	c=28.381(1)
	alpha=90	beta=92.770(4)	gamma=90
Temperature:	173 K		

	Calculated	Reported
Volume	5702.2(6)	5702.2(6)
Space group	C 2/c	C 1 2/c 1
Hall group	-C 2yc	-C 2yc
Moiety formula	C38 H52 Co N10 O12 S4 U2, 2(C H2 Cl2)	C38 H52 Co N10 O12 S4 U2, 2(C H2 Cl2)
Sum formula	C40 H56 Cl4 Co N10 O12 S4 U2	C40 H56 Cl4 Co N10 O12 S4 U2
Mr	1673.98	1673.97
Dx,g cm-3	1.950	1.950
Z	4	4
Mu (mm-1)	6.351	6.351
F000	3220.0	3220.0
F000'	3150.15	
h,k,lmax	22,12,33	22,12,33
Nref	5020	4980
Tmin,Tmax	0.358,0.683	0.196,0.479
Tmin'	0.253	

Correction method= # Reported T Limits: Tmin=0.196 Tmax=0.479
AbsCorr = INTEGRATION

Data completeness= 0.992 Theta(max)= 24.997

```
R(reflections)= 0.0281( 4299)                      wR2(reflections)=
                                                   0.0653( 4980)
S = 1.047                  Npar= 331
```

The following ALERTS were generated. Each ALERT has the format
test-name_ALERT_alert-type_alert-level.
Click on the hyperlinks for more details of the test.

Alert level B

```
PLAT910_ALERT_3_B Missing # of FCF Reflection(s) Below Theta(Min).        12 Note
                 1  1  0,   2  0  0,  -1  1  1,   1  1  1,  -2  0  2,  -1  1  2,
                 0  0  2,   1  1  2,   2  0  2,  -1  1  3,   1  1  3,   0  0  4,
```

Author Response: This is due to the automated data collection routine of the IPDS

Alert level C

```
PLAT220_ALERT_2_C NonSolvent   Resd 1  C   Ueq(max)/Ueq(min) Range        3.1 Ratio
PLAT232_ALERT_2_C Hirshfeld Test Diff (M-X)  U1       --S1       .        5.3 s.u.
PLAT243_ALERT_4_C High   'Solvent' Ueq as Compared to Neighbors of        C54 Check
PLAT260_ALERT_2_C Large Average Ueq of Residue Including       Cl1      0.161 Check
PLAT413_ALERT_2_C Short Inter XH3 .. XHn     H10A     ..H54B     .       2.05 Ang.
                                          1/2-x,1/2-y,2-z  =      7_557 Check
PLAT906_ALERT_3_C Large K Value in the Analysis of Variance ......      2.145 Check
PLAT911_ALERT_3_C Missing FCF Refl Between Thmin & STh/L=    0.595        29 Report
                20  0  0,  20  4  0,  21  1  0,  22  0  0,  22  2  0, -22  2  1,
               -21  1  1, -20  4  1,  20  4  1,  21  3  1, -22  0  2,  19  1  2,
                20  0  2,  21  1  2,  22  0  2,  19  1  3,  20  2  3,  21  1  3,
               -22  0  4,  17  1  4,  18  0  4,  19  1  4,  20  0  4,  12  0  6,
                16  0  6,  12  0 24,  12  0 26,  11  1 27,  11  1 28,
```

Alert level G

```
PLAT012_ALERT_1_G N.O.K.   _shelx_res_checksum Found in CIF ......     Please Check
PLAT083_ALERT_2_G SHELXL Second Parameter in WGHT  Unusually Large      13.10 Why ?
PLAT153_ALERT_1_G The s.u.'s on the Cell Axes   are Equal ..(Note)      0.001 Ang.
PLAT344_ALERT_2_G Unusual sp3      Angle Range in Solvent/Ion for        C54 Check
PLAT480_ALERT_4_G Long H...A H-Bond Reported H20A     ..O31      .        2.62 Ang.
PLAT480_ALERT_4_G Long H...A H-Bond Reported H18C     ..O10      .        2.64 Ang.
PLAT794_ALERT_5_G Tentative Bond Valency for Co1       (II)      .        1.92 Info
PLAT909_ALERT_3_G Percentage of I>2sig(I) Data at Theta(Max) Still         76% Note
PLAT913_ALERT_3_G Missing # of Very Strong Reflections in FCF ....          1 Note
                 2  0  0,
PLAT941_ALERT_3_G Average HKL Measurement Multiplicity ..........         4.0 Low
PLAT967_ALERT_5_G Note: Two-Theta Cutoff Value in Embedded .res ..       50.0 Degree
PLAT969_ALERT_5_G The 'Henn et al.' R-Factor-gap value ..........      3.102 Note
               Predicted wR2: Based on SigI**2  2.11 or SHELX Weight  6.23
PLAT978_ALERT_2_G Number C-C Bonds with Positive Residual Density.          1 Info
```

---

0 **ALERT level A** = Most likely a serious problem - resolve or explain

1 **ALERT level B** = A potentially serious problem, consider carefully
7 **ALERT level C** = Check. Ensure it is not caused by an omission or oversight
13 **ALERT level G** = General information/check it is not something unexpected

2 ALERT type 1 CIF construction/syntax error, inconsistent or missing data
7 ALERT type 2 Indicator that the structure model may be wrong or deficient
6 ALERT type 3 Indicator that the structure quality may be low
3 ALERT type 4 Improvement, methodology, query or suggestion
3 ALERT type 5 Informative message, check

Datablock: et2_fe_5c

Bond precision: C-C = 0.0086 A Wavelength=0.71073

Cell:	a=18.774(2)	b=10.632(1)	c=28.219(3)
	alpha=90	beta=92.14(1)	gamma=90
Temperature:	473 K		

	Calculated	Reported
Volume	5628.7(10)	5628.7(10)
Space group	C 2/c	C 1 2/c 1
Hall group	-C 2yc	-C 2yc
Moiety formula	C38 H52 Fe N10 O12 S4 U2 [+ solvent]	C38 H52 Fe N10 O12 S4 U2, 4[H2O]
Sum formula	C38 H52 Fe N10 O12 S4 U2 [+ solvent]	C38 H60 Fe N10 O16 S4 U2
Mr	1501.05	1573.11
Dx,g cm-3	1.771	1.856
Z	4	4
Mu (mm-1)	6.202	6.212
F000	2880.0	3040.0
F000'	2807.79	
h,k,lmax	23,13,34	23,13,34
Nref	5536	5516
Tmin,Tmax	0.479,0.883	0.476,0.732
Tmin'	0.470	

Correction method= # Reported T Limits: Tmin=0.476 Tmax=0.732
AbsCorr = INTEGRATION

Data completeness= 0.996 Theta(max)= 25.998

R(reflections)= 0.0315(4241) wR2(reflections)= 0.0617(5516)

S = 0.922 Npar= 303

The following ALERTS were generated. Each ALERT has the format
test-name_ALERT_alert-type_alert-level.
Click on the hyperlinks for more details of the test.

Alert level B

```
PLAT910_ALERT_3_B Missing # of FCF Reflection(s) Below Theta(Min).         12 Note
                1  1  0,   2  0  0,  -1  1  1,   1  1  1,  -2  0  2,  -1  1  2,
                0  0  2,   1  1  2,   2  0  2,  -1  1  3,   1  1  3,   0  0  4,
```

Author Response: This due to the automated data collection routine of the IPDS

Alert level C

```
PLAT220_ALERT_2_C NonSolvent   Resd 1  C   Ueq(max)/Ueq(min) Range        3.3 Ratio
PLAT241_ALERT_2_C High   'MainMol' Ueq as Compared to Neighbors of        S11 Check
PLAT342_ALERT_3_C Low Bond Precision on  C-C Bonds ..............    0.00864 Ang.
PLAT906_ALERT_3_C Large K Value in the Analysis of Variance ......      2.459 Check
PLAT911_ALERT_3_C Missing FCF Refl Between Thmin & STh/L=    0.600          6 Report
                4  0  0,   6  0  0,   4  0  2,   6  0  2,   8  0  2,  10  0  2,
```

Alert level G

```
FORMU01_ALERT_2_G  There is a discrepancy between the atom counts in the
            _chemical_formula_sum and the formula from the _atom_site* data.
            Atom count from _chemical_formula_sum:C38 H60 Fe1 N10 O16 S4 U2
            Atom count from the _atom_site data:  C38 H52 Fe1 N10 O12 S4 U2
CELLZ01_ALERT_1_G Difference between formula and atom_site contents detected.
CELLZ01_ALERT_1_G ALERT: Large difference may be due to a
            symmetry error - see SYMMG tests
           From the CIF: _cell_formula_units_Z    4
           From the CIF: _chemical_formula_sum  C38 H60 Fe N10 O16 S4 U2
           TEST: Compare cell contents of formula and atom_site data

           atom    Z*formula  cif sites diff
           C        152.00    152.00    0.00
           H        240.00    208.00   32.00
           Fe         4.00      4.00    0.00
           N         40.00     40.00    0.00
           O         64.00     48.00   16.00
           S         16.00     16.00    0.00
           U          8.00      8.00    0.00
PLAT041_ALERT_1_G Calc. and Reported SumFormula    Strings  Differ     Please Check
             Calc: C38 H52 Fe N10 O12 S4 U2
             Rep.: C38 H60 Fe N10 O16 S4 U2
PLAT042_ALERT_1_G Calc. and Reported MoietyFormula Strings  Differ     Please Check
             Calc: C38 H52 Fe N10 O12 S4 U2
             Rep.: C38 H52 Fe N10 O12 S4 U2, 4[H2O]
PLAT380_ALERT_4_G Incorrectly? Oriented X(sp2)-Methyl Moiety .....        C34 Check
PLAT605_ALERT_4_G Largest Solvent Accessible VOID in the Structure        101 A**3
PLAT794_ALERT_5_G Tentative Bond Valency for Fe1       (II)      .       2.26 Info
PLAT868_ALERT_4_G ALERTS Due to the Use of _smtbx_masks Suppressed          ! Info
PLAT912_ALERT_4_G Missing # of FCF Reflections Above STh/L=  0.600          2 Note
```

```
PLAT913_ALERT_3_G Missing # of Very Strong Reflections in FCF ....          1 Note
                2  0  0,
PLAT941_ALERT_3_G Average HKL Measurement Multiplicity ..........         3.0 Low
PLAT969_ALERT_5_G The 'Henn et al.' R-Factor-gap value ..........       1.705 Note
              Predicted wR2: Based on SigI**2  3.62 or SHELX Weight  6.70
PLAT978_ALERT_2_G Number C-C Bonds with Positive Residual Density.          1 Info
```

---

0 **ALERT level A** = Most likely a serious problem - resolve or explain
1 **ALERT level B** = A potentially serious problem, consider carefully
5 **ALERT level C** = Check. Ensure it is not caused by an omission or oversight
14 **ALERT level G** = General information/check it is not something unexpected

4 ALERT type 1 CIF construction/syntax error, inconsistent or missing data
4 ALERT type 2 Indicator that the structure model may be wrong or deficient
6 ALERT type 3 Indicator that the structure quality may be low
4 ALERT type 4 Improvement, methodology, query or suggestion
2 ALERT type 5 Informative message, check

Datablock: et2_mn_5d

Bond precision:	C-C = 0.0077 A		Wavelength=0.71073
Cell:	a=9.1432(6)	b=20.9021(11)	c=31.498(2)
	alpha=90	beta=91.747(2)	gamma=90
Temperature:	293 K		

	Calculated	Reported
Volume	6016.9(6)	6016.9(6)
Space group	P 21/c	P 1 21/c 1
Hall group	-P 2ybc	-P 2ybc
Moiety formula	C38 H52 Mn N10 O12 S4 U2, 2(C H2 Cl2)	C38 H52 Mn N10 O12 S4 U2, 2(C H2 Cl2)
Sum formula	C40 H56 Cl4 Mn N10 O12 S4 U2	C40 H56 Cl4 Mn N10 O12 S4 U2
Mr	1669.99	1669.98
Dx,g cm-3	1.844	1.844
Z	4	4
Mu (mm-1)	5.953	5.953
F000	3212.0	3212.0
F000'	3142.09	
h,k,lmax	11,26,40	11,26,40
Nref	13443	13337
Tmin,Tmax	0.531,0.621	0.613,0.783
Tmin'	0.485	

```
Correction method= # Reported T Limits: Tmin=0.613 Tmax=0.783
AbsCorr = INTEGRATION

Data completeness= 0.992          Theta(max)= 27.207

R(reflections)= 0.0313( 12475)                  wR2(reflections)=
                                                0.0748( 13337)
S = 1.145                  Npar= 695
```

The following ALERTS were generated. Each ALERT has the format
test-name_ALERT_alert-type_alert-level.
Click on the hyperlinks for more details of the test.

Alert level B

```
PLAT220_ALERT_2_B NonSolvent   Resd 1  C   Ueq(max)/Ueq(min) Range        7.7 Ratio
```

Author Response: Assignment of atoms checked - no unusual results found

---

Alert level C

```
PLAT213_ALERT_2_C Atom C59              has ADP max/min Ratio .....        4.0 prolat
PLAT220_ALERT_2_C NonSolvent   Resd 1  N   Ueq(max)/Ueq(min) Range        3.1 Ratio
```

Author Response: Assignment of atoms checked - no unusual results found

```
PLAT222_ALERT_3_C NonSolvent Resd 1  H   Uiso(max)/Uiso(min) Range        6.8 Ratio
PLAT242_ALERT_2_C Low    'MainMol' Ueq as Compared to Neighbors of        C57 Check
PLAT244_ALERT_4_C Low    'Solvent' Ueq as Compared to Neighbors of        C81 Check
PLAT906_ALERT_3_C Large K Value in the Analysis of Variance ......      2.065 Check
PLAT910_ALERT_3_C Missing # of FCF Reflection(s) Below Theta(Min).          5 Note
                0  2  0,   0  1  1,   0  2  1,   0  0  2,   0  1  2,
PLAT911_ALERT_3_C Missing FCF Refl Between Thmin & STh/L=    0.600         44 Report
                1  0  0,   2  0  0,   0  4  0,   1  1  1,  -1  3  1,   2  3  1,
                0  4  1,  -2  0  2,   2  0  2,  -1  2  2,   0  2  2,   0  3  2,
                1  1  3,  -1  3  3,  -1  0  4,   0  0  4,   1  0  4,  -2  4  4,
               -1  4  4,   1  4  4,  -1  1  5,   0  1  5,   2  1  5,   1  4  5,
                0  0  6,   1  0  6,  -1  2  6,   1  2  6,  -1  1  7,   0  1  7,
               -3  3  7,  -1  3  9,  -1  0 10,   0  0 10,   0  3 11,   2  4 11,
               -1  7 13,   1  3 14,   0  3 20,   0  1 21,   0  1 27,  -1  4 27,
                2  1 28,   1  2 35,
PLAT971_ALERT_2_C Check Calcd Resid. Dens.  1.94Ang From Cl3            2.42 eA-3
PLAT971_ALERT_2_C Check Calcd Resid. Dens.  1.25Ang From Cl5            2.16 eA-3
PLAT971_ALERT_2_C Check Calcd Resid. Dens.  0.91Ang From Cl3            2.04 eA-3
PLAT971_ALERT_2_C Check Calcd Resid. Dens.  1.57Ang From C82            1.85 eA-3
PLAT971_ALERT_2_C Check Calcd Resid. Dens.  1.64Ang From Cl5            1.67 eA-3
PLAT972_ALERT_2_C Check Calcd Resid. Dens.  0.77Ang From Cl2           -1.57 eA-3
PLAT972_ALERT_2_C Check Calcd Resid. Dens.  0.83Ang From Cl1           -1.56 eA-3
PLAT976_ALERT_2_C Check Calcd Resid. Dens.  0.96Ang From O45     .     -0.58 eA-3
```

Alert level G

```
PLAT083_ALERT_2_G SHELXL Second Parameter in WGHT  Unusually Large      40.33 Why ?
PLAT199_ALERT_1_G Reported _cell_measurement_temperature ..... (K)        293 Check
PLAT200_ALERT_1_G Reported   _diffrn_ambient_temperature ..... (K)        293 Check
PLAT299_ALERT_4_G Atom Site Occupancy Constrained at ............         0.5 Check
              Cl3     Cl4     C82     H82A    H82B    Cl5     Cl6     C83
              H83A    H83B
PLAT302_ALERT_4_G Anion/Solvent/Minor-Residue Disorder (Resd    3)       100% Note
PLAT302_ALERT_4_G Anion/Solvent/Minor-Residue Disorder (Resd    4)       100% Note
PLAT304_ALERT_4_G Non-Integer Number of Atoms in ..... (Resd    3)       2.50 Check
PLAT304_ALERT_4_G Non-Integer Number of Atoms in ..... (Resd    4)       2.50 Check
PLAT432_ALERT_2_G Short Inter X...Y Contact  C63       ..C73      .       3.17 Ang.
                                                1+x,y,z  =      1_655 Check
PLAT434_ALERT_2_G Short Inter HL..HL Contact Cl1       ..Cl6      .       3.13 Ang.
                                         -x,1/2+y,1/2-z  =      2_555 Check
PLAT794_ALERT_5_G Tentative Bond Valency for Mn1       (II)       .       2.21 Info
PLAT912_ALERT_4_G Missing # of FCF Reflections Above STh/L=  0.600         57 Note
PLAT933_ALERT_2_G Number of HKL-OMIT Records in Embedded .res File         42 Note
               -3  3  7,  -2  0  2,  -2  4  4,  -1  0  4,  -1  0 10,  -1  1  5,
               -1  1  7,  -1  2  2,  -1  2  6,  -1  3  1,  -1  3  3,  -1  3  9,
               -1  4  4,  -1  4 27,  -1  7 13,   0  0  4,   0  0  6,   0  0 10,
                0  1  5,   0  1  7,   0  1 21,   0  1 27,   0  2  2,   0  3  2,
                0  3 11,   0  3 20,   0  4  1,   1  0  4,   1  0  6,   1  1  1,
                1  1  3,   1  2  6,   1  2 35,   1  3 14,   1  4  4,   1  4  5,
                2  0  0,   2  0  2,   2  1  5,   2  1 28,   2  3  1,   2  4 11,
PLAT969_ALERT_5_G The 'Henn et al.' R-Factor-gap value ..........      4.026 Note
              Predicted wR2: Based on SigI**2  1.86 or SHELX Weight  6.53
PLAT978_ALERT_2_G Number C-C Bonds with Positive Residual Density.          0 Info
```

```
   0 ALERT level A = Most likely a serious problem - resolve or explain
   1 ALERT level B = A potentially serious problem, consider carefully
  16 ALERT level C = Check. Ensure it is not caused by an omission or oversight
  15 ALERT level G = General information/check it is not something unexpected

   2 ALERT type 1 CIF construction/syntax error, inconsistent or missing data
  17 ALERT type 2 Indicator that the structure model may be wrong or deficient
   4 ALERT type 3 Indicator that the structure quality may be low
   7 ALERT type 4 Improvement, methodology, query or suggestion
   2 ALERT type 5 Informative message, check
```

Datablock: morph_co_6b

```
Bond precision:    C-C = 0.0104 A            Wavelength=0.71073

Cell:              a=44.055(5)     b=10.7980(12)        c=29.051(3)
                   alpha=90        beta=128.050(2)      gamma=90
Temperature:       100 K
```

	Calculated	Reported
Volume	10883(2)	10882.7(19)
Space group	C 2/c	C 1 2/c 1
Hall group	-C 2yc	-C 2yc
Moiety formula	2(C38 H44 Co N10 O16 S4 U2), C H2 Cl2 [+ solvent]	C38 H44 Co N10 O16 S4 U2, 0.5(C H2 Cl2), 0.5[CH4O], 1[CH4O]
Sum formula	C77 H90 Cl2 Co2 N20 O32 S8 U4 [+ solvent]	C40 H51 Cl Co N10 O17.50 S4 U2
Mr	3205.06	1650.58
Dx,g cm-3	1.956	2.015
Z	4	8
Mu (mm-1)	6.513	6.518
F000	6128.0	6344.0
F000'	5985.04	
h,k,lmax	55,13,36	55,13,36
Nref	11346	11239
Tmin,Tmax	0.584,0.722	0.555,0.745
Tmin'	0.349	

Correction method= # Reported T Limits: Tmin=0.555 Tmax=0.745
AbsCorr = MULTI-SCAN

Data completeness= 0.991 Theta(max)= 26.542

R(reflections)= 0.0387(9822) wR2(reflections)= 0.0706(11239)

S = 1.139 Npar= 668

The following ALERTS were generated. Each ALERT has the format
test-name_ALERT_alert-type_alert-level.
Click on the hyperlinks for more details of the test.

Alert level C

```
PLAT220_ALERT_2_C NonSolvent   Resd 1  C   Ueq(max)/Ueq(min) Range        4.3 Ratio
PLAT220_ALERT_2_C NonSolvent   Resd 1  O   Ueq(max)/Ueq(min) Range        3.9 Ratio
PLAT336_ALERT_2_C Long Bond Distance for ..... C71      -Cl2            1.920 Ang.
PLAT342_ALERT_3_C Low Bond Precision on  C-C Bonds ...............    0.01041 Ang.
PLAT413_ALERT_2_C Short Inter XH3 .. XHn     H34B     ..H57B     .      2.12 Ang.
                                        1/2-x,-1/2+y,1/2-z  =      4_545 Check
PLAT767_ALERT_4_C INS Embedded LIST 6 Instruction Should be LIST 4     Please Check
PLAT906_ALERT_3_C Large K Value in the Analysis of Variance ......      2.932 Check
PLAT910_ALERT_3_C Missing # of FCF Reflection(s) Below Theta(Min).         6 Note
               1  1  0,   2  0  0,  -1  1  1,  -4  0  2,  -2  0  2,   0  0  2,
PLAT971_ALERT_2_C Check Calcd Resid. Dens.  0.09Ang From Cl2            1.93 eA-3
PLAT971_ALERT_2_C Check Calcd Resid. Dens.  0.84Ang From U2             1.55 eA-3
PLAT972_ALERT_2_C Check Calcd Resid. Dens.  0.61Ang From Cl2           -1.79 eA-3
```

```
PLAT972_ALERT_2_C Check Calcd Resid. Dens.  1.52Ang From O30           -1.71 eA-3
PLAT972_ALERT_2_C Check Calcd Resid. Dens.  1.41Ang From O30           -1.60 eA-3
PLAT972_ALERT_2_C Check Calcd Resid. Dens.  2.14Ang From S1            -1.52 eA-3
PLAT975_ALERT_2_C Check Calcd Resid. Dens.  0.89Ang From O40     .      0.83 eA-3
PLAT976_ALERT_2_C Check Calcd Resid. Dens.  0.72Ang From O40     .     -0.91 eA-3
PLAT977_ALERT_2_C Check Negative Difference Density on H34A      .     -0.52 eA-3
PLAT977_ALERT_2_C Check Negative Difference Density on H34B      .     -0.46 eA-3
PLAT977_ALERT_2_C Check Negative Difference Density on H34C      .     -0.65 eA-3
PLAT977_ALERT_2_C Check Negative Difference Density on H74A      .     -0.53 eA-3
PLAT977_ALERT_2_C Check Negative Difference Density on H74B      .     -0.47 eA-3
```

Alert level G

```
FORMU01_ALERT_1_G  There is a discrepancy between the atom counts in the
            _chemical_formula_sum and _chemical_formula_moiety. This is
            usually due to the moiety formula being in the wrong format.
            Atom count from _chemical_formula_sum:   C40 H51 Cl1 Co1 N10 O17.5 S4
            Atom count from _chemical_formula_moiety:C39 H47 Cl1 Co1 N10 O16.5 S4
FORMU01_ALERT_2_G  There is a discrepancy between the atom counts in the
            _chemical_formula_sum and the formula from the _atom_site* data.
            Atom count from _chemical_formula_sum:C40 H51 Cl1 Co1 N10 O17.5 S4 U2
            Atom count from the _atom_site data:  C38.5 H45 Cl1 Co1 N10 O16 S4 U2
CELLZ01_ALERT_1_G Difference between formula and atom_site contents detected.
CELLZ01_ALERT_1_G ALERT: Large difference may be due to a
            symmetry error - see SYMMG tests
           From the CIF: _cell_formula_units_Z    8
           From the CIF: _chemical_formula_sum  C40 H51 Cl Co N10 O17.50 S4 U2
           TEST: Compare cell contents of formula and atom_site data

           atom    Z*formula  cif sites diff
           C        320.00    308.00   12.00
           H        408.00    360.00   48.00
           Cl         8.00      8.00    0.00
           Co         8.00      8.00    0.00
           N         80.00     80.00    0.00
           O        140.00    128.00   12.00
           S         32.00     32.00    0.00
           U         16.00     16.00    0.00
PLAT041_ALERT_1_G Calc. and Reported SumFormula    Strings  Differ     Please Check
             Calc: C38.50 H45 Cl Co N10 O16 S4 U2
             Rep.: C40 H51 Cl Co N10 O17.50 S4 U2
PLAT042_ALERT_1_G Calc. and Reported MoietyFormula Strings  Differ     Please Check
             Calc: 2(C38 H44 Co N10 O16 S4 U2), C H2 Cl2
             Rep.: C38 H44 Co N10 O16 S4 U2, 0.5(C H2 Cl2), 0.5[C
                   H4O], 1[CH4O]
PLAT045_ALERT_1_G Calculated and Reported Z Differ by a Factor ...      0.500 Check
PLAT083_ALERT_2_G SHELXL Second Parameter in WGHT  Unusually Large     194.91 Why ?
PLAT128_ALERT_4_G Alternate Setting for Input Space Group     C2/c       I2/a Note
PLAT232_ALERT_2_G Hirshfeld Test Diff (M-X)  U2       --S41      .        5.4 s.u.
PLAT299_ALERT_4_G Atom Site Occupancy Constrained at ............         0.5 Check
             Cl1     Cl2     C71     H71A    H71B
PLAT302_ALERT_4_G Anion/Solvent/Minor-Residue Disorder (Resd    2)       100% Note
PLAT304_ALERT_4_G Non-Integer Number of Atoms in ..... (Resd    2)       2.50 Check
PLAT380_ALERT_4_G Incorrectly? Oriented X(sp2)-Methyl Moiety .....        C34 Check
PLAT380_ALERT_4_G Incorrectly? Oriented X(sp2)-Methyl Moiety .....        C74 Check
PLAT398_ALERT_2_G Deviating  C-O-C   Angle From 120 for O11      .      109.3 Degree
PLAT398_ALERT_2_G Deviating  C-O-C   Angle From 120 for O21      .      109.8 Degree
```

```
PLAT398_ALERT_2_G Deviating  C-O-C   Angle From 120 for O51      .      109.5 Degree
PLAT605_ALERT_4_G Largest Solvent Accessible VOID in the Structure        133 A**3
PLAT794_ALERT_5_G Tentative Bond Valency for Co1       (II)      .       1.79 Info
PLAT868_ALERT_4_G ALERTS Due to the Use of _smtbx_masks Suppressed          ! Info
PLAT912_ALERT_4_G Missing # of FCF Reflections Above STh/L=  0.600        101 Note
PLAT969_ALERT_5_G The 'Henn et al.' R-Factor-gap value ..........      2.679 Note
              Predicted wR2: Based on SigI**2  2.64 or SHELX Weight  6.20
PLAT978_ALERT_2_G Number C-C Bonds with Positive Residual Density.          0 Info
```

```
   0 ALERT level A = Most likely a serious problem - resolve or explain
   0 ALERT level B = A potentially serious problem, consider carefully
  21 ALERT level C = Check. Ensure it is not caused by an omission or oversight
  24 ALERT level G = General information/check it is not something unexpected

   6 ALERT type 1 CIF construction/syntax error, inconsistent or missing data
  24 ALERT type 2 Indicator that the structure model may be wrong or deficient
   3 ALERT type 3 Indicator that the structure quality may be low
  10 ALERT type 4 Improvement, methodology, query or suggestion
   2 ALERT type 5 Informative message, check
```

Datablock: et2_ni_i_7

Bond precision: C-C = 0.0174 A   Wavelength=0.71073

Cell:	a=21.79(3)	b=9.958(14)	c=25.88(3)
	alpha=90	beta=101.36(3)	gamma=90
Temperature:	100 K		

	Calculated	Reported
Volume	5506(13)	5504(13)
Space group	P 21/n	P 1 21/n 1
Hall group	-P 2yn	-P 2yn
Moiety formula	C34 H46.05 I2 N10 Ni2 O6 S4 U, 2(C4 H8 O)	C34 H46.052 I2 N10 Ni2 O6 S4 U, 2(C4 H8 O)
Sum formula	C42 H62.05 I2 N10 Ni2 O8 S4 U	C42 H62.05 I2 N10 Ni2 O8 S4 U
Mr	1572.52	1572.56
Dx,g cm-3	1.897	1.898
Z	4	4
Mu (mm-1)	4.940	4.942
F000	3064.2	3064.0
F000'	3027.64	
h,k,lmax	25,11,30	25,11,30
Nref	9150	9123
Tmin,Tmax	0.708,0.952	0.576,0.745
Tmin'	0.261	

```
Correction method= # Reported T Limits: Tmin=0.576 Tmax=0.745
AbsCorr = MULTI SCAN

Data completeness= 0.997            Theta(max)= 24.498

                                                 wR2(reflections)=
R(reflections)= 0.0509( 8110)                    0.1048( 9123)

S = 1.066                  Npar= 407
```

The following ALERTS were generated. Each ALERT has the format
test-name_ALERT_alert-type_alert-level.
Click on the hyperlinks for more details of the test.

Alert level B

```
PLAT230_ALERT_2_B Hirshfeld Test Diff for    N6       --C7       .       10.0 s.u.
```

Author Response: Part of a disordered area of the refinement

```
PLAT230_ALERT_2_B Hirshfeld Test Diff for    N36      --C39      .        7.7 s.u.
```

Author Response: Part of a disordered area of the refinement

Alert level C

```
THETM01_ALERT_3_C  The value of sine(theta_max)/wavelength is less than 0.590
            Calculated sin(theta_max)/wavelength =    0.5834
PLAT042_ALERT_1_C Calc. and Reported MoietyFormula Strings  Differ     Please Check
              Calc: C34 H46.05 I2 N10 Ni2 O6 S4 U, 2(C4 H8 O)
              Rep.: C34 H46.052 I2 N10 Ni2 O6 S4 U, 2(C4 H8 O)
PLAT148_ALERT_3_C s.u. on the      a    - Axis is (Too) Large ....      0.030 Ang.
PLAT148_ALERT_3_C s.u. on the      b    - Axis is (Too) Large ....     0.0140 Ang.
PLAT148_ALERT_3_C s.u. on the      c    - Axis is (Too) Large ....      0.030 Ang.
PLAT213_ALERT_2_C Atom S21             has ADP max/min Ratio .....        3.4 prolat
PLAT230_ALERT_2_C Hirshfeld Test Diff for    S21      --C1       .        5.7 s.u.
```

Author Response: Part of a disordered area of the refinement

```
PLAT230_ALERT_2_C Hirshfeld Test Diff for    O15      --C14      .        5.5 s.u.
```

Author Response: Part of a disordered area of the refinement

```
PLAT230_ALERT_2_C Hirshfeld Test Diff for    O35      --C34      .        5.5 s.u.
```

Author Response: Part of a disordered area of the refinement

```
PLAT241_ALERT_2_C High   'MainMol' Ueq as Compared to Neighbors of        S21 Check
PLAT243_ALERT_4_C High   'Solvent' Ueq as Compared to Neighbors of        C62 Check
PLAT243_ALERT_4_C High   'Solvent' Ueq as Compared to Neighbors of        C65 Check
PLAT243_ALERT_4_C High   'Solvent' Ueq as Compared to Neighbors of        C72 Check
PLAT243_ALERT_4_C High   'Solvent' Ueq as Compared to Neighbors of        C75 Check
PLAT244_ALERT_4_C Low    'Solvent' Ueq as Compared to Neighbors of        O61 Check
PLAT244_ALERT_4_C Low    'Solvent' Ueq as Compared to Neighbors of        O71 Check
PLAT250_ALERT_2_C Large U3/U1 Ratio for <U(i,j)> Tensor(Resd    1)        2.1 Note
PLAT342_ALERT_3_C Low Bond Precision on  C-C Bonds ..............    0.01743 Ang.
PLAT767_ALERT_4_C INS Embedded LIST 6 Instruction Should be LIST 4     Please Check
PLAT906_ALERT_3_C Large K Value in the Analysis of Variance ......      2.346 Check
PLAT910_ALERT_3_C Missing # of FCF Reflection(s) Below Theta(Min).          6 Note
                2  0  0,  -1  0  1,   0  1  1,   1  0  1,  -2  0  2,   0  0  2,
PLAT911_ALERT_3_C Missing FCF Refl Between Thmin & STh/L=    0.583         20 Report
                0  2  0,   2  8  3,   0  0  4,   0  9  5,   2  0  6, -14  0 10,
               -4  0 12,  13  0 13,  17  0 13,  18  0 14,  -9  1 15,  -8  6 16,
               12  0 16,  11  0 17,  -6  0 18,  -3  0 21,  -9  2 22,  -3  3 24,
               -5  6 25,  -8  2 27,
PLAT971_ALERT_2_C Check Calcd Resid. Dens.  1.04Ang From C72             2.35 eA-3
PLAT971_ALERT_2_C Check Calcd Resid. Dens.  0.97Ang From C75             2.02 eA-3
PLAT971_ALERT_2_C Check Calcd Resid. Dens.  0.92Ang From N36             1.95 eA-3
PLAT971_ALERT_2_C Check Calcd Resid. Dens.  1.20Ang From C72             1.77 eA-3
PLAT971_ALERT_2_C Check Calcd Resid. Dens.  0.37Ang From S21             1.76 eA-3
PLAT971_ALERT_2_C Check Calcd Resid. Dens.  0.09Ang From N6              1.62 eA-3
PLAT971_ALERT_2_C Check Calcd Resid. Dens.  0.87Ang From N33             1.61 eA-3
PLAT972_ALERT_2_C Check Calcd Resid. Dens.  0.51Ang From O61            -2.32 eA-3
PLAT972_ALERT_2_C Check Calcd Resid. Dens.  0.21Ang From N36            -2.02 eA-3
PLAT972_ALERT_2_C Check Calcd Resid. Dens.  0.14Ang From N33            -1.99 eA-3
PLAT972_ALERT_2_C Check Calcd Resid. Dens.  0.41Ang From O61            -1.87 eA-3
PLAT972_ALERT_2_C Check Calcd Resid. Dens.  0.15Ang From C18            -1.73 eA-3
PLAT972_ALERT_2_C Check Calcd Resid. Dens.  0.03Ang From C1             -1.60 eA-3
PLAT972_ALERT_2_C Check Calcd Resid. Dens.  1.11Ang From U1             -1.58 eA-3
PLAT972_ALERT_2_C Check Calcd Resid. Dens.  0.83Ang From O2             -1.52 eA-3
PLAT977_ALERT_2_C Check Negative Difference Density on H65A      .      -0.34 eA-3
```

Alert level G

```
PLAT002_ALERT_2_G Number of Distance or Angle Restraints on AtSite         14 Note
PLAT068_ALERT_1_G Reported F000 Differs from Calcd (or Missing)...     Please Check
PLAT083_ALERT_2_G SHELXL Second Parameter in WGHT  Unusually Large     119.89 Why ?
PLAT171_ALERT_4_G The CIF-Embedded .res File Contains EADP Records         14 Report
PLAT176_ALERT_4_G The CIF-Embedded .res File Contains SADI Records          4 Report
PLAT230_ALERT_2_G Hirshfeld Test Diff for    N36      --C37A     .        7.0 s.u.
```

Author Response: Part of a disordered area of the refinement

```
PLAT230_ALERT_2_G Hirshfeld Test Diff for    N36      --C37B     .        6.7 s.u.
```

Author Response: Part of a disordered area of the refinement

```
PLAT232_ALERT_2_G Hirshfeld Test Diff (M-X)  I2C      --Ni2      .        5.6 s.u.
PLAT299_ALERT_4_G Atom Site Occupancy Constrained at ............         0.5 Check
              I2B     I2C     C3      C27     H3A     H3B     H3C     H27A
              H27B
```

```
PLAT301_ALERT_3_G Main Residue  Disorder ..............(Resd    1)        14% Note
PLAT304_ALERT_4_G Non-Integer Number of Atoms in ..... (Resd    1)     105.05 Check
PLAT398_ALERT_2_G Deviating  C-O-C   Angle From 120 for O71      .       96.9 Degree
PLAT413_ALERT_2_G Short Inter XH3 .. XHn     H75A     ..H30C     .       1.98 Ang.
                                                 x,y,-1+z  =      1_554 Check
PLAT794_ALERT_5_G Tentative Bond Valency for U1        (VI)      .       5.20 Info
PLAT860_ALERT_3_G Number of Least-Squares Restraints ............          10 Note
PLAT909_ALERT_3_G Percentage of I>2sig(I) Data at Theta(Max) Still        77% Note
PLAT913_ALERT_3_G Missing # of Very Strong Reflections in FCF ....          1 Note
                2  0  6,
PLAT933_ALERT_2_G Number of HKL-OMIT Records in Embedded .res File         17 Note
              -14  0 10,  -9  1 15,  -9  2 22,  -8  2 27,  -8  6 16,  -6  0 18,
               -5  6 25,  -4  0 12,  -3  0 21,  -3  3 24,  -2  0  2,   0  0  4,
                0  1  1,   0  2  0,   0  9  5,   2  0  6,   2  8  3,
PLAT960_ALERT_3_G Number of Intensities with I  <   - 2*sig(I) ...          2 Check
PLAT967_ALERT_5_G Note: Two-Theta Cutoff Value in Embedded .res ..       49.0 Degree
PLAT969_ALERT_5_G The 'Henn et al.' R-Factor-gap value ..........      5.478 Note
              Predicted wR2: Based on SigI**2  1.91 or SHELX Weight  9.84
PLAT978_ALERT_2_G Number C-C Bonds with Positive Residual Density.          0 Info
```

---

0 **ALERT level A** = Most likely a serious problem - resolve or explain
2 **ALERT level B** = A potentially serious problem, consider carefully
38 **ALERT level C** = Check. Ensure it is not caused by an omission or oversight
22 **ALERT level G** = General information/check it is not something unexpected

2 ALERT type 1 CIF construction/syntax error, inconsistent or missing data
33 ALERT type 2 Indicator that the structure model may be wrong or deficient
13 ALERT type 3 Indicator that the structure quality may be low
11 ALERT type 4 Improvement, methodology, query or suggestion
3 ALERT type 5 Informative message, check

Datablock: morph_antianti_8

Bond precision: C-C = 0.0080 A   Wavelength=0.71073

Cell:	a=12.009(6)	b=14.640(9)	c=25.11(2)
	alpha=91.38(2)	beta=90.96(2)	gamma=94.32(1)
Temperature:	174 K		

	Calculated	Reported
Volume	4400(5)	4400(5)
Space group	P -1	P -1
Hall group	-P 1	-P 1
Moiety formula	C36 H40 N10 O14 S4 U2, C36 H44 N10 O14 S4 U2, 4(C20 H20 P) [+ s	C18 H20 N5 O7 S2 U, C18 H22 N5 O7 S2 U, 2(C20 H20 P), 4[CH4O]
Sum formula	C152 H164 N20 O28 P4 S8 U4 [+ solvent]	C80 H98 N10 O18 P2 S4 U2
Mr	4051.51	2153.92
Dx,g cm-3	1.529	1.626
Z	1	2
Mu (mm-1)	3.869	3.878
F000	1996.0	2140.0
F000'	1959.66	
h,k,lmax	14,17,29	14,17,29
Nref	14611	14564
Tmin,Tmax	0.417,0.604	0.573,0.746
Tmin'	0.406	

Correction method= # Reported T Limits: Tmin=0.573 Tmax=0.746
AbsCorr = MULTI-SCAN

Data completeness= 0.997 Theta(max)= 24.496

R(reflections)= 0.0313(13982) wR2(reflections)= 0.0700(14564)

S = 1.041 Npar= 883

The following ALERTS were generated. Each ALERT has the format
test-name_ALERT_alert-type_alert-level.
Click on the hyperlinks for more details of the test.

Alert level C

```
THETM01_ALERT_3_C  The value of sine(theta_max)/wavelength is less than 0.590
            Calculated sin(theta_max)/wavelength =    0.5834
PLAT220_ALERT_2_C NonSolvent   Resd 1  C   Ueq(max)/Ueq(min) Range        3.8 Ratio
PLAT220_ALERT_2_C NonSolvent   Resd 1  N   Ueq(max)/Ueq(min) Range        4.0 Ratio
PLAT220_ALERT_2_C NonSolvent   Resd 1  O   Ueq(max)/Ueq(min) Range        4.0 Ratio
PLAT220_ALERT_2_C NonSolvent   Resd 2  C   Ueq(max)/Ueq(min) Range        4.7 Ratio
PLAT220_ALERT_2_C NonSolvent   Resd 2  N   Ueq(max)/Ueq(min) Range        3.4 Ratio
PLAT220_ALERT_2_C NonSolvent   Resd 2  O   Ueq(max)/Ueq(min) Range        4.4 Ratio
PLAT230_ALERT_2_C Hirshfeld Test Diff for    C27      --C28       .       5.5 s.u.
PLAT241_ALERT_2_C High   'MainMol' Ueq as Compared to Neighbors of        C2 Check
PLAT241_ALERT_2_C High   'MainMol' Ueq as Compared to Neighbors of       C37 Check
PLAT241_ALERT_2_C High   'MainMol' Ueq as Compared to Neighbors of      C122 Check
PLAT241_ALERT_2_C High   'MainMol' Ueq as Compared to Neighbors of      C132 Check
```

```
PLAT242_ALERT_2_C Low    'MainMol' Ueq as Compared to Neighbors of         N3 Check
PLAT242_ALERT_2_C Low    'MainMol' Ueq as Compared to Neighbors of       C133 Check
PLAT242_ALERT_2_C Low    'MainMol' Ueq as Compared to Neighbors of       C136 Check
PLAT767_ALERT_4_C INS Embedded LIST 6 Instruction Should be LIST 4     Please Check
PLAT910_ALERT_3_C Missing # of FCF Reflection(s) Below Theta(Min).         10 Note
                1  0  0,  -1  1  0,   0  1  0,   0 -1  1,  -1  0  1,   0  0  1,
                1  0  1,   0  1  1,   0 -1  2,   0  0  2,
PLAT911_ALERT_3_C Missing FCF Refl Between Thmin & STh/L=    0.583         37 Report
                4  1  0,   2  3  0,  -1 -5  1,  -1 -1  1,   2  1  1,   0  3  1,
                4  3  1,   5  3  1,  -1  0  2,  -1  2  2,   0  2  2,   4  2  2,
                2  4  2,   3 -5  3,   5  1  3,   5  2  3,   1  3  3,   3  4  3,
                5  4  3,   3 -4  4,   2 -5  5,   2  1  5,   3  1  5,   3  3  5,
                3  4  5,   2 -4  6,   2 -1  6,   2  2  7,   3  2  7,   2  3  8,
                0-13 14,  -2-12 15,   0-12 17,  -2 -7 22,   0 -7 23,  -1 -2 25,
                1 -1 26,
PLAT971_ALERT_2_C Check Calcd Resid. Dens.  0.21Ang From P1             1.79 eA-3
PLAT971_ALERT_2_C Check Calcd Resid. Dens.  0.08Ang From C2             1.65 eA-3
PLAT972_ALERT_2_C Check Calcd Resid. Dens.  0.09Ang From O40           -1.95 eA-3
PLAT972_ALERT_2_C Check Calcd Resid. Dens.  0.08Ang From C93           -1.70 eA-3
PLAT972_ALERT_2_C Check Calcd Resid. Dens.  0.18Ang From N1            -1.59 eA-3
PLAT975_ALERT_2_C Check Calcd Resid. Dens.  1.03Ang From O40     .      1.49 eA-3
```

Alert level G

```
FORMU01_ALERT_2_G  There is a discrepancy between the atom counts in the
            _chemical_formula_sum and the formula from the _atom_site* data.
            Atom count from _chemical_formula_sum:C80 H98 N10 O18 P2 S4 U2
            Atom count from the _atom_site data:  C76 H82 N10 O14 P2 S4 U2
CELLZ01_ALERT_1_G Difference between formula and atom_site contents detected.
CELLZ01_ALERT_1_G ALERT: Large difference may be due to a
            symmetry error - see SYMMG tests
           From the CIF: _cell_formula_units_Z    2
           From the CIF: _chemical_formula_sum  C80 H98 N10 O18 P2 S4 U2
           TEST: Compare cell contents of formula and atom_site data

           atom    Z*formula  cif sites diff
           C        160.00    152.00    8.00
           H        196.00    164.00   32.00
           N         20.00     20.00    0.00
           O         36.00     28.00    8.00
           P          4.00      4.00    0.00
           S          8.00      8.00    0.00
           U          4.00      4.00    0.00
PLAT002_ALERT_2_G Number of Distance or Angle Restraints on AtSite          6 Note
PLAT041_ALERT_1_G Calc. and Reported SumFormula    Strings  Differ     Please Check
              Calc: C76 H82 N10 O14 P2 S4 U2
              Rep.: C80 H98 N10 O18 P2 S4 U2
PLAT042_ALERT_1_G Calc. and Reported MoietyFormula Strings  Differ     Please Check
              Calc: C36 H40 N10 O14 S4 U2, C36 H44 N10 O14 S4 U2, 4(C20 H20 P)
              Rep.: C18 H20 N5 O7 S2 U, C18 H22 N5 O7 S2 U, 2(C20
                    H20 P), 4[CH4O]
PLAT045_ALERT_1_G Calculated and Reported Z Differ by a Factor ...      0.500 Check
PLAT083_ALERT_2_G SHELXL Second Parameter in WGHT  Unusually Large      27.22 Why ?
PLAT171_ALERT_4_G The CIF-Embedded .res File Contains EADP Records         10 Report
PLAT176_ALERT_4_G The CIF-Embedded .res File Contains SADI Records          2 Report
PLAT187_ALERT_4_G The CIF-Embedded .res File Contains RIGU Records          1 Report
PLAT230_ALERT_2_G Hirshfeld Test Diff for    N1       --C7A      .       11.0 s.u.
```

```
PLAT230_ALERT_2_G Hirshfeld Test Diff for    N1       --C9A      .        9.0 s.u.
PLAT230_ALERT_2_G Hirshfeld Test Diff for    N1       --C9B      .        8.7 s.u.
PLAT299_ALERT_4_G Atom Site Occupancy Constrained at ............        0.5 Check
              C1      C3      C5      C6      C7      C9      C75     C76
              C77     C78     C79     C80     H1      H3      H5      H6
              H7      H75     H76     H77     H78     H79
PLAT301_ALERT_3_G Main Residue  Disorder .............(Resd    1)         6% Note
PLAT302_ALERT_4_G Anion/Solvent/Minor-Residue Disorder (Resd    3)        29% Note
PLAT398_ALERT_2_G Deviating  C-O-C   Angle From 120 for O40      .      108.1 Degree
PLAT411_ALERT_2_G Short Inter H...H Contact  H6       ..H102     .       2.05 Ang.
                                                   1+x,y,z  =      1_655 Check
PLAT605_ALERT_4_G Largest Solvent Accessible VOID in the Structure        275 A**3
PLAT720_ALERT_4_G Number of Unusual/Non-Standard Labels .........           8 Note
              H7AA    H7AB    H9AA    H9AB    H7BA    H7BB    H9BA    H9BB
PLAT764_ALERT_4_G Overcomplete CIF Bond List Detected (Rep/Expd) .       1.15 Ratio
PLAT773_ALERT_2_G Check long C-C Bond in CIF: C80      --C7              1.79 Ang.
PLAT773_ALERT_2_G Check long C-C Bond in CIF: C75      --C1              1.75 Ang.
PLAT773_ALERT_2_G Check long C-C Bond in CIF: C76      --C3              2.04 Ang.
PLAT773_ALERT_2_G Check long C-C Bond in CIF: C78      --C7              1.79 Ang.
PLAT773_ALERT_2_G Check long C-C Bond in CIF: C78      --C1              1.99 Ang.
PLAT779_ALERT_4_G Suspect or Irrelevant (Bond) Angle(s) in CIF ...      16.60 Deg.
              C80  -P2   -C9      1_555   1_555   1_555 ......     #  279 Check
PLAT779_ALERT_4_G Suspect or Irrelevant (Bond) Angle(s) in CIF ...      30.50 Deg.
              C79  -C80  -C7      1_555   1_555   1_555 ......     #  399 Check
PLAT779_ALERT_4_G Suspect or Irrelevant (Bond) Angle(s) in CIF ...      22.70 Deg.
              C5   -C80  -C75     1_555   1_555   1_555 ......     #  401 Check
PLAT779_ALERT_4_G Suspect or Irrelevant (Bond) Angle(s) in CIF ...      34.00 Deg.
              C9   -C80  -C7      1_555   1_555   1_555 ......     #  407 Check
PLAT779_ALERT_4_G Suspect or Irrelevant (Bond) Angle(s) in CIF ...      17.30 Deg.
              C80  -C75  -C9      1_555   1_555   1_555 ......     #  411 Check
PLAT779_ALERT_4_G Suspect or Irrelevant (Bond) Angle(s) in CIF ...      24.90 Deg.
              C76  -C75  -C1      1_555   1_555   1_555 ......     #  414 Check
PLAT779_ALERT_4_G Suspect or Irrelevant (Bond) Angle(s) in CIF ...      44.00 Deg.
              C5   -C75  -C1      1_555   1_555   1_555 ......     #  420 Check
PLAT779_ALERT_4_G Suspect or Irrelevant (Bond) Angle(s) in CIF ...      29.40 Deg.
              C77  -C76  -C3      1_555   1_555   1_555 ......     #  428 Check
PLAT779_ALERT_4_G Suspect or Irrelevant (Bond) Angle(s) in CIF ...      16.20 Deg.
              C1   -C76  -C3      1_555   1_555   1_555 ......     #  433 Check
PLAT779_ALERT_4_G Suspect or Irrelevant (Bond) Angle(s) in CIF ...      22.80 Deg.
              C5   -C76  -C75     1_555   1_555   1_555 ......     #  435 Check
PLAT779_ALERT_4_G Suspect or Irrelevant (Bond) Angle(s) in CIF ...      43.20 Deg.
              C78  -C79  -C6      1_555   1_555   1_555 ......     #  443 Check
PLAT779_ALERT_4_G Suspect or Irrelevant (Bond) Angle(s) in CIF ...      22.50 Deg.
              C9   -C79  -C80     1_555   1_555   1_555 ......     #  450 Check
PLAT779_ALERT_4_G Suspect or Irrelevant (Bond) Angle(s) in CIF ...      31.40 Deg.
              C1   -C77  -C76     1_555   1_555   1_555 ......     #  460 Check
PLAT779_ALERT_4_G Suspect or Irrelevant (Bond) Angle(s) in CIF ...      30.60 Deg.
              C79  -C78  -C7      1_555   1_555   1_555 ......     #  465 Check
PLAT779_ALERT_4_G Suspect or Irrelevant (Bond) Angle(s) in CIF ...      44.70 Deg.
              C77  -C78  -C3      1_555   1_555   1_555 ......     #  471 Check
PLAT779_ALERT_4_G Suspect or Irrelevant (Bond) Angle(s) in CIF ...      29.30 Deg.
              C77  -C78  -C1      1_555   1_555   1_555 ......     #  472 Check
PLAT779_ALERT_4_G Suspect or Irrelevant (Bond) Angle(s) in CIF ...      41.60 Deg.
              C3   -C78  -C1      1_555   1_555   1_555 ......     #  483 Check
PLAT779_ALERT_4_G Suspect or Irrelevant (Bond) Angle(s) in CIF ...      33.40 Deg.
              C7   -C6   -C79     1_555   1_555   1_555 ......     #  496 Check
PLAT779_ALERT_4_G Suspect or Irrelevant (Bond) Angle(s) in CIF ...      40.40 Deg.
```

```
             C6   -C7   -C78     1_555   1_555   1_555 ......     #  510 Check
PLAT779_ALERT_4_G Suspect or Irrelevant (Bond) Angle(s) in CIF ...      12.60 Deg.
             C9   -C7   -C80     1_555   1_555   1_555 ......     #  512 Check
PLAT779_ALERT_4_G Suspect or Irrelevant (Bond) Angle(s) in CIF ...      41.00 Deg.
             C77  -C3   -C76     1_555   1_555   1_555 ......     #  517 Check
PLAT779_ALERT_4_G Suspect or Irrelevant (Bond) Angle(s) in CIF ...       9.20 Deg.
             C1   -C3   -C76     1_555   1_555   1_555 ......     #  527 Check
PLAT779_ALERT_4_G Suspect or Irrelevant (Bond) Angle(s) in CIF ...      40.80 Deg.
             C77  -C1   -C78     1_555   1_555   1_555 ......     #  540 Check
PLAT779_ALERT_4_G Suspect or Irrelevant (Bond) Angle(s) in CIF ...      44.60 Deg.
             C3   -C1   -C78     1_555   1_555   1_555 ......     #  546 Check
PLAT779_ALERT_4_G Suspect or Irrelevant (Bond) Angle(s) in CIF ...      15.50 Deg.
             C5   -C1   -C75     1_555   1_555   1_555 ......     #  549 Check
PLAT779_ALERT_4_G Suspect or Irrelevant (Bond) Angle(s) in CIF ...      22.70 Deg.
             C80  -C5   -C9      1_555   1_555   1_555 ......     #  555 Check
PLAT779_ALERT_4_G Suspect or Irrelevant (Bond) Angle(s) in CIF ...      27.60 Deg.
             C75  -C5   -H5      1_555   1_555   1_555 ......     #  559 Check
PLAT779_ALERT_4_G Suspect or Irrelevant (Bond) Angle(s) in CIF ...      32.30 Deg.
             C76  -C5   -C1      1_555   1_555   1_555 ......     #  561 Check
PLAT779_ALERT_4_G Suspect or Irrelevant (Bond) Angle(s) in CIF ...      40.50 Deg.
             C79  -C9   -C7      1_555   1_555   1_555 ......     #  575 Check
PLAT779_ALERT_4_G Suspect or Irrelevant (Bond) Angle(s) in CIF ...      17.40 Deg.
             C5   -C9   -C75     1_555   1_555   1_555 ......     #  581 Check
PLAT789_ALERT_4_G Atoms with Negative _atom_site_disorder_group  #         11 Check
PLAT794_ALERT_5_G Tentative Bond Valency for U1        (VI)      .       5.53 Info
PLAT794_ALERT_5_G Tentative Bond Valency for U2        (VI)      .       5.53 Info
PLAT822_ALERT_4_G CIF-embedded .res Contains Negative PART Numbers          1 Check
PLAT860_ALERT_3_G Number of Least-Squares Restraints ............          38 Note
PLAT868_ALERT_4_G ALERTS Due to the Use of _smtbx_masks Suppressed          ! Info
PLAT883_ALERT_1_G No Info/Value for _atom_sites_solution_primary .     Please Do !
PLAT909_ALERT_3_G Percentage of I>2sig(I) Data at Theta(Max) Still        91% Note
PLAT913_ALERT_3_G Missing # of Very Strong Reflections in FCF ....          2 Note
               0  1  1,   2  1  5,
PLAT933_ALERT_2_G Number of HKL-OMIT Records in Embedded .res File         20 Note
              -2-12 15,   0-13 14,   0  2  2,   0  3  1,   1  3  3,   2 -1  6,
               2  1  1,   2  1  5,   2  2  7,   2  3  0,   2  3  8,   2  4  2,
               3  2  7,   3  4  3,   3  4  5,   4  1  0,   5  1  3,   5  2  3,
               5  3  1,   5  4  3,
PLAT967_ALERT_5_G Note: Two-Theta Cutoff Value in Embedded .res ..       49.0 Degree
PLAT969_ALERT_5_G The 'Henn et al.' R-Factor-gap value ..........       3.078 Note
             Predicted wR2: Based on SigI**2  2.28 or SHELX Weight  6.72
PLAT978_ALERT_2_G Number C-C Bonds with Positive Residual Density.          2 Info
```

---

0 **ALERT level A** = Most likely a serious problem - resolve or explain
0 **ALERT level B** = A potentially serious problem, consider carefully
24 **ALERT level C** = Check. Ensure it is not caused by an omission or oversight
70 **ALERT level G** = General information/check it is not something unexpected

6 ALERT type 1 CIF construction/syntax error, inconsistent or missing data
35 ALERT type 2 Indicator that the structure model may be wrong or deficient
7 ALERT type 3 Indicator that the structure quality may be low
42 ALERT type 4 Improvement, methodology, query or suggestion
4 ALERT type 5 Informative message, check

It is advisable to attempt to resolve as many as possible of the alerts in all categories. Often the minor alerts point to easily fixed oversights, errors and omissions in your CIF or refinement strategy, so attention to these fine details can be worthwhile. In order to resolve some of the more serious problems it may be necessary to carry out additional measurements or structure refinements. However, the purpose of your study may justify the reported deviations and the more serious of these should normally be commented upon in the discussion or experimental section of a paper or in the "special_details" fields of the CIF. checkCIF was carefully designed to identify outliers and unusual parameters, but every test has its limitations and alerts that are not important in a particular case may appear. Conversely, the absence of alerts does not guarantee there are no aspects of the results needing attention. It is up to the individual to critically assess their own results and, if necessary, seek expert advice.

Publication of your CIF in IUCr journals

A basic structural check has been run on your CIF. These basic checks will be run on all CIFs submitted for publication in IUCr journals (*Acta Crystallographica*, *Journal of Applied Crystallography*, *Journal of Synchrotron Radiation*); however, if you intend to submit to *Acta Crystallographica Section C* or *E* or *IUCrData*, you should make sure that full publication checks are run on the final version of your CIF prior to submission.

Publication of your CIF in other journals

Please refer to the *Notes for Authors* of the relevant journal for any special instructions relating to CIF submission.

PLATON version of 22/08/2024; check.def file version of 21/08/2024

Datablock Et2_antianti_1 - ellipsoid plot

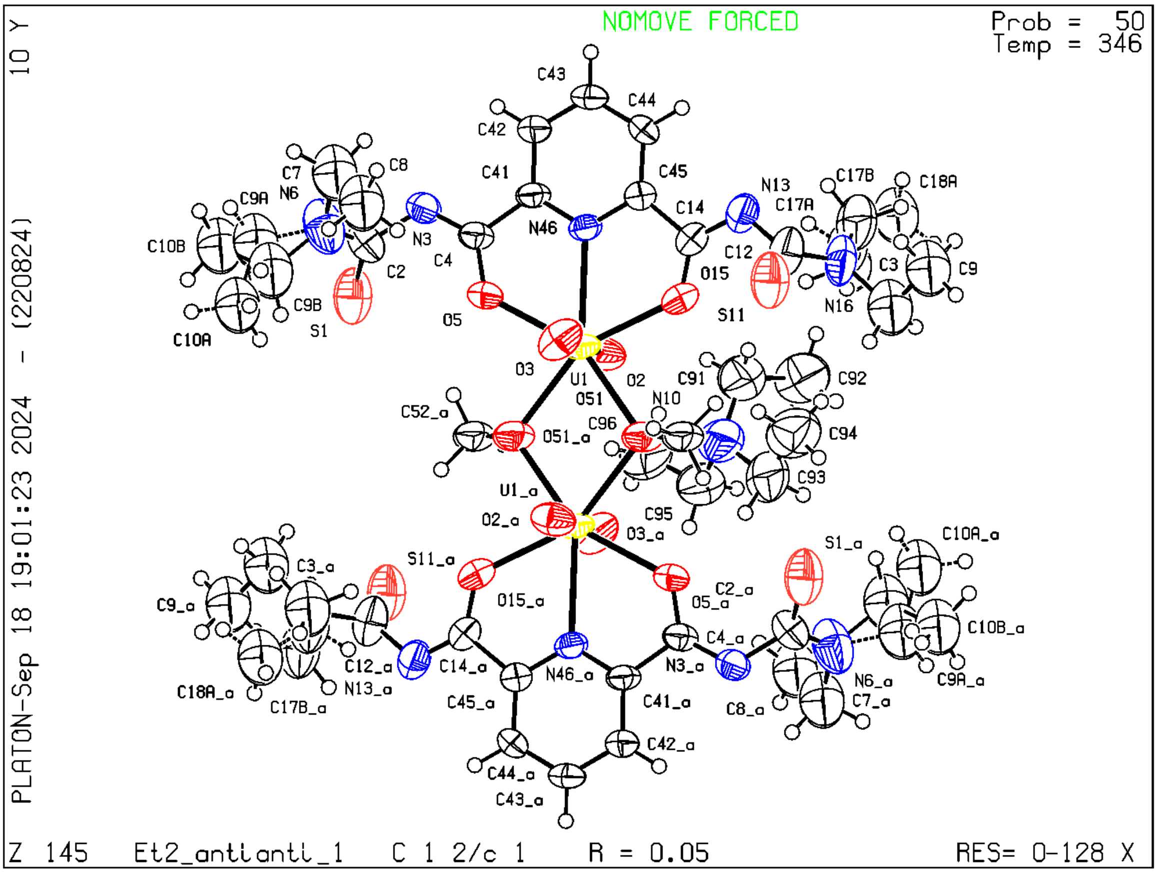

Datablock Et2_synanti_1 - ellipsoid plot

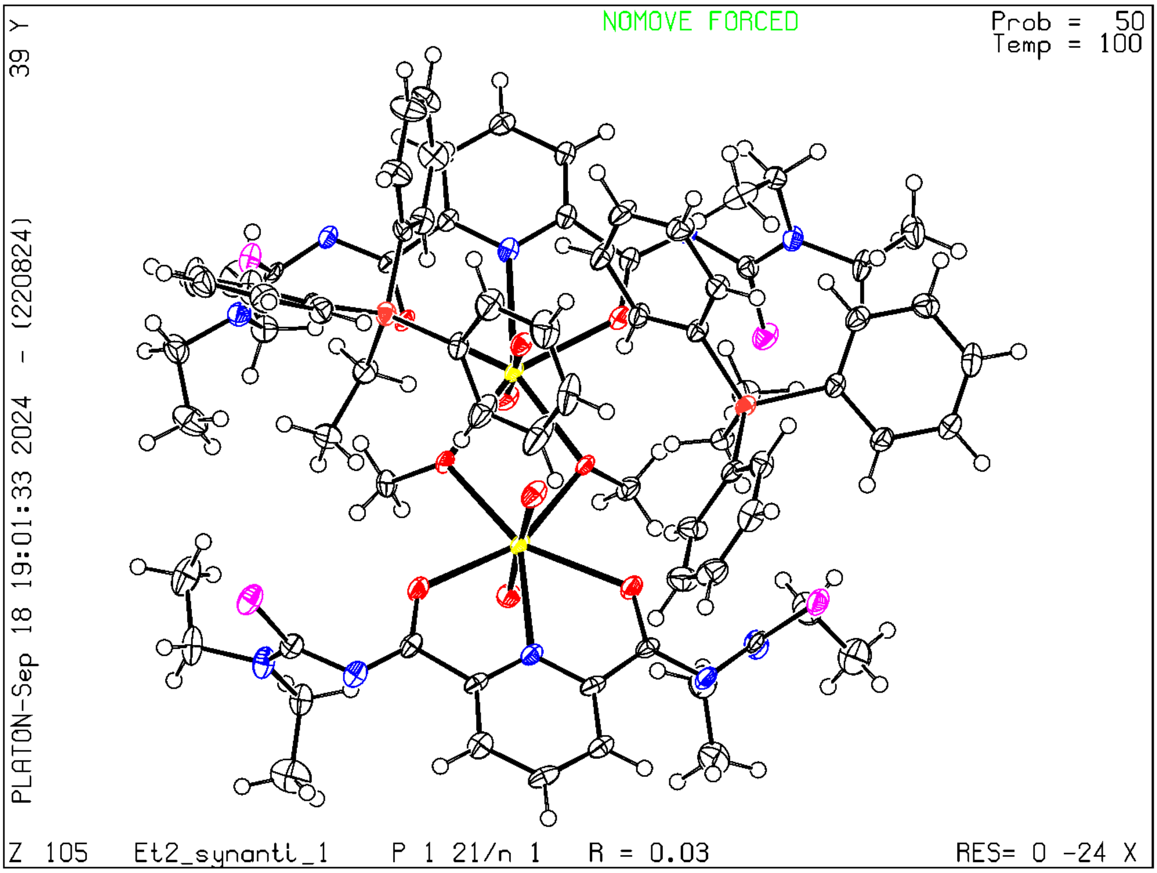

Datablock et2_pb_3 - ellipsoid plot

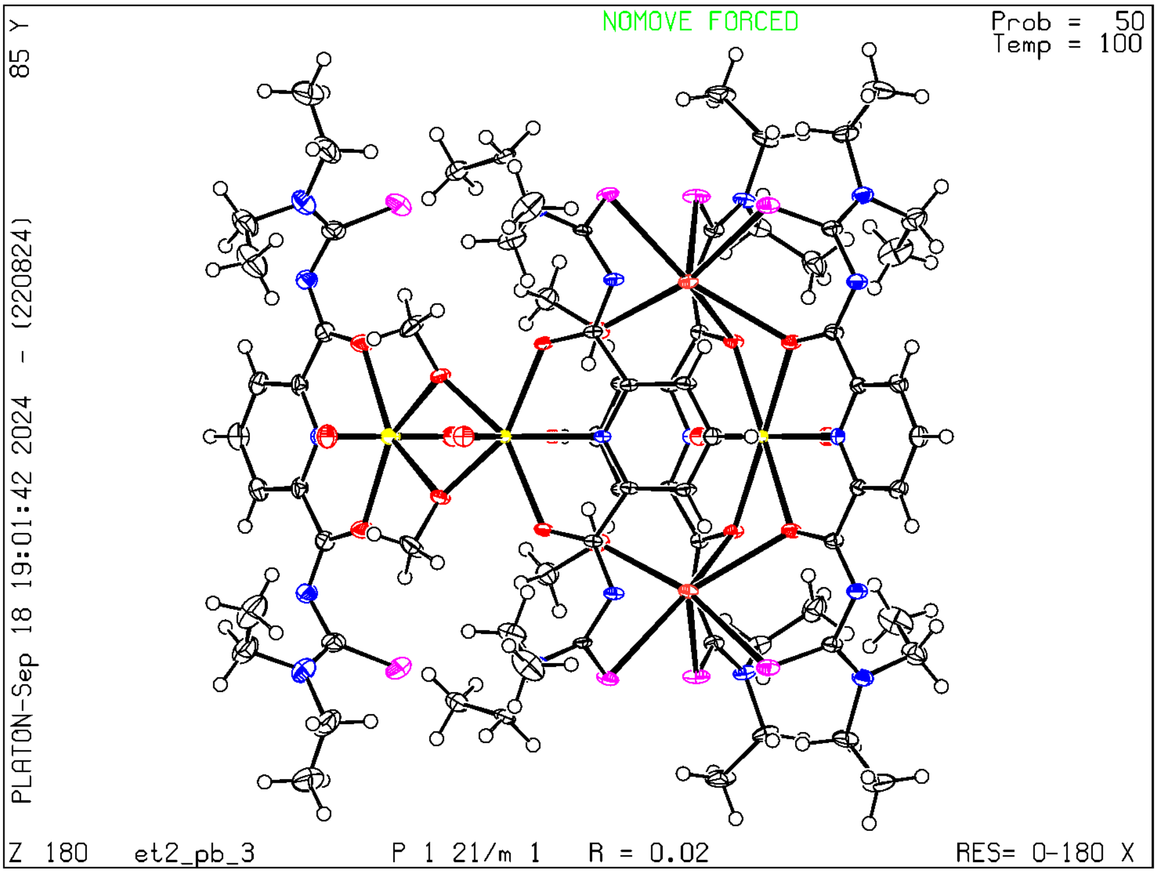

Datablock et2_tetramer_4 - ellipsoid plot

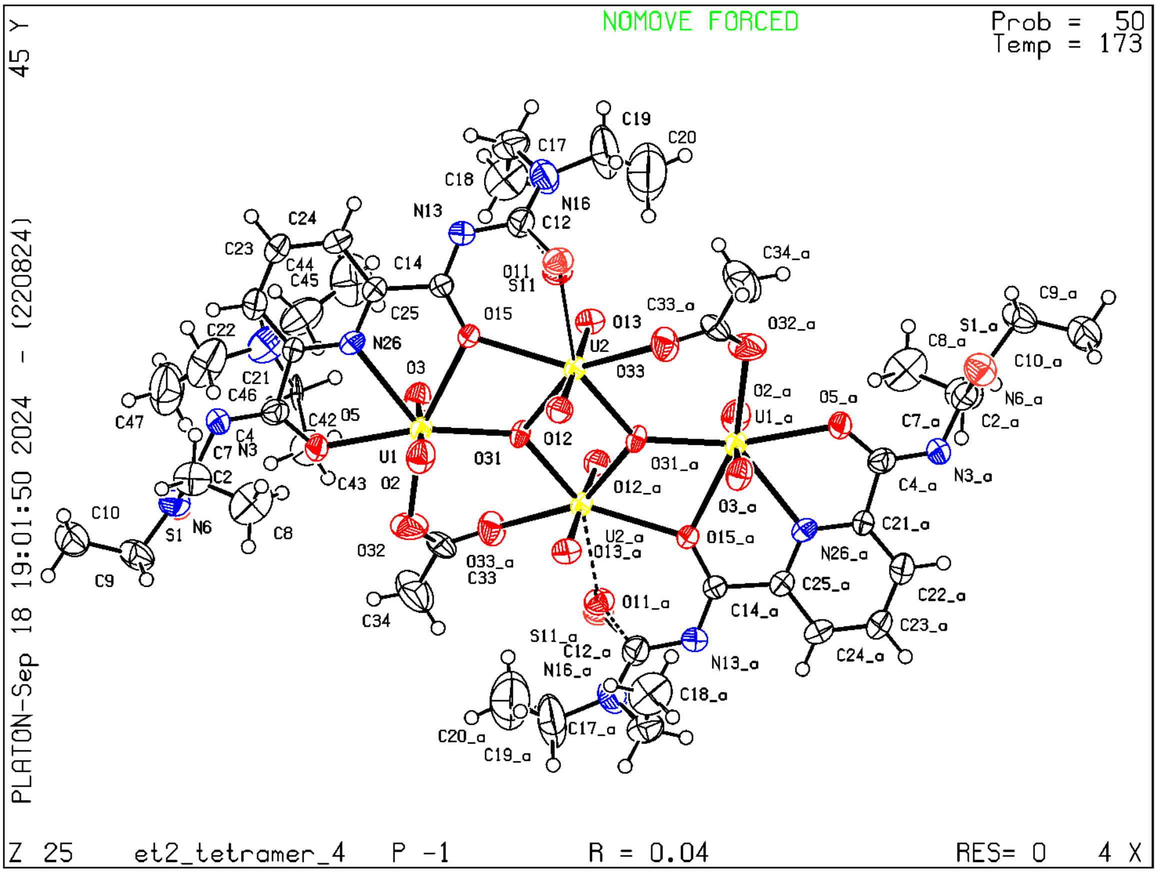

Datablock et2_ni_5a - ellipsoid plot

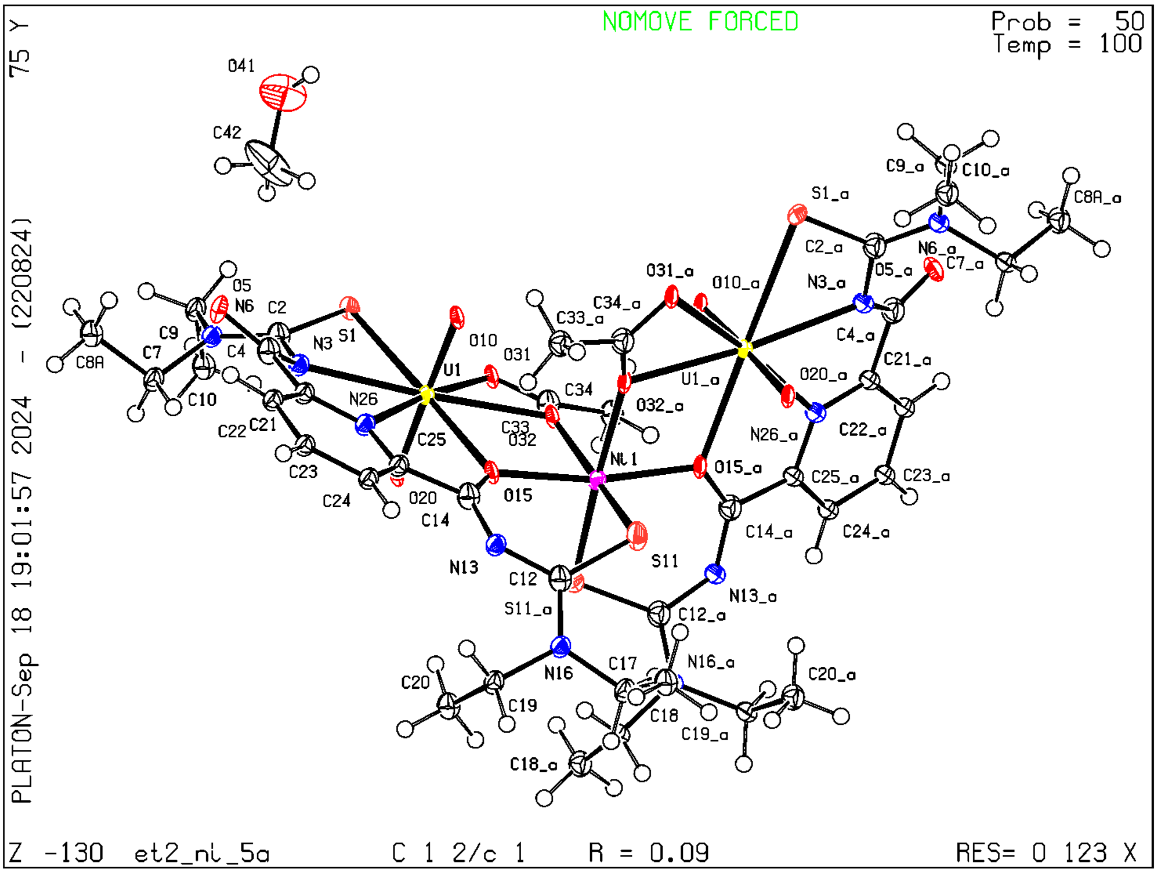

Datablock et2_co_5b - ellipsoid plot

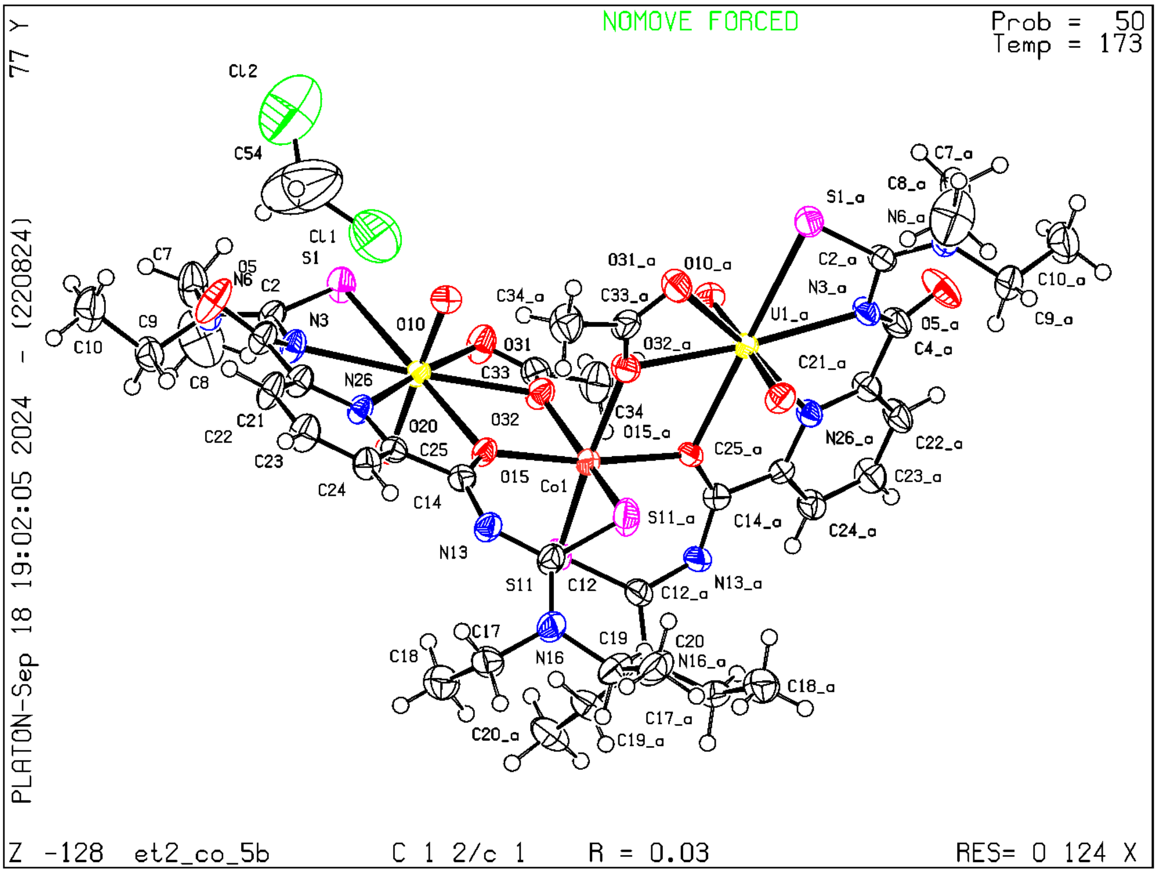

Datablock et2_fe_5c - ellipsoid plot

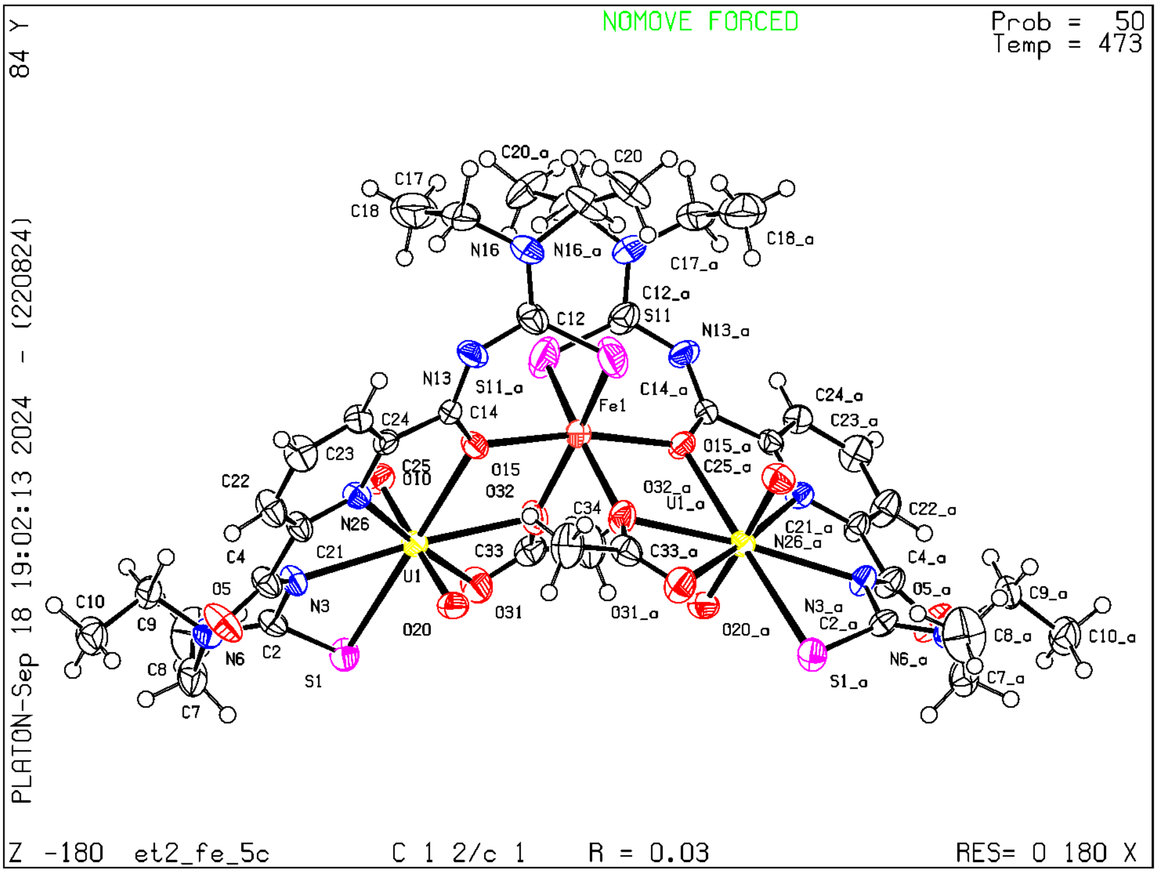

Datablock et2_mn_5d - ellipsoid plot

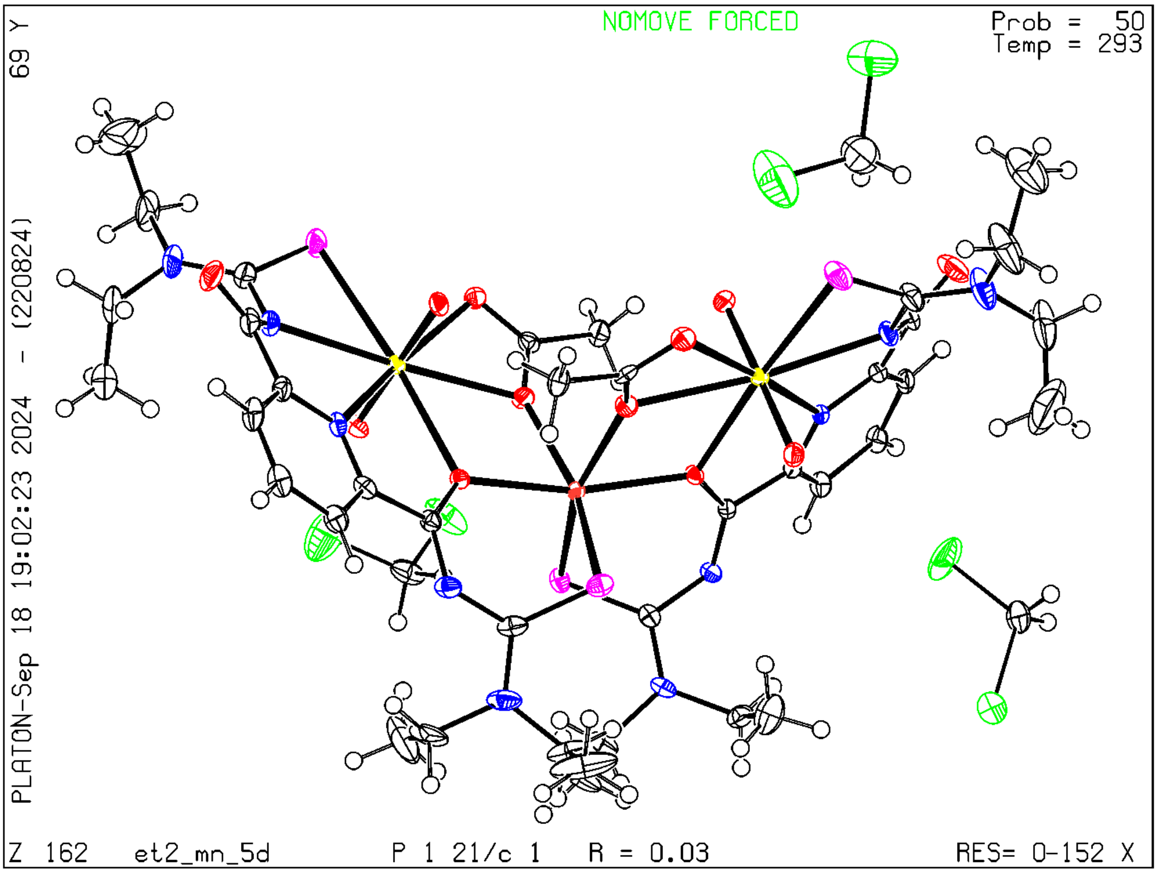

Datablock morph_co_6b - ellipsoid plot

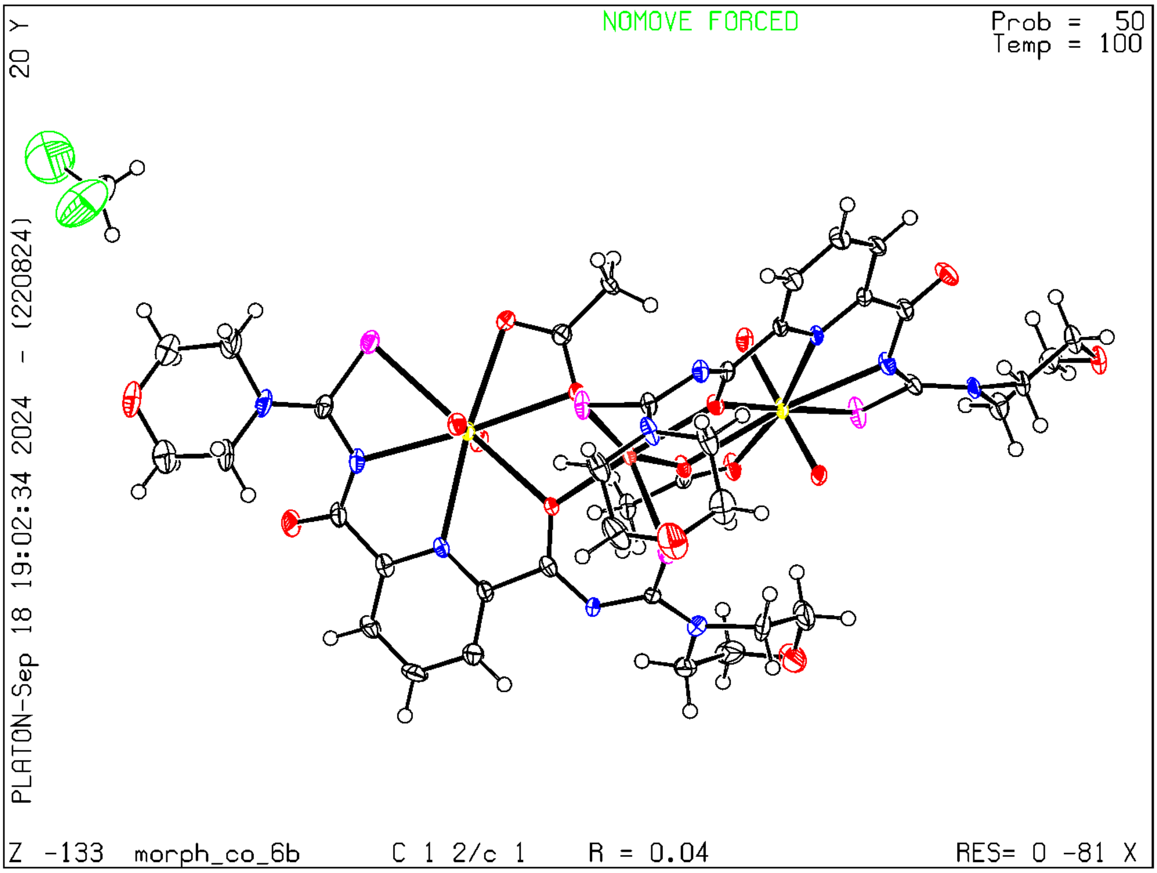

Datablock et2_ni_i_7 - ellipsoid plot

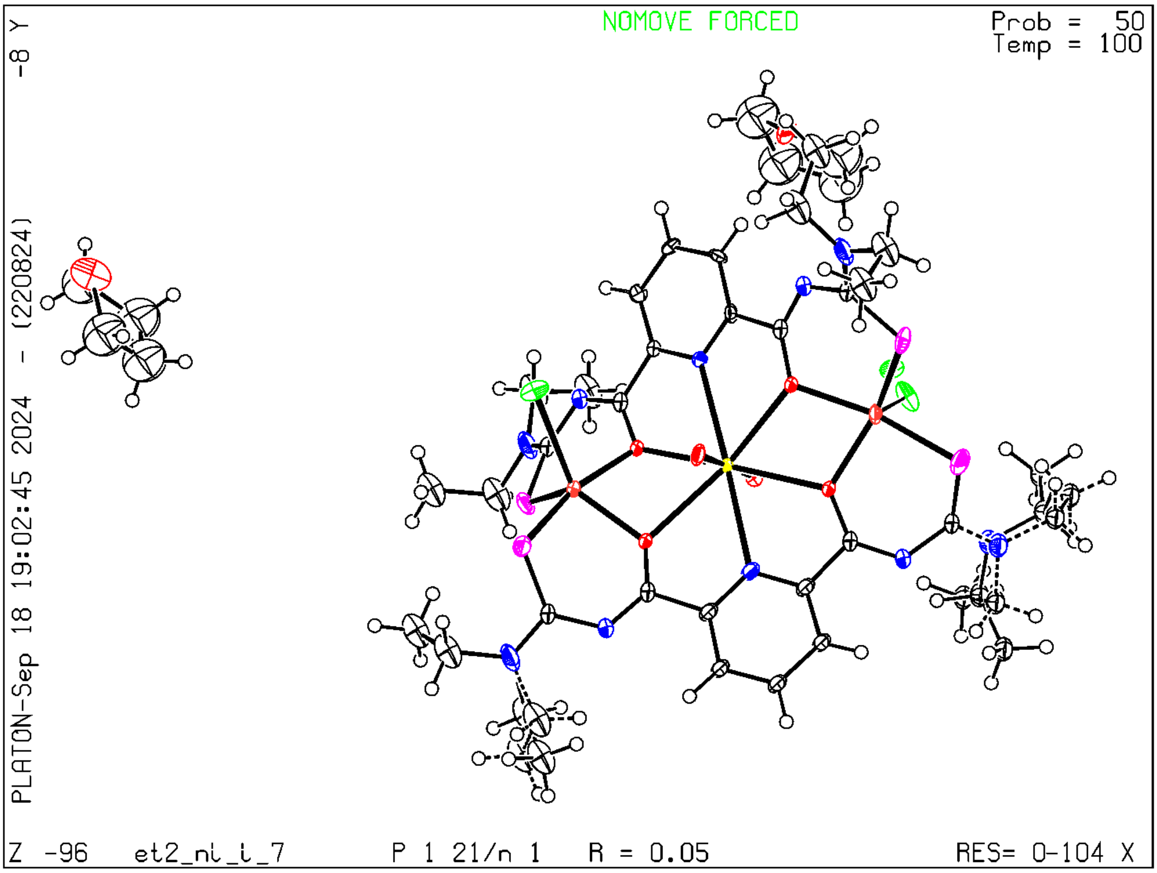

Datablock morph_antianti_8 - ellipsoid plot

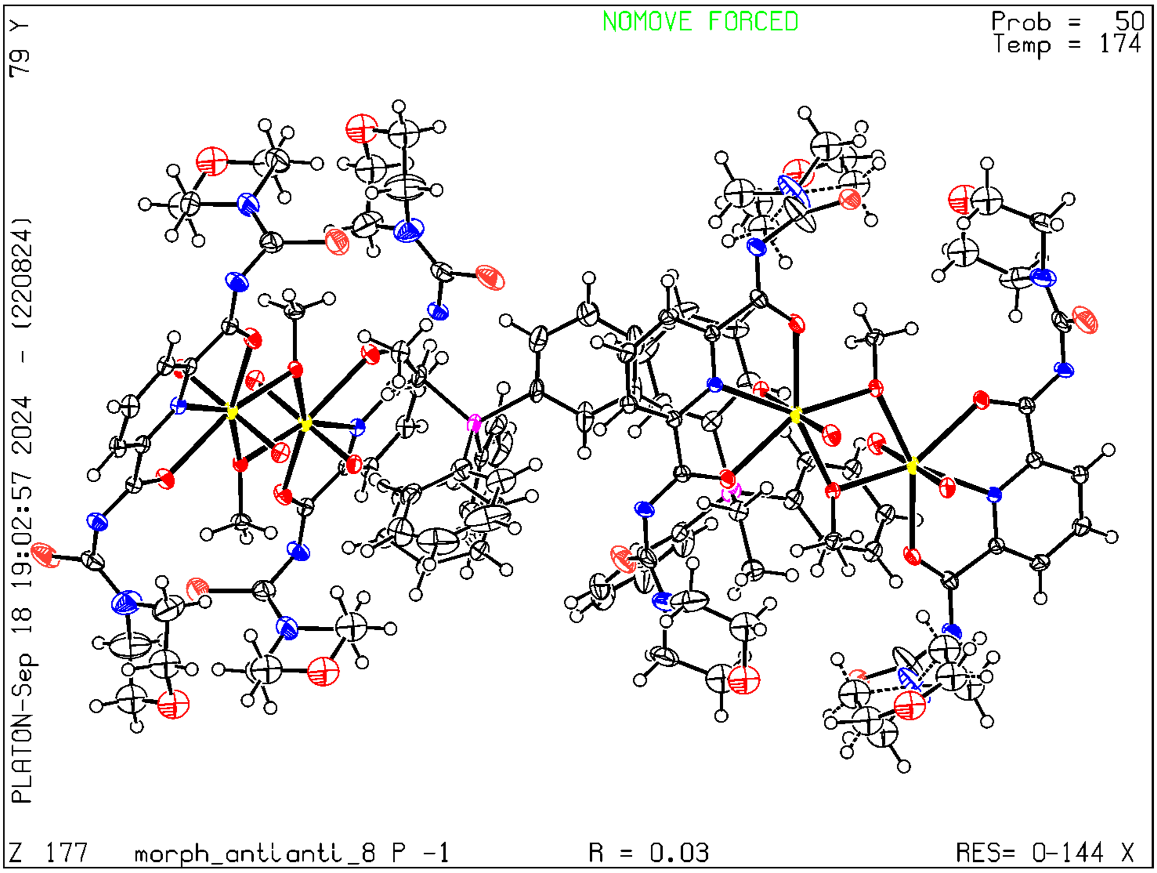

Supplement: Supplementary file 1 [file molecules-29-05001-s001.zip › checkcif_UO2_thiourea.pdf]
