# Supplementary material for: Bimetallic Uranium Complexes with 2,6-Dipicolinoylbis(N,N-Dialkylthioureas)
Source: Molecules. 2024 Oct 22;29(21):5001. doi: 10.3390/molecules29215001 (PMC11548025; doi:10.3390/molecules29215001)
Supplement: Supplementary file 1 [file molecules-29-05001-s001.zip › Supplementary Material_uranium_revised.pdf]

Supplementary Materials to the paper entitled:

Bimetallic uranium complexes with 2,6-dipicolinoylbis(*N*,*N*-dialkylthioureas)

Christelle Njiki Noufele [1], Dennis Schulze [1], Maximilian Roca Jungfer [2], Adelheid Hagenbach [2], and Ulrich Abram [1*]

[1] Institute of Chemistry and Biochemistry, Freie Universität Berlin, Fabeckstr. 34/36, 14195 Berlin, Germany.

[2] Ruprecht-Karls Universität Heidelberg, Im Neuenheimer Feld 271, D-69120 Heidelberg, Germany.

Table of content

1. Crystallographic data 6

Table S1: Crystallographic data and data collection parameters 6

Table S2: Crystallographic data and data collection parameters for inspection only 12

Figure S1. Ellipsoid representation of the structure of $(HNEt_3)_2$*anti,anti*-[**1**], also illustrating the disorderd parts of the molecule. The thermal ellipsoids are set at a 30% probability level. Hydrogen atoms are omitted for clarity. 14

Table S3. Bond lengths (Å) in $(HNEt_3)_2$*anti,anti*-[**1**]. 14

Table S4. Bond angles (°) in $(HNEt_3)_2$*anti,anti*-[**1**]. 14

Figure 2. Ellipsoid representation of the structure of $(EtPPh_3)_2$*syn,anti*-[**1**], also illustrating the disorderd parts of the molecule. The thermal ellipsoids are set at a 30% probability level. Hydrogen atoms are omitted for clarity. 16

Table S5. Bond lengths (Å) $(EtPPh_3)_2$*syn,anti*-[**1**]. 16

Table S6. Bond angles (°) $(EtPPh_3)_2$*syn,anti*-[**1**]. 17

Figure S3. Ellipsoid representation of the structure of $[Pb_2(UO_2)_3(L^{Et2})_3(\mu\text{-}OMe)_2(MeOH)_2]$ (**3**), also illustrating the disorderd parts of the molecule. The thermal ellipsoids are set at a 30% probability level. Hydrogen atoms are omitted for clarity. 20

Table S7. Bond lengths (Å) $[Pb_2(UO_2)_3(L^{Et2})_3(\mu\text{-}OMe)_2(MeOH)_2]$ (**3**). 20

Table S8. Bond angles (°) $[Pb_2(UO_2)_3(L^{Et2})_3(\mu\text{-}OMe)_2(MeOH)_2]$ (**3**). 21

Figure S4. Ellipsoid representation of the structure of $[\{(UO_2)_2(L^{Et2})(\mu_2\text{-}OAc)(\mu_3\text{-}O)\}_2]$ (**4**), also illustrating the partial S/O (90/10) exchange. The thermal ellipsoids are set at a 30% probability level. Hydrogen atoms are omitted for clarity. 24

Table S9. Bond lengths (Å) of $[\{(UO_2)_2(L^{Et2})(\mu_2\text{-}OAc)(\mu_3\text{-}O)\}_2]$ (**4**). 24

Table S10. Bond angles (°) in of $[\{(UO_2)_2(L^{Et2})(\mu_2\text{-}OAc)(\mu_3\text{-}O)\}_2]$ (**4**). 25

Figure S5. Ellipsoid representation of the structure of $[Ni\{UO_2(L^{Et2})(OAc)\}_2]$ (**5a**) x MeOH. The thermal ellipsoids are set at a 30% probability level. Hydrogen atoms are omitted for clarity. 27

Table S11. Bond lengths (Å) of $[Ni\{UO_2(L^{Et2})(OAc)\}_2]$ (**5a**). 27

Table S12. Bond angles (°) in $[Ni\{UO_2(L^{Et2})(OAc)\}_2]$ (**5a**). 29

Figure S6. Ellipsoid representation of the structure of $[Co\{UO_2(L^{Et2})(OAc)\}_2]$ (**5b**) x CH_2Cl_2. The thermal ellipsoids are set at a 30% probability level. Hydrogen atoms are omitted for clarity. 31

Table S13. Bond lengths (Å) of $[Co\{UO_2(L^{Et2})(OAc)\}_2]$ (**5b**). 31

Table S14. Bond angles (°) in $[Co\{UO_2(L^{Et2})(OAc)\}_2]$ (**5b**). 32

Figure S7. Ellipsoid representation of the structure of [Fe{UO_2(L^{Et2})(OAc)}$_2$] (**5c**). The thermal ellipsoids are set at a 30% probability level. Hydrogen atoms are omitted for clarity. 34

Table S15. Bond lengths (Å) of [Fe{UO_2(L^{Et2})(OAc)}$_2$] (**5c**). 34

Table S16. Bond angles (°) in [Fe{UO_2(L^{Et2})(OAc)}$_2$] (**5c**). 34

Figure S8. Ellipsoid representation of the structure of [Mn{UO_2(L^{Et2})(OAc)}$_2$] (**5d**) x CH_2Cl_2. The thermal ellipsoids are set at a 30% probability level. Hydrogen atoms are omitted for clarity. 36

Table S17. Bond lengths (Å) of [Mn{UO_2(L^{Et2})(OAc)}$_2$] (**5d**). 36

Table S18. Bond angles (°) in [Mn{UO_2(L^{Et2})(OAc)}$_2$] (**5d**). 37

Figure S9. Ellipsoid representation of the structure of [Co{UO_2(L^{morph})(OAc)}$_2$] (**6b**) x CH_2Cl_2. The thermal ellipsoids are set at a 30% probability level. Hydrogen atoms are omitted for clarity. 40

Table S19. Bond lengths (Å) of [Co{UO_2(L^{morph})(OAc)}$_2$] (**6b**). 40

Table S20. Bond angles (°) in [Co{UO_2(L^{morph})(OAc)}$_2$] (**6b**). 41

Figure S10. Ellipsoid representation of the structure of [(UO_2)(NiI)$_2$(L^{Et2})$_2$] (**7**) x THF, also illustrating the disorderd parts of the molecule. The thermal ellipsoids are set at a 30% probability level. Hydrogen atoms are omitted for clarity. 44

Table S21. Bond lengths (Å) of [(UO_2)(NiI)$_2$(L^{Et2})$_2$] (**7**). 44

Table S22. Bond angles (°) in [(UO_2)(NiI)$_2$(L^{Et2})$_2$] (**7**). 45

Figure S11. Ellipsoid representation of the structure of ($EtPPh_3$)$_2$*anti,anti*-[{UO_2(L^{morph})(μ-OMe)}$_2$], ($EtPPh_3$)$_2$*anti,anti*-[**8**], also illustrating the disorderd parts of the molecule. The thermal ellipsoids are set at a 30% probability level. Hydrogen atoms are omitted for clarity. 48

Table S23. Bond lengths (Å) of ($EtPPh_3$)$_2$*anti,anti*-[{UO_2(L^{morph})(μ-OMe)}$_2$], ($EtPPh_3$)$_2$*anti,anti*-[**8**]. 48

Table S24. Bond angles (°) in ($EtPPh_3$)$_2$*anti,anti*-[{UO_2(L^{morph})(μ-OMe)}$_2$], ($EtPPh_3$)$_2$*anti,anti*-[**8**]. 50

Figure S12. Representation of the structure of ($HNEt_3$)$_2$*anti,anti*-[{UO_2(L^{morph})(μ-OMe)}$_2$] x MeOH, ($HNEt_3$)$_2$*anti,anti*-[**8**]. The thermal ellipsoids are set at a 30% probability level. Hydrogen atoms are omitted for clarity. 54

Table S25. Bond lengths (Å) of ($HNEt_3$)$_2$*anti,anti*-[{UO_2(L^{morph})(μ-OMe)}$_2$], ($HNEt_3$)$_2$*anti,anti*-[**8**]. 54

Table S26. Bond angles (°) in ($HNEt_3$)$_2$*anti,anti*-[{UO_2(L^{morph})(μ-OMe)}$_2$], ($HNEt_3$)$_2$*anti,anti*-[**8**]. 55

Figure S13. Representation of the structure of [{UO_2(L^{Et2})(μ-OMe)}$_2${Au(PPh_3)}$_2$] (**2**). The thermal ellipsoids are set at a 30% probability level. Hydrogen atoms are omitted for clarity. 56

Table S27. Bond lengths (Å) of [{$UO_2(L^{Et2})(\mu$-OMe)}$_2${Au(PPh_3)}$_2$] (**2**). 56

Table S28. Bond angles (°) in [{$UO_2(L^{Et2})(\mu$-OMe)}$_2${Au(PPh_3)}$_2$] (**2**). 57

Figure S14. Ellipsoid representation of the structure of [Ni{$UO_2(L^{morph})$(OAc)}$_2$] (**6a**), also illustrating the disorderd parts of the molecule. The thermal ellipsoids are set at a 30% probability level. Hydrogen atoms are omitted for clarity. 60

Table S29. Bond lengths (Å) of [Ni{$UO_2(L^{morph})$(OAc)}$_2$] (**6a**). 60

Table S30. Bond angles (°) in [Ni{$UO_2(L^{morph})$(OAc)}$_2$] (**6a**). 61

Figure S15. Ellipsoid representation of the structure of [Zn{$UO_2(L^{morph})$(OAc)}$_2$] (**6e**), also illustrating the disorderd parts of the molecule. The thermal ellipsoids are set at a 30% probability level. Hydrogen atoms are omitted for clarity. 65

Table S31. Bond lengths (Å) of [Zn{$UO_2(L^{morph})$(OAc)}$_2$] (**6e**). 65

Table S32: Bond angles (°) in [Zn{$UO_2(L^{morph})$(OAc)}$_2$] (**6e**). 66

2. Selected Spectroscopic Data 70

Figure S16: ^{1}H NMR spectra of $(HNEt_3)_2$[{$UO_2(L^{Et2})(\mu$-OMe)}$_2$], $(HNEt_3)_2$[**1**]: (a) in $CDCl_3$ and (b) in DMSO. 70

Figure S17: ESI(–) mass spectrum of $(HNEt_3)_2$[{$UO_2(L^{Et2})(\mu$-OMe)}$_2$], $(HNEt_3)_2$[**1**]: (a) overview spectrum, (b) high-mass region with assignment.. 71

Figure S18: ESI(+) mass spectrum of [$Pb_2(UO_2)_3(L^{Et2})_3(\mu$-OMe)$_2$(MeOH)$_2$] (**3**). 72

Figure S19: ESI(+) mass spectrum of [Zn{$UO_2(L^{Et2})$(OAc)}$_2$] (**5e**). 72

Figure S20: ^{1}H NMR spectrum of [Cd{$UO_2(L^{Et2})$(OAc)}$_2$] (**6f**) in $CDCl_3$. 73

Figure S21: ESI(+) mass spectrum of [Cd{$UO_2(L^{Et2})$(OAc)}$_2$] (**6f**). 73

Figure S22: ESI(+) mass spectrum of [$(UO_2)(NiI)_2(L^{Et2})_2$] (**7**): (a) overview spectrum, (b) high-mass region with assignment. 74

3. Computational Chemistry 75

Figure S23: Considered (potential) conformers of H_2L^{Phthal} 75

Table S33: DFT calculations of the different conformations of H_2L^{Phthal}. Optimization level: B3LYP/6-311G. Single-point level: PBE0-GD3BJ/def2-TZVPPD. 75

Figure S24: Considered (potential) conformers of H_2L^{Et2}. Stable conformers are black, while unstable conformers are given in red. Grey conformers have not been considered. 76

Table S34: DFT calculations of the different conformations of H_2L^{morph}. Optimization level: B3LYP/6-311G. Single-point level: PBE0-GD3BJ/def2-TZVPPD. The most stable isomer is bold. 76

Figure S25: Considered (potential) conformers of H_2L^{morph}. Stable conformers are black, while unstable conformers are given in red. Grey conformers have not been considered. 77

Table S35: DFT calculations of the different conformations of H_2L^{morph}. Optimization level: B3LYP/6-311G. Single-point level: PBE0-GD3BJ/def2-TZVPPD. The most stable isomer is bold. 77

Table S36: DFT calculations of the different conformations of H_2L^{morph}. Level: B3LYP/LANL2DZ(uranium)+6-311++G**(others). The most stable isomer is bold. 79

Table S37: DFT calculations of the different conformations of H_2L^{morph}. Optimization level: B3LYP/LANL2DZ(uranium)+6-311++G**(others). Single-point level: PBE0-GD3BJ/StuttgartRLC(uranium)+def2-TZVPPD(others). The most stable isomer is bold. 79

4. References 79

1. Crystallographic data

Table S1: Crystallographic data and data collection parameters.

	$(HNEt_3)_2$ *anti,anti*-[{$UO_2(L^{Et2})(\mu$-OMe)}$_2$] (HNEt$_3$)$_2$*anti,anti*-[**1**]	$(EtPPh_3)_2$*syn,anti*-[{$UO_2(L^{Et2})(\mu$-OMe)}$_2$] (EtPPh$_3$)$_2$*syn,anti*-[**1**]
Empirical formula	$C_{48}H_{84}N_{12}O_{10}S_4U_2$	$C_{76}H_{92}N_{10}O_{10}S_4U_2P_2$
Formula weight	1593.57	1971.83
Temperature/K	293(2)	100
Crystal system	Monoclinic	Monoclinic
Space group	C2/c	$P2_1/n$
a/Å	33.452(2)	14.2770(10
b/Å	9.702(2)	25.6320(10)
c/Å	22.278(2)	22.7380(10)
α/°	90	90
β/°	119.96(2)	97.39
γ/°	90	90
Volume/Å^3	6264.2(18)	8251.8(8)
Z	4	4
ρ_{calc} / gcm^{-3}	1.690	1.587
μ / mm^{-1}	5.357	4.120
F(000)	3136.0	3904.0
Crystal size / mm^3	0.22 × 0.22 × 0.04	0.42 × 0.11 × 0.08
Radiation	MoKα (λ = 0.71073)	MoKα (λ = 0.71073)
2Θ range for data collection/°	9.406 to 48.992	4.506 to 52.892
Index ranges	$-38 \le h \le 38$, $-11 \le k \le 10$, $-25 \le l \le 24$	$17 \le h \le 17$, $-32 \le k \le 32$, $-28 \le l \le 28$
Reflections collected	22207	187388
Independent reflections	5164 [R_{int} = 0.0659, R_{sigma} = 0.0543]	6073 [R_{int} = 0.0505, R_{sigma} = 0.0350]
Data/restraints/parameters	5164/311/320	16919/0/919
Goodness-of-fit on F^2	1.051	1.087
Final R indexes [I>=2σ (I)]	R_1 = 0.0496, wR_2 = 0.1146	R_1 = 0.0314, wR_2 = 0.0657
Final R indexes [all data]	R_1 = 0.0760, wR_2 = 0.1241	R_1 = 0.0447, wR_2 = 0.0739
Largest diff. peak/hole / e Å^{-3}	2.19/-1.90	1.71/-1.39
Diffractometer	STOE IPDS T2	Bruker CCD
Remarks	-	-
CCDC access code	2381544	2381545

Table S1: Crystallographic data and data collection parameters (continued)

	$[Pb_2(UO_2)_3(L^{Et2})_3(\mu\text{-}OMe)_2(MeOH)_2]$ (**3**)	$[\{(UO_2)_2(L^{Et2})(\mu_2\text{-}OAc)(\mu_3\text{-}O)\}_2]$ ($\{HNEt_3\}_2$[**4**]))
Empirical formula	$C_{78}H_{130}N_{20}O_{24}Pb_2S_8U_3$	$C_{49}H_{82}N_{12}O_{18.25}S_{3.75}U_4$
Formula weight	3116.96	2203.61
Temperature/K	100(2)	173.15
Crystal system	Monoclinic	Triclinic
Space group	$P2_1/m$	P-1
a/Å	10.686(2)	11.6510(10)
b/Å	29.664(6)	11.7360(10)
c/Å	17.074(3)	3.8890(10)
α/°	90	112.570(10)
β/°	100.81(3)	100.570(10)
γ/°	90	100.570(10)
Volume/Å^3	5316.2(18)	1709.5(3)
Z	2	1
ρ_{calc} / gcm^{-3}	1.947	2.140
μ / mm^{-1}	7.942	9.631
F(000)	2996.0	1034.0
Crystal size / mm^3	0.07 × 0.03 × 0.02	0.21 × 0.13 × 0.08
Radiation	MoKα (λ = 0.71073)	MoKα (λ = 0.71073)
2Θ range for data collection/°	4.754 to 54.404	6.574 to 52
Index ranges	-13 ≤ h ≤ 12, -33 ≤ k ≤ 38, -21 ≤ l ≤ 21	-14 ≤ h ≤ 14, -14 ≤ k ≤ 14, -17 ≤ l ≤ 17
Reflections collected	46287	14795
Independent reflections	11816 [R_{int} = 0.0227, R_{sigma} = 0.0207]	6659 [R_{int} = 0.0890, R_{sigma} = 0.0689]
Data/restraints/parameters	11816/0/592	6659/44/401
Goodness-of-fit on F^2	1.079	1.045
Final R indexes [I>=2σ (I)]	R_1 = 0.0234, wR_2 = 0.0486	R_1 = 0.0445, wR_2 = 0.1082
Final R indexes [all data]	R_1 = 0.0286, wR_2 = 0.0508	R_1 = 0.0555, wR_2 = 0.1132
Largest diff. peak/hole / e Å^{-3}	1.59/-1.61	2.20/-2.43
Diffractometer	Bruker CCD	STOE IPDS T2
Remarks	A solvent mask was calculated and 200 electrons were found in a volume of 690 A^3 in 1 void per unit cell. This is consistent with the presence of 6 methanol molecules per formula unit which account for 216 electrons per unit cell.	Due to partial hydrolysis of the ligand during the synthesis of the uranium complex, a sulfur/oxygen disorder of 90/10 per cent is observed for the two sulfur atoms coordinated to uranium. This has been considered during the refinement.
CCDC access code	2381546	2381547

Table S1: Crystallographic data and data collection parameters (continued)

	$[Ni\{UO_2(L^{Et2})(OAc)\}_2]$ (**5a**)	$[Co\{UO_2(L^{Et2})(OAc)\}_2]$ (**5b**)
Empirical formula	$C_{40}H_{60}N_{10}NiO_{14}S_4U_2$	$U_2CoN_{10}S_4O_{12}C_{40}H_{56}Cl_4$
Formula weight	1567.99	1673.97
Temperature/K	100	173.15
Crystal system	Monoclinic	Monoclinic
Space group	C2/c	C2/c
a/Å	18.684(2)	18.8010(10)
b/Å	10.5544(11)	10.6990(10)
c/Å	27.845(3)	28.3810(10)
α/°	90	90
β/°	92.732(4)	92.770(4)
γ/°	90	90
Volume/Å^3	5484.7(10)	5702.2(6)
Z	4	4
ρ_{calc} / gcm^{-3}	1.899	1.950
μ / mm^{-1}	6.450	6.351
F(000)	3032.0	3220.0
Crystal size / mm^3	0.17 × 0.15 × 0.10	0.21 × 0.14 × 0.06
Radiation	MoKα (λ = 0.71073)	MoKα (λ = 0.71073)
2Θ range for data collection/°	4.434 to 49.5	7.034 to 49.994
Index ranges	$-22 \le h \le 22$, $-12 \le k \le 10$, $-32 \le l \le 32$	$-22 \le h \le 22$, $-12 \le k \le 12$, $-33 \le l \le 33$
Reflections collected	35671	19897
Independent reflections	4678 [R_{int} = 0.1227, R_{sigma} = 0.0639]	4980 [R_{int} = 0.0360, R_{sigma} = 0.0272]
Data/restraints/parameters	4678/0/179	4980/0/331
Goodness-of-fit on F^2	1.183	1.047
Final R indexes [I>=2σ (I)]	R_1 = 0.0893, wR_2 = 0.1983	R_1 = 0.0281, wR_2 = 0.0631
Final R indexes [all data]	R_1 = 0.1061, wR_2 = 0.2076	R_1 = 0.0359, wR_2 = 0.0653
Largest diff. peak/hole / e Å^{-3}	4.02/-5.26	0.68/-1.26
Diffractometer	Bruker CCD	STOE IPDS
Remarks	-	-
CCDC access code	2381548	2381549

Table S1: Crystallographic data and data collection parameters (continued)

	$[Fe\{UO_2(L^{Et2})(OAc)\}_2]$ (**5c**)	$[Mn\{UO_2(L^{Et2})(OAc)\}_2]$ (**5d**)
Empirical formula	$C_{38}H_{60}FeN_{10}O_{16}S_4U_2$	$C_{40}H_{56}Cl_4MnN_{10}O_{12}S_4U_2$
Formula weight	1573.11	1669.98
Temperature/K	200(2)	293.15
Crystal system	Monoclinic	Monoclinic
Space group	C2/c	$P2_1/c$
a/Å	18.774(2)	9.1432(6)
b/Å	10.6320(10)	20.9021(11)
c/Å	28.219(3)	31.498(2)
α/°	90	90
β/°	92.140(10)	91.747(2)
γ/°	90	90
Volume/Å^3	5628.7(10)	6016.9(6)
Z	4	4
ρ_{calc} / gcm^{-3}	1.856	1.844
μ / mm^{-1}	6.212	5.953
F(000)	3040.0	3212.0
Crystal size / mm^3	0.12 × 0.12 × 0.02	0.12 × 0.09 × 0.08
Radiation	MoKα (λ = 0.71073)	MoKα (λ = 0.71073)
2Θ range for data collection/°	7.1 to 51.996	4.342 to 54.414
Index ranges	$-23 \le h \le 19$, $-13 \le k \le 13$, $-34 \le l \le 34$	- $-11 \le h \le 11$, $-26 \le k \le 26$, $-40 \le l \le 40$
Reflections collected	16292	196098
Independent reflections	5516 [R_{int} = 0.0490, R_{sigma} = 0.0524]	13337 [R_{int} = 0.0431, R_{sigma} = 0.0185]
Data/restraints/parameters	5516/0/303	13337/0/695
Goodness-of-fit on F^2	0.922	1.145
Final R indexes [I>=2σ (I)]	R_1 = 0.0315, wR_2 = 0.0587	R_1 = 0.0313, wR_2 = 0.0733
Final R indexes [all data]	R_1 = 0.0478, wR_2 = 0.0617	R_1 = 0.0348, wR_2 = 0.0748
Largest diff. peak/hole / e Å^{-3}	0.68/-1.09	2.44/-1.70
Diffractometer	STOE IPDS	STOE IPDS
Remarks	A solvent mask was calculated and 168 electrons were found in a volume of 904 A^3 in 1 void per unit cell. This is consistent with the presence of 4 H_2O per formula unit which account for 160 electrons per unit cell.	-
CCDC access code	2381550	2381551

Table S1: Crystallographic data and data collection parameters (continued)

	$[Co\{UO_2(L^{morph})(OAc)\}_2]$ x 0.5 CH_2Cl_2 (**6b**)	$[(UO_2)(NiI)_2(L^{Et2})_2]$ (**7**)
Empirical formula	$C_{40}H_{51}ClCoN_{10}O_{17.5}S_4U_2$	$C_{42}H_{62}I_2N_{10}Ni_2O_8S_4U$
Formula weight	1650.58	1572.56
Temperature/K	100.03	100.00
Crystal system	Monoclinic	Monoclinic
Space group	C2/c	$P2_1/n$
a/Å	44.055(5)	21.79(3)
b/Å	10.7980(12)	9.958(14)
c/Å	29.051(3)	25.88(3
α/°	90	90
β/°	128.050(2)	101.36(3)
γ/°	90	90
Volume/Å^3	10882.7(19)	5504(13)
Z	8	4
ρ_{calc} / gcm^{-3}	2.015	1.898
μ / mm^{-1}	6.518	4.942
F(000)	6344.0	3064.0
Crystal size / mm^3	0.16 × 0.07 × 0.05	0.27 × 0.06 × 0.01
Radiation	MoKα (λ = 0.71073)	MoKα (λ = 0.71073)
2Θ range for data collection/°	4.622 to 53.084	4.514 to 48.996
Index ranges	-55 ≤ h ≤ 54, -13 ≤ k ≤ 13, -33 ≤ l ≤ 36	-25 ≤ h ≤ 25, -11 ≤ k ≤ 11, -30 ≤ l ≤ 30
Reflections collected	84818	115543
Independent reflections	11239 [R_{int} = 0.0564, R_{sigma} = 0.0320]	9123 [R_{int} = 0.0590, R_{sigma} = 0.0229]
Data/restraints/parameters	11239/0/668	9123/10/407
Goodness-of-fit on F^2	1.139	1.066
Final R indexes [I>=2σ (I)]	R_1 = 0.0387, wR_2 = 0.0682	R_1 = 0.0509, wR_2 = 0.1005
Final R indexes [all data]	R_1 = 0.0480, wR_2 = 0.0706	R_1 = 0.0589, wR_2 = 0.1048
Largest diff. peak/hole / e Å^{-3}	1.70/-1.78	2.43/-2.17
Diffractometer	Bruker CCD	Bruker CCD
Remarks	A solvent mask was calculated and 220 electrons were found in a volume of 800 A^3 in 2 voids per unit cell. This is consistent with the presence of 4 molecules of MeOH per formula unit which account for 216 electrons per unit cell.	-
CCDC access code	23815452	2381553

Table S1: Crystallographic data and data collection parameters (continued)

	$(EtPPh_3)_2$*anti,anti*-[{UO_2(Lmorph)(μ-OMe)}$_2$] $(EtPPh_3)_2$*anti,anti*-[**8**]
Empirical formula	$C_{80}H_{98}N_{10}O_{18}P_2S_4U_2$
Formula weight	2153.92
Temperature/K	174.10
Crystal system	Triclinic
Space group	P-1
a/Å	12.009(6)
b/Å	14.640(9)
c/Å	25.11(2)
α/°	91.38(2)
β/°	90.96(2)
γ/°	94.320(10)
Volume/Å^3	4400(5)
Z	2
ρ_{calc} / gcm^{-3}	1.626
μ / mm^{-1}	3.878
F(000)	2140.0
Crystal size / mm^3	0.23 × 0.19 × 0.13
Radiation	MoKα (λ = 0.71073)
2Θ range for data collection/°	4.334 to 48.992
Index ranges	-13 ≤ h ≤ 14, -17 ≤ k ≤ 17, -29 ≤ l ≤ 29
Reflections collected	81346
Independent reflections	14564 [R_{int} = 0.0311, R_{sigma} = 0.0206]
Data/restraints/parameters	14564/38/883
Goodness-of-fit on F^2	1.041
Final R indexes [I>=2σ (I)]	R_1 = 0.0313, wR_2 = 0.0692
Final R indexes [all data]	R_1 = 0.0327, wR_2 = 0.0700
Largest diff. peak/hole / e Å^{-3}	1.67/-1.90
Diffractometer	Bruker CCD
Remarks	A solvent mask was calculated and 154 electrons were found in a volume of 725 A^3 in 2 voids per unit cell. This is consistent with the presence of 4 MeOH molecules per formula unit which account for 144 electrons per unit cell.
CCDC access code	2381563

Table S2: Crystallographic data and data collection parameters for inspection only.

	$(HNEt_3)_2$*anti,anti*-[{UO_2(Lmorph)(μ-OMe)}$_2$]<br>$(HNEt_3)_2$*anti,anti*-[8]<br>**For inspection only**	[{UO_2(L^{Et2})(μ-OMe)}$_2${Au(PPh_3)}$_2$] (**2**)<br>**For inspection only**
Empirical formula	$C_{50}H_{84}N_{12}O_{16}S_4U_2$	$C_{72}H_{82}Au_2N_{10}O_{10}P_2S_4U_2$
Formula weight	856.79	2307.65
Temperature/K	293(2)	200(2) K
Crystal system	Triclinic	Triclinic
Space group	P-1	P-1
a/Å	9.125(5)	9.9110(8)
b/Å	12.577(5)	15.6270(15)
c/Å	15.095(5)	31.476(2)
α/°	99.167(5)	100.980(7)
β/°	106.726(5)	97.040(6)
γ/°	97.276(5))	96.710(7)
Volume/Å^3	1610.4(12)	4700.2(7)
Z	1	2
ρ_{calc} / gcm^{-3}	1.767	1.631
μ / mm^{-1}	5.223	6.721
F(000)	844.0	2200
Crystal size / mm^3	0.28 × 0.17 × 0.02	0.13 x 0.22 x 0.13
Radiation	MoKα (λ = 0.71073)	MoKα (λ = 0.71073)
2Θ range for data collection/°	4.74 to 48.998	3.18 to 25.00
Index ranges	$-10 \le h \le 9$, $-14 \le k \le 14$, $-17 \le l \le 17$	-11<=h<=11, -18<=k<=18, -37<=l<=37
Reflections collected	10715	36196
Independent reflections	5264 [R_{int} = 0.0932, R_{sigma} = 0.0869]	16463 [R(int) = 0.1645]
Data/restraints/parameters	5264/2/328	16463 / 70 / 919
Goodness-of-fit on F^2	1.402	0.895
Final R indexes [I>=2σ (I)]	R_1 = 0.1273, wR_2 = 0.3293	R_1 = 0.0721, wR2 = 0.1726
Final R indexes [all data]	R_1 = 0.1429, wR_2 = 0.3390	R_1 = 0.1573, wR2 = 0.2059
Largest diff. peak/hole / e Å^{-3}	13.28/-2.46	1.281/-1.672
Diffractometer	STOE IPDS	STOE IPDS
Remarks		
CCDC access code	Not deposited because of the low quality of the data set	Not deposited because of the low quality of the data set

Table S2: Crystallographic data and data collection parameters for inspection only (continued)

	$[Ni\{UO_2(L^{morph})(OAc)\}_2]$ x 1.5 CH_2Cl_2 (**6a**) **For inspection only**	$[Zn\{UO_2(L^{morph})(OAc)\}_2]$ x 1.5 CH_2Cl_2 (**6e**) **For inspection only**
Empirical formula	$C_{39.5}H_{45}Cl_3N_{10}NiO_{16}S_4U_2$	$C_{39.5}H_3Cl_3N_{10}O_{16}S_4U_2Zn$
Formula weight	1685.21	1649.54
Temperature/K	100.04	100(2) K
Crystal system	Monoclinic	Monoclinic
Space group	C2/c	C2/c
a/Å	44.570(4)	44.414(4)
b/Å	10.7330(10)	10.7390(8)
c/Å	29.030(2)	29.198(2)
α/°	90	90
β/°	128.850(2)	128.640(2)
γ/°	90	90
Volume/Å^3	10815.1(16)	10877.6(15)
Z	8	8
ρ_{calc} / gcm^{-3}	2.070	2.015
μ / mm^{-1}	6.697	6.751
F(000)	6456.0	6136.0
Crystal size / mm^3	0.28 × 0.17 × 0.02	0.3 × 0.06 × 0.03
Radiation	MoKα (λ = 0.71073)	MoKα (λ = 0.71073)
2Θ range for data collection/°	4.656 to 54.472	4.644 to 54.4
Index ranges	-57 ≤ h ≤ 57, -13 ≤ k ≤ 13, -35 ≤ l ≤ 37	-56 ≤ h ≤ 56, -13 ≤ k ≤ 13, -37 ≤ l ≤ 37
Reflections collected	141453	137912
Independent reflections	11992 [R_{int} = 0.0469, R_{sigma} = 0.0233]	12051 [R_{int} = 0.0618, R_{sigma} = 0.0300]
Data/restraints/parameters	11992/55/404	12051/31/592
Goodness-of-fit on F^2	11992/55/404	1.070
Final R indexes [I>=2σ (I)]	R_1 = 0.0715, wR_2 = 0.1555	R_1 = 0.0535, wR_2 = 0.1066
Final R indexes [all data]	R_1 = 0.0779, wR_2 = 0.1588	R_1 = 0.0628, wR_2 = 0.1112
Largest diff. peak/hole / e Å^{-3}	4.99/-4.52	5.11/-4.02
Diffractometer	Bruker CCD	Bruker CCD
Remarks	A solvent mask was calculated and 464 electrons were found in a volume of 1552 A^3 in 2 voids per unit cell. This is consistent with the presence of 1.5 CH_2Cl_2 per formula unit which account for 504 electrons per unit cell.	A solvent mask was calculated and 480 electrons were found in a volume of 1432 A^3 in 2 voids per unit cell. This is consistent with the presence of 1.5 CH_2Cl_2 per formula unit which account for 504 electrons per unit cell.
CCDC access code	Not deposited because of the low quality of the data set	Not deposited because of the low quality of the data set

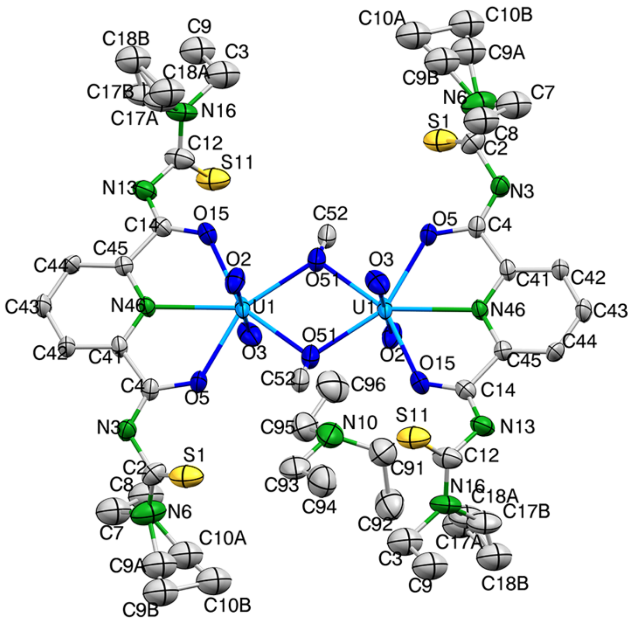

Figure S1. Ellipsoid representation of the structure of $(HNEt_3)_2$*anti,anti*-[**1**], also illustrating the disorderd parts of the molecule. The thermal ellipsoids are set at a 30% probability level. Hydrogen atoms are omitted for clarity.

Table S3. Bond lengths (Å) in $(HNEt_3)_2$*anti,anti*-[**1**].

U1	U1[1]	3.7825(7)	C14	C45	1.505(13)	N6	C9A	1.48(5)
U1	O2	1.768(7)	N16	C17A	1.45(6)	N6	C9B	1.49(5)
U1	O3	1.762(7)	N16	C17B	1.46(4)	C7	C8	1.46(2)
U1	O5	2.357(7)	N16	C3	1.54(2)	S11	C12	1.678(13)
U1	O15	2.348(8)	C41	C42	1.393(12)	C12	N13	1.400(14)
U1	N46	2.518(6)	C41	N46	1.341(11)	C12	N16	1.311(15)
U1	O51	2.357(7)	C42	C43	1.372(14)	N13	C14	1.292(14)
U1	O51[1]	2.377(8)	C43	C44	1.393(15)	C14	O15	1.280(12)
S1	C2	1.672(12)	C44	C45	1.389(12)	C3	C9	1.41(2)
C2	N3	1.420(14)	C45	N46	1.365(12)	N10	C93	1.55(2)
C2	N6	1.316(16)	C9A	C10A	1.52(7)	N10	C91	1.469(19)
N3	C4	1.276(12)	C9B	C10B	1.41(6)	N10	C95	1.57(2)
C4	O5	1.289(10)	C17A	C18A	1.29(7)	C96	C95	1.47(3)
C4	C41	1.482(13)	C18B	C17B	1.47(5)	C92	C91	1.53(2)
N6	C7	1.54(2)	O51	C52	1.664(13)	C93	C94	1.49(3)

[1]1/2-X,3/2-Y,-Z

Table S4. Bond angles (°) in $(HNEt_3)_2$*anti,anti*-[**1**].

O2	U1	U1[1]	89.1(2)	C8	C7	N6	105.7(15)
O2	U1	O5	92.2(3)	N13	C12	S11	120.5(9)
O2	U1	O15	88.8(3)	N16	C12	S11	123.6(10)
O2	U1	N46	88.9(3)	N16	C12	N13	115.8(11)
O2	U1	O51[1]	88.0(3)	C14	N13	C12	118.7(10)

O2	U1	O51	90.6(3)	N13	C14	C45	117.6(9)
O3	U1	U1[1]	91.0(3)	O15	C14	N13	127.3(9)
O3	U1	O2	179.5(4)	O15	C14	C45	115.1(9)
O3	U1	O5	88.2(3)	C14	O15	U1	127.6(6)
O3	U1	O15	90.7(4)	C12	N16	C17A	120(3)
O3	U1	N46	91.0(3)	C12	N16	C17B	120(2)
O3	U1	O51[1]	92.4(4)	C12	N16	C3	120.9(12)
O3	U1	O51	89.2(3)	C17A	N16	C3	115(3)
O5	U1	U1[1]	116.21(14)	C17B	N16	C3	119(2)
O5	U1	N46	63.3(2)	C42	C41	C4	125.2(8)
O5	U1	O51[1]	79.5(2)	N46	C41	C4	113.6(7)
O15	U1	U1[1]	116.65(15)	N46	C41	C42	121.0(9)
O15	U1	O5	127.1(2)	C43	C42	C41	119.2(9)
O15	U1	N46	63.9(2)	C42	C43	C44	120.6(8)
O15	U1	O51	79.6(3)	C45	C44	C43	117.8(9)
O15	U1	O51[1]	153.3(2)	C44	C45	C14	125.9(9)
N46	U1	U1[1]	177.89(17)	N46	C45	C14	112.3(8)
O51[1]	U1	U1[1]	36.78(19)	N46	C45	C44	121.5(8)
O51	U1	U1[1]	37.2(2)	C41	N46	U1	120.3(6)
O51	U1	O5	153.2(2)	C41	N46	C45	119.9(7)
O51[1]	U1	N46	142.5(3)	C45	N46	U1	119.8(5)
O51	U1	N46	143.4(3)	N6	C9A	C10A	113(4)
O51	U1	O51[1]	73.9(3)	C10B	C9B	N6	104(4)
N3	C2	S1	122.0(9)	C18A	C17A	N16	116(4)
N6	C2	S1	124.3(10)	N16	C17B	C18B	107(3)
N6	C2	N3	113.7(11)	U1	O51	U1[1]	106.1(3)
C4	N3	C2	121.0(8)	C52	O51	U1[1]	114.7(5)
N3	C4	O5	127.5(9)	C52	O51	U1	118.2(6)
N3	C4	C41	117.6(8)	C9	C3	N16	102.6(15)
O5	C4	C41	114.9(8)	C93	N10	C95	110.1(12)
C4	O5	U1	127.8(6)	C91	N10	C93	116.8(14)
C2	N6	C7	123.3(12)	C91	N10	C95	110.3(14)
C2	N6	C9A	122(2)	C94	C93	N10	110.0(15)
C2	N6	C9B	119.4(19)	N10	C91	C92	115.5(13)
C9A	N6	C7	111(2)	C96	C95	N10	108.7(13)
C9B	N6	C7	115.6(19)				

[1]1/2-x,3/2-y,-z

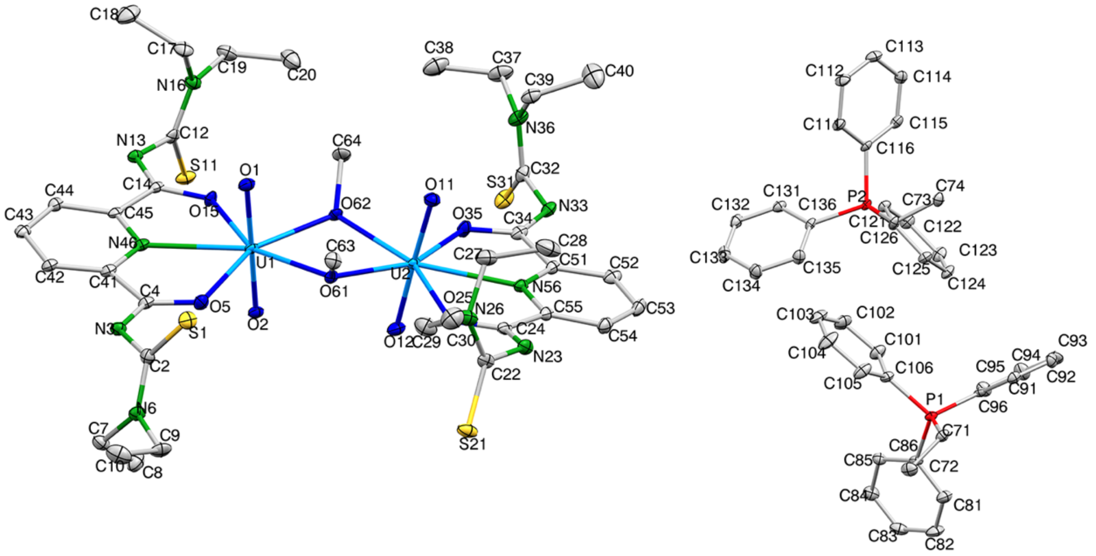

Figure 2. Ellipsoid representation of the structure of (EtPPh$_3$)$_2$*syn,anti*-[**1**], also illustrating the disorderd parts of the molecule. The thermal ellipsoids are set at a 30% probability level. Hydrogen atoms are omitted for clarity.

Table S5. Bond lengths (Å) (EtPPh$_3$)$_2$*syn,anti*-[**1**].

U1	U2	3.7434(3)	N26	C27	1.474(6)
U1	O61	2.332(3)	C14	C45	1.497(6)
U1	O62	2.350(3)	C45	C44	1.391(6)
U1	O1	1.782(3)	C34	C51	1.500(6)
U1	O5	2.359(3)	C42	C41	1.359(6)
U1	O2	1.786(3)	C42	C43	1.383(6)
U1	O15	2.362(3)	C96	C91	1.386(6)
U1	N46	2.537(4)	C96	C95	1.396(6)
U2	O61	2.351(3)	C55	C24	1.491(7)
U2	O62	2.341(3)	C55	C54	1.382(6)
U2	O25	2.357(3)	C125	C126	1.396(6)
U2	O12	1.791(3)	C125	C124	1.393(6)
U2	O35	2.365(3)	C136	C135	1.400(6)
U2	O11	1.788(3)	C136	C131	1.402(6)
U2	N56	2.531(4)	C43	C44	1.385(6)
P2	C73	1.797(4)	C71	C72	1.535(7)
P2	C136	1.795(5)	C126	C121	1.395(6)
P2	C126	1.795(4)	C91	C92	1.379(7)
P2	C116	1.798(5)	C111	C116	1.400(6)
P1	C96	1.801(5)	C111	C112	1.378(7)
P1	C71	1.802(5)	C135	C134	1.390(7)
P1	C106	1.800(5)	C54	C53	1.398(7)
P1	C86	1.801(5)	C121	C122	1.390(6)
S21	C22	1.686(5)	C51	C52	1.392(6)
S11	C12	1.690(5)	C17	C18	1.514(7)
S1	C2	1.687(5)	C116	C115	1.392(7)
S31	C32	1.695(5)	C53	C52	1.394(7)
O61	C63	1.438(5)	C131	C132	1.384(7)

O62	C64	1.436(5)	C114	C115	1.380(7)
O25	C24	1.288(5)	C114	C113	1.394(7)
O5	C4	1.284(5)	C92	C93	1.381(7)
O35	C34	1.291(5)	C106	C105	1.383(7)
O15	C14	1.291(5)	C106	C101	1.388(7)
N46	C45	1.336(5)	C86	C85	1.394(7)
N46	C41	1.364(6)	C86	C81	1.402(7)
N13	C14	1.297(5)	C20	C19	1.502(8)
N13	C12	1.383(6)	C124	C123	1.372(7)
N56	C55	1.347(6)	C38	C37	1.501(8)
N56	C51	1.343(6)	C93	C94	1.388(7)
N3	C4	1.297(6)	C9	C10	1.523(7)
N3	C2	1.379(6)	C95	C94	1.387(7)
N23	C22	1.386(6)	C84	C85	1.397(7)
N23	C24	1.299(6)	C84	C83	1.380(8)
N33	C34	1.292(6)	C7	C8	1.522(7)
N33	C32	1.388(6)	C133	C134	1.391(7)
N6	C2	1.337(6)	C133	C132	1.386(8)
N6	C9	1.470(6)	C122	C123	1.398(7)
N6	C7	1.465(6)	C112	C113	1.386(7)
C73	C74	1.529(6)	C83	C82	1.373(8)
N16	C12	1.337(6)	C105	C104	1.393(8)
N16	C17	1.486(6)	C29	C30	1.507(8)
N16	C19	1.462(6)	C101	C102	1.400(8)
C4	C41	1.526(6)	C81	C82	1.393(7)
N36	C32	1.335(6)	C104	C103	1.385(8)
N36	C37	1.475(6)	C102	C103	1.376(9)
N36	C39	1.478(6)	C27	C28	1.517(8)
N26	C22	1.344(6)	C39	C40	1.508(8)
N26	C29	1.478(7)			

Table S6. Bond angles (°) $(EtPPh_3)_2$*syn,anti*-[**1**].

O61	U1	U2	37.11(7)	C37	N36	C39	115.0(4)
O61	U1	O62	72.42(10)	C22	N26	C29	122.9(4)
O61	U1	O5	81.95(10)	C22	N26	C27	121.4(4)
O61	U1	O15	151.40(10)	C27	N26	C29	115.7(4)
O61	U1	N46	145.03(11)	O15	C14	N13	126.5(4)
O62	U1	U2	36.98(7)	O15	C14	C45	115.3(4)
O62	U1	O5	154.12(10)	N13	C14	C45	118.2(4)
O62	U1	O15	79.06(10)	N46	C45	C14	113.1(4)
O62	U1	N46	142.27(11)	N46	C45	C44	121.7(4)
O1	U1	U2	98.78(10)	C44	C45	C14	125.2(4)
O1	U1	O61	88.76(12)	O35	C34	N33	128.7(4)
O1	U1	O62	91.81(13)	O35	C34	C51	114.6(4)
O1	U1	O5	91.16(13)	N33	C34	C51	116.6(4)
O1	U1	O2	179.39(14)	C41	C42	C43	118.0(4)
O1	U1	O15	90.18(12)	C91	C96	P1	121.0(3)
O1	U1	N46	86.23(13)	C91	C96	C95	120.1(4)
O5	U1	U2	117.23(7)	C95	C96	P1	118.9(4)

O5	U1	O15	126.64(10)	N56	C55	C24	112.5(4)
O5	U1	N46	63.60(11)	N56	C55	C54	121.5(4)
O2	U1	U2	81.75(10)	C54	C55	C24	126.0(4)
O2	U1	O61	91.85(12)	C124	C125	C126	119.9(4)
O2	U1	O62	88.42(12)	C135	C136	P2	120.1(3)
O2	U1	O5	88.88(13)	C135	C136	C131	120.1(4)
O2	U1	O15	89.30(12)	C131	C136	P2	119.8(4)
O2	U1	N46	93.24(12)	N3	C2	S1	122.0(3)
O15	U1	U2	115.21(7)	N6	C2	S1	123.6(3)
O15	U1	N46	63.29(11)	N6	C2	N3	114.2(4)
N46	U1	U2	174.84(8)	N46	C41	C4	110.8(4)
O61	U2	U1	36.77(7)	C42	C41	N46	123.3(4)
O61	U2	O25	77.75(10)	C42	C41	C4	125.8(4)
O61	U2	O35	155.13(10)	C42	C43	C44	120.0(4)
O61	U2	N56	141.18(11)	C72	C71	P1	114.8(4)
O62	U2	U1	37.15(7)	N23	C22	S21	119.4(4)
O62	U2	O61	72.25(10)	N26	C22	S21	123.2(4)
O62	U2	O25	150.00(11)	N26	C22	N23	117.2(4)
O62	U2	O35	82.89(10)	C43	C44	C45	118.6(4)
O62	U2	N56	146.41(11)	C125	C126	P2	119.1(3)
O25	U2	U1	113.45(8)	C121	C126	P2	120.8(3)
O25	U2	O35	127.10(11)	C121	C126	C125	120.1(4)
O25	U2	N56	63.52(12)	N13	C12	S11	119.4(3)
O12	U2	U1	82.49(11)	N16	C12	S11	123.5(4)
O12	U2	O61	89.57(13)	N16	C12	N13	116.8(4)
O12	U2	O62	91.87(12)	N33	C32	S31	120.8(3)
O12	U2	O25	88.07(13)	N36	C32	S31	123.0(4)
O12	U2	O35	90.35(14)	N36	C32	N33	116.0(4)
O12	U2	N56	91.57(13)	O25	C24	N23	126.0(4)
O35	U2	U1	118.71(7)	O25	C24	C55	115.8(4)
O35	U2	N56	63.68(11)	N23	C24	C55	118.2(4)
O11	U2	U1	98.75(10)	C92	C91	C96	120.2(4)
O11	U2	O61	90.36(13)	C112	C111	C116	119.8(5)
O11	U2	O62	90.19(13)	C134	C135	C136	119.5(5)
O11	U2	O25	89.77(13)	C55	C54	C53	119.0(4)
O11	U2	O12	177.81(14)	C122	C121	C126	119.3(4)
O11	U2	O35	90.63(13)	N56	C51	C34	114.0(4)
O11	U2	N56	87.10(13)	N56	C51	C52	121.4(4)
N56	U2	U1	173.52(8)	C52	C51	C34	124.4(4)
C73	P2	C116	107.4(2)	N16	C17	C18	111.6(4)
C136	P2	C73	108.7(2)	C111	C116	P2	117.9(3)
C136	P2	C116	108.9(2)	C115	C116	P2	121.6(3)
C126	P2	C73	111.0(2)	C115	C116	C111	119.7(4)
C126	P2	C136	111.2(2)	C52	C53	C54	119.1(4)
C126	P2	C116	109.5(2)	C132	C131	C136	119.5(5)
C96	P1	C71	109.4(2)	C51	C52	C53	118.8(5)
C96	P1	C86	106.0(2)	C115	C114	C113	119.3(5)
C106	P1	C96	111.2(2)	C91	C92	C93	120.4(5)
C106	P1	C71	110.1(2)	C105	C106	P1	120.7(4)
C106	P1	C86	109.2(2)	C105	C106	C101	119.6(5)
C86	P1	C71	110.7(2)	C101	C106	P1	119.7(4)

U1	O61	U2	106.12(11)	C85	C86	P1	122.3(4)
C63	O61	U1	118.6(3)	C85	C86	C81	119.8(4)
C63	O61	U2	120.2(3)	C81	C86	P1	117.8(4)
U2	O62	U1	105.87(11)	C123	C124	C125	120.1(4)
C64	O62	U1	120.9(3)	N16	C19	C20	111.3(4)
C64	O62	U2	119.7(3)	C114	C115	C116	120.5(4)
C24	O25	U2	126.2(3)	C92	C93	C94	119.6(5)
C4	O5	U1	127.6(3)	N6	C9	C10	111.7(4)
C34	O35	U2	127.7(3)	C94	C95	C96	119.0(5)
C14	O15	U1	127.4(3)	C83	C84	C85	120.0(5)
C45	N46	U1	120.2(3)	N6	C7	C8	112.3(4)
C45	N46	C41	118.4(4)	C132	C133	C134	120.1(5)
C41	N46	U1	121.0(3)	C121	C122	C123	120.3(4)
C14	N13	C12	118.4(4)	C135	C134	C133	120.2(5)
C55	N56	U2	120.0(3)	C131	C132	C133	120.6(5)
C51	N56	U2	119.8(3)	C86	C85	C84	119.6(5)
C51	N56	C55	120.2(4)	N36	C37	C38	111.7(5)
C4	N3	C2	121.0(4)	C111	C112	C113	120.1(5)
C24	N23	C22	117.5(4)	C82	C83	C84	120.6(5)
C34	N33	C32	121.5(4)	C124	C123	C122	120.3(4)
C2	N6	C9	122.3(4)	C106	C105	C104	120.0(5)
C2	N6	C7	121.4(4)	N26	C29	C30	112.9(5)
C7	N6	C9	116.3(4)	C95	C94	C93	120.8(5)
C74	C73	P2	112.8(3)	C106	C101	C102	120.3(5)
C12	N16	C17	121.6(4)	C82	C81	C86	119.4(5)
C12	N16	C19	123.0(4)	C112	C113	C114	120.6(5)
C19	N16	C17	115.4(4)	C103	C104	C105	120.1(6)
O5	C4	N3	128.3(4)	C83	C82	C81	120.4(5)
O5	C4	C41	116.4(4)	C103	C102	C101	119.5(5)
N3	C4	C41	115.3(4)	N26	C27	C28	111.7(4)
C32	N36	C37	122.0(4)	C102	C103	C104	120.4(5)
C32	N36	C39	123.0(4)	N36	C39	C40	112.1(4)

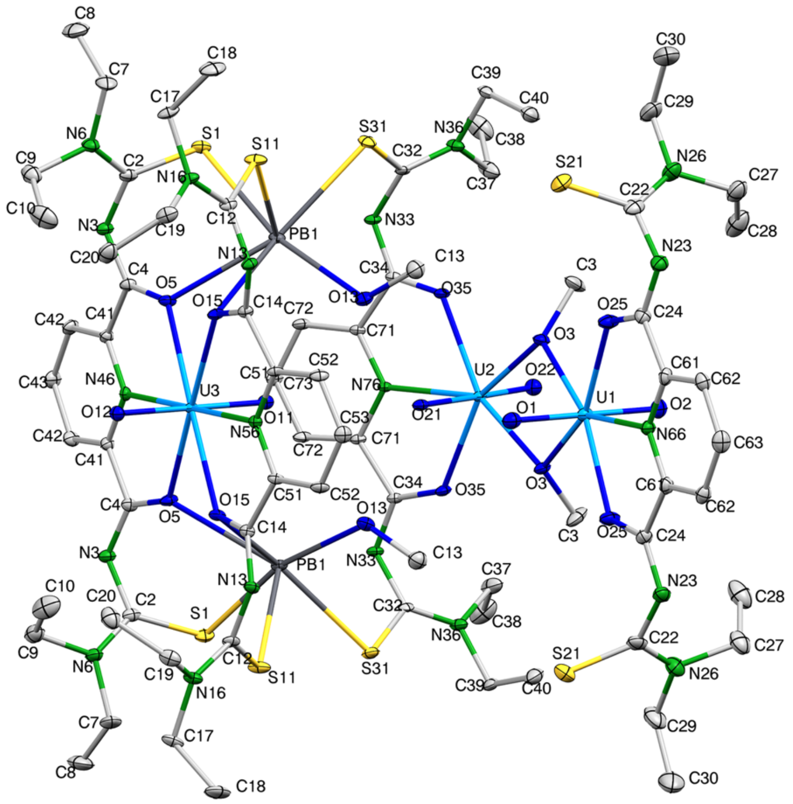

Figure S3. Ellipsoid representation of the structure of $[Pb_2(UO_2)_3(L^{Et2})_3(\mu\text{-}OMe)_2(MeOH)_2]$ (**3**), also illustrating the disorderd parts of the molecule. The thermal ellipsoids are set at a 30% probability level. Hydrogen atoms are omitted for clarity.

Table S7. Bond lengths (Å) $[Pb_2(UO_2)_3(L^{Et2})_3(\mu\text{-}OMe)_2(MeOH)_2]$ (**3**).

U2	U1	3.7114(9)	N6	C7	1.476(5)
U2	O21	1.806(4)	C4	N3	1.294(5)
U2	O35[1]	2.355(2)	C4	C41	1.485(5)
U2	O35	2.355(2)	C4	O5	1.285(4)
U2	O3[1]	2.292(3)	N3	C2	1.378(5)
U2	O3	2.292(3)	C14	O15	1.288(4)
U2	N76	2.541(4)	N16	C12	1.327(5)
U2	O22	1.780(4)	N16	C17	1.471(5)
U3	N46	2.654(4)	N16	C19	1.477(5)
U3	N56	2.647(4)	N23	C22	1.394(5)
U3	O11	1.770(4)	N23	C24	1.297(5)
U3	O5[1]	2.468(2)	C41	C42	1.390(5)
U3	O5	2.468(2)	C52	C53	1.388(4)
U3	O15	2.470(2)	N26	C22	1.337(6)
U3	O15[1]	2.470(2)	N26	C27	1.480(6)
U3	O12	1.763(4)	N26	C29	1.464(6)
Pb1	S11	2.7203(10)	C43	C42	1.382(5)
Pb1	S1	2.8826(11)	C43	C42[1]	1.382(5)
Pb1	S31	2.7954(11)	C63	C62[1]	1.381(5)
Pb1	O5	2.710(3)	C63	C62	1.381(5)

Pb1	O15	2.735(3)	C61	C24	1.498(5)
Pb1	O13	2.686(3)	C61	C62	1.393(5)
U1	O2	1.773(4)	C17	C18	1.518(6)
U1	O25[1]	2.346(3)	C9	C10	1.513(7)
U1	O25	2.346(3)	C7	C8	1.523(7)
U1	O1	1.778(4)	C19	C20	1.517(6)
U1	N66	2.527(5)	C27	C28	1.500(8)
U1	O3[1]	2.359(3)	C29	C30	1.510(9)
U1	O3	2.359(3)	S31	C32	1.700(4)
S11	C12	1.720(4)	O35	C34	1.283(4)
S1	C2	1.713(4)	O3	C3	1.428(5)
S21	C22	1.685(5)	N33	C34	1.303(4)
N46	C41	1.344(4)	N33	C32	1.401(4)
N46	C41[1]	1.344(4)	N76	C71[1]	1.346(4)
N56	C51[1]	1.342(4)	N76	C71	1.346(4)
N56	C51	1.342(4)	C34	C71	1.504(5)
O25	C24	1.285(5)	C32	N36	1.317(5)
C51	C14	1.483(5)	C71	C72	1.384(5)
C51	C52	1.386(5)	O13	C13	1.433(5)
N13	C14	1.296(5)	N36	C39	1.475(5)
N13	C12	1.369(5)	N36	C37	1.469(5)
N66	C61[1]	1.343(4)	C73	C72	1.383(4)
N66	C61	1.343(4)	C73	C72[1]	1.383(4)
N6	C2	1.322(5)	C39	C40	1.521(6)
N6	C9	1.472(5)	C37	C38	1.512(7)

[1]+x,3/2-y,+z

Table S8. Bond angles (°) [Pb$_2$(UO$_2$)$_3$(L^{Et2})$_3$(μ-OMe)$_2$(MeOH)$_2$] (**3**).

O21	U2	U1	80.29(12)	O3	U1	N66	144.30(6)
O21	U2	O35	89.51(9)	O3[1]	U1	O3	71.10(12)
O21	U2	O35[1]	89.51(9)	C12	S11	Pb1	108.63(12)
O21	U2	O3[1]	89.43(12)	C2	S1	Pb1	97.32(13)
O21	U2	O3	89.43(12)	C41[1]	N46	U3	121.1(2)
O21	U2	N76	84.84(15)	C41	N46	U3	121.1(2)
O35	U2	U1	116.12(6)	C41	N46	C41[1]	117.6(4)
O35[1]	U2	U1	116.11(6)	C51[1]	N56	U3	120.9(2)
O35	U2	O35[1]	126.76(12)	C51	N56	U3	120.9(2)
O35	U2	N76	63.45(6)	C51[1]	N56	C51	117.6(4)
O35[1]	U2	N76	63.45(6)	C24	O25	U1	127.9(2)
O3[1]	U2	U1	37.69(6)	N56	C51	C14	112.9(3)
O3	U2	U1	37.69(6)	N56	C51	C52	123.0(3)
O3	U2	O35	79.86(9)	C52	C51	C14	124.1(3)
O3[1]	U2	O35[1]	79.86(9)	C14	N13	C12	124.2(3)
O3	U2	O35[1]	153.36(9)	C61[1]	N66	U1	119.9(2)
O3[1]	U2	O35	153.36(9)	C61	N66	U1	119.9(2)
O3	U2	O3[1]	73.51(13)	C61	N66	C61[1]	120.1(5)
O3	U2	N76	142.84(7)	C2	N6	C9	120.8(3)
O3[1]	U2	N76	142.85(7)	C2	N6	C7	122.9(4)

N76	U2	U1	165.13(10)	C9	N6	C7	116.2(3)
O22	U2	U1	103.34(13)	N3	C4	C41	117.1(3)
O22	U2	O21	176.37(17)	O5	C4	N3	127.8(3)
O22	U2	O35	88.86(9)	O5	C4	C41	115.1(3)
O22	U2	O35^1	88.86(9)	C4	N3	C2	122.2(3)
O22	U2	O3^1	93.47(12)	N13	C14	C51	116.7(3)
O22	U2	O3	93.47(12)	O15	C14	C51	115.7(3)
O22	U2	N76	91.53(16)	O15	C14	N13	127.5(3)
N56	U3	N46	179.27(13)	C12	N16	C17	122.9(3)
O11	U3	N46	94.16(15)	C12	N16	C19	121.1(3)
O11	U3	N56	86.58(15)	C17	N16	C19	115.9(3)
O11	U3	O5^1	87.75(9)	C24	N23	C22	118.2(3)
O11	U3	O5	87.75(9)	N6	C2	S1	122.9(3)
O11	U3	O15	90.71(8)	N6	C2	N3	117.3(4)
O11	U3	O15^1	90.71(8)	N3	C2	S1	119.5(3)
O5^1	U3	N46	59.95(6)	N13	C12	S11	123.7(3)
O5	U3	N46	59.95(6)	N16	C12	S11	119.9(3)
O5	U3	N56	120.11(6)	N16	C12	N13	115.9(3)
O5^1	U3	N56	120.11(6)	N46	C41	C4	113.1(3)
O5	U3	O5^1	119.15(12)	N46	C41	C42	122.8(3)
O5	U3	O15^1	178.33(9)	C42	C41	C4	124.0(3)
O5	U3	O15	60.10(8)	C51	C52	C53	118.8(4)
O5^1	U3	O15	178.33(9)	C22	N26	C27	122.7(4)
O5^1	U3	O15^1	60.10(8)	C22	N26	C29	122.2(4)
O15	U3	N46	119.54(6)	C29	N26	C27	115.1(4)
O15^1	U3	N46	119.54(6)	C52^1	C53	C52	118.5(5)
O15	U3	N56	60.42(6)	C42	C43	C42^1	118.9(5)
O15^1	U3	N56	60.42(6)	C62	C63	C62^1	120.8(5)
O15^1	U3	O15	120.60(12)	C43	C42	C41	118.9(4)
O12	U3	N46	86.18(16)	N66	C61	C24	113.2(3)
O12	U3	N56	93.09(16)	N66	C61	C62	121.4(4)
O12	U3	O11	179.66(17)	C62	C61	C24	125.3(4)
O12	U3	O5	92.42(9)	N23	C22	S21	120.6(4)
O12	U3	O5^1	92.42(9)	N26	C22	S21	122.3(3)
O12	U3	O15	89.12(9)	N26	C22	N23	116.9(4)
O12	U3	O15^1	89.12(9)	N16	C17	C18	111.5(3)
S11	Pb1	S1	83.94(4)	O25	C24	N23	126.5(4)
S11	Pb1	S31	75.60(4)	O25	C24	C61	115.1(3)
S11	Pb1	O15	76.77(6)	N23	C24	C61	118.3(4)
S31	Pb1	S1	81.08(3)	C63	C62	C61	118.1(4)
O5	Pb1	S11	106.67(7)	N6	C9	C10	110.9(4)
O5	Pb1	S1	76.86(6)	N6	C7	C8	110.5(4)
O5	Pb1	S31	157.38(6)	N16	C19	C20	111.4(3)
O5	Pb1	O15	54.01(8)	N26	C27	C28	110.8(4)
O15	Pb1	S1	117.22(6)	N26	C29	C30	112.3(5)
O15	Pb1	S31	144.72(6)	C32	S31	Pb1	97.08(13)
O13	Pb1	S11	88.74(7)	C34	O35	U2	128.0(2)
O13	Pb1	S1	166.81(6)	U3	O5	Pb1	102.84(9)
O13	Pb1	S31	86.48(6)	C4	O5	U3	128.8(2)

O13	Pb1	O5	115.87(8)	C4	O5	Pb1	122.5(2)
O13	Pb1	O15	71.37(8)	U2	O3	U1	105.86(10)
O2	U1	U2	98.32(13)	C3	O3	U2	123.6(3)
O2	U1	O25	89.67(10)	C3	O3	U1	121.6(2)
O2	U1	O25[1]	89.67(10)	U3	O15	Pb1	102.07(9)
O2	U1	O1	178.88(19)	C14	O15	U3	128.6(2)
O2	U1	N66	86.80(17)	C14	O15	Pb1	108.4(2)
O2	U1	O3[1]	89.76(13)	C34	N33	C32	118.0(3)
O2	U1	O3	89.76(13)	C71[1]	N76	U2	119.9(2)
O25[1]	U1	U2	116.13(7)	C71	N76	U2	119.9(2)
O25	U1	U2	116.12(7)	C71[1]	N76	C71	119.8(4)
O25[1]	U1	O25	127.26(14)	O35	C34	N33	126.5(3)
O25	U1	N66	63.65(7)	O35	C34	C71	115.5(3)
O25[1]	U1	N66	63.65(7)	N33	C34	C71	118.0(3)
O25[1]	U1	O3[1]	80.82(9)	N33	C32	S31	118.4(3)
O25[1]	U1	O3	151.92(9)	N36	C32	S31	121.1(3)
O25	U1	O3[1]	151.92(9)	N36	C32	N33	120.4(3)
O25	U1	O3	80.82(9)	N76	C71	C34	112.8(3)
O1	U1	U2	82.80(13)	N76	C71	C72	121.7(3)
O1	U1	O25[1]	89.83(10)	C72	C71	C34	125.5(3)
O1	U1	O25	89.83(10)	C13	O13	Pb1	125.4(2)
O1	U1	N66	92.07(17)	C32	N36	C39	121.3(3)
O1	U1	O3	91.15(13)	C32	N36	C37	123.2(3)
O1	U1	O3[1]	91.15(13)	C37	N36	C39	115.5(3)
N66	U1	U2	174.88(11)	C72	C73	C72[1]	121.0(5)
O3[1]	U1	U2	36.45(6)	C73	C72	C71	117.9(3)
O3	U1	U2	36.45(6)	N36	C39	C40	111.9(3)
O3[1]	U1	N66	144.30(6)	N36	C37	C38	111.8(4)

[1]+x,3/2-y,+z

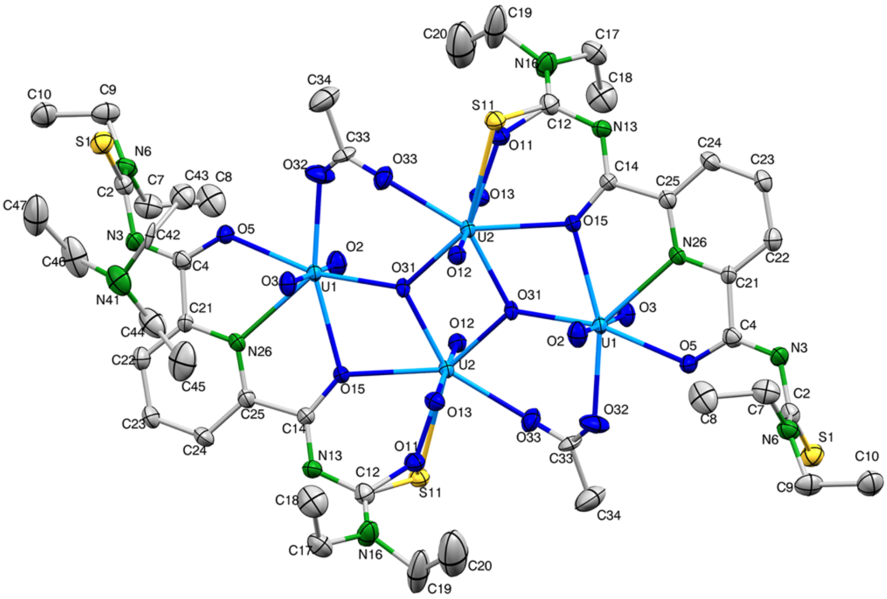

Figure S4. Ellipsoid representation of the structure of $[\{(UO_2)_2(L^{Et2})(\mu_2\text{-OAc})(\mu_3\text{-O})\}_2]$ (**4**), also illustrating the partial S/O (90/10) exchange. The thermal ellipsoids are set at a 30% probability level. Hydrogen atoms are omitted for clarity.

Table S9. Bond lengths (Å) of $[\{(UO_2)_2(L^{Et2})(\mu_2\text{-OAc})(\mu_3\text{-O})\}_2]$ (**4**).

U1	U2	3.8722(5)	N3	C4	1.301(11)
U1	O2	1.768(7)	N3	C2	1.387(11)
U1	O3	1.774(7)	N13	C14	1.274(10)
U1	O15	2.500(5)	N13	C12	1.372(12)
U1	O31	2.240(5)	N16	C12	1.317(13)
U1	O5	2.385(6)	N16	C17	1.493(15)
U1	O32	2.375(7)	N16	C19	1.505(17)
U1	N26	2.558(7)	N6	C2	1.342(13)
U2	U2[1]	3.6555(8)	N6	C9	1.449(12)
U2	S11	2.911(4)	N6	C7	1.494(15)
U2	O11	2.40(8)	C21	C22	1.366(12)
U2	O12	1.802(6)	C21	C4	1.485(12)
U2	O13	1.775(6)	C22	C23	1.403(14)
U2	O15	2.508(5)	C23	C24	1.370(13)
U2	O31	2.254(5)	C24	C25	1.391(12)
U2	O31[1]	2.255(5)	C25	C14	1.509(12)
U2	O33	2.370(6)	C10	C9	1.499(16)
S1	C2	1.681(11)	C7	C8	1.496(18)
S11	O11	0.71(10)	C33	C34	1.470(14)
S11	C12	1.700(12)	C43	C42	1.521(19)
O11	C12	1.47(6)	C42	N41	1.48(2)
O15	C14	1.304(10)	C44	C45	1.47(2)
O5	C4	1.267(11)	C44	N41	1.475(17)
O32	C33	1.269(12)	C17	C18	1.468(18)
O33	C33[1]	1.211(11)	C19	C20	1.34(3)
N26	C21	1.336(10)	N41	C46	1.542(17)
N26	C25	1.350(11)	C46	C47	1.50(2)

[1]2-x,2-y,-z

Table S10. Bond angles (°) in of [{$(UO_2)_2(L^{Et2})(\mu_2\text{-}OAc)(\mu_3\text{-}O)$}$_2$] (4).

O2	U1	U2	82.5(2)	O33	U2	U1	166.47(19)
O2	U1	O3	176.5(3)	O33	U2	U2[1]	114.41(18)
O2	U1	O15	88.6(3)	O33	U2	S11	70.53(19)
O2	U1	O31	92.2(3)	O33	U2	O11	75.7(16)
O2	U1	O5	87.1(3)	O33	U2	O15	141.8(2)
O2	U1	O32	92.0(3)	O11	S11	U2	39(5)
O2	U1	N26	92.0(3)	O11	S11	C12	59(5)
O3	U1	U2	99.0(2)	C12	S11	U2	96.4(4)
O3	U1	O15	90.6(3)	S11	O11	U2	130(6)
O3	U1	O31	90.7(2)	S11	O11	C12	96(6)
O3	U1	O5	90.6(3)	C12	O11	U2	130(6)
O3	U1	O32	89.9(3)	U1	O15	U2	101.27(19)
O3	U1	N26	84.6(3)	C14	O15	U1	125.0(5)
O15	U1	U2	39.44(13)	C14	O15	U2	133.6(5)
O15	U1	N26	62.9(2)	U1	O31	U2	119.0(2)
O31	U1	U2	30.60(14)	U1	O31	U2[1]	131.6(2)
O31	U1	O15	68.30(19)	U2	O31	U2[1]	108.3(2)
O31	U1	O5	166.6(2)	C4	O5	U1	128.0(5)
O31	U1	O32	92.6(2)	C33	O32	U1	122.1(6)
O31	U1	N26	130.8(2)	C33[1]	O33	U2	142.9(7)
O5	U1	U2	161.23(16)	C21	N26	U1	119.7(5)
O5	U1	O15	125.06(19)	C21	N26	C25	119.5(7)
O5	U1	N26	62.6(2)	C25	N26	U1	120.8(6)
O32	U1	U2	121.8(2)	C4	N3	C2	123.4(8)
O32	U1	O15	160.9(2)	C14	N13	C12	122.8(8)
O32	U1	O5	74.1(2)	C12	N16	C17	121.5(9)
O32	U1	N26	136.2(3)	C12	N16	C19	123.5(11)
N26	U1	U2	102.04(16)	C17	N16	C19	115.0(10)
U2[1]	U2	U1	65.940(12)	C2	N6	C9	122.8(9)
S11	U2	U1	108.86(6)	C2	N6	C7	120.9(8)
S11	U2	U2[1]	174.78(6)	C9	N6	C7	116.2(9)
O11	U2	U1	106.1(15)	N26	C21	C22	122.2(8)
O11	U2	U2[1]	167(2)	N26	C21	C4	113.6(7)
O11	U2	S11	11(2)	C22	C21	C4	124.2(8)
O11	U2	O15	67.1(15)	C21	C22	C23	118.9(9)
O12	U2	U1	82.61(19)	C24	C23	C22	119.1(9)
O12	U2	U2[1]	91.6(2)	C23	C24	C25	119.0(9)
O12	U2	S11	87.2(2)	N26	C25	C24	121.3(8)
O12	U2	O11	97(2)	N26	C25	C14	115.8(7)
O12	U2	O15	92.1(2)	C24	C25	C14	122.9(8)
O12	U2	O31[1]	93.1(2)	O5	C4	N3	130.3(8)
O12	U2	O31	89.5(2)	O5	C4	C21	115.6(8)
O12	U2	O33	83.9(3)	N3	C4	C21	114.2(8)
O13	U2	U1	99.6(2)	N3	C2	S1	121.7(8)
O13	U2	U2[1]	93.6(2)	N6	C2	S1	123.7(8)
O13	U2	S11	87.6(2)	N6	C2	N3	114.1(9)
O13	U2	O11	78(2)	O15	C14	C25	114.5(7)

O13	U2	O12	174.8(3)	N13	C14	O15	130.6(8)
O13	U2	O15	86.8(3)	N13	C14	C25	114.8(8)
O13	U2	O31[1]	91.1(3)	O11	C12	S11	25(4)
O13	U2	O31	94.7(2)	N13	C12	S11	121.0(8)
O13	U2	O33	93.9(3)	N13	C12	O11	118(3)
O15	U2	U1	39.29(12)	N16	C12	S11	122.9(8)
O15	U2	U2[1]	103.64(13)	N16	C12	O11	122(3)
O15	U2	S11	71.34(15)	N16	C12	N13	115.9(10)
O31[1]	U2	U1	101.60(13)	N6	C9	C10	112.0(9)
O31	U2	U1	30.39(13)	N6	C7	C8	111.9(10)
O31	U2	U2[1]	35.85(13)	O32	C33	C34	116.2(10)
O31[1]	U2	U2[1]	35.82(13)	O33[1]	C33	O32	123.2(8)
O31	U2	S11	139.00(14)	O33[1]	C33	C34	120.5(10)
O31[1]	U2	S11	149.30(15)	N41	C42	C43	113.0(11)
O31	U2	O11	134.7(16)	C45	C44	N41	112.2(12)
O31[1]	U2	O11	151.4(15)	C18	C17	N16	113.4(11)
O31[1]	U2	O15	139.23(19)	C20	C19	N16	112(2)
O31	U2	O15	67.95(18)	C42	N41	C46	113.2(11)
O31	U2	O31[1]	71.7(2)	C44	N41	C42	117.0(11)
O31	U2	O33	149.5(2)	C44	N41	C46	107.1(11)
O31[1]	U2	O33	79.0(2)	C47	C46	N41	112.2(13)

[1]2-x,2-y,-z

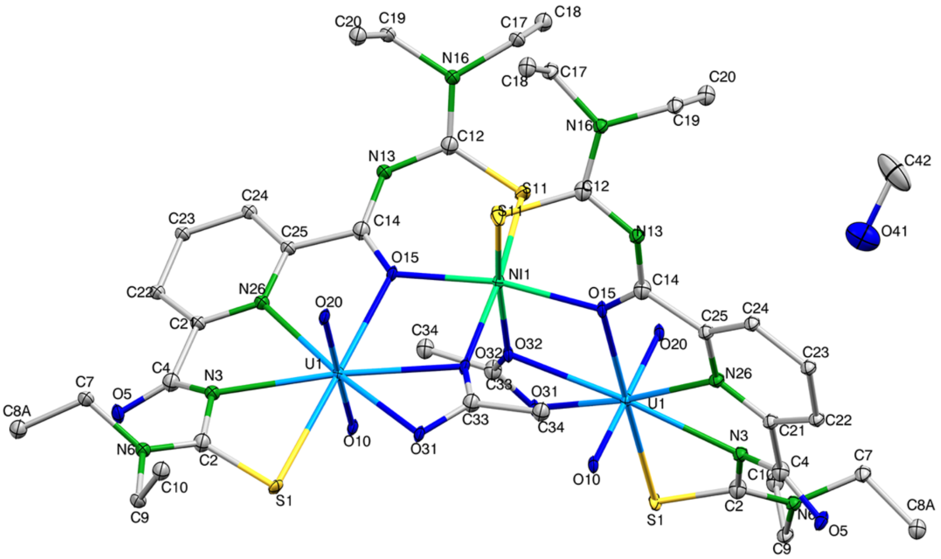

Figure S5. Ellipsoid representation of the structure of $[Ni\{UO_2(L^{Et2})(OAc)\}_2]$ (**5a**) x MeOH. The thermal ellipsoids are set at a 30% probability level. Hydrogen atoms are omitted for clarity.

Table S11. Bond lengths (Å) of $[Ni\{UO_2(L^{Et2})(OAc)\}_2]$ (**5a**).

O21	U2	U1	80.29(12)	O3	U1	N66	144.30(6)
O21	U2	O35	89.51(9)	O3^{1}	U1	O3	71.10(12)
O21	U2	O35^{1}	89.51(9)	C12	S11	Pb1	108.63(12)
O21	U2	O3^{1}	89.43(12)	C2	S1	Pb1	97.32(13)
O21	U2	O3	89.43(12)	C41^{1}	N46	U3	121.1(2)
O21	U2	N76	84.84(15)	C41	N46	U3	121.1(2)
O35	U2	U1	116.12(6)	C41	N46	C41^{1}	117.6(4)
O35^{1}	U2	U1	116.11(6)	C51^{1}	N56	U3	120.9(2)
O35	U2	O35^{1}	126.76(12)	C51	N56	U3	120.9(2)
O35	U2	N76	63.45(6)	C51^{1}	N56	C51	117.6(4)
O35^{1}	U2	N76	63.45(6)	C24	O25	U1	127.9(2)
O3^{1}	U2	U1	37.69(6)	N56	C51	C14	112.9(3)
O3	U2	U1	37.69(6)	N56	C51	C52	123.0(3)
O3	U2	O35	79.86(9)	C52	C51	C14	124.1(3)
O3^{1}	U2	O35^{1}	79.86(9)	C14	N13	C12	124.2(3)
O3	U2	O35^{1}	153.36(9)	C61^{1}	N66	U1	119.9(2)
O3^{1}	U2	O35	153.36(9)	C61	N66	U1	119.9(2)
O3	U2	O3^{1}	73.51(13)	C61	N66	C61^{1}	120.1(5)
O3	U2	N76	142.84(7)	C2	N6	C9	120.8(3)
O3^{1}	U2	N76	142.85(7)	C2	N6	C7	122.9(4)
N76	U2	U1	165.13(10)	C9	N6	C7	116.2(3)
O22	U2	U1	103.34(13)	N3	C4	C41	117.1(3)
O22	U2	O21	176.37(17)	O5	C4	N3	127.8(3)
O22	U2	O35	88.86(9)	O5	C4	C41	115.1(3)
O22	U2	O35^{1}	88.86(9)	C4	N3	C2	122.2(3)
O22	U2	O3^{1}	93.47(12)	N13	C14	C51	116.7(3)
O22	U2	O3	93.47(12)	O15	C14	C51	115.7(3)

O22	U2	N76	91.53(16)	O15	C14	N13	127.5(3)
N56	U3	N46	179.27(13)	C12	N16	C17	122.9(3)
O11	U3	N46	94.16(15)	C12	N16	C19	121.1(3)
O11	U3	N56	86.58(15)	C17	N16	C19	115.9(3)
O11	U3	O5[1]	87.75(9)	C24	N23	C22	118.2(3)
O11	U3	O5	87.75(9)	N6	C2	S1	122.9(3)
O11	U3	O15	90.71(8)	N6	C2	N3	117.3(4)
O11	U3	O15[1]	90.71(8)	N3	C2	S1	119.5(3)
O5[1]	U3	N46	59.95(6)	N13	C12	S11	123.7(3)
O5	U3	N46	59.95(6)	N16	C12	S11	119.9(3)
O5	U3	N56	120.11(6)	N16	C12	N13	115.9(3)
O5[1]	U3	N56	120.11(6)	N46	C41	C4	113.1(3)
O5	U3	O5[1]	119.15(12)	N46	C41	C42	122.8(3)
O5	U3	O15[1]	178.33(9)	C42	C41	C4	124.0(3)
O5	U3	O15	60.10(8)	C51	C52	C53	118.8(4)
O5[1]	U3	O15	178.33(9)	C22	N26	C27	122.7(4)
O5[1]	U3	O15[1]	60.10(8)	C22	N26	C29	122.2(4)
O15	U3	N46	119.54(6)	C29	N26	C27	115.1(4)
O15[1]	U3	N46	119.54(6)	C52[1]	C53	C52	118.5(5)
O15	U3	N56	60.42(6)	C42	C43	C42[1]	118.9(5)
O15[1]	U3	N56	60.42(6)	C62	C63	C62[1]	120.8(5)
O15[1]	U3	O15	120.60(12)	C43	C42	C41	118.9(4)
O12	U3	N46	86.18(16)	N66	C61	C24	113.2(3)
O12	U3	N56	93.09(16)	N66	C61	C62	121.4(4)
O12	U3	O11	179.66(17)	C62	C61	C24	125.3(4)
O12	U3	O5	92.42(9)	N23	C22	S21	120.6(4)
O12	U3	O5[1]	92.42(9)	N26	C22	S21	122.3(3)
O12	U3	O15	89.12(9)	N26	C22	N23	116.9(4)
O12	U3	O15[1]	89.12(9)	N16	C17	C18	111.5(3)
S11	Pb1	S1	83.94(4)	O25	C24	N23	126.5(4)
S11	Pb1	S31	75.60(4)	O25	C24	C61	115.1(3)
S11	Pb1	O15	76.77(6)	N23	C24	C61	118.3(4)
S31	Pb1	S1	81.08(3)	C63	C62	C61	118.1(4)
O5	Pb1	S11	106.67(7)	N6	C9	C10	110.9(4)
O5	Pb1	S1	76.86(6)	N6	C7	C8	110.5(4)
O5	Pb1	S31	157.38(6)	N16	C19	C20	111.4(3)
O5	Pb1	O15	54.01(8)	N26	C27	C28	110.8(4)
O15	Pb1	S1	117.22(6)	N26	C29	C30	112.3(5)
O15	Pb1	S31	144.72(6)	C32	S31	Pb1	97.08(13)
O13	Pb1	S11	88.74(7)	C34	O35	U2	128.0(2)
O13	Pb1	S1	166.81(6)	U3	O5	Pb1	102.84(9)
O13	Pb1	S31	86.48(6)	C4	O5	U3	128.8(2)
O13	Pb1	O5	115.87(8)	C4	O5	Pb1	122.5(2)
O13	Pb1	O15	71.37(8)	U2	O3	U1	105.86(10)
O2	U1	U2	98.32(13)	C3	O3	U2	123.6(3)
O2	U1	O25	89.67(10)	C3	O3	U1	121.6(2)
O2	U1	O25[1]	89.67(10)	U3	O15	Pb1	102.07(9)
O2	U1	O1	178.88(19)	C14	O15	U3	128.6(2)
O2	U1	N66	86.80(17)	C14	O15	Pb1	108.4(2)

O2	U1	O3[1]	89.76(13)	C34	N33	C32	118.0(3)
O2	U1	O3	89.76(13)	C71[1]	N76	U2	119.9(2)
O25[1]	U1	U2	116.13(7)	C71	N76	U2	119.9(2)
O25	U1	U2	116.12(7)	C71[1]	N76	C71	119.8(4)
O25[1]	U1	O25	127.26(14)	O35	C34	N33	126.5(3)
O25	U1	N66	63.65(7)	O35	C34	C71	115.5(3)
O25[1]	U1	N66	63.65(7)	N33	C34	C71	118.0(3)
O25[1]	U1	O3[1]	80.82(9)	N33	C32	S31	118.4(3)
O25[1]	U1	O3	151.92(9)	N36	C32	S31	121.1(3)
O25	U1	O3[1]	151.92(9)	N36	C32	N33	120.4(3)
O25	U1	O3	80.82(9)	N76	C71	C34	112.8(3)
O1	U1	U2	82.80(13)	N76	C71	C72	121.7(3)
O1	U1	O25[1]	89.83(10)	C72	C71	C34	125.5(3)
O1	U1	O25	89.83(10)	C13	O13	Pb1	125.4(2)
O1	U1	N66	92.07(17)	C32	N36	C39	121.3(3)
O1	U1	O3	91.15(13)	C32	N36	C37	123.2(3)
O1	U1	O3[1]	91.15(13)	C37	N36	C39	115.5(3)
N66	U1	U2	174.88(11)	C72	C73	C72[1]	121.0(5)
O3[1]	U1	U2	36.45(6)	C73	C72	C71	117.9(3)
O3	U1	U2	36.45(6)	N36	C39	C40	111.9(3)
O3[1]	U1	N66	144.30(6)	N36	C37	C38	111.8(4)

[1]+x,3/2-y,+z

Table S12. Bond angles (°) in [Ni{UO_2(L^{Et2})(OAc)}$_2$] (**5a**).

S1	U1	C33	91.7(4)	O32	Ni1	O32[1]	82.3(7)
O31	U1	S1	66.5(3)	C33	O31	U1	94.0(10)
O31	U1	C33	25.5(5)	C12	N16	C19	120.9(15)
O31	U1	N26	174.9(4)	C12	N16	C17	122.9(15)
O15	U1	S1	179.0(3)	C17	N16	C19	116.1(14)
O15	U1	O31	113.6(4)	N6	C2	S1	122.7(14)
O15	U1	C33	88.5(5)	N6	C2	N3	123.8(16)
O15	U1	N26	62.2(4)	N3	C2	S1	113.0(13)
N3	U1	S1	57.4(3)	C2	N6	C7	123.8(15)
N3	U1	O31	122.5(4)	C2	N6	C9	120.3(15)
N3	U1	O15	122.3(4)	C9	N6	C7	114.1(14)
N3	U1	C33	147.9(5)	C14	N13	C12	128.0(16)
N3	U1	N26	61.1(5)	N13	C14	C25	115.8(16)
O32	U1	S1	116.2(3)	O15	C14	N13	129.0(17)
O32	U1	O31	51.4(4)	O15	C14	C25	115.1(16)
O32	U1	O15	64.1(4)	N16	C12	S11	121.1(14)
O32	U1	N3	173.6(4)	N16	C12	N13	115.0(16)
O32	U1	C33	26.1(5)	N13	C12	S11	123.6(15)
O32	U1	N26	125.2(4)	C25	C24	C23	119.4(16)
N26	U1	S1	117.7(3)	C21	C22	C23	118.4(16)
N26	U1	C33	150.6(5)	C24	C23	C22	118.6(16)
O10	U1	S1	89.9(4)	N16	C19	C20	109.6(14)
O10	U1	O31	97.4(4)	Ni1	O15	U1	107.6(5)
O10	U1	O15	91.1(5)	C14	O15	U1	126.7(11)

O10	U1	N3	94.3(5)	C14	O15	Ni1	125.2(11)
O10	U1	C33	93.9(5)	C2	N3	U1	105.0(11)
O10	U1	O32	85.3(5)	C4	N3	U1	124.2(12)
O10	U1	N26	85.6(5)	C4	N3	C2	124.7(16)
O20	U1	S1	92.5(4)	C22	C21	C4	121.1(16)
O20	U1	O31	84.2(5)	N26	C21	C22	123.6(16)
O20	U1	O15	86.5(5)	N26	C21	C4	115.2(15)
O20	U1	N3	86.4(5)	O31	C33	U1	60.5(9)
O20	U1	C33	86.8(5)	O31	C33	C34	122.8(16)
O20	U1	O32	94.3(5)	O31	C33	O32	117.1(16)
O20	U1	N26	92.6(5)	C34	C33	U1	175.4(14)
O20	U1	O10	177.5(5)	O32	C33	U1	57.3(8)
C2	S1	U1	81.7(7)	O32	C33	C34	119.9(16)
C12	S11	Ni1	99.2(7)	N6	C7	C8A	112.7(15)
S11	Ni1	S11[1]	99.0(3)	N16	C17	C18	111.1(15)
O15	Ni1	S11[1]	93.1(3)	Ni1	O32	U1	109.8(5)
O15[1]	Ni1	S11	93.1(3)	C33	O32	U1	96.6(10)
O15[1]	Ni1	S11[1]	94.1(3)	C33	O32	Ni1	146.4(12)
O15	Ni1	S11	94.1(3)	C21	N26	U1	120.5(11)
O15	Ni1	O15[1]	169.0(7)	C21	N26	C25	118.2(15)
O32	Ni1	S11	168.7(4)	C25	N26	U1	120.7(11)
O32[1]	Ni1	S11[1]	168.7(4)	C24	C25	C14	123.7(16)
O32[1]	Ni1	S11	89.9(4)	N26	C25	C14	114.6(15)
O32	Ni1	S11[1]	89.9(4)	N26	C25	C24	121.7(16)
O32[1]	Ni1	O15[1]	78.4(4)	O5	C4	N3	128.6(17)
O32	Ni1	O15	78.4(4)	O5	C4	C21	121.0(16)
O32	Ni1	O15[1]	93.2(5)	N3	C4	C21	110.4(16)
O32[1]	Ni1	O15	93.2(5)	N6	C9	C10	112.6(15)

[1]-x,+y,3/2-z

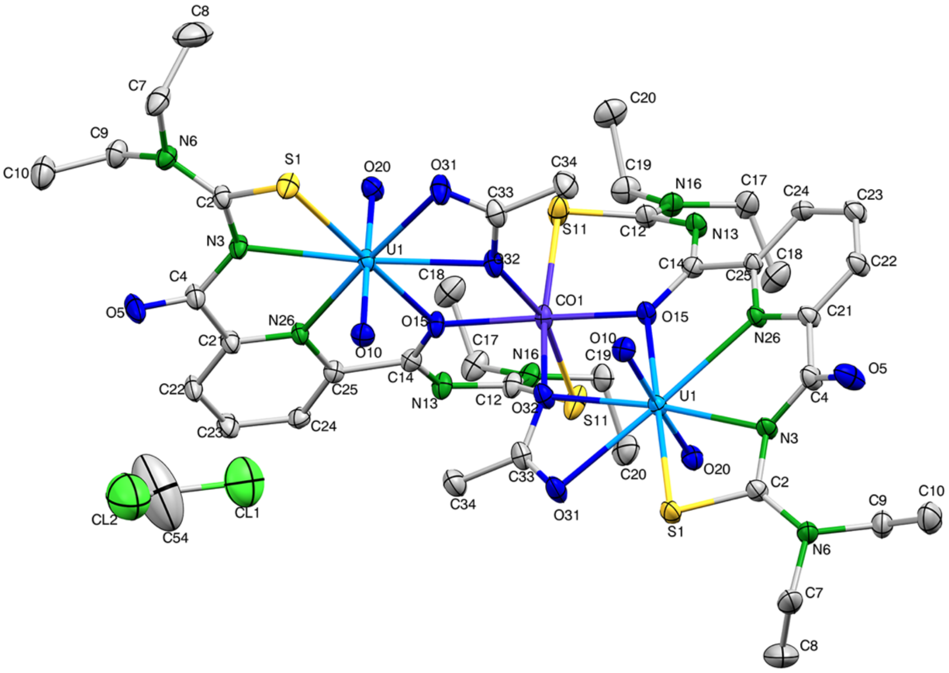

Figure S6. Ellipsoid representation of the structure of [Co{UO_2(L^{Et2})(OAc)}$_2$] (**5b**) x CH_2Cl_2. The thermal ellipsoids are set at a 30% probability level. Hydrogen atoms are omitted for clarity.

Table S13. Bond lengths (Å) of [Co{UO_2(L^{Et2})(OAc)}$_2$] (**5b**).

U1	S1	2.8550(13)	N16	C19	1.468(6)
U1	O31	2.525(3)	N3	C2	1.363(6)
U1	N26	2.565(4)	N3	C4	1.353(6)
U1	O20	1.763(3)	O5	C4	1.220(6)
U1	O10	1.764(3)	C25	C24	1.395(6)
U1	N3	2.478(4)	C25	C14	1.498(6)
U1	O15	2.493(3)	C24	C23	1.380(7)
U1	O32	2.449(3)	C2	N6	1.318(6)
U1	C33	2.885(5)	N6	C7	1.476(7)
Co1	O15	2.133(3)	N6	C9	1.485(6)
Co1	O15[1]	2.133(3)	C22	C23	1.368(7)
Co1	S11[1]	2.3616(16)	C7	C8	1.513(10)
Co1	S11	2.3615(16)	O15	C14	1.286(6)
Co1	O32	2.093(4)	N13	C14	1.295(6)
Co1	O32[1]	2.093(4)	N13	C12	1.371(6)
S1	C2	1.708(5)	S11	C12[1]	1.715(5)
O31	C33	1.236(6)	O32	C33	1.281(6)
N26	C21	1.343(6)	Cl2	C54	1.551(13)
N26	C25	1.346(6)	C33	C34	1.498(7)
C21	C4	1.511(7)	C17	C18	1.507(8)
C21	C22	1.380(7)	C19	C20	1.513(8)
N16	C12	1.328(7)	C9	C10	1.509(8)
N16	C17	1.468(7)	Cl1	C54	1.727(15)

[1] -x,+y,3/2-z

Table S14. Bond angles (°) in [Co{UO$_2$(L^{Et2})(OAc)}$_2$] (**5b**).

S1	U1	C33	91.26(11)	C21	N26	U1	120.2(3)
O31	U1	S1	66.12(9)	C21	N26	C25	118.3(4)
O31	U1	N26	176.09(12)	C25	N26	U1	121.0(3)
O31	U1	C33	25.31(13)	N26	C21	C4	116.0(4)
N26	U1	S1	117.76(9)	N26	C21	C22	122.5(5)
N26	U1	C33	150.89(14)	C22	C21	C4	121.4(4)
O20	U1	S1	93.72(12)	C12	N16	C17	120.7(4)
O20	U1	O31	86.55(14)	C12	N16	C19	122.1(4)
O20	U1	N26	92.57(14)	C19	N16	C17	117.1(4)
O20	U1	O10	176.92(16)	C2	N3	U1	105.7(3)
O20	U1	N3	87.15(15)	C4	N3	U1	123.6(3)
O20	U1	O15	85.75(14)	C4	N3	C2	125.7(4)
O20	U1	O32	92.53(14)	N26	C25	C24	122.2(4)
O20	U1	C33	87.47(15)	N26	C25	C14	114.9(4)
O10	U1	S1	89.24(12)	C24	C25	C14	123.0(4)
O10	U1	O31	95.45(14)	C23	C24	C25	118.0(5)
O10	U1	N26	85.28(14)	N3	C2	S1	111.9(3)
O10	U1	N3	93.76(15)	N6	C2	S1	123.1(4)
O10	U1	O15	91.30(14)	N6	C2	N3	124.8(4)
O10	U1	O32	86.92(14)	N3	C4	C21	111.0(4)
O10	U1	C33	93.34(15)	O5	C4	C21	120.6(4)
N3	U1	S1	56.62(9)	O5	C4	N3	128.4(5)
N3	U1	O31	121.73(12)	C2	N6	C7	119.6(4)
N3	U1	N26	61.98(12)	C2	N6	C9	124.8(4)
N3	U1	O15	123.35(11)	C7	N6	C9	114.2(4)
N3	U1	C33	146.94(14)	C23	C22	C21	118.7(5)
O15	U1	S1	179.46(9)	C22	C23	C24	120.2(5)
O15	U1	O31	113.81(11)	N6	C7	C8	111.7(6)
O15	U1	N26	62.31(11)	Co1	O15	U1	108.77(12)
O15	U1	C33	88.70(13)	C14	O15	U1	126.0(3)
O32	U1	S1	116.53(8)	C14	O15	Co1	125.1(3)
O32	U1	O31	51.36(11)	C14	N13	C12	126.3(4)
O32	U1	N26	124.94(12)	O15	C14	C25	115.0(4)
O32	U1	N3	173.08(11)	O15	C14	N13	129.9(4)
O32	U1	O15	63.49(10)	N13	C14	C25	115.1(4)
O32	U1	C33	26.19(14)	C12[1]	S11	Co1	98.60(18)
O15	Co1	O15[1]	170.83(18)	Co1	O32	U1	111.79(13)
O15	Co1	S11	93.16(10)	C33	O32	U1	96.3(3)
O15	Co1	S11[1]	92.46(9)	C33	O32	Co1	146.8(3)
O15[1]	Co1	S11	92.46(9)	O31	C33	U1	60.8(2)
O15[1]	Co1	S11[1]	93.16(10)	O31	C33	O32	117.9(4)
S11	Co1	S11[1]	104.47(9)	O31	C33	C34	122.5(5)
O32	Co1	O15[1]	96.99(13)	O32	C33	U1	57.5(2)
O32	Co1	O15	75.93(12)	O32	C33	C34	119.7(5)
O32[1]	Co1	O15	96.99(13)	C34	C33	U1	172.0(4)
O32[1]	Co1	O15[1]	75.92(12)	N16	C12	N13	114.5(4)
O32[1]	Co1	S11[1]	88.43(11)	N16	C12	S11[1]	120.7(4)

O32	Co1	S11[1]	163.24(10)	N13	C12	S11[1]	124.5(4)
O32	Co1	S11	88.43(11)	N16	C17	C18	112.6(5)
O32[1]	Co1	S11	163.23(10)	N16	C19	C20	112.4(5)
O32[1]	Co1	O32	81.2(2)	N6	C9	C10	113.5(5)
C2	S1	U1	82.66(16)	Cl2	C54	Cl1	126.3(9)
C33	O31	U1	93.9(3)				

[1]-x,+y,3/2-z

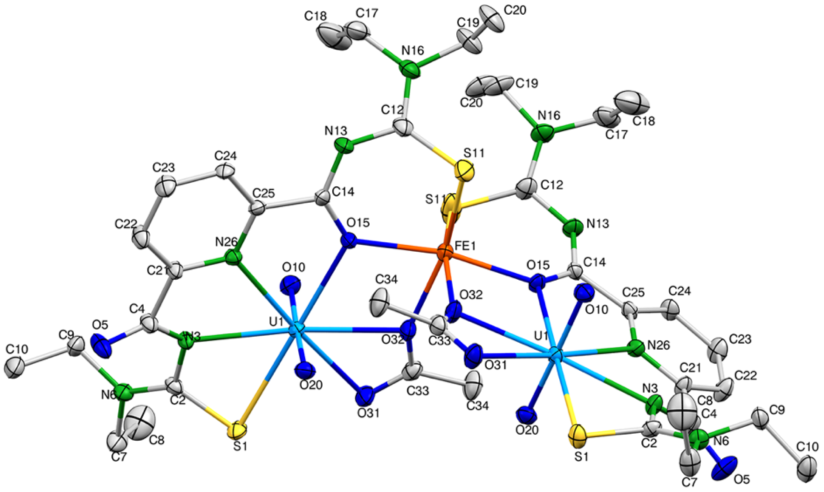

Figure S7. Ellipsoid representation of the structure of [Fe{UO$_2$(L^{Et2})(OAc)}$_2$] (**5c**). The thermal ellipsoids are set at a 30% probability level. Hydrogen atoms are omitted for clarity.

Table S15. Bond lengths (Å) of [Fe{UO$_2$(L^{Et2})(OAc)}$_2$] (**5c**).

U1	S1	2.8593(13)	O15	C14	1.300(6)
U1	O10	1.759(4)	O32	C33	1.278(7)
U1	O20	1.758(4)	N6	C2	1.314(7)
U1	N26	2.561(4)	N6	C7	1.468(7)
U1	O31	2.535(4)	N6	C9	1.472(7)
U1	N3	2.468(4)	O5	C4	1.228(6)
U1	O15	2.494(3)	C21	C22	1.379(7)
U1	O32	2.462(3)	C21	C4	1.501(7)
U1	C33	2.902(5)	C14	N13	1.304(6)
Fe1	S11[1]	2.410(2)	C14	C25	1.495(7)
Fe1	S11	2.410(2)	N13	C12	1.375(7)
Fe1	O15[1]	2.155(3)	C24	C25	1.382(7)
Fe1	O15	2.155(3)	C24	C23	1.388(8)
Fe1	O32	2.119(4)	C22	C23	1.387(8)
Fe1	O32[1]	2.119(4)	C7	C8	1.510(10)
S1	C2	1.723(5)	C12	N16	1.315(7)
S11	C12	1.715(6)	N16	C17	1.464(8)
N26	C21	1.347(6)	N16	C19	1.467(7)
N26	C25	1.355(6)	C33	C34	1.495(7)
O31	C33	1.240(7)	C20	C19	1.535(9)
N3	C2	1.368(6)	C17	C18	1.489(10)
N3	C4	1.348(6)	C9	C10	1.525(8)

[1]1-x,+y,3/2-z

Table S16. Bond angles (°) in [Fe{UO$_2$(L^{Et2})(OAc)}$_2$] (**5c**).

S1	U1	C33	90.70(12)	C12	S11	Fe1	98.1(2)
O10	U1	S1	93.03(13)	C21	N26	U1	120.1(3)
O10	U1	N26	93.67(15)	C21	N26	C25	117.7(4)
O10	U1	O31	85.17(16)	C25	N26	U1	121.9(3)

O10	U1	N3	87.45(16)	C33	O31	U1	94.2(3)
O10	U1	O15	85.98(15)	C2	N3	U1	106.1(3)
O10	U1	O32	92.44(16)	C4	N3	U1	123.9(3)
O10	U1	C33	87.33(17)	C4	N3	C2	125.0(4)
O20	U1	S1	89.61(12)	Fe1	O15	U1	108.54(13)
O20	U1	O10	177.36(18)	C14	O15	U1	125.1(3)
O20	U1	N26	85.04(15)	C14	O15	Fe1	125.9(3)
O20	U1	O31	95.98(16)	Fe1	O32	U1	110.94(15)
O20	U1	N3	93.94(16)	C33	O32	U1	96.6(3)
O20	U1	O15	91.39(14)	C33	O32	Fe1	148.2(4)
O20	U1	O32	86.47(16)	C2	N6	C7	120.2(4)
O20	U1	C33	92.71(17)	C2	N6	C9	123.8(4)
N26	U1	S1	117.65(9)	C7	N6	C9	114.8(4)
N26	U1	C33	151.49(15)	N26	C21	C22	122.6(5)
O31	U1	S1	65.78(9)	N26	C21	C4	115.9(4)
O31	U1	N26	176.47(13)	C22	C21	C4	121.5(5)
O31	U1	C33	25.23(14)	O15	C14	N13	128.8(5)
N3	U1	S1	56.82(9)	O15	C14	C25	116.1(4)
N3	U1	N26	61.68(13)	N13	C14	C25	115.0(4)
N3	U1	O31	121.53(13)	C14	N13	C12	126.4(5)
N3	U1	O15	123.07(12)	C25	C24	C23	118.7(5)
N3	U1	C33	146.73(15)	N26	C25	C14	113.8(4)
O15	U1	S1	179.01(9)	N26	C25	C24	122.8(5)
O15	U1	N26	62.44(12)	C24	C25	C14	123.4(4)
O15	U1	O31	114.12(12)	N3	C2	S1	111.2(4)
O15	U1	C33	89.27(14)	N6	C2	S1	123.6(4)
O32	U1	S1	115.79(9)	N6	C2	N3	124.9(5)
O32	U1	N26	125.71(13)	C21	C22	C23	119.2(5)
O32	U1	O31	51.10(13)	N6	C7	C8	112.1(6)
O32	U1	N3	172.58(13)	C22	C23	C24	118.8(5)
O32	U1	O15	64.30(12)	N3	C4	C21	110.7(4)
O32	U1	C33	25.94(14)	O5	C4	N3	128.7(5)
S11[1]	Fe1	S11	105.64(13)	O5	C4	C21	120.6(5)
O15	Fe1	S11[1]	95.86(11)	N13	C12	S11	124.2(4)
O15	Fe1	S11	91.18(10)	N16	C12	S11	120.6(4)
O15[1]	Fe1	S11[1]	91.18(10)	N16	C12	N13	114.8(5)
O15[1]	Fe1	S11	95.86(11)	C12	N16	C17	120.7(5)
O15	Fe1	O15[1]	168.35(18)	C12	N16	C19	122.3(5)
O32[1]	Fe1	S11	87.47(13)	C17	N16	C19	117.0(5)
O32	Fe1	S11[1]	87.48(13)	O31	C33	U1	60.6(3)
O32[1]	Fe1	S11[1]	162.82(12)	O31	C33	O32	117.8(5)
O32	Fe1	S11	162.82(12)	O31	C33	C34	122.4(5)
O32	Fe1	O15	76.21(13)	O32	C33	U1	57.4(3)
O32	Fe1	O15[1]	94.89(14)	O32	C33	C34	119.8(5)
O32[1]	Fe1	O15	94.89(14)	C34	C33	U1	173.7(4)
O32[1]	Fe1	O15[1]	76.21(13)	N16	C17	C18	111.1(7)
O32[1]	Fe1	O32	82.1(2)	N16	C19	C20	110.9(5)
C2	S1	U1	82.37(17)	N6	C9	C10	114.4(5)

[1]1-x,+y,3/2-z

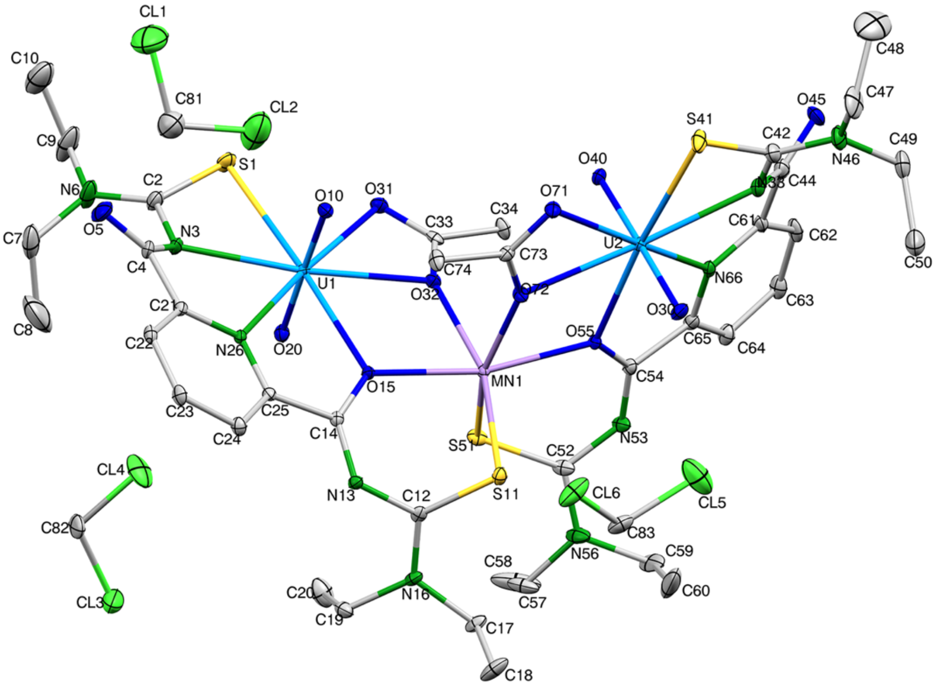

Figure S8. Ellipsoid representation of the structure of [Mn{$UO_2(L^{Et2})(OAc)$}$_2$] (**5d**) x CH_2Cl_2. The thermal ellipsoids are set at a 30% probability level. Hydrogen atoms are omitted for clarity.

Table S17. Bond lengths (Å) of [Mn{$UO_2(L^{Et2})(OAc)$}$_2$] (**5d**).

U2	S41	2.8862(12)	N16	C17	1.474(6)
U2	O40	1.777(3)	N16	C19	1.469(6)
U2	O30	1.771(3)	N26	C21	1.350(5)
U2	O55	2.521(3)	N26	C25	1.342(6)
U2	O72	2.476(3)	N53	C52	1.363(6)
U2	O71	2.519(3)	N53	C54	1.281(6)
U2	N66	2.557(4)	N56	C52	1.329(6)
U2	N33	2.480(4)	N56	C59	1.483(8)
U2	C73	2.899(4)	N56	C57	1.468(7)
U1	S1	2.8712(12)	N66	C61	1.349(6)
U1	O31	2.511(3)	N66	C65	1.334(6)
U1	O32	2.475(3)	N33	C42	1.361(7)
U1	O10	1.768(3)	N33	C44	1.358(6)
U1	O20	1.779(3)	N46	C42	1.331(6)
U1	O15	2.518(3)	N46	C47	1.475(7)
U1	N3	2.472(4)	N46	C49	1.480(7)
U1	N26	2.549(4)	C33	C34	1.501(6)
U1	C33	2.892(4)	C4	C21	1.494(6)
Mn1	S11	2.5395(13)	C7	C8	1.477(13)
Mn1	S51	2.5153(13)	C9	C10	1.467(11)
Mn1	O32	2.177(3)	C14	C25	1.491(6)
Mn1	O15	2.216(3)	C17	C18	1.528(7)
Mn1	O55	2.228(3)	C19	C20	1.518(7)
Mn1	O72	2.164(3)	C21	C22	1.384(6)

S1	C2	1.713(5)	C22	C23	1.390(7)
S11	C12	1.724(5)	C23	C24	1.386(6)
S51	C52	1.721(5)	C24	C25	1.391(6)
S41	C42	1.715(5)	C54	C65	1.498(6)
O31	C33	1.248(6)	C59	C60	1.497(12)
O32	C33	1.284(5)	C57	C58	1.522(8)
O5	C4	1.228(6)	C61	C62	1.390(7)
O15	C14	1.302(5)	C61	C44	1.495(7)
O55	C54	1.297(5)	C62	C63	1.375(8)
O45	C44	1.233(6)	C63	C64	1.385(7)
O72	C73	1.285(5)	C64	C65	1.403(6)
O71	C73	1.247(5)	C47	C48	1.499(11)
N3	C2	1.375(6)	C49	C50	1.515(9)
N3	C4	1.356(6)	C73	C74	1.495(6)
N6	C2	1.321(7)	Cl2	C81	1.763(8)
N6	C7	1.484(8)	Cl1	C81	1.739(8)
N6	C9	1.484(7)	Cl6	C83	1.760(11)
N13	C12	1.377(5)	Cl4	C82	1.700(12)
N13	C14	1.281(6)	Cl3	C82	1.786(12)
N16	C12	1.319(6)	Cl5	C83	1.725(12)

Table S18. Bond angles (°) in [Mn{UO_2(L^{Et2})(OAc)}$_2$] (**5d**).

S41	U2	C73	93.28(9)	Mn1	O55	U2	111.20(12)
O40	U2	S41	92.49(11)	C54	O55	U2	123.9(3)
O40	U2	O55	92.75(12)	C54	O55	Mn1	124.5(3)
O40	U2	O72	89.08(13)	Mn1	O72	U2	115.24(13)
O40	U2	O71	86.26(13)	C73	O72	U2	95.6(3)
O40	U2	N66	83.36(13)	C73	O72	Mn1	148.5(3)
O40	U2	N33	90.76(14)	C73	O71	U2	94.6(3)
O40	U2	C73	87.14(13)	C2	N3	U1	106.3(3)
O30	U2	S41	88.15(11)	C4	N3	U1	120.9(3)
O30	U2	O40	179.25(15)	C4	N3	C2	125.9(4)
O30	U2	O55	86.62(13)	C2	N6	C7	122.4(5)
O30	U2	O72	90.27(13)	C2	N6	C9	121.5(5)
O30	U2	O71	93.62(13)	C7	N6	C9	115.7(5)
O30	U2	N66	96.70(13)	C14	N13	C12	125.9(4)
O30	U2	N33	89.92(14)	C12	N16	C17	122.6(4)
O30	U2	C73	92.43(13)	C12	N16	C19	121.7(4)
O55	U2	S41	174.69(7)	C19	N16	C17	115.7(4)
O55	U2	N66	62.39(11)	C21	N26	U1	119.2(3)
O55	U2	C73	87.88(11)	C25	N26	U1	121.7(3)
O72	U2	S41	119.31(7)	C25	N26	C21	118.6(4)
O72	U2	O55	61.70(9)	C54	N53	C52	128.8(4)
O72	U2	O71	51.57(10)	C52	N56	C59	121.2(5)
O72	U2	N66	123.01(11)	C52	N56	C57	121.7(5)
O72	U2	N33	175.69(12)	C57	N56	C59	117.0(5)
O72	U2	C73	26.18(11)	C61	N66	U2	119.8(3)
O71	U2	S41	68.00(8)	C65	N66	U2	121.7(3)

O71	U2	O55	113.27(10)	C65	N66	C61	118.2(4)
O71	U2	N66	168.45(11)	C42	N33	U2	106.6(3)
O71	U2	C73	25.39(11)	C44	N33	U2	121.4(3)
N66	U2	S41	117.40(9)	C44	N33	C42	125.7(4)
N66	U2	C73	148.13(12)	C42	N46	C47	121.7(5)
N33	U2	S41	56.40(10)	C42	N46	C49	122.3(5)
N33	U2	O55	122.61(12)	C47	N46	C49	115.2(4)
N33	U2	O71	124.12(12)	O31	C33	U1	60.0(2)
N33	U2	N66	61.23(13)	O31	C33	O32	118.2(4)
N33	U2	C73	149.51(13)	O31	C33	C34	122.4(4)
S1	U1	C33	92.90(9)	O32	C33	U1	58.5(2)
O31	U1	S1	67.46(8)	O32	C33	C34	119.4(4)
O31	U1	O15	112.38(10)	C34	C33	U1	174.0(3)
O31	U1	N26	172.76(11)	N3	C2	S1	112.0(3)
O31	U1	C33	25.47(12)	N6	C2	S1	124.4(4)
O32	U1	S1	118.78(8)	N6	C2	N3	123.1(5)
O32	U1	O31	51.62(10)	O5	C4	N3	128.7(5)
O32	U1	O15	61.77(10)	O5	C4	C21	120.5(4)
O32	U1	N26	122.13(11)	N3	C4	C21	110.8(4)
O32	U1	C33	26.24(12)	C8	C7	N6	111.8(7)
O10	U1	S1	91.19(11)	C10	C9	N6	111.0(6)
O10	U1	O31	92.18(13)	N13	C12	S11	121.6(3)
O10	U1	O32	85.98(13)	N16	C12	S11	122.7(3)
O10	U1	O20	178.25(15)	N16	C12	N13	115.4(4)
O10	U1	O15	93.80(13)	O15	C14	C25	115.2(4)
O10	U1	N3	89.61(14)	N13	C14	O15	129.1(4)
O10	U1	N26	83.31(13)	N13	C14	C25	115.7(4)
O10	U1	C33	90.53(13)	N16	C17	C18	110.9(4)
O20	U1	S1	90.45(11)	N16	C19	C20	111.6(4)
O20	U1	O31	89.01(13)	N26	C21	C4	115.3(4)
O20	U1	O32	93.76(13)	N26	C21	C22	122.4(4)
O20	U1	O15	84.55(12)	C22	C21	C4	122.3(4)
O20	U1	N3	90.78(14)	C21	C22	C23	118.5(4)
O20	U1	N26	95.37(13)	C24	C23	C22	119.4(4)
O20	U1	C33	89.99(13)	C23	C24	C25	118.6(4)
O15	U1	S1	175.00(7)	N26	C25	C14	114.6(4)
O15	U1	N26	62.48(10)	N26	C25	C24	122.3(4)
O15	U1	C33	87.15(11)	C24	C25	C14	123.1(4)
N3	U1	S1	56.85(9)	N53	C52	S51	123.5(4)
N3	U1	O31	124.30(12)	N56	C52	S51	121.4(4)
N3	U1	O32	173.77(12)	N56	C52	N53	114.7(4)
N3	U1	O15	123.03(11)	O55	C54	C65	115.2(4)
N3	U1	N26	61.54(12)	N53	C54	O55	130.3(4)
N3	U1	C33	149.74(13)	N53	C54	C65	114.5(4)
N26	U1	S1	118.15(8)	N56	C59	C60	111.1(5)
N26	U1	C33	148.36(12)	N56	C57	C58	109.6(5)
S51	Mn1	S11	99.00(4)	N66	C61	C62	122.6(4)
O32	Mn1	S11	159.51(9)	N66	C61	C44	115.4(4)
O32	Mn1	S51	88.58(9)	C62	C61	C44	121.9(4)

O32	Mn1	O15	71.38(11)	C63	C62	C61	118.6(4)
O32	Mn1	O55	96.65(12)	C62	C63	C64	119.7(4)
O15	Mn1	S11	88.70(8)	C63	C64	C65	118.2(5)
O15	Mn1	S51	97.40(9)	N66	C65	C54	115.3(4)
O15	Mn1	O55	166.51(11)	N66	C65	C64	122.5(4)
O55	Mn1	S11	102.55(9)	C64	C65	C54	122.1(4)
O55	Mn1	S51	88.29(8)	N33	C42	S41	112.2(3)
O72	Mn1	S11	93.89(9)	N46	C42	S41	124.2(4)
O72	Mn1	S51	157.93(9)	N46	C42	N33	123.3(5)
O72	Mn1	O32	85.43(13)	O45	C44	N33	128.6(5)
O72	Mn1	O15	100.75(11)	O45	C44	C61	121.3(5)
O72	Mn1	O55	71.38(11)	N33	C44	C61	110.1(4)
C2	S1	U1	82.57(17)	N46	C47	C48	111.6(6)
C12	S11	Mn1	94.48(14)	N46	C49	C50	111.0(5)
C52	S51	Mn1	98.33(16)	O72	C73	U2	58.2(2)
C42	S41	U2	82.22(17)	O72	C73	C74	119.2(4)
C33	O31	U1	94.6(3)	O71	C73	U2	60.0(2)
Mn1	O32	U1	114.94(13)	O71	C73	O72	118.2(4)
C33	O32	U1	95.3(3)	O71	C73	C74	122.6(4)
C33	O32	Mn1	146.1(3)	C74	C73	U2	177.3(3)
Mn1	O15	U1	111.90(12)	Cl1	C81	Cl2	113.4(4)
C14	O15	U1	122.8(3)	Cl5	C83	Cl6	112.2(6)
C14	O15	Mn1	125.2(3)	Cl4	C82	Cl3	113.9(6)

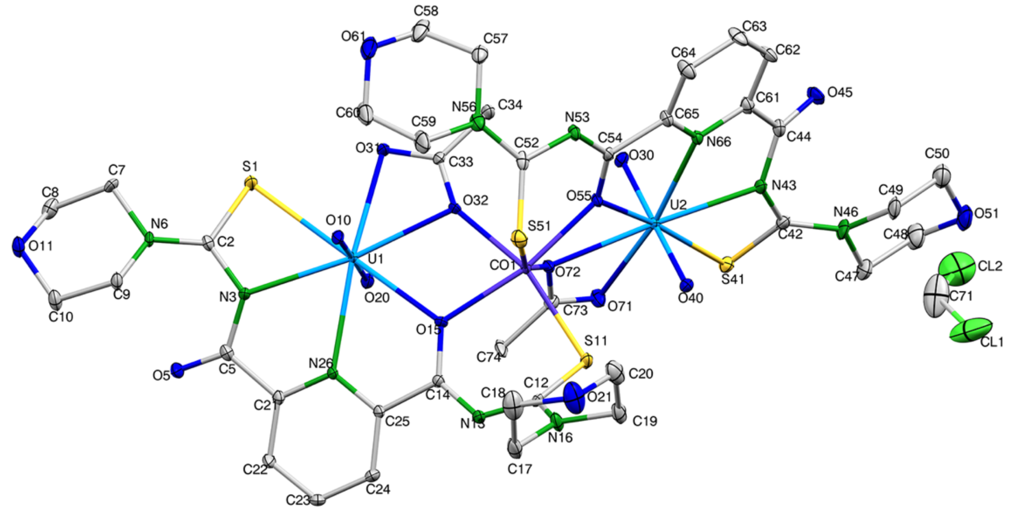

Figure S9. Ellipsoid representation of the structure of [Co{UO_2(L^{morph})(OAc)}$_2$] (**6b**) x CH_2Cl_2. The thermal ellipsoids are set at a 30% probability level. Hydrogen atoms are omitted for clarity.

Table S19. Bond lengths (Å) of [Co{UO_2(L^{morph})(OAc)}$_2$] (**6b**).

U1	O32	2.462(4)	N3	C5	1.364(7)
U1	S1	2.8720(16)	N3	C2	1.366(7)
U1	O10	1.766(4)	N13	C14	1.281(7)
U1	O15	2.487(4)	N13	C12	1.359(7)
U1	O31	2.517(4)	C54	C65	1.504(7)
U1	N26	2.549(4)	C33	C34	1.499(7)
U1	N3	2.460(4)	C25	C24	1.390(8)
U1	O20	1.774(4)	C25	C14	1.498(7)
U1	C33	2.881(5)	C23	C22	1.387(8)
U2	S41	2.8538(15)	C23	C24	1.372(8)
U2	O55	2.490(4)	N6	C9	1.473(7)
U2	O72	2.482(4)	N6	C7	1.468(7)
U2	N66	2.562(5)	N6	C2	1.315(7)
U2	O71	2.505(4)	C22	C21	1.390(8)
U2	N43	2.465(5)	N56	C52	1.333(8)
U2	C73	2.890(6)	N56	C57	1.476(8)
U2	O40	1.770(4)	N56	C59	1.469(8)
U2	O30	1.776(4)	C21	C5	1.501(8)
Co1	S11	2.3967(17)	N16	C12	1.320(8)
Co1	S51	2.4405(18)	N16	C19	1.464(7)
Co1	O55	2.139(4)	N16	C17	1.478(8)
Co1	O32	2.075(4)	C9	C10	1.522(8)
Co1	O15	2.168(4)	C65	C64	1.386(8)
Co1	O72	2.120(4)	O21	C20	1.430(9)
S11	C12	1.730(6)	O21	C18	1.441(9)
S51	C52	1.721(6)	C61	C44	1.492(8)
S41	C42	1.712(6)	C61	C62	1.378(8)
O55	C54	1.294(7)	C73	C74	1.500(8)
O32	C33	1.279(7)	C7	C8	1.520(8)

S1	C2	1.719(6)	O61	C60	1.416(10)
O15	C14	1.301(6)	O61	C58	1.419(10)
O72	C73	1.280(7)	C42	N46	1.332(7)
O5	C5	1.223(7)	C57	C58	1.515(9)
O11	C8	1.429(8)	C63	C62	1.395(9)
O11	C10	1.431(8)	C63	C64	1.388(9)
O45	C44	1.222(7)	C19	C20	1.486(10)
O31	C33	1.247(7)	C60	C59	1.521(12)
N26	C25	1.335(7)	C17	C18	1.493(11)
N26	C21	1.341(7)	N46	C47	1.467(9)
N66	C65	1.339(7)	N46	C49	1.474(9)
N66	C61	1.343(7)	C47	C48	1.521(10)
O71	C73	1.257(7)	O51	C50	1.425(9)
N43	C44	1.364(8)	O51	C48	1.418(9)
N43	C42	1.360(8)	C49	C50	1.512(10)
N53	C54	1.299(7)	Cl1	C71	1.35(2)
N53	C52	1.369(7)	Cl2	C71	1.92(2)

Table S20. Bond angles (°) in [Co{UO_2(L^{morph})(OAc)}$_2$] (**6b**).

O32	U1	S1	117.63(9)	C8	O11	C10	109.4(5)
O32	U1	O15	61.86(12)	C33	O31	U1	93.8(3)
O32	U1	O31	51.63(13)	C25	N26	U1	120.7(3)
O32	U1	N26	124.51(13)	C25	N26	C21	118.5(5)
O32	U1	C33	26.22(14)	C21	N26	U1	119.7(4)
S1	U1	C33	91.73(12)	C65	N66	U2	121.2(3)
O10	U1	O32	89.01(15)	C65	N66	C61	118.9(5)
O10	U1	S1	91.25(14)	C61	N66	U2	119.4(4)
O10	U1	O15	89.04(16)	C73	O71	U2	94.5(4)
O10	U1	O31	93.74(17)	C44	N43	U2	122.2(4)
O10	U1	N26	95.64(17)	C42	N43	U2	106.3(4)
O10	U1	N3	85.48(17)	C42	N43	C44	126.0(5)
O10	U1	O20	178.3(2)	C54	N53	C52	125.4(5)
O10	U1	C33	93.73(17)	C5	N3	U1	124.1(4)
O15	U1	S1	179.41(9)	C5	N3	C2	123.3(5)
O15	U1	O31	113.32(12)	C2	N3	U1	107.1(3)
O15	U1	N26	62.97(13)	C14	N13	C12	125.5(5)
O15	U1	C33	87.74(14)	O55	C54	N53	128.6(5)
O31	U1	S1	66.15(9)	O55	C54	C65	115.2(5)
O31	U1	N26	169.82(14)	N53	C54	C65	116.0(5)
O31	U1	C33	25.58(14)	O32	C33	U1	58.3(3)
N26	U1	S1	117.50(11)	O32	C33	C34	119.5(5)
N26	U1	C33	148.99(15)	O31	C33	U1	60.6(3)
N3	U1	O32	171.81(14)	O31	C33	O32	118.3(5)
N3	U1	S1	56.57(11)	O31	C33	C34	122.1(5)
N3	U1	O15	123.97(14)	C34	C33	U1	171.4(4)
N3	U1	O31	122.66(14)	N26	C25	C24	122.0(5)
N3	U1	N26	62.22(15)	N26	C25	C14	115.4(5)
N3	U1	C33	148.21(16)	C24	C25	C14	122.5(5)

O20	U1	O32	91.58(16)	C24	C23	C22	119.6(5)
O20	U1	S1	89.94(13)	C7	N6	C9	112.3(5)
O20	U1	O15	89.78(16)	C2	N6	C9	125.1(5)
O20	U1	O31	87.91(17)	C2	N6	C7	121.9(5)
O20	U1	N26	82.68(17)	C23	C22	C21	117.7(5)
O20	U1	N3	94.11(17)	C52	N56	C57	123.9(5)
O20	U1	C33	87.51(17)	C52	N56	C59	123.3(6)
S41	U2	C73	92.21(13)	C59	N56	C57	112.6(5)
O55	U2	S41	172.05(10)	N26	C21	C22	122.9(5)
O55	U2	N66	62.52(13)	N26	C21	C5	116.6(5)
O55	U2	O71	112.87(12)	C22	C21	C5	120.5(5)
O55	U2	C73	87.21(15)	C12	N16	C19	125.2(5)
O72	U2	S41	118.38(9)	C12	N16	C17	122.7(5)
O72	U2	O55	61.40(12)	C19	N16	C17	111.2(5)
O72	U2	N66	122.47(13)	N6	C9	C10	109.1(5)
O72	U2	O71	51.77(13)	N66	C65	C54	115.0(5)
O72	U2	C73	26.18(15)	N66	C65	C64	122.4(5)
N66	U2	S41	118.96(11)	C64	C65	C54	122.7(5)
N66	U2	C73	148.56(16)	C20	O21	C18	109.8(5)
O71	U2	S41	66.64(10)	N53	C52	S51	122.9(4)
O71	U2	N66	171.68(14)	N56	C52	S51	122.0(5)
O71	U2	C73	25.69(15)	N56	C52	N53	114.8(5)
N43	U2	S41	57.06(12)	N66	C61	C44	116.5(5)
N43	U2	O55	123.02(15)	N66	C61	C62	122.2(6)
N43	U2	O72	175.41(15)	C62	C61	C44	121.2(5)
N43	U2	N66	62.12(16)	C23	C24	C25	119.1(6)
N43	U2	O71	123.69(15)	O15	C14	C25	114.8(5)
N43	U2	C73	149.24(17)	N13	C14	O15	129.9(5)
O40	U2	S41	87.42(13)	N13	C14	C25	115.3(5)
O40	U2	O55	84.65(16)	O45	C44	N43	127.6(6)
O40	U2	O72	91.14(15)	O45	C44	C61	121.8(6)
O40	U2	N66	95.62(17)	N43	C44	C61	110.6(5)
O40	U2	O71	90.69(17)	O5	C5	N3	128.1(6)
O40	U2	N43	88.17(17)	O5	C5	C21	121.1(5)
O40	U2	C73	89.38(18)	N3	C5	C21	110.8(5)
O40	U2	O30	177.77(18)	O72	C73	U2	58.8(3)
O30	U2	S41	94.49(13)	O72	C73	C74	120.2(5)
O30	U2	O55	93.45(15)	O71	C73	U2	59.8(3)
O30	U2	O72	88.96(15)	O71	C73	O72	118.3(5)
O30	U2	N66	82.46(17)	O71	C73	C74	121.5(6)
O30	U2	O71	91.12(17)	C74	C73	U2	173.1(5)
O30	U2	N43	91.91(17)	N6	C7	C8	108.4(5)
O30	U2	C73	91.69(17)	C60	O61	C58	110.3(6)
S11	Co1	S51	101.50(6)	O11	C8	C7	111.3(5)
O55	Co1	S11	100.72(11)	N43	C42	S41	112.6(4)
O55	Co1	S51	88.62(11)	N46	C42	S41	122.6(5)
O55	Co1	O15	167.82(15)	N46	C42	N43	124.5(6)
O32	Co1	S11	159.48(12)	N3	C2	S1	111.2(4)
O32	Co1	S51	92.68(12)	N6	C2	S1	124.3(5)

O32	Co1	O55	94.31(15)	N6	C2	N3	124.3(5)
O32	Co1	O15	73.66(14)	N56	C57	C58	110.8(5)
O32	Co1	O72	84.69(16)	C64	C63	C62	118.9(6)
O15	Co1	S11	90.54(11)	N13	C12	S11	122.6(5)
O15	Co1	S51	93.70(11)	N16	C12	S11	121.4(4)
O72	Co1	S11	86.37(11)	N16	C12	N13	115.7(5)
O72	Co1	S51	161.31(11)	C61	C62	C63	118.9(5)
O72	Co1	O55	73.17(14)	C65	C64	C63	118.6(6)
O72	Co1	O15	103.24(15)	O11	C10	C9	110.7(5)
C12	S11	Co1	96.21(19)	N16	C19	C20	109.4(6)
C52	S51	Co1	93.8(2)	O21	C20	C19	111.9(6)
C42	S41	U2	82.5(2)	O61	C60	C59	112.7(6)
Co1	O55	U2	108.41(15)	N16	C17	C18	107.9(6)
C54	O55	U2	125.4(3)	O21	C18	C17	110.8(6)
C54	O55	Co1	126.2(3)	O61	C58	C57	110.3(7)
Co1	O32	U1	114.28(16)	N56	C59	C60	109.0(7)
C33	O32	U1	95.5(3)	C42	N46	C47	121.9(6)
C33	O32	Co1	147.4(3)	C42	N46	C49	125.1(6)
C2	S1	U1	82.4(2)	C47	N46	C49	112.1(5)
Co1	O15	U1	109.98(15)	N46	C47	C48	108.8(6)
C14	O15	U1	124.9(3)	C48	O51	C50	109.4(5)
C14	O15	Co1	124.7(3)	N46	C49	C50	108.4(6)
Co1	O72	U2	109.32(16)	O51	C50	C49	110.3(6)
C73	O72	U2	95.0(3)	O51	C48	C47	112.1(7)
C73	O72	Co1	140.7(4)	Cl1	C71	Cl2	127.7(13)

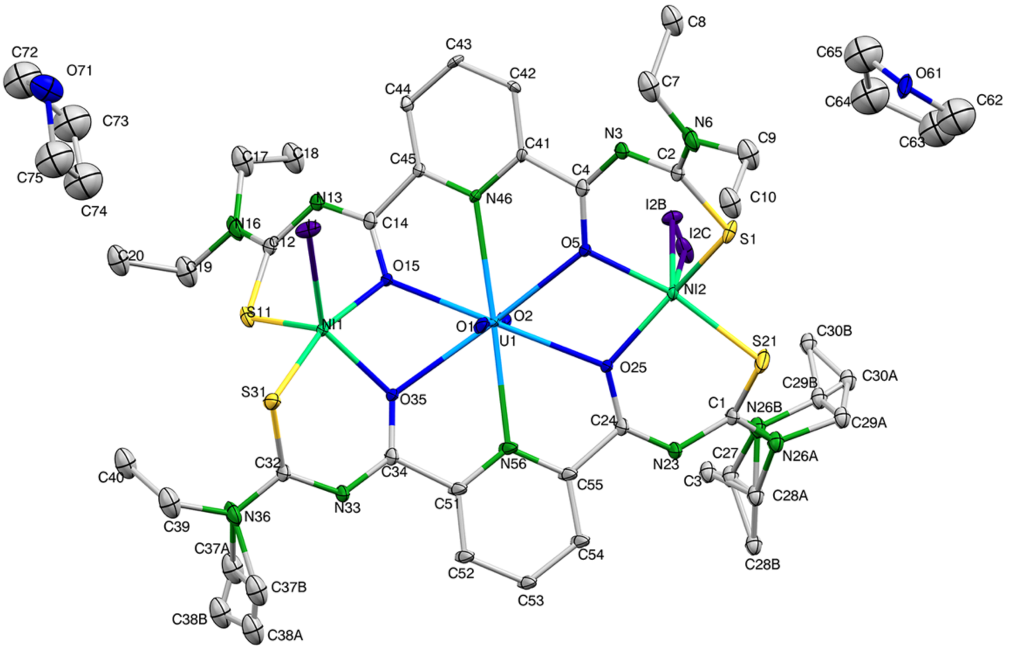

Figure S10. Ellipsoid representation of the structure of $[(UO_2)(NiI)_2(L^{Et2})_2]$ (**7**) x THF, also illustrating the disorderd parts of the molecule. The thermal ellipsoids are set at a 30% probability level. Hydrogen atoms are omitted for clarity.

Table S21. Bond lengths (Å) of $[(UO_2)(NiI)_2(L^{Et2})_2]$ (**7**).

U1	O32	2.462(4)	N3	C5	1.364(7)
U1	S1	2.8720(16)	N3	C2	1.366(7)
U1	O10	1.766(4)	N13	C14	1.281(7)
U1	O15	2.487(4)	N13	C12	1.359(7)
U1	O31	2.517(4)	C54	C65	1.504(7)
U1	N26	2.549(4)	C33	C34	1.499(7)
U1	N3	2.460(4)	C25	C24	1.390(8)
U1	O20	1.774(4)	C25	C14	1.498(7)
U1	C33	2.881(5)	C23	C22	1.387(8)
U2	S41	2.8538(15)	C23	C24	1.372(8)
U2	O55	2.490(4)	N6	C9	1.473(7)
U2	O72	2.482(4)	N6	C7	1.468(7)
U2	N66	2.562(5)	N6	C2	1.315(7)
U2	O71	2.505(4)	C22	C21	1.390(8)
U2	N43	2.465(5)	N56	C52	1.333(8)
U2	C73	2.890(6)	N56	C57	1.476(8)
U2	O40	1.770(4)	N56	C59	1.469(8)
U2	O30	1.776(4)	C21	C5	1.501(8)
Co1	S11	2.3967(17)	N16	C12	1.320(8)
Co1	S51	2.4405(18)	N16	C19	1.464(7)
Co1	O55	2.139(4)	N16	C17	1.478(8)
Co1	O32	2.075(4)	C9	C10	1.522(8)
Co1	O15	2.168(4)	C65	C64	1.386(8)
Co1	O72	2.120(4)	O21	C20	1.430(9)
S11	C12	1.730(6)	O21	C18	1.441(9)

S51	C52	1.721(6)	C61	C44	1.492(8)
S41	C42	1.712(6)	C61	C62	1.378(8)
O55	C54	1.294(7)	C73	C74	1.500(8)
O32	C33	1.279(7)	C7	C8	1.520(8)
S1	C2	1.719(6)	O61	C60	1.416(10)
O15	C14	1.301(6)	O61	C58	1.419(10)
O72	C73	1.280(7)	C42	N46	1.332(7)
O5	C5	1.223(7)	C57	C58	1.515(9)
O11	C8	1.429(8)	C63	C62	1.395(9)
O11	C10	1.431(8)	C63	C64	1.388(9)
O45	C44	1.222(7)	C19	C20	1.486(10)
O31	C33	1.247(7)	C60	C59	1.521(12)
N26	C25	1.335(7)	C17	C18	1.493(11)
N26	C21	1.341(7)	N46	C47	1.467(9)
N66	C65	1.339(7)	N46	C49	1.474(9)
N66	C61	1.343(7)	C47	C48	1.521(10)
O71	C73	1.257(7)	O51	C50	1.425(9)
N43	C44	1.364(8)	O51	C48	1.418(9)
N43	C42	1.360(8)	C49	C50	1.512(10)
N53	C54	1.299(7)	Cl1	C71	1.35(2)
N53	C52	1.369(7)	Cl2	C71	1.92(2)

Table S22. Bond angles (°) in $[(UO_2)(NiI)_2(L^{Et2})_2]$ (**7**).

O32	U1	S1	117.63(9)	C8	O11	C10	109.4(5)
O32	U1	O15	61.86(12)	C33	O31	U1	93.8(3)
O32	U1	O31	51.63(13)	C25	N26	U1	120.7(3)
O32	U1	N26	124.51(13)	C25	N26	C21	118.5(5)
O32	U1	C33	26.22(14)	C21	N26	U1	119.7(4)
S1	U1	C33	91.73(12)	C65	N66	U2	121.2(3)
O10	U1	O32	89.01(15)	C65	N66	C61	118.9(5)
O10	U1	S1	91.25(14)	C61	N66	U2	119.4(4)
O10	U1	O15	89.04(16)	C73	O71	U2	94.5(4)
O10	U1	O31	93.74(17)	C44	N43	U2	122.2(4)
O10	U1	N26	95.64(17)	C42	N43	U2	106.3(4)
O10	U1	N3	85.48(17)	C42	N43	C44	126.0(5)
O10	U1	O20	178.3(2)	C54	N53	C52	125.4(5)
O10	U1	C33	93.73(17)	C5	N3	U1	124.1(4)
O15	U1	S1	179.41(9)	C5	N3	C2	123.3(5)
O15	U1	O31	113.32(12)	C2	N3	U1	107.1(3)
O15	U1	N26	62.97(13)	C14	N13	C12	125.5(5)
O15	U1	C33	87.74(14)	O55	C54	N53	128.6(5)
O31	U1	S1	66.15(9)	O55	C54	C65	115.2(5)
O31	U1	N26	169.82(14)	N53	C54	C65	116.0(5)
O31	U1	C33	25.58(14)	O32	C33	U1	58.3(3)
N26	U1	S1	117.50(11)	O32	C33	C34	119.5(5)
N26	U1	C33	148.99(15)	O31	C33	U1	60.6(3)
N3	U1	O32	171.81(14)	O31	C33	O32	118.3(5)
N3	U1	S1	56.57(11)	O31	C33	C34	122.1(5)

N3	U1	O15	123.97(14)
N3	U1	O31	122.66(14)
N3	U1	N26	62.22(15)
N3	U1	C33	148.21(16)
O20	U1	O32	91.58(16)
O20	U1	S1	89.94(13)
O20	U1	O15	89.78(16)
O20	U1	O31	87.91(17)
O20	U1	N26	82.68(17)
O20	U1	N3	94.11(17)
O20	U1	C33	87.51(17)
S41	U2	C73	92.21(13)
O55	U2	S41	172.05(10)
O55	U2	N66	62.52(13)
O55	U2	O71	112.87(12)
O55	U2	C73	87.21(15)
O72	U2	S41	118.38(9)
O72	U2	O55	61.40(12)
O72	U2	N66	122.47(13)
O72	U2	O71	51.77(13)
O72	U2	C73	26.18(15)
N66	U2	S41	118.96(11)
N66	U2	C73	148.56(16)
O71	U2	S41	66.64(10)
O71	U2	N66	171.68(14)
O71	U2	C73	25.69(15)
N43	U2	S41	57.06(12)
N43	U2	O55	123.02(15)
N43	U2	O72	175.41(15)
N43	U2	N66	62.12(16)
N43	U2	O71	123.69(15)
N43	U2	C73	149.24(17)
O40	U2	S41	87.42(13)
O40	U2	O55	84.65(16)
O40	U2	O72	91.14(15)
O40	U2	N66	95.62(17)
O40	U2	O71	90.69(17)
O40	U2	N43	88.17(17)
O40	U2	C73	89.38(18)
O40	U2	O30	177.77(18)
O30	U2	S41	94.49(13)
O30	U2	O55	93.45(15)
O30	U2	O72	88.96(15)
O30	U2	N66	82.46(17)
O30	U2	O71	91.12(17)
O30	U2	N43	91.91(17)
O30	U2	C73	91.69(17)
S11	Co1	S51	101.50(6)
O55	Co1	S11	100.72(11)
C34	C33	U1	171.4(4)
N26	C25	C24	122.0(5)
N26	C25	C14	115.4(5)
C24	C25	C14	122.5(5)
C24	C23	C22	119.6(5)
C7	N6	C9	112.3(5)
C2	N6	C9	125.1(5)
C2	N6	C7	121.9(5)
C23	C22	C21	117.7(5)
C52	N56	C57	123.9(5)
C52	N56	C59	123.3(6)
C59	N56	C57	112.6(5)
N26	C21	C22	122.9(5)
N26	C21	C5	116.6(5)
C22	C21	C5	120.5(5)
C12	N16	C19	125.2(5)
C12	N16	C17	122.7(5)
C19	N16	C17	111.2(5)
N6	C9	C10	109.1(5)
N66	C65	C54	115.0(5)
N66	C65	C64	122.4(5)
C64	C65	C54	122.7(5)
C20	O21	C18	109.8(5)
N53	C52	S51	122.9(4)
N56	C52	S51	122.0(5)
N56	C52	N53	114.8(5)
N66	C61	C44	116.5(5)
N66	C61	C62	122.2(6)
C62	C61	C44	121.2(5)
C23	C24	C25	119.1(6)
O15	C14	C25	114.8(5)
N13	C14	O15	129.9(5)
N13	C14	C25	115.3(5)
O45	C44	N43	127.6(6)
O45	C44	C61	121.8(6)
N43	C44	C61	110.6(5)
O5	C5	N3	128.1(6)
O5	C5	C21	121.1(5)
N3	C5	C21	110.8(5)
O72	C73	U2	58.8(3)
O72	C73	C74	120.2(5)
O71	C73	U2	59.8(3)
O71	C73	O72	118.3(5)
O71	C73	C74	121.5(6)
C74	C73	U2	173.1(5)
N6	C7	C8	108.4(5)
C60	O61	C58	110.3(6)
O11	C8	C7	111.3(5)
N43	C42	S41	112.6(4)

O55	Co1	S51	88.62(11)	N46	C42	S41	122.6(5)
O55	Co1	O15	167.82(15)	N46	C42	N43	124.5(6)
O32	Co1	S11	159.48(12)	N3	C2	S1	111.2(4)
O32	Co1	S51	92.68(12)	N6	C2	S1	124.3(5)
O32	Co1	O55	94.31(15)	N6	C2	N3	124.3(5)
O32	Co1	O15	73.66(14)	N56	C57	C58	110.8(5)
O32	Co1	O72	84.69(16)	C64	C63	C62	118.9(6)
O15	Co1	S11	90.54(11)	N13	C12	S11	122.6(5)
O15	Co1	S51	93.70(11)	N16	C12	S11	121.4(4)
O72	Co1	S11	86.37(11)	N16	C12	N13	115.7(5)
O72	Co1	S51	161.31(11)	C61	C62	C63	118.9(5)
O72	Co1	O55	73.17(14)	C65	C64	C63	118.6(6)
O72	Co1	O15	103.24(15)	O11	C10	C9	110.7(5)
C12	S11	Co1	96.21(19)	N16	C19	C20	109.4(6)
C52	S51	Co1	93.8(2)	O21	C20	C19	111.9(6)
C42	S41	U2	82.5(2)	O61	C60	C59	112.7(6)
Co1	O55	U2	108.41(15)	N16	C17	C18	107.9(6)
C54	O55	U2	125.4(3)	O21	C18	C17	110.8(6)
C54	O55	Co1	126.2(3)	O61	C58	C57	110.3(7)
Co1	O32	U1	114.28(16)	N56	C59	C60	109.0(7)
C33	O32	U1	95.5(3)	C42	N46	C47	121.9(6)
C33	O32	Co1	147.4(3)	C42	N46	C49	125.1(6)
C2	S1	U1	82.4(2)	C47	N46	C49	112.1(5)
Co1	O15	U1	109.98(15)	N46	C47	C48	108.8(6)
C14	O15	U1	124.9(3)	C48	O51	C50	109.4(5)
C14	O15	Co1	124.7(3)	N46	C49	C50	108.4(6)
Co1	O72	U2	109.32(16)	O51	C50	C49	110.3(6)
C73	O72	U2	95.0(3)	O51	C48	C47	112.1(7)
C73	O72	Co1	140.7(4)	Cl1	C71	Cl2	127.7(13)

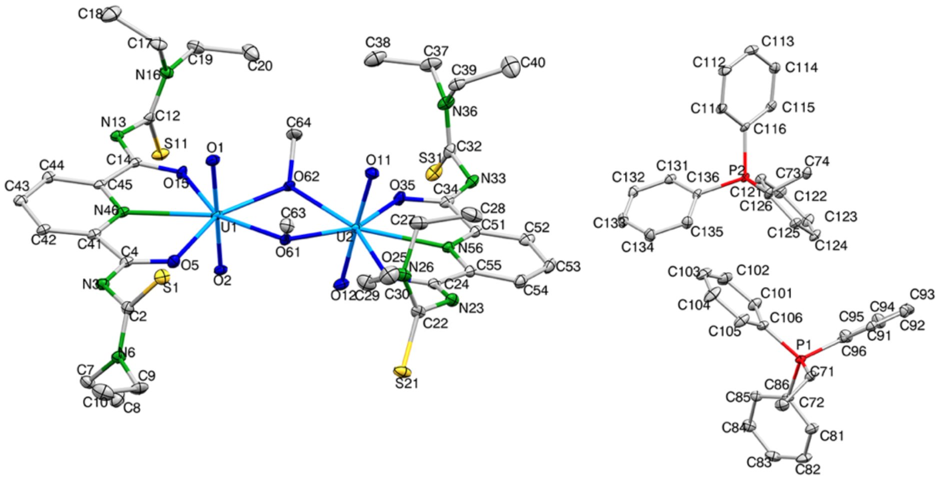

Figure S11. Ellipsoid representation of the structure of $(EtPPh_3)_2$*anti,anti*-[{UO_2(L^{morph})(μ-OMe)}$_2$], $(EtPPh_3)_2$*anti,anti*-[**8**], also illustrating the disorderd parts of the molecule. The thermal ellipsoids are set at a 30% probability level. Hydrogen atoms are omitted for clarity.

Table S23. Bond lengths (Å) of $(EtPPh_3)_2$*anti,anti*-[{UO_2(L^{morph})(μ-OMe)}$_2$], $(EtPPh_3)_2$*anti,anti*-[**8**].

U1	U1^{1}	3.822(2)	C53	C54	1.386(7)
U1	O1	1.784(3)	C54	C55	1.392(6)
U1	O2	1.791(3)	C55	N56	1.343(5)
U1	O5	2.372(3)	O61	C62	1.445(5)
U1	O15	2.362(3)	O62	C64	1.443(5)
U1	N46	2.557(4)	P1	C71	1.805(4)
U1	O61^{1}	2.365(3)	P1	C86	1.804(5)
U1	O61	2.342(3)	P1	C96	1.802(5)
U2	U2^{2}	3.8308(18)	P1	C106	1.795(5)
U2	O3	1.785(3)	P2	C73	1.800(5)
U2	O4	1.794(3)	P2	C126	1.796(6)
U2	O25	2.355(3)	P2	C136	1.796(5)
U2	O35	2.367(3)	P2	C80	1.763(14)
U2	N56	2.555(4)	P2	C9	1.877(11)
U2	O62	2.356(3)	C71	C72	1.532(6)
U2	O62^{2}	2.358(3)	C73	C74	1.537(7)
S1	C2	1.659(6)	C81	C82	1.395(7)
C2	N3	1.413(7)	C81	C86	1.394(7)
C2	N1	1.338(8)	C82	C83	1.382(9)
N3	C4	1.302(6)	C83	C84	1.391(8)
C4	O5	1.289(5)	C84	C85	1.383(7)
C4	C41	1.510(6)	C85	C86	1.404(7)
C8	C7A	1.435(14)	C91	C92	1.377(6)
C8	C7B	1.336(15)	C91	C96	1.395(6)
C8	O10	1.429(9)	C92	C93	1.402(6)
C7A	N1	1.500(13)	C93	C94	1.389(6)
C9A	C10	1.414(13)	C94	C95	1.387(6)
C9A	N1	1.560(14)	C95	C96	1.397(6)
C7B	N1	1.671(18)	C101	C102	1.385(9)
C9B	C10	1.295(15)	C101	C106	1.389(7)

C9B	N1	1.562(18)	C102	C103	1.374(10)
C10	O10	1.404(9)	C103	C104	1.374(9)
S11	C12	1.671(7)	C105	C104	1.393(7)
C12	N13	1.400(6)	C105	C106	1.393(7)
C12	N16	1.357(8)	C121	C122	1.362(12)
N13	C14	1.292(6)	C121	C126	1.397(8)
C14	O15	1.295(5)	C122	C123	1.368(13)
C14	C45	1.508(6)	C123	C124	1.394(11)
N16	C17	1.470(7)	C124	C125	1.393(8)
N16	C19	1.455(10)	C125	C126	1.387(8)
C17	C18	1.514(10)	C131	C132	1.395(8)
C18	O20	1.363(9)	C131	C136	1.369(8)
C19	C20	1.516(10)	C132	C133	1.373(8)
C20	O20	1.429(8)	C133	C134	1.377(9)
S21	C22	1.676(6)	C134	C135	1.384(8)
C22	N23	1.411(6)	C135	C136	1.394(7)
C22	N26	1.341(7)	C80	C75	1.40(2)
N23	C24	1.291(6)	C80	C79	1.398(16)
C24	O25	1.295(5)	C80	C7	1.794(17)
C24	C51	1.503(6)	C80	C5	1.277(18)
N26	C27	1.475(7)	C80	C9	0.536(13)
N26	C29	1.469(7)	C75	C76	1.396(18)
C27	C28	1.480(10)	C75	C1	1.745(19)
C28	O30	1.450(8)	C75	C5	0.542(14)
C29	C30	1.515(10)	C75	C9	1.677(18)
C30	O30	1.438(9)	C76	C77	1.419(14)
S31	C32	1.679(6)	C76	C3	2.04(2)
C32	N33	1.398(6)	C76	C1	0.759(15)
C32	N36	1.330(7)	C76	C5	1.300(16)
N33	C34	1.297(6)	C79	C78	1.400(13)
C34	O35	1.296(5)	C79	C6	1.678(15)
C34	C55	1.498(6)	C79	C7	0.924(13)
N36	C37	1.482(8)	C79	C9	1.265(15)
N36	C39	1.486(8)	C77	C78	1.385(13)
C37	C38	1.452(10)	C77	C3	1.061(18)
C38	O40	1.414(9)	C77	C1	1.039(16)
C39	C40	1.470(9)	C78	C6	1.163(17)
C40	O40	1.455(9)	C78	C7	1.792(15)
C41	C42	1.387(6)	C78	C3	1.403(16)
C41	N46	1.349(5)	C78	C1	1.994(17)
C42	C43	1.391(7)	C6	C7	1.385(17)
C43	C44	1.380(7)	C6	C3	1.36(2)
C44	C45	1.392(6)	C7	C9	1.385(16)
C45	N46	1.341(5)	C3	C1	1.33(2)
C51	C52	1.391(6)	C1	C5	1.403(18)
C51	N56	1.344(5)	C5	C9	1.392(17)
C52	C53	1.383(6)			

[1]1-x,2-y,1-z; [2]1-x,1-y,2-z

Table S24. Bond angles (°) in $(EtPPh_3)_2$*anti,anti*-[{UO_2(L^{morph})(μ-OMe)}$_2$], $(EtPPh_3)_2$*anti,anti*-[**8**].

O1	U1	U1[1]	90.01(11)	C81	C86	P1	121.5(4)
O1	U1	O2	178.77(13)	C81	C86	C85	120.3(4)
O1	U1	O5	89.03(13)	C85	C86	P1	118.2(4)
O1	U1	O15	89.05(13)	C92	C91	C96	120.5(4)
O1	U1	N46	89.17(13)	C91	C92	C93	119.8(4)
O1	U1	O61[1]	93.65(13)	C94	C93	C92	119.9(4)
O1	U1	O61	86.34(12)	C95	C94	C93	120.1(4)
O2	U1	U1[1]	89.77(11)	C94	C95	C96	120.0(4)
O2	U1	O5	90.03(13)	C91	C96	P1	119.9(3)
O2	U1	O15	92.14(13)	C91	C96	C95	119.7(4)
O2	U1	N46	91.12(14)	C95	C96	P1	120.3(3)
O2	U1	O61	92.78(12)	C102	C101	C106	119.3(6)
O2	U1	O61[1]	86.87(13)	C103	C102	C101	120.6(6)
O5	U1	U1[1]	119.98(8)	C102	C103	C104	120.3(6)
O5	U1	N46	63.22(11)	C104	C105	C106	119.0(5)
O15	U1	U1[1]	113.44(7)	C103	C104	C105	120.4(6)
O15	U1	O5	126.54(11)	C101	C106	P1	118.8(4)
O15	U1	N46	63.33(10)	C101	C106	C105	120.4(5)
O15	U1	O61[1]	78.20(10)	C105	C106	P1	120.8(4)
N46	U1	U1[1]	176.68(8)	C122	C121	C126	120.7(8)
O61[1]	U1	U1[1]	35.49(7)	C121	C122	C123	120.1(7)
O61	U1	U1[1]	35.90(7)	C122	C123	C124	120.9(7)
O61[1]	U1	O5	155.19(10)	C125	C124	C123	119.2(7)
O61	U1	O5	84.20(11)	C126	C125	C124	119.7(6)
O61	U1	O15	148.85(10)	C121	C126	P2	122.0(5)
O61[1]	U1	N46	141.39(10)	C125	C126	P2	118.4(4)
O61	U1	N46	147.19(10)	C125	C126	C121	119.5(6)
O61	U1	O61[1]	71.39(12)	C136	C131	C132	120.6(5)
O3	U2	U2[2]	89.14(10)	C133	C132	C131	119.9(6)
O3	U2	O4	179.73(14)	C132	C133	C134	119.8(5)
O3	U2	O25	89.32(12)	C133	C134	C135	120.4(5)
O3	U2	O35	90.90(12)	C134	C135	C136	120.0(5)
O3	U2	N56	90.43(13)	C131	C136	P2	122.2(4)
O3	U2	O62	87.16(12)	C131	C136	C135	119.2(5)
O3	U2	O62[2]	91.44(12)	C135	C136	P2	118.5(4)
O4	U2	U2[2]	90.59(10)	P2	C80	C7	107.8(8)
O4	U2	O25	90.86(12)	C75	C80	P2	119.6(10)
O4	U2	O35	89.16(12)	C75	C80	C7	124.2(11)
O4	U2	N56	89.84(12)	C79	C80	P2	120.1(10)
O4	U2	O62[2]	88.31(12)	C79	C80	C75	120.3(12)
O4	U2	O62	92.65(12)	C79	C80	C7	30.5(6)
O25	U2	U2[2]	118.99(8)	C5	C80	P2	133.1(11)
O25	U2	O35	126.54(10)	C5	C80	C75	22.7(7)
O25	U2	N56	63.34(11)	C5	C80	C79	104.0(11)
O25	U2	O62[2]	154.56(10)	C5	C80	C7	101.9(10)
O25	U2	O62	83.35(11)	C9	C80	P2	94(2)
O35	U2	U2[2]	114.46(7)	C9	C80	C75	112(3)

O35	U2	N56	63.20(10)	C9	C80	C79	65(2)
N56	U2	U2^{2}	177.62(8)	C9	C80	C7	34(2)
O62	U2	U2^{2}	35.66(7)	C9	C80	C5	91(2)
O62^{2}	U2	U2^{2}	35.63(7)	C80	C75	C1	100.6(10)
O62^{2}	U2	O35	78.87(10)	C80	C75	C9	17.3(6)
O62	U2	O35	150.04(10)	C76	C75	C80	120.2(11)
O62	U2	N56	146.64(11)	C76	C75	C1	24.9(6)
O62^{2}	U2	N56	142.05(11)	C76	C75	C9	112.5(10)
O62	U2	O62^{2}	71.30(12)	C5	C75	C80	65(2)
N3	C2	S1	119.5(4)	C5	C75	C76	69(2)
N1	C2	S1	124.5(4)	C5	C75	C1	44(2)
N1	C2	N3	115.6(5)	C5	C75	C9	50(2)
C4	N3	C2	117.3(5)	C9	C75	C1	89.5(9)
N3	C4	C41	117.6(4)	C75	C76	C77	119.3(10)
O5	C4	N3	127.0(4)	C75	C76	C3	109.6(9)
O5	C4	C41	115.4(4)	C77	C76	C3	29.4(6)
C4	O5	U1	128.0(3)	C1	C76	C75	104.2(16)
C7B	C8	O10	124.8(10)	C1	C76	C77	45.5(12)
O10	C8	C7A	117.6(8)	C1	C76	C3	16.2(13)
C8	C7A	N1	107.1(9)	C1	C76	C5	81.4(15)
C10	C9A	N1	103.5(9)	C5	C76	C75	22.8(6)
C8	C7B	N1	103.0(11)	C5	C76	C77	102.7(10)
C10	C9B	N1	109.5(11)	C5	C76	C3	87.6(9)
C9B	C10	O10	122.8(10)	C80	C79	C78	119.3(10)
O10	C10	C9A	123.4(8)	C80	C79	C6	122.0(10)
C10	O10	C8	113.2(6)	C78	C79	C6	43.2(7)
N13	C12	S11	120.2(5)	C7	C79	C80	99.2(12)
N16	C12	S11	123.7(4)	C7	C79	C78	98.9(11)
N16	C12	N13	115.9(6)	C7	C79	C6	55.6(10)
C14	N13	C12	119.2(4)	C7	C79	C9	76.8(11)
N13	C14	O15	126.9(4)	C9	C79	C80	22.5(6)
N13	C14	C45	117.6(4)	C9	C79	C78	120.7(9)
O15	C14	C45	115.4(4)	C9	C79	C6	106.1(10)
C14	O15	U1	128.0(3)	C78	C77	C76	119.8(9)
C12	N16	C17	122.5(6)	C3	C77	C76	109.7(13)
C12	N16	C19	125.2(5)	C3	C77	C78	68.5(11)
C19	N16	C17	112.2(6)	C1	C77	C76	31.4(9)
N16	C17	C18	108.2(6)	C1	C77	C78	109.9(11)
O20	C18	C17	114.0(7)	C1	C77	C3	78.3(14)
N16	C19	C20	110.1(6)	C79	C78	C7	30.6(5)
O20	C20	C19	112.1(6)	C79	C78	C3	117.6(10)
C18	O20	C20	111.2(6)	C79	C78	C1	95.1(7)
N23	C22	S21	119.0(4)	C77	C78	C79	120.9(9)
N26	C22	S21	124.3(4)	C77	C78	C7	123.2(8)
N26	C22	N23	116.5(5)	C77	C78	C3	44.7(9)
C24	N23	C22	117.0(4)	C77	C78	C1	29.3(6)
N23	C24	O25	127.1(4)	C6	C78	C79	81.2(10)
N23	C24	C51	117.7(4)	C6	C78	C77	107.2(12)
O25	C24	C51	115.1(4)	C6	C78	C7	50.6(8)

C24	O25	U2	128.1(3)
C22	N26	C27	124.7(5)
C22	N26	C29	123.9(5)
C29	N26	C27	111.3(5)
N26	C27	C28	110.3(6)
O30	C28	C27	111.3(6)
N26	C29	C30	109.4(6)
O30	C30	C29	111.4(6)
C30	O30	C28	110.0(5)
N33	C32	S31	120.5(4)
N36	C32	S31	124.2(4)
N36	C32	N33	115.2(5)
C34	N33	C32	116.9(4)
N33	C34	C55	118.2(4)
O35	C34	N33	126.4(4)
O35	C34	C55	115.3(4)
C34	O35	U2	127.9(3)
C32	N36	C37	122.3(6)
C32	N36	C39	124.7(5)
C37	N36	C39	112.7(5)
C38	C37	N36	111.0(6)
O40	C38	C37	112.4(6)
C40	C39	N36	106.9(6)
O40	C40	C39	112.2(6)
C38	O40	C40	108.1(5)
C42	C41	C4	125.5(4)
N46	C41	C4	112.7(4)
N46	C41	C42	121.7(4)
C41	C42	C43	118.3(4)
C44	C43	C42	120.2(4)
C43	C44	C45	118.2(4)
C44	C45	C14	125.0(4)
N46	C45	C14	112.9(4)
N46	C45	C44	122.0(4)
C41	N46	U1	120.3(3)
C45	N46	U1	120.2(3)
C45	N46	C41	119.4(4)
C52	C51	C24	125.5(4)
N56	C51	C24	113.0(4)
N56	C51	C52	121.5(4)
C53	C52	C51	118.4(4)
C52	C53	C54	120.4(4)
C53	C54	C55	117.9(4)
C54	C55	C34	124.9(4)
N56	C55	C34	113.2(4)
N56	C55	C54	121.8(4)
C51	N56	U2	119.9(3)
C55	N56	U2	120.1(3)
C55	N56	C51	119.8(4)

C6	C78	C3	63.1(12)
C6	C78	C1	90.9(11)
C7	C78	C1	94.0(7)
C3	C78	C7	97.0(9)
C3	C78	C1	41.6(9)
C2	N1	C7A	123.8(7)
C2	N1	C9A	124.4(7)
C2	N1	C7B	111.4(7)
C2	N1	C9B	115.9(7)
C7A	N1	C9A	106.0(8)
C9B	N1	C7B	101.2(10)
C78	C6	C79	55.6(8)
C78	C6	C7	88.9(10)
C78	C6	C3	67.2(12)
C7	C6	C79	33.4(6)
C3	C6	C79	103.8(12)
C3	C6	C7	122.8(15)
C79	C7	C80	50.3(9)
C79	C7	C78	50.5(9)
C79	C7	C6	91.0(12)
C79	C7	C9	62.7(10)
C78	C7	C80	84.6(8)
C6	C7	C80	115.2(12)
C6	C7	C78	40.4(7)
C9	C7	C80	12.6(7)
C9	C7	C78	92.7(9)
C9	C7	C6	117.2(13)
C77	C3	C76	41.0(10)
C77	C3	C78	66.7(9)
C77	C3	C6	115.8(13)
C77	C3	C1	50.1(11)
C78	C3	C76	87.7(9)
C6	C3	C76	120.0(10)
C6	C3	C78	49.8(9)
C1	C3	C76	9.2(7)
C1	C3	C78	93.9(11)
C1	C3	C6	120.0(12)
C75	C1	C78	96.3(7)
C76	C1	C75	50.9(13)
C76	C1	C77	103.1(17)
C76	C1	C78	116.2(14)
C76	C1	C3	154.6(19)
C76	C1	C5	66.3(14)
C77	C1	C75	119.3(11)
C77	C1	C78	40.8(8)
C77	C1	C3	51.6(11)
C77	C1	C5	120.1(12)
C3	C1	C75	133.8(11)
C3	C1	C78	44.6(7)

U1	O61	U1[1]	108.61(12)	C3	C1	C5	120.5(13)
C62	O61	U1[1]	117.5(2)	C5	C1	C75	15.5(7)
C62	O61	U1	117.2(2)	C5	C1	C78	88.9(8)
U2	O62	U2[2]	108.70(11)	C80	C5	C76	140.6(12)
C64	O62	U2	117.9(2)	C80	C5	C1	130.1(13)
C64	O62	U2[2]	119.1(2)	C80	C5	C9	22.7(6)
C86	P1	C71	107.2(2)	C75	C5	C80	92(3)
C96	P1	C71	113.0(2)	C75	C5	C76	89(3)
C96	P1	C86	108.9(2)	C75	C5	C1	121(3)
C106	P1	C71	110.1(2)	C75	C5	C9	113(3)
C106	P1	C86	109.9(2)	C76	C5	C1	32.3(7)
C106	P1	C96	107.8(2)	C76	C5	C9	143.9(12)
C73	P2	C9	107.4(4)	C9	C5	C1	119.1(12)
C126	P2	C73	110.1(2)	C80	C9	P2	70(2)
C126	P2	C9	116.0(4)	C80	C9	C75	51(2)
C136	P2	C73	110.9(2)	C80	C9	C79	93(2)
C136	P2	C126	107.8(2)	C80	C9	C7	133(3)
C136	P2	C9	104.6(4)	C80	C9	C5	67(2)
C80	P2	C73	109.6(5)	C75	C9	P2	100.9(7)
C80	P2	C126	100.6(4)	C79	C9	P2	120.6(8)
C80	P2	C136	117.3(4)	C79	C9	C75	110.5(10)
C80	P2	C9	16.6(4)	C79	C9	C7	40.5(7)
C72	C71	P1	117.4(3)	C79	C9	C5	104.9(10)
C74	C73	P2	116.4(3)	C7	C9	P2	122.9(9)
C86	C81	C82	119.3(5)	C7	C9	C75	135.1(11)
C83	C82	C81	120.6(5)	C7	C9	C5	120.2(11)
C82	C83	C84	119.8(5)	C5	C9	P2	116.8(9)
C85	C84	C83	120.7(5)	C5	C9	C75	17.4(6)
C84	C85	C86	119.3(5)				

[1]1-x,2-y,1-z; [2]1-x,1-y,2-z

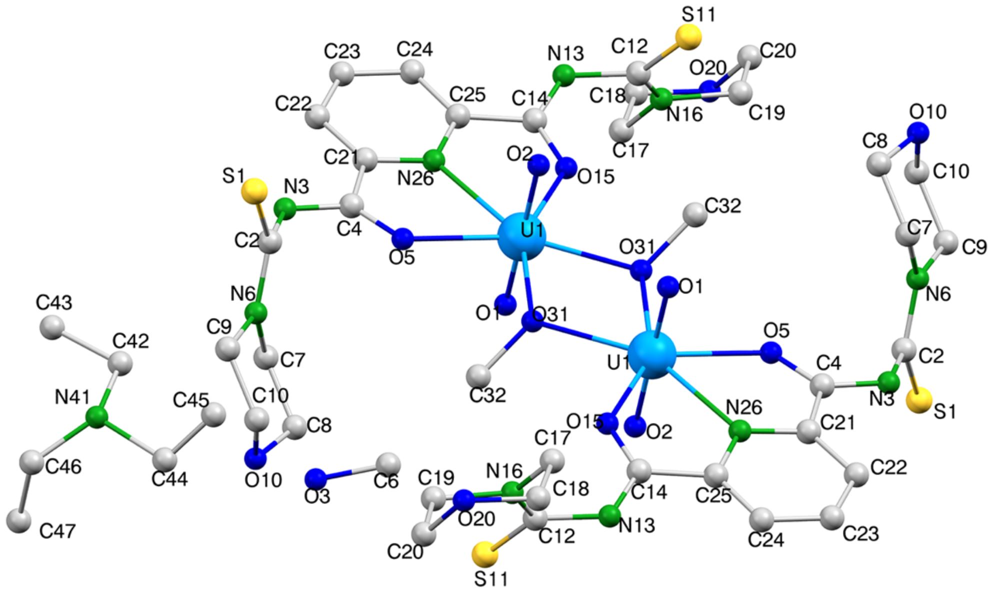

Figure S12. Representation of the structure of $(HNEt_3)_2$*anti,anti*-[{UO_2(L^{morph})(μ-OMe)}$_2$] x MeOH, $(HNEt_3)_2$*anti,anti*-[**8**]. The thermal ellipsoids are set at a 30% probability level. Hydrogen atoms are omitted for clarity.

Table S25. Bond lengths (Å) of $(HNEt_3)_2$*anti,anti*-[{UO_2(L^{morph})(μ-OMe)}$_2$], $(HNEt_3)_2$*anti,anti*-[**8**].

U1	U1[1]	3.817(2)	N13	C14	1.29(3)
U1	O1	1.739(18)	C14	O15	1.29(3)
U1	O2	1.80(2)	C14	C25	1.47(3)
U1	O5	2.369(17)	N16	C17	1.40(5)
U1	O15	2.354(16)	N16	C19	1.54(4)
U1	N26	2.536(19)	C17	C18	1.53(5)
U1	O31	2.337(16)	C18	O20	1.45(4)
U1	O31[1]	2.353(14)	C19	C20	1.51(6)
S1	C2	1.62(4)	C20	O20	1.41(5)
C2	N3	1.45(4)	C21	C22	1.41(3)
C2	N6	1.38(4)	C21	N26	1.34(3)
N3	C4	1.31(3)	C22	C23	1.33(4)
C4	O5	1.26(3)	C23	C24	1.37(4)
C4	C21	1.46(4)	C24	C25	1.38(3)
N6	C7	1.51(5)	C25	N26	1.36(3)
N6	C9	1.45(4)	O31	C32	1.47(3)
C7	C8	1.46(6)	N41	C42	1.47(7)
C8	O10	1.38(5)	N41	C44	1.66(10)
C9	C10	1.45(6)	N41	C46	1.51(2)
C10	O10	1.48(5)	C42	C43	1.53(11)
S11	C12	1.67(3)	C44	C45	1.34(12)
C12	N13	1.41(3)	C46	C47	1.50(2)
C12	N16	1.34(4)	O3	C6	1.40(8)

[1]1-x,-y,1-z

Table S26. Bond angles (°) in $(HNEt_3)_2$*anti,anti*-[{UO_2(L^{morph})(μ-OMe)}$_2$], $(HNEt_3)_2$*anti,anti*-[**8**].

O1	U1	U1[1]	90.2(6)	N6	C9	C10	112(4)
O1	U1	O2	178.5(8)	C9	C10	O10	111(4)
O1	U1	O5	91.2(8)	C8	O10	C10	114(3)
O1	U1	O15	89.0(7)	N13	C12	S11	122(2)
O1	U1	N26	88.9(7)	N16	C12	S11	126(2)
O1	U1	O31	89.1(7)	N16	C12	N13	112(3)
O1	U1	O31[1]	91.2(7)	C14	N13	C12	119(2)
O2	U1	U1[1]	91.0(7)	N13	C14	C25	119(2)
O2	U1	O5	89.1(9)	O15	C14	N13	127(2)
O2	U1	O15	89.6(9)	O15	C14	C25	114.0(18)
O2	U1	N26	90.0(8)	C14	O15	U1	129.0(13)
O2	U1	O31	92.4(8)	C12	N16	C17	128(3)
O2	U1	O31[1]	89.2(8)	C12	N16	C19	119(3)
O5	U1	U1[1]	116.7(4)	C17	N16	C19	113(3)
O5	U1	N26	62.7(6)	N16	C17	C18	110(3)
O15	U1	U1[1]	117.6(4)	O20	C18	C17	113(3)
O15	U1	O5	125.7(6)	C20	C19	N16	111(3)
O15	U1	N26	63.0(6)	O20	C20	C19	111(3)
N26	U1	U1[1]	178.9(4)	C20	O20	C18	111(3)
O31[1]	U1	U1[1]	35.4(4)	C22	C21	C4	129(2)
O31	U1	U1[1]	35.7(4)	N26	C21	C4	111(2)
O31[1]	U1	O5	152.0(6)	N26	C21	C22	120(2)
O31	U1	O5	81.1(6)	C23	C22	C21	119(2)
O31[1]	U1	O15	82.2(5)	C22	C23	C24	122(2)
O31	U1	O15	153.2(6)	C23	C24	C25	119(3)
O31	U1	N26	143.7(5)	C24	C25	C14	125(2)
O31[1]	U1	N26	145.3(6)	N26	C25	C14	114(2)
O31	U1	O31[1]	71.1(6)	N26	C25	C24	121(2)
N3	C2	S1	119(2)	C21	N26	U1	121.1(15)
N6	C2	S1	127(2)	C21	N26	C25	120(2)
N6	C2	N3	113(3)	C25	N26	U1	119.1(15)
C4	N3	C2	118(2)	U1	O31	U1[1]	108.9(6)
N3	C4	C21	115(2)	C32	O31	U1	116.2(16)
O5	C4	N3	127(2)	C32	O31	U1[1]	118.3(16)
O5	C4	C21	118(2)	C42	N41	C44	112(5)
C4	O5	U1	126.5(14)	C42	N41	C46	136(6)
C2	N6	C7	126(3)	C46	N41	C44	108(5)
C2	N6	C9	118(3)	N41	C42	C43	105(6)
C9	N6	C7	116(3)	C45	C44	N41	111(6)
C8	C7	N6	114(3)	C47	C46	N41	118(4)
O10	C8	C7	109(3)				

[1]1-x,-y,1-z

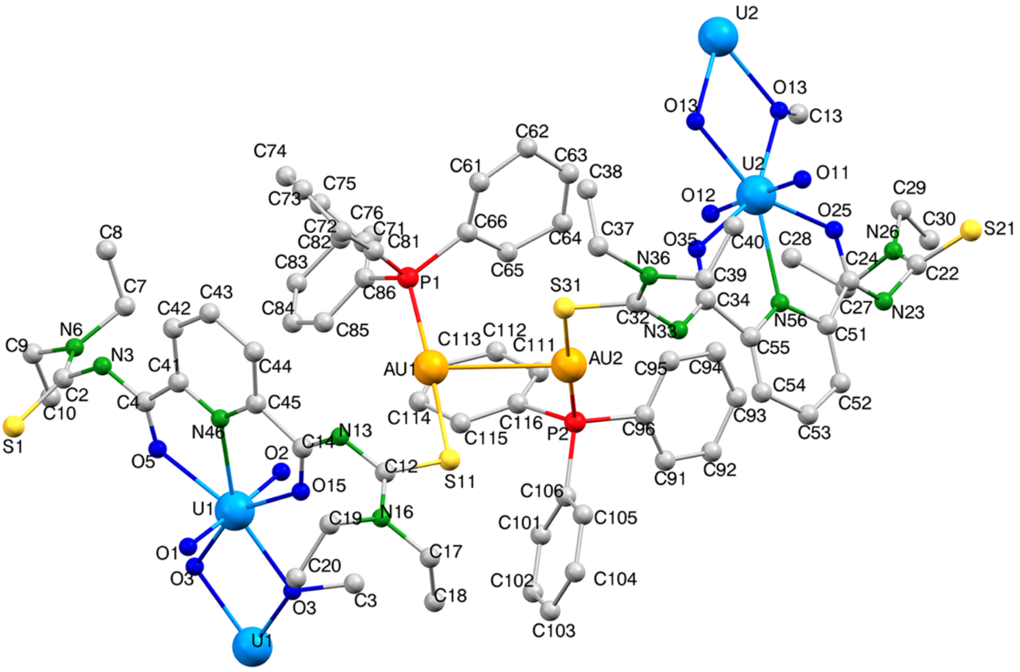

Figure S13. Representation of the structure of $[\{UO_2(L^{Et2})(\mu\text{-}OMe)\}_2\{Au(PPh_3)\}_2]$ (**2**). The thermal ellipsoids are set at a 30% probability level. Hydrogen atoms are omitted for clarity.

Table S27. Bond lengths (Å) of $[\{UO_2(L^{Et2})(\mu\text{-}OMe)\}_2\{Au(PPh_3)\}_2]$ (**2**).

U1	U1[1]	3.8201(13)	N33	C34	1.26(3)
U1	O1	1.775(10)	C34	O35	1.27(3)
U1	O2	1.762(10)	C34	C55	1.50(4)
U1	O5	2.378(11)	N36	C37	1.62(4)
U1	O15	2.395(10)	N36	C39	1.46(4)
U1	N46	2.528(13)	C37	C38	1.42(5)
U1	O3	2.324(12)	C39	C40	1.17(5)
U1	O3[1]	2.372(10)	C41	C42	1.40(2)
U2	U2[2]	3.7913(17)	C41	N46	1.334(19)
U2	O11	1.739(16)	C42	C43	1.35(2)
U2	O12	1.762(12)	C43	C44	1.36(3)
U2	O25	2.368(17)	C44	C45	1.43(2)
U2	O35	2.390(15)	C45	N46	1.35(2)
U2	N56	2.59(2)	C51	C52	1.44(4)
U2	O13	2.324(13)	C51	N56	1.35(3)
U2	O13[2]	2.343(13)	C52	C53	1.34(5)
Au2	Au1	3.2099(13)	C53	C54	1.37(4)
Au2	P2	2.242(7)	C54	C55	1.31(3)
Au2	S31	2.318(6)	C55	N56	1.40(3)
Au1	P1	2.270(5)	C61	C62	1.44(4)
Au1	S11	2.334(5)	C61	C66	1.29(3)
P1	C66	1.75(2)	C62	C63	1.33(4)
P1	C76	1.76(2)	C63	C64	1.32(3)

P1	C86	1.846(19)	C64	C65	1.43(3)
P2	C96	1.77(2)	C65	C66	1.37(3)
P2	C106	1.778(16)	C71	C72	1.31(4)
P2	C116	1.78(3)	C71	C76	1.36(3)
S1	C2	1.67(2)	C72	C73	1.38(4)
C2	N3	1.351(19)	C73	C74	1.35(4)
C2	N6	1.35(2)	C74	C75	1.38(4)
N3	C4	1.319(19)	C75	C76	1.37(3)
C4	O5	1.255(17)	C81	C82	1.37(2)
C4	C41	1.47(2)	C81	C86	1.38(3)
N6	C7	1.46(2)	C82	C83	1.42(3)
N6	C9	1.50(2)	C83	C84	1.33(3)
C7	C8	1.52(3)	C84	C85	1.34(2)
C9	C10	1.50(3)	C85	C86	1.42(2)
S11	C12	1.69(2)	C91	C92	1.3900
C12	N13	1.40(2)	C91	C96	1.3900
C12	N16	1.36(2)	C92	C93	1.3900
N13	C14	1.29(2)	C93	C94	1.3900
C14	O15	1.245(19)	C94	C95	1.3900
C14	C45	1.54(2)	C95	C96	1.3900
N16	C17	1.43(3)	O3	U1^{1}	2.372(10)
N16	C19	1.51(3)	O3	C3	1.42(2)
C17	C18	1.46(3)	C101	C102	1.3900
C19	C20	1.46(3)	C101	C106	1.3900
S21	C22	1.67(3)	C102	C103	1.3900
C22	N23	1.35(3)	C103	C104	1.3900
C22	N26	1.31(6)	C104	C105	1.3900
N23	C24	1.33(2)	C105	C106	1.3900
C24	O25	1.26(2)	O13	U2^{2}	2.343(13)
C24	C51	1.48(3)	O13	C13	1.39(2)
N26	C27	1.46(3)	C111	C112	1.412(17)
N26	C29	1.51(3)	C111	C116	1.403(16)
C27	C28	1.53(4)	C112	C113	1.413(17)
C29	C30	1.51(4)	C113	C114	1.418(16)
S31	C32	1.72(2)	C114	C115	1.425(16)
C32	N33	1.32(3)	C115	C116	1.420(17)
C32	N36	1.38(3)			

^{1}-x,1-y,1-z; ^{2}-x,1-y,-z

Table S28. Bond angles (°) in [{UO$_2$(L^{Et2})(μ-OMe)}$_2${Au(PPh$_3$)}$_2$] (**2**).

O1	U1	U1^{1}	91.6(4)	N26	C22	N23	113(4)
O1	U1	O5	91.0(5)	C24	N23	C22	121(3)
O1	U1	O15	89.2(4)	N23	C24	C51	116(3)
O1	U1	N46	89.3(5)	O25	C24	N23	124(3)
O1	U1	O3^{1}	89.0(4)	O25	C24	C51	120(3)
O1	U1	O3	93.7(5)	C24	O25	U2	124.8(18)

O2	U1	U1[1]	91.5(4)	C22	N26	C27	122(5)
O2	U1	O1	176.9(5)	C22	N26	C29	123(6)
O2	U1	O5	88.2(5)	C27	N26	C29	114(6)
O2	U1	O15	88.8(5)	N26	C27	C28	116(6)
O2	U1	N46	87.6(5)	C30	C29	N26	83(8)
O2	U1	O3[1]	93.8(5)	C32	S31	Au2	100.1(10)
O2	U1	O3	88.5(5)	N33	C32	S31	124.1(19)
O5	U1	U1[1]	115.1(3)	N33	C32	N36	119(2)
O5	U1	O15	125.3(4)	N36	C32	S31	116(2)
O5	U1	N46	62.0(4)	C34	N33	C32	118(2)
O15	U1	U1[1]	119.7(3)	N33	C34	O35	127(3)
O15	U1	N46	63.2(4)	N33	C34	C55	112(2)
N46	U1	U1[1]	177.0(3)	O35	C34	C55	121(2)
O3[1]	U1	U1[1]	35.2(3)	C34	O35	U2	125.6(16)
O3	U1	U1[1]	36.0(3)	C32	N36	C37	119(2)
O3	U1	O5	150.7(4)	C32	N36	C39	121(3)
O3[1]	U1	O5	80.1(4)	C39	N36	C37	120(2)
O3	U1	O15	83.8(4)	C38	C37	N36	117(3)
O3[1]	U1	O15	154.6(4)	C40	C39	N36	123(5)
O3[1]	U1	N46	142.0(4)	C42	C41	C4	127.3(15)
O3	U1	N46	146.8(4)	N46	C41	C4	111.7(14)
O3	U1	O3[1]	71.1(4)	N46	C41	C42	120.7(15)
O11	U2	U2[2]	90.4(5)	C43	C42	C41	122.0(17)
O11	U2	O12	178.8(7)	C42	C43	C44	119.8(19)
O11	U2	O25	91.0(8)	C43	C44	C45	115.8(18)
O11	U2	O35	88.6(7)	C44	C45	C14	123.8(17)
O11	U2	N56	90.4(7)	N46	C45	C14	111.6(14)
O11	U2	O13	93.2(6)	N46	C45	C44	124.6(17)
O11	U2	O13[2]	87.4(6)	C41	N46	U1	121.7(9)
O12	U2	U2[2]	90.6(5)	C41	N46	C45	117.0(14)
O12	U2	O25	87.9(8)	C45	N46	U1	121.2(11)
O12	U2	O35	91.6(7)	C52	C51	C24	128(3)
O12	U2	N56	88.7(8)	N56	C51	C24	112(3)
O12	U2	O13	87.2(6)	N56	C51	C52	119(3)
O12	U2	O13[2]	93.8(6)	C53	C52	C51	115(3)
O25	U2	U2[2]	116.9(5)	C52	C53	C54	124(3)
O25	U2	O35	127.5(6)	C55	C54	C53	120(3)
O25	U2	N56	63.9(7)	C54	C55	C34	133(3)
O35	U2	U2[2]	115.7(4)	C54	C55	N56	118(3)
O35	U2	N56	63.6(7)	N56	C55	C34	109(2)
N56	U2	U2[2]	178.9(5)	C51	N56	U2	118(2)
O13	U2	U2[2]	35.9(3)	C51	N56	C55	122(2)
O13[2]	U2	U2[2]	35.5(3)	C55	N56	U2	119.8(18)
O13	U2	O25	81.1(6)	C66	C61	C62	124(3)
O13[2]	U2	O25	152.2(5)	C63	C62	C61	117(3)
O13[2]	U2	O35	80.2(5)	C64	C63	C62	124(3)
O13	U2	O35	151.4(5)	C63	C64	C65	114(3)
O13[2]	U2	N56	143.8(7)	C66	C65	C64	126(2)
O13	U2	N56	144.8(7)	C61	C66	P1	128(2)

O13	U2	O13[2]	71.4(5)	C61	C66	C65	114(2)
P2	Au2	Au1	96.81(19)	C65	C66	P1	116.5(16)
P2	Au2	S31	178.6(3)	C72	C71	C76	128(3)
S31	Au2	Au1	83.91(16)	C71	C72	C73	115(3)
P1	Au1	Au2	100.86(15)	C74	C73	C72	125(3)
P1	Au1	S11	176.6(2)	C73	C74	C75	114(3)
S11	Au1	Au2	81.97(14)	C76	C75	C74	126(2)
C66	P1	Au1	115.1(7)	C71	C76	P1	121.6(18)
C66	P1	C76	105.8(10)	C71	C76	C75	112(2)
C66	P1	C86	106.3(9)	C75	C76	P1	126.2(17)
C76	P1	Au1	109.5(6)	C82	C81	C86	119.8(19)
C76	P1	C86	106.8(9)	C81	C82	C83	120.2(19)
C86	P1	Au1	112.8(7)	C84	C83	C82	117.9(18)
C96	P2	Au2	112.7(8)	C83	C84	C85	124.5(19)
C96	P2	C106	107.2(10)	C84	C85	C86	118.0(19)
C96	P2	C116	105.5(14)	C81	C86	P1	122.0(15)
C106	P2	Au2	112.1(7)	C81	C86	C85	119.6(18)
C106	P2	C116	106.6(11)	C85	C86	P1	118.4(15)
C116	P2	Au2	112.3(9)	C92	C91	C96	120.0
N3	C2	S1	121.3(15)	C91	C92	C93	120.0
N6	C2	S1	122.9(14)	C94	C93	C92	120.0
N6	C2	N3	115.5(17)	C93	C94	C95	120.0
C4	N3	C2	123.0(15)	C96	C95	C94	120.0
N3	C4	C41	116.1(15)	C91	C96	P2	120.3(13)
O5	C4	N3	127.3(15)	C95	C96	P2	119.0(13)
O5	C4	C41	116.5(14)	C95	C96	C91	120.0
C4	O5	U1	127.9(10)	U1	O3	U1[1]	108.9(4)
C2	N6	C7	120.4(14)	C3	O3	U1[1]	118.9(10)
C2	N6	C9	121.6(17)	C3	O3	U1	117.2(10)
C7	N6	C9	118.1(16)	C102	C101	C106	120.0
N6	C7	C8	111(2)	C103	C102	C101	120.0
C10	C9	N6	112.1(18)	C102	C103	C104	120.0
C12	S11	Au1	100.5(6)	C105	C104	C103	120.0
N13	C12	S11	120.4(13)	C104	C105	C106	120.0
N16	C12	S11	122.5(14)	C101	C106	P2	121.2(12)
N16	C12	N13	116.8(17)	C105	C106	P2	118.7(12)
C14	N13	C12	116.4(15)	C105	C106	C101	120.0
N13	C14	C45	114.5(17)	U2	O13	U2[2]	108.6(5)
O15	C14	N13	129.8(16)	C13	O13	U2	120.9(12)
O15	C14	C45	115.7(13)	C13	O13	U2[2]	120.0(12)
C14	O15	U1	128.2(10)	C116	C111	C112	121(4)
C12	N16	C17	118.9(18)	C111	C112	C113	120(4)
C12	N16	C19	121.9(17)	C112	C113	C114	118(4)
C17	N16	C19	118.9(16)	C113	C114	C115	124(3)
N16	C17	C18	113(2)	C116	C115	C114	116(3)
C20	C19	N16	110(2)	C111	C116	P2	117(3)
N23	C22	S21	121(3)	C111	C116	C115	122(3)
N26	C22	S21	125(4)	C115	C116	P2	122(2)

[1]-x,1-y,1-z; [2]-x,1-y,-z

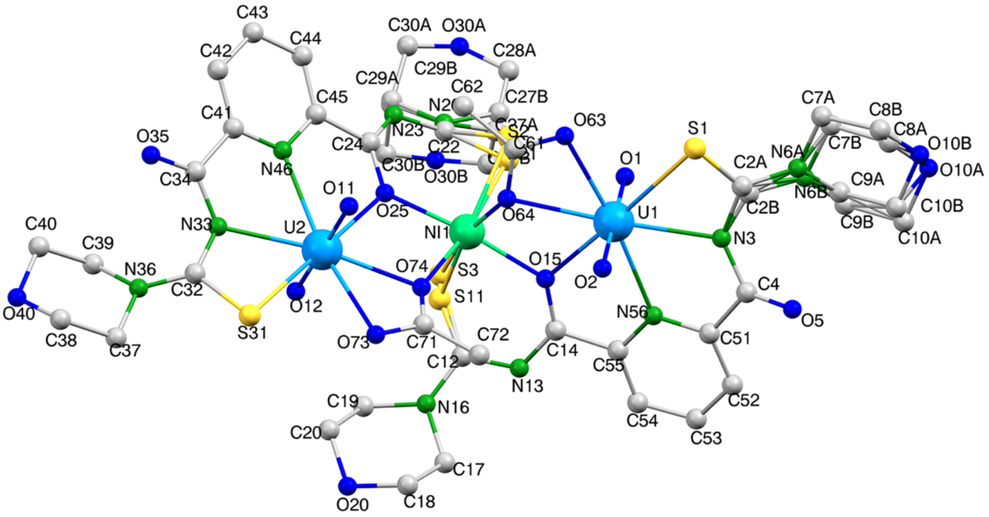

Figure S14. Ellipsoid representation of the structure of [Ni{UO2(L^{morph})(OAc)}2] (**6a**), also illustrating the disorderd parts of the molecule. The thermal ellipsoids are set at a 30% probability level. Hydrogen atoms are omitted for clarity.

Table S29. Bond lengths (Å) of [Ni{UO2(L^{morph})(OAc)}2] (**6a**).

U1	O1	1.775(8)	N16	C19	1.445(17)
U1	O2	1.772(8)	C17	C18	1.61(2)
U1	S1	2.868(3)	C18	O20	1.413(16)
U1	N3	2.469(10)	C19	C20	1.54(2)
U1	O15	2.486(8)	C20	O20	1.422(18)
U1	N56	2.569(10)	S21	C22	1.725(13)
U1	C61	2.876(11)	S21	S2	0.545(10)
U1	O63	2.483(8)	C22	N23	1.379(15)
U1	O64	2.479(7)	C22	N26	1.34(2)
U2	O11	1.774(8)	C22	S2	1.713(16)
U2	O12	1.769(8)	N23	C24	1.298(17)
U2	O25	2.503(8)	C24	O25	1.305(16)
U2	S31	2.876(3)	C24	C45	1.488(16)
U2	N33	2.460(8)	N26	C27A	1.468(16)
U2	N46	2.557(10)	N26	C29A	1.464(17)
U2	C71	2.896(12)	N26	C27B	1.469(18)
U2	O73	2.513(10)	N26	C29B	1.465(18)
U2	O74	2.456(7)	C27A	C28A	1.48(3)
Ni1	S11	2.427(5)	C28A	O30A	1.419(19)
Ni1	O15	2.078(8)	C29A	C30A	1.48(3)
Ni1	S21	2.268(5)	C30A	O30A	1.415(18)
Ni1	O25	2.067(7)	C27B	C28B	1.47(3)
Ni1	O64	2.079(7)	C28B	O30B	1.42(2)
Ni1	O74	2.068(9)	C29B	C30B	1.47(3)
Ni1	S2	2.585(10)	C30B	O30B	1.42(2)
Ni1	S3	2.400(11)	S31	C32	1.727(13)

S1	C2A	1.52(3)	C32	N33	1.359(16)
S1	C2B	1.88(3)	C32	N36	1.321(15)
N3	C4	1.362(17)	N33	C34	1.353(15)
N3	C2A	1.48(3)	C34	O35	1.232(14)
N3	C2B	1.26(3)	C34	C41	1.506(17)
C4	O5	1.230(16)	N36	C37	1.471(19)
C4	C51	1.484(18)	N36	C39	1.47(2)
C2A	N6A	1.34(2)	C37	C38	1.503(18)
N6A	C7A	1.46(2)	C38	O40	1.420(18)
N6A	C9A	1.46(2)	C39	C40	1.528(18)
C7A	C8A	1.64(2)	C40	O40	1.427(18)
C8A	O10A	1.45(2)	N46	C45	1.344(16)
C9A	C10A	1.62(2)	N46	C41	1.344(15)
C10A	O10A	1.44(2)	C45	C44	1.376(16)
C2B	N6B	1.34(2)	C44	C43	1.385(16)
N6B	C7B	1.46(2)	C43	C42	1.395(17)
N6B	C9B	1.46(2)	C42	C41	1.376(16)
C7B	C8B	1.63(2)	N56	C55	1.338(16)
C8B	O10B	1.44(2)	N56	C51	1.355(15)
C9B	C10B	1.64(2)	C55	C54	1.398(16)
C10B	O10B	1.45(2)	C54	C53	1.394(16)
S11	C12	1.527(14)	C53	C52	1.365(18)
S11	S3	0.633(9)	C52	C51	1.399(16)
C12	N13	1.371(16)	C61	C62	1.504(17)
C12	N16	1.370(17)	C61	O63	1.249(14)
C12	S3	2.128(17)	C61	O64	1.279(13)
N13	C14	1.297(16)	C71	C72	1.474(17)
C14	O15	1.288(15)	C71	O73	1.262(15)
C14	C55	1.485(16)	C71	O74	1.274(17)
N16	C17	1.49(2)			

Table S30. Bond angles (°) in [Ni{UO_2(L^{morph})(OAc)}$_2$] (**6a**).

O1	U1	S1	88.8(3)	O10B	C8B	C7B	88(2)
O1	U1	N3	87.3(4)	N6B	C9B	C10B	90(2)
O1	U1	O15	85.5(3)	O10B	C10B	C9B	122(3)
O1	U1	N56	94.7(4)	C8B	O10B	C10B	112(2)
O1	U1	C61	88.0(3)	C12	S11	Ni1	99.1(6)
O1	U1	O63	87.4(3)	S3	S11	Ni1	80.1(11)
O1	U1	O64	92.6(3)	S3	S11	C12	158.4(12)
O2	U1	O1	178.5(4)	S11	C12	S3	6.3(3)
O2	U1	S1	92.5(3)	N13	C12	S11	125.6(11)
O2	U1	N3	92.7(4)	N13	C12	S3	119.9(10)
O2	U1	O15	93.3(3)	N16	C12	S11	122.8(10)
O2	U1	N56	83.9(4)	N16	C12	N13	111.6(12)
O2	U1	C61	92.8(3)	N16	C12	S3	128.3(10)
O2	U1	O63	93.9(3)	C14	N13	C12	125.0(12)
O2	U1	O64	87.5(3)	N13	C14	C55	115.1(11)
S1	U1	C61	93.1(2)	O15	C14	N13	129.5(11)

N3	U1	S1	56.7(3)	O15	C14	C55	115.3(10)
N3	U1	O15	122.8(4)	Ni1	O15	U1	108.3(3)
N3	U1	N56	62.1(4)	C14	O15	U1	125.4(7)
N3	U1	C61	149.4(4)	C14	O15	Ni1	126.1(7)
N3	U1	O63	123.9(3)	C12	N16	C17	126.4(11)
N3	U1	O64	175.7(3)	C12	N16	C19	125.7(13)
O15	U1	S1	174.2(2)	C19	N16	C17	107.9(11)
O15	U1	N56	62.1(3)	N16	C17	C18	114.0(11)
O15	U1	C61	86.8(3)	O20	C18	C17	107.1(13)
N56	U1	S1	118.4(2)	N16	C19	C20	111.5(13)
N56	U1	C61	148.4(3)	O20	C20	C19	114.8(12)
O63	U1	S1	67.44(19)	C18	O20	C20	110.6(11)
O63	U1	O15	112.3(2)	C22	S21	Ni1	100.3(5)
O63	U1	N56	173.7(3)	S2	S21	Ni1	120.3(15)
O63	U1	C61	25.6(3)	S2	S21	C22	79.6(13)
O64	U1	S1	119.05(17)	N23	C22	S21	126.2(11)
O64	U1	O15	61.5(2)	N23	C22	S2	118.4(11)
O64	U1	N56	122.1(3)	N26	C22	S21	118.8(9)
O64	U1	C61	26.3(3)	N26	C22	N23	114.9(11)
O64	U1	O63	51.8(2)	N26	C22	S2	123.5(10)
O11	U2	O25	90.4(3)	S2	C22	S21	18.2(4)
O11	U2	S31	90.1(3)	C24	N23	C22	126.0(12)
O11	U2	N33	94.6(3)	N23	C24	O25	128.3(11)
O11	U2	N46	82.4(4)	N23	C24	C45	115.6(12)
O11	U2	C71	86.5(4)	O25	C24	C45	116.0(11)
O11	U2	O73	86.7(4)	Ni1	O25	U2	110.1(3)
O11	U2	O74	91.8(3)	C24	O25	U2	123.9(7)
O12	U2	O11	178.4(4)	C24	O25	Ni1	125.5(7)
O12	U2	O25	88.2(3)	C22	N26	C27A	124.3(16)
O12	U2	S31	91.3(3)	C22	N26	C29A	123(2)
O12	U2	N33	85.7(3)	C22	N26	C27B	121(2)
O12	U2	N46	96.4(4)	C22	N26	C29B	122(3)
O12	U2	C71	94.1(4)	C29A	N26	C27A	112(2)
O12	U2	O73	94.4(4)	C29B	N26	C27B	113(4)
O12	U2	O74	88.1(3)	N26	C27A	C28A	104.4(17)
O25	U2	S31	179.56(18)	O30A	C28A	C27A	111.2(19)
O25	U2	N46	62.8(3)	N26	C29A	C30A	104(2)
O25	U2	C71	87.2(3)	O30A	C30A	C29A	113(3)
O25	U2	O73	113.0(3)	C30A	O30A	C28A	113.0(19)
S31	U2	C71	92.8(3)	N26	C27B	C28B	92(2)
N33	U2	O25	123.3(3)	O30B	C28B	C27B	114(3)
N33	U2	S31	56.7(2)	N26	C29B	C30B	93(3)
N33	U2	N46	62.0(3)	O30B	C30B	C29B	110(4)
N33	U2	C71	149.4(3)	C28B	O30B	C30B	111(3)
N33	U2	O73	123.7(3)	C32	S31	U2	81.5(4)
N46	U2	S31	117.3(2)	N33	C32	S31	111.7(8)
N46	U2	C71	147.8(3)	N36	C32	S31	123.3(11)
O73	U2	S31	67.0(2)	N36	C32	N33	125.0(12)
O73	U2	N46	168.2(4)	C32	N33	U2	106.4(7)

O73	U2	C71	25.8(3)	C34	N33	U2	124.4(7)
O74	U2	O25	61.9(3)	C34	N33	C32	123.9(10)
O74	U2	S31	118.1(2)	N33	C34	C41	111.3(10)
O74	U2	N33	171.7(3)	O35	C34	N33	127.8(12)
O74	U2	N46	124.3(3)	O35	C34	C41	120.8(11)
O74	U2	C71	25.9(4)	C32	N36	C37	122.6(13)
O74	U2	O73	51.4(3)	C32	N36	C39	124.8(13)
S11	Ni1	S2	107.9(3)	C37	N36	C39	111.8(11)
O15	Ni1	S11	87.6(2)	N36	C37	C38	109.3(12)
O15	Ni1	S21	94.3(3)	O40	C38	C37	112.5(13)
O15	Ni1	O64	75.2(3)	N36	C39	C40	109.2(13)
O15	Ni1	S2	98.9(3)	O40	C40	C39	109.2(12)
O15	Ni1	S3	94.6(3)	C38	O40	C40	110.0(10)
S21	Ni1	S11	98.4(2)	C45	N46	U2	121.0(7)
S21	Ni1	S2	10.5(3)	C45	N46	C41	118.2(10)
S21	Ni1	S3	84.6(3)	C41	N46	U2	119.3(8)
O25	Ni1	S11	95.5(2)	N46	C45	C24	114.5(10)
O25	Ni1	O15	168.9(4)	N46	C45	C44	121.9(11)
O25	Ni1	S21	95.8(3)	C44	C45	C24	123.6(11)
O25	Ni1	O64	99.8(3)	C45	C44	C43	119.7(11)
O25	Ni1	O74	76.1(3)	C44	C43	C42	118.7(11)
O25	Ni1	S2	90.3(3)	C41	C42	C43	118.1(11)
O25	Ni1	S3	91.0(3)	N46	C41	C34	115.8(10)
O64	Ni1	S11	160.8(2)	N46	C41	C42	123.3(11)
O64	Ni1	S21	91.6(2)	C42	C41	C34	120.9(11)
O64	Ni1	S2	83.7(3)	C55	N56	U1	121.0(7)
O64	Ni1	S3	168.9(3)	C55	N56	C51	119.0(10)
O74	Ni1	S11	86.6(3)	C51	N56	U1	119.7(8)
O74	Ni1	O15	93.5(3)	N56	C55	C14	115.0(11)
O74	Ni1	S21	170.9(3)	N56	C55	C54	122.3(11)
O74	Ni1	O64	85.8(3)	C54	C55	C14	122.6(12)
O74	Ni1	S2	161.2(3)	C53	C54	C55	117.6(12)
O74	Ni1	S3	99.5(3)	C52	C53	C54	120.7(11)
S3	Ni1	S11	15.1(2)	C53	C52	C51	118.5(11)
S3	Ni1	S2	93.6(4)	N56	C51	C4	116.3(10)
C2A	S1	U1	84.6(10)	N56	C51	C52	121.6(12)
C2B	S1	U1	80.2(8)	C52	C51	C4	121.8(11)
C4	N3	U1	123.3(8)	C62	C61	U1	168.9(10)
C4	N3	C2A	128.6(15)	O63	C61	U1	59.3(6)
C2A	N3	U1	101.2(11)	O63	C61	C62	122.4(10)
C2B	N3	U1	111.2(14)	O63	C61	O64	118.0(10)
C2B	N3	C4	123.5(16)	O64	C61	U1	59.3(5)
N3	C4	C51	111.5(11)	O64	C61	C62	119.4(10)
O5	C4	N3	127.1(13)	C61	O63	U1	95.1(7)
O5	C4	C51	121.4(13)	Ni1	O64	U1	108.5(3)
N3	C2A	S1	116.9(15)	C61	O64	U1	94.4(6)
N6A	C2A	S1	123(2)	C61	O64	Ni1	138.2(8)
N6A	C2A	N3	118(2)	C72	C71	U2	170.8(10)
C2A	N6A	C7A	122(2)	O73	C71	U2	59.9(7)

C2A	N6A	C9A	126(2)	O73	C71	C72	122.5(14)
C9A	N6A	C7A	111(2)	O73	C71	O74	116.4(11)
N6A	C7A	C8A	96(2)	O74	C71	U2	57.4(5)
O10A	C8A	C7A	132(3)	O74	C71	C72	121.1(11)
N6A	C9A	C10A	129(3)	C71	O73	U2	94.3(8)
O10A	C10A	C9A	94(2)	Ni1	O74	U2	111.9(4)
C10A	O10A	C8A	111(2)	C71	O74	U2	96.7(7)
N3	C2B	S1	107.3(15)	C71	O74	Ni1	147.8(7)
N3	C2B	N6B	128(2)	S21	S2	Ni1	49.2(13)
N6B	C2B	S1	124(2)	S21	S2	C22	82.1(14)
C2B	N6B	C7B	122(2)	C22	S2	Ni1	89.4(6)
C2B	N6B	C9B	127(2)	S11	S3	Ni1	84.8(11)
C7B	N6B	C9B	111(2)	S11	S3	C12	15.3(8)
N6B	C7B	C8B	121(3)	C12	S3	Ni1	85.0(5)

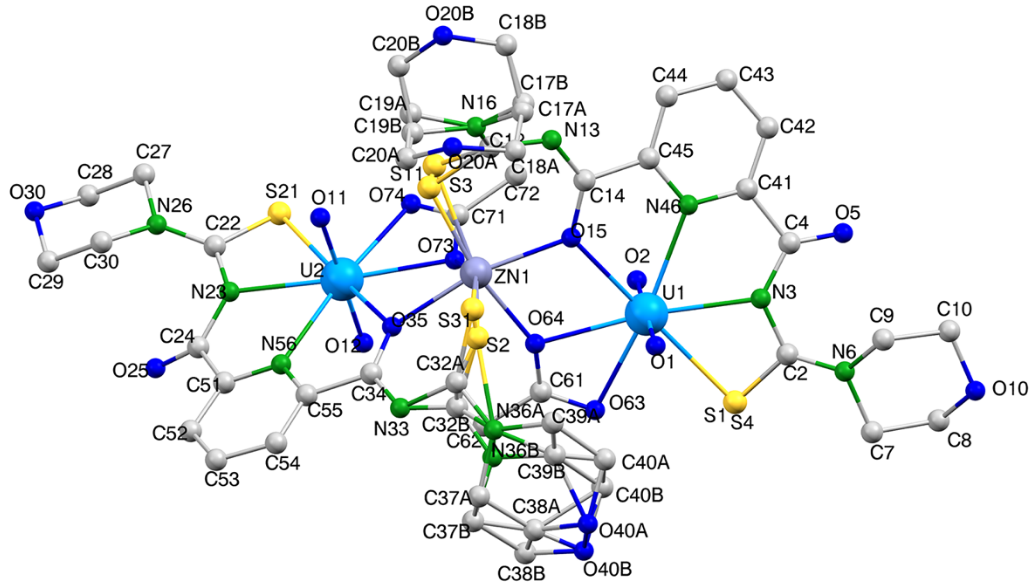

Figure S15. Ellipsoid representation of the structure of [Zn{UO_2(L^{morph})(OAc)}$_2$] (**6e**), also illustrating the disorderd parts of the molecule. The thermal ellipsoids are set at a 30% probability level. Hydrogen atoms are omitted for clarity.

Table S31. Bond lengths (Å) of [Zn{UO_2(L^{morph})(OAc)}$_2$] (**6e**).

U1	O1	1.765(6)	C20B	O20B	1.448(14)
U1	O2	1.772(6)	C20B	C19B	1.475(19)
U1	S1	2.929(4)	S21	C22	1.702(11)
U1	N3	2.465(6)	C22	N23	1.366(13)
U1	O15	2.486(5)	C22	N26	1.337(11)
U1	N46	2.556(6)	N23	C24	1.367(12)
U1	C61	2.876(9)	C24	O25	1.228(11)
U1	O63	2.503(7)	C24	C51	1.500(15)
U1	O64	2.465(5)	N26	C27	1.480(15)
U1	S4	2.792(9)	N26	C30	1.486(18)
U2	O11	1.773(6)	C27	C28	1.528(14)
U2	S21	2.869(2)	C28	O30	1.44(2)
U2	N23	2.473(7)	C29	C30	1.530(14)
U2	O35	2.478(5)	C29	O30	1.449(18)
U2	N56	2.572(7)	S31	C32A	1.663(18)
U2	C71	2.868(8)	S31	C32B	2.29(2)
U2	O73	2.473(5)	S31	S2	0.663(6)
U2	O74	2.481(6)	N33	C34	1.310(11)
U2	O12	1.774(6)	N33	C32A	1.519(19)
Zn1	S11	2.478(3)	N33	C32B	1.256(19)
Zn1	O15	2.173(5)	C34	O35	1.282(10)
Zn1	S31	2.432(3)	C34	C55	1.496(11)
Zn1	O35	2.181(5)	C32A	N36A	1.334(16)
Zn1	O64	2.107(6)	C32A	S2	1.073(18)
Zn1	O73	2.149(5)	N36A	C37A	1.483(14)
Zn1	S2	2.479(7)	N36A	C39A	1.479(13)

Zn1	S3	2.167(7)	N36A	S2	2.004(14)
S1	C2	1.769(9)	C37A	C38A	1.53(2)
C2	N3	1.365(10)	C38A	O40A	1.436(13)
C2	N6	1.326(9)	C39A	C40A	1.52(2)
C2	S4	1.614(11)	C40A	O40A	1.432(13)
N3	C4	1.349(9)	C32B	N36B	1.327(18)
C4	O5	1.229(9)	C32B	S2	1.67(2)
C4	C41	1.503(10)	N36B	C37B	1.484(15)
N6	C7	1.486(10)	N36B	C39B	1.478(14)
N6	C9	1.486(10)	C37B	C38B	1.55(3)
C7	C8	1.516(12)	C38B	O40B	1.431(14)
C8	O10	1.439(10)	C39B	C40B	1.53(3)
C9	C10	1.527(10)	C40B	O40B	1.444(14)
C10	O10	1.436(10)	N46	C45	1.348(9)
S11	C12	1.718(9)	N46	C41	1.353(9)
S11	S3	0.566(7)	C45	C44	1.385(12)
C12	N13	1.372(11)	C44	C43	1.391(12)
C12	N16	1.326(12)	C43	C42	1.392(11)
C12	S3	1.791(11)	C42	C41	1.387(10)
N13	C14	1.277(10)	N56	C55	1.342(12)
C14	O15	1.305(10)	N56	C51	1.351(11)
C14	C45	1.499(11)	C55	C54	1.395(12)
N16	C17A	1.479(13)	C54	C53	1.405(15)
N16	C19A	1.478(12)	C53	C52	1.388(17)
N16	C17B	1.482(14)	C52	C51	1.391(14)
N16	C19B	1.507(13)	C61	C62	1.504(13)
C17A	C18A	1.48(2)	C61	O63	1.253(11)
C18A	O20A	1.438(13)	C61	O64	1.266(13)
C20A	O20A	1.437(13)	C71	C72	1.508(12)
C20A	C19A	1.475(18)	C71	O73	1.267(9)
C17B	C18B	1.48(2)	C71	O74	1.258(10)
C18B	O20B	1.438(14)			

Table S32: Bond angles (°) in [Zn{UO_2(L^{morph})(OAc)}$_2$] (**6e**).

O1	U1	O2	178.6(3)	N13	C14	O15	130.0(8)
O1	U1	S1	93.0(2)	N13	C14	C45	115.0(8)
O1	U1	N3	85.4(2)	O15	C14	C45	115.0(7)
O1	U1	O15	88.3(2)	Zn1	O15	U1	110.9(2)
O1	U1	N46	96.5(2)	C14	O15	U1	125.3(5)
O1	U1	C61	93.7(3)	C14	O15	Zn1	123.1(5)
O1	U1	O63	93.7(3)	C12	N16	C17A	122.6(13)
O1	U1	O64	88.4(2)	C12	N16	C19A	122.7(9)
O1	U1	S4	86.4(2)	C12	N16	C17B	124.2(14)
O2	U1	S1	88.3(2)	C12	N16	C19B	125.1(11)
O2	U1	N3	94.8(2)	C19A	N16	C17A	113.6(15)
O2	U1	O15	90.4(2)	C17B	N16	C19B	108.6(17)
O2	U1	N46	82.3(2)	N16	C17A	C18A	98.8(13)

O2	U1	C61	86.8(3)	O20A	C18A	C17A	110.2(16)
O2	U1	O63	87.3(3)	O20A	C20A	C19A	112.6(13)
O2	U1	O64	91.5(2)	C20A	O20A	C18A	111.0(11)
O2	U1	S4	94.9(2)	C20A	C19A	N16	98.7(11)
N3	U1	S1	56.96(17)	C18B	C17B	N16	97.9(15)
N3	U1	O15	122.9(2)	O20B	C18B	C17B	113.4(19)
N3	U1	N46	61.9(2)	O20B	C20B	C19B	111.6(15)
N3	U1	C61	149.4(3)	C18B	O20B	C20B	110.5(13)
N3	U1	O63	123.7(2)	C20B	C19B	N16	97.7(12)
N3	U1	O64	171.91(19)	C22	S21	U2	82.6(3)
N3	U1	S4	55.1(2)	N23	C22	S21	112.5(6)
O15	U1	S1	178.64(14)	N26	C22	S21	123.6(9)
O15	U1	N46	62.65(19)	N26	C22	N23	123.4(11)
O15	U1	C61	87.5(2)	C22	N23	U2	106.2(6)
O15	U1	O63	113.3(2)	C22	N23	C24	125.7(8)
O15	U1	S4	174.44(19)	C24	N23	U2	123.0(5)
N46	U1	S1	116.87(16)	N23	C24	C51	110.4(8)
N46	U1	C61	148.0(2)	O25	C24	N23	127.9(11)
N46	U1	S4	116.5(2)	O25	C24	C51	121.8(9)
C61	U1	S1	92.7(2)	C22	N26	C27	120.7(11)
O63	U1	S1	66.92(18)	C22	N26	C30	125.0(10)
O63	U1	N46	168.8(2)	C27	N26	C30	114.0(9)
O63	U1	C61	25.8(3)	N26	C27	C28	107.5(11)
O63	U1	S4	68.6(2)	O30	C28	C27	112.4(14)
O64	U1	S1	118.36(18)	O30	C29	C30	109.8(10)
O64	U1	O15	61.9(2)	N26	C30	C29	108.6(12)
O64	U1	N46	124.1(2)	C28	O30	C29	111.3(9)
O64	U1	C61	26.0(3)	C32A	S31	Zn1	92.4(6)
O64	U1	O63	51.5(2)	C32B	S31	Zn1	85.9(5)
O64	U1	S4	119.4(2)	S2	S31	Zn1	86.3(7)
S4	U1	S1	6.79(17)	S2	S31	C32A	21.4(8)
S4	U1	C61	94.3(3)	S2	S31	C32B	17.0(7)
O11	U2	S21	88.12(19)	C34	N33	C32A	120.7(9)
O11	U2	N23	87.2(3)	C32B	N33	C34	128.7(11)
O11	U2	O35	85.5(2)	N33	C34	C55	115.2(8)
O11	U2	N56	95.1(3)	O35	C34	N33	128.7(8)
O11	U2	C71	88.4(2)	O35	C34	C55	116.0(7)
O11	U2	O73	92.0(2)	Zn1	O35	U2	108.1(2)
O11	U2	O74	88.1(2)	C34	O35	U2	125.9(5)
O11	U2	O12	177.9(3)	C34	O35	Zn1	126.0(5)
N23	U2	S21	56.6(2)	N33	C32A	S31	129.7(10)
N23	U2	O35	122.5(2)	N36A	C32A	S31	121.4(12)
N23	U2	N56	62.0(3)	N36A	C32A	N33	108.8(13)
N23	U2	C71	149.4(3)	S2	C32A	S31	13.0(5)
N23	U2	O73	175.5(2)	S2	C32A	N33	138.3(12)
N23	U2	O74	123.6(2)	S2	C32A	N36A	112.2(13)
O35	U2	S21	173.60(16)	C32A	N36A	C37A	123.8(12)
O35	U2	N56	62.1(2)	C32A	N36A	C39A	124.0(13)
O35	U2	C71	87.23(19)	C32A	N36A	S2	29.7(8)

O35	U2	O74	113.04(17)	C37A	N36A	S2	153.4(9)
N56	U2	S21	118.29(16)	C39A	N36A	C37A	112.0(11)
N56	U2	C71	148.6(2)	C39A	N36A	S2	94.6(8)
C71	U2	S21	92.97(16)	N36A	C37A	C38A	111.1(12)
O73	U2	S21	118.92(12)	O40A	C38A	C37A	108.6(13)
O73	U2	O35	61.79(16)	N36A	C39A	C40A	109.7(13)
O73	U2	N56	122.50(19)	O40A	C40A	C39A	110.9(11)
O73	U2	C71	26.11(19)	C40A	O40A	C38A	110.3(11)
O73	U2	O74	51.95(17)	N33	C32B	S31	104.7(12)
O74	U2	S21	67.03(13)	N33	C32B	N36B	125.3(17)
O74	U2	N56	173.82(19)	N33	C32B	S2	111.3(14)
O74	U2	C71	25.9(2)	N36B	C32B	S31	126.6(14)
O12	U2	S21	93.61(19)	N36B	C32B	S2	120.8(14)
O12	U2	N23	92.7(3)	S2	C32B	S31	6.7(3)
O12	U2	O35	92.8(2)	C32B	N36B	C37B	123.0(14)
O12	U2	N56	83.0(3)	C32B	N36B	C39B	123.5(15)
O12	U2	C71	92.8(2)	C39B	N36B	C37B	113.5(13)
O12	U2	O73	88.2(2)	N36B	C37B	C38B	110.5(14)
O12	U2	O74	93.7(2)	O40B	C38B	C37B	110.5(16)
S11	Zn1	S2	115.0(2)	N36B	C39B	C40B	108.9(16)
O15	Zn1	S11	90.40(17)	O40B	C40B	C39B	110.5(14)
O15	Zn1	S31	95.09(16)	C38B	O40B	C40B	111.3(12)
O15	Zn1	O35	164.9(2)	C45	N46	U1	121.0(5)
O15	Zn1	S2	98.0(2)	C45	N46	C41	117.9(6)
S31	Zn1	S11	99.97(13)	C41	N46	U1	119.4(5)
S31	Zn1	S2	15.48(14)	N46	C45	C14	114.2(7)
O35	Zn1	S11	102.53(18)	N46	C45	C44	122.5(7)
O35	Zn1	S31	90.47(17)	C44	C45	C14	123.3(7)
O35	Zn1	S2	83.8(2)	C45	C44	C43	119.3(7)
O64	Zn1	S11	157.09(18)	C44	C43	C42	118.7(7)
O64	Zn1	O15	73.1(2)	C41	C42	C43	118.7(7)
O64	Zn1	S31	97.25(18)	N46	C41	C4	115.5(6)
O64	Zn1	O35	92.3(2)	N46	C41	C42	122.9(7)
O64	Zn1	O73	83.0(2)	C42	C41	C4	121.6(7)
O64	Zn1	S2	83.6(2)	C55	N56	U2	121.5(5)
O64	Zn1	S3	168.3(3)	C55	N56	C51	118.4(8)
O73	Zn1	S11	85.13(16)	C51	N56	U2	119.9(7)
O73	Zn1	O15	101.83(19)	N56	C55	C34	113.9(7)
O73	Zn1	S31	162.32(16)	N56	C55	C54	123.5(8)
O73	Zn1	O35	71.9(2)	C54	C55	C34	122.7(9)
O73	Zn1	S2	151.6(2)	C55	C54	C53	117.1(10)
O73	Zn1	S3	93.1(2)	C52	C53	C54	120.1(9)
S3	Zn1	S11	11.7(2)	C53	C52	C51	118.4(9)
S3	Zn1	O15	97.1(2)	N56	C51	C24	116.2(8)
S3	Zn1	S31	89.8(2)	N56	C51	C52	122.4(10)
S3	Zn1	O35	97.0(2)	C52	C51	C24	121.4(9)
S3	Zn1	S2	104.4(3)	C62	C61	U1	171.3(7)
C2	S1	U1	79.9(3)	O63	C61	U1	60.3(5)
N3	C2	S1	111.7(5)	O63	C61	C62	121.8(11)

N3	C2	S4	110.3(6)	O63	C61	O64	118.0(8)
N6	C2	S1	124.2(6)	O64	C61	U1	58.6(4)
N6	C2	N3	124.0(7)	O64	C61	C62	120.1(8)
N6	C2	S4	124.1(7)	C61	O63	U1	94.0(6)
S4	C2	S1	11.2(4)	Zn1	O64	U1	114.0(3)
C2	N3	U1	106.8(4)	C61	O64	U1	95.4(5)
C4	N3	U1	124.4(5)	C61	O64	Zn1	147.7(5)
C4	N3	C2	123.1(6)	C72	C71	U2	170.3(8)
N3	C4	C41	111.6(6)	O73	C71	U2	59.2(4)
O5	C4	N3	128.4(7)	O73	C71	C72	119.5(7)
O5	C4	C41	119.9(7)	O74	C71	U2	59.6(4)
C2	N6	C7	121.8(7)	O74	C71	C72	121.9(7)
C2	N6	C9	125.0(7)	O74	C71	O73	118.4(7)
C9	N6	C7	112.4(6)	Zn1	O73	U2	109.4(2)
N6	C7	C8	108.3(7)	C71	O73	U2	94.7(4)
O10	C8	C7	111.2(7)	C71	O73	Zn1	136.6(5)
N6	C9	C10	108.1(7)	C71	O74	U2	94.5(5)
O10	C10	C9	109.9(7)	S31	S2	Zn1	78.2(7)
C10	O10	C8	110.6(6)	S31	S2	C32A	145.6(13)
C12	S11	Zn1	92.6(4)	S31	S2	N36A	154.8(9)
S3	S11	Zn1	51.1(9)	S31	S2	C32B	156.3(10)
S3	S11	C12	88.0(10)	C32A	S2	Zn1	108.9(10)
S11	C12	S3	18.4(2)	C32A	S2	N36A	38.0(8)
N13	C12	S11	120.1(7)	N36A	S2	Zn1	126.8(5)
N13	C12	S3	128.0(7)	C32B	S2	Zn1	100.0(7)
N16	C12	S11	122.7(6)	S11	S3	Zn1	117.2(10)
N16	C12	N13	115.8(8)	S11	S3	C12	73.5(9)
N16	C12	S3	115.8(7)	C12	S3	Zn1	101.8(4)
C14	N13	C12	126.0(9)	C2	S4	U1	86.7(4)

2. Selected Spectroscopic Data

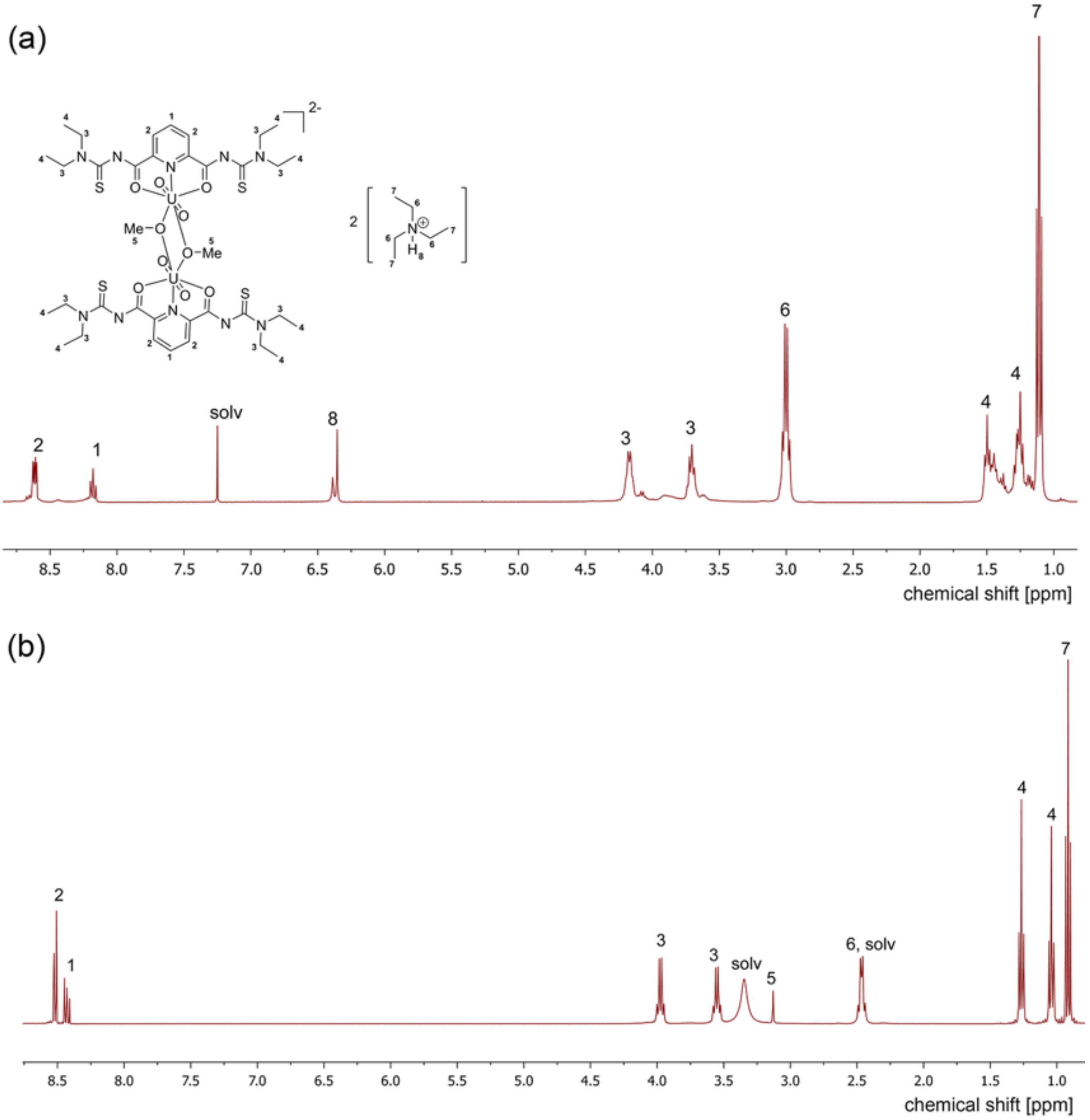

Figure S16: ^{1}H NMR spectra of $(HNEt_3)_2[\{UO_2(L^{Et2})(\mu\text{-}OMe)\}_2]$, $(HNEt_3)_2$[**1**]: (a) in $CDCl_3$ and (b) in DMSO.

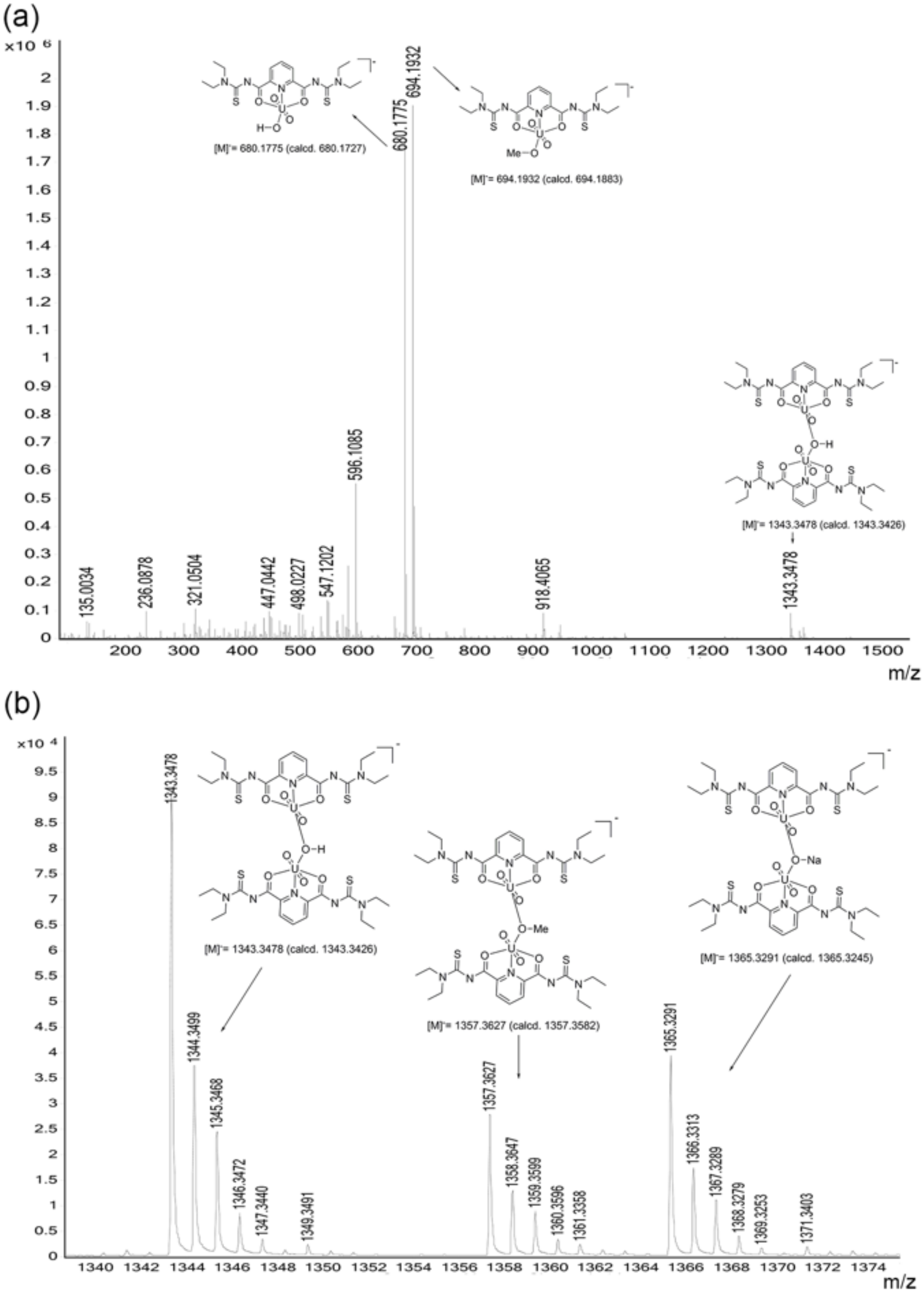

Figure S17: ESI(–) mass spectrum of $(HNEt_3)_2[\{UO_2(L^{Et2})(\mu\text{-}OMe)\}_2]$, $(HNEt_3)_2$[**1**]: (a) overview spectrum, (b) high-mass region with assignment..

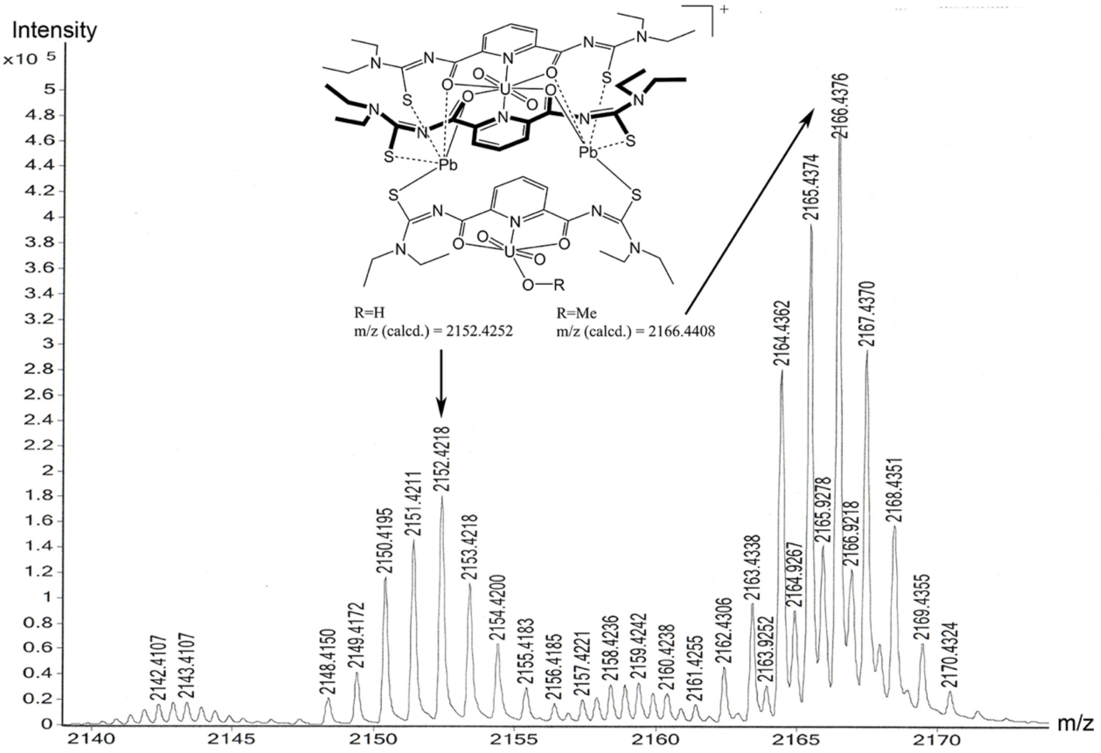

Figure S18: ESI(+) mass spectrum of $[Pb_2(UO_2)_3(L^{Et2})_3(\mu\text{-}OMe)_2(MeOH)_2]$ (**3**).

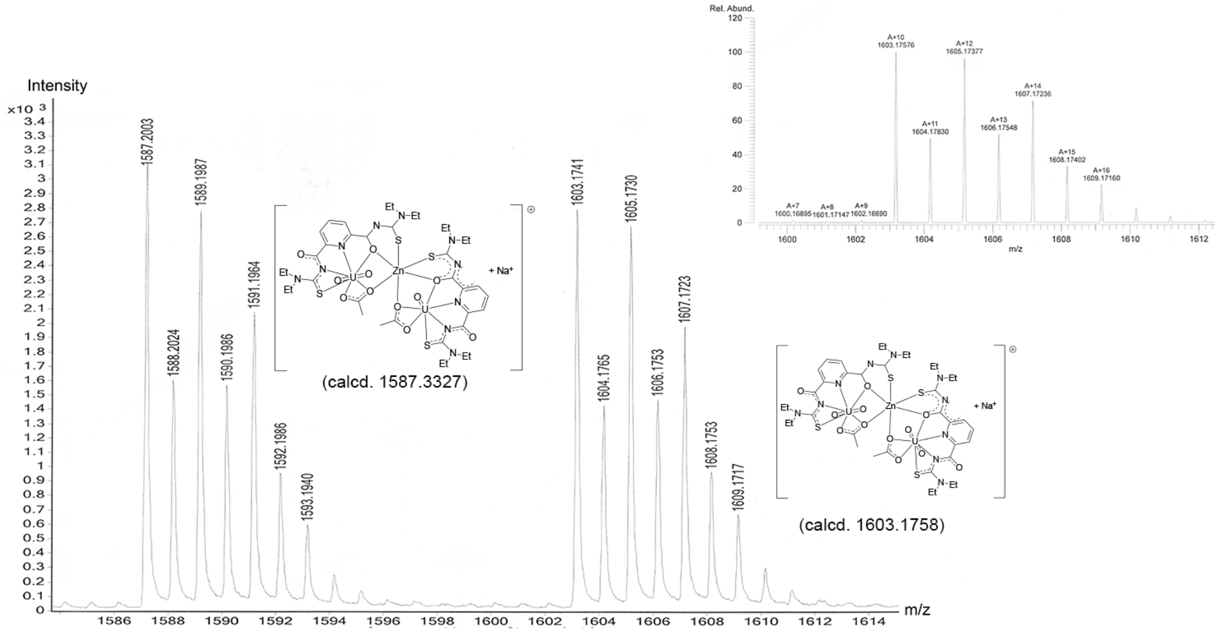

Figure S19: ESI(+) mass spectrum of $[Zn\{UO_2(L^{Et2})(OAc)\}_2]$ (**5e**).

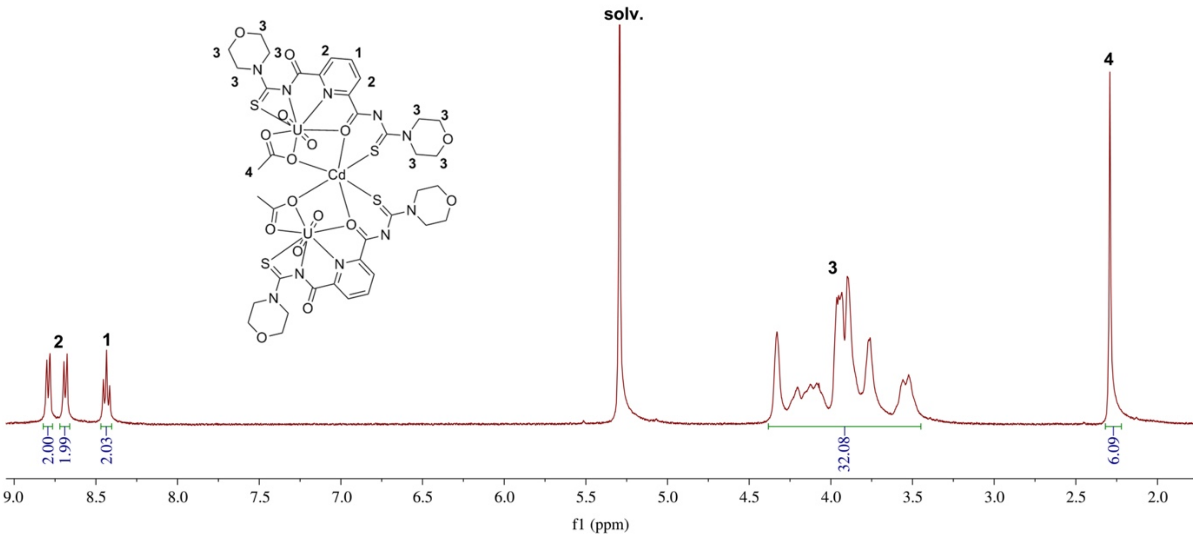

Figure S20: ^{1}H NMR spectrum of $[Cd\{UO_2(L^{Et2})(OAc)\}_2]$ (**6f**) in $CDCl_3$.

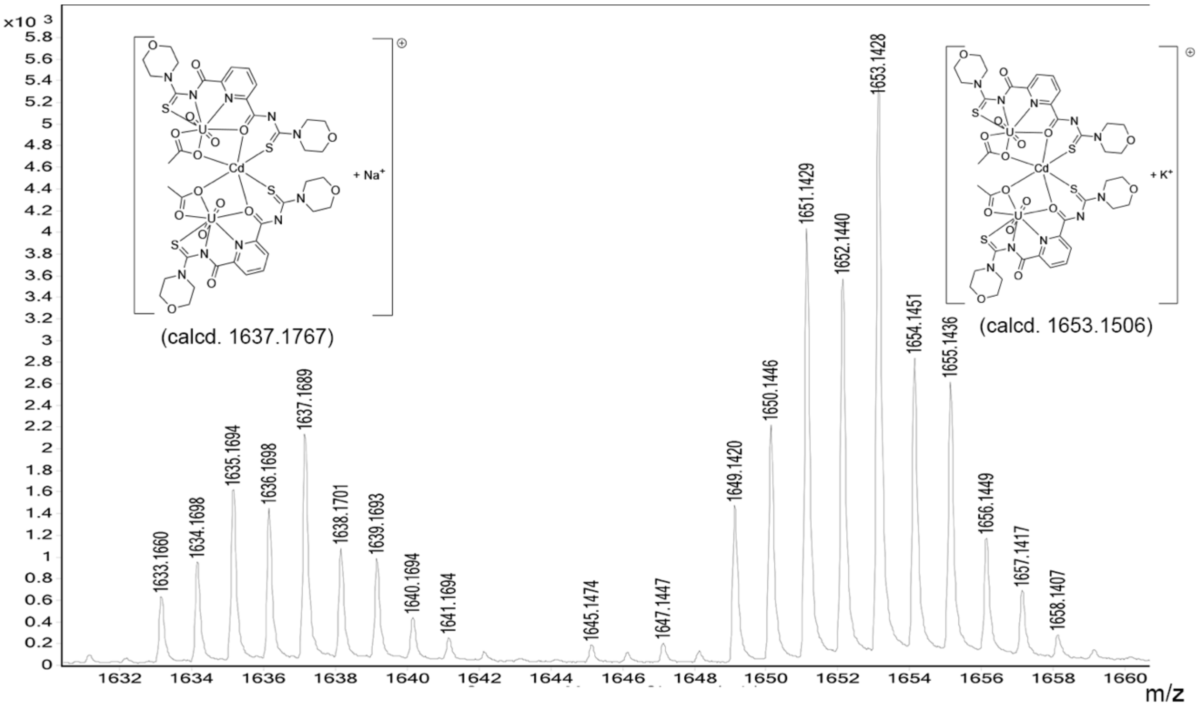

Figure S21: ESI(+) mass spectrum of $[Cd\{UO_2(L^{Et2})(OAc)\}_2]$ (**6f**).

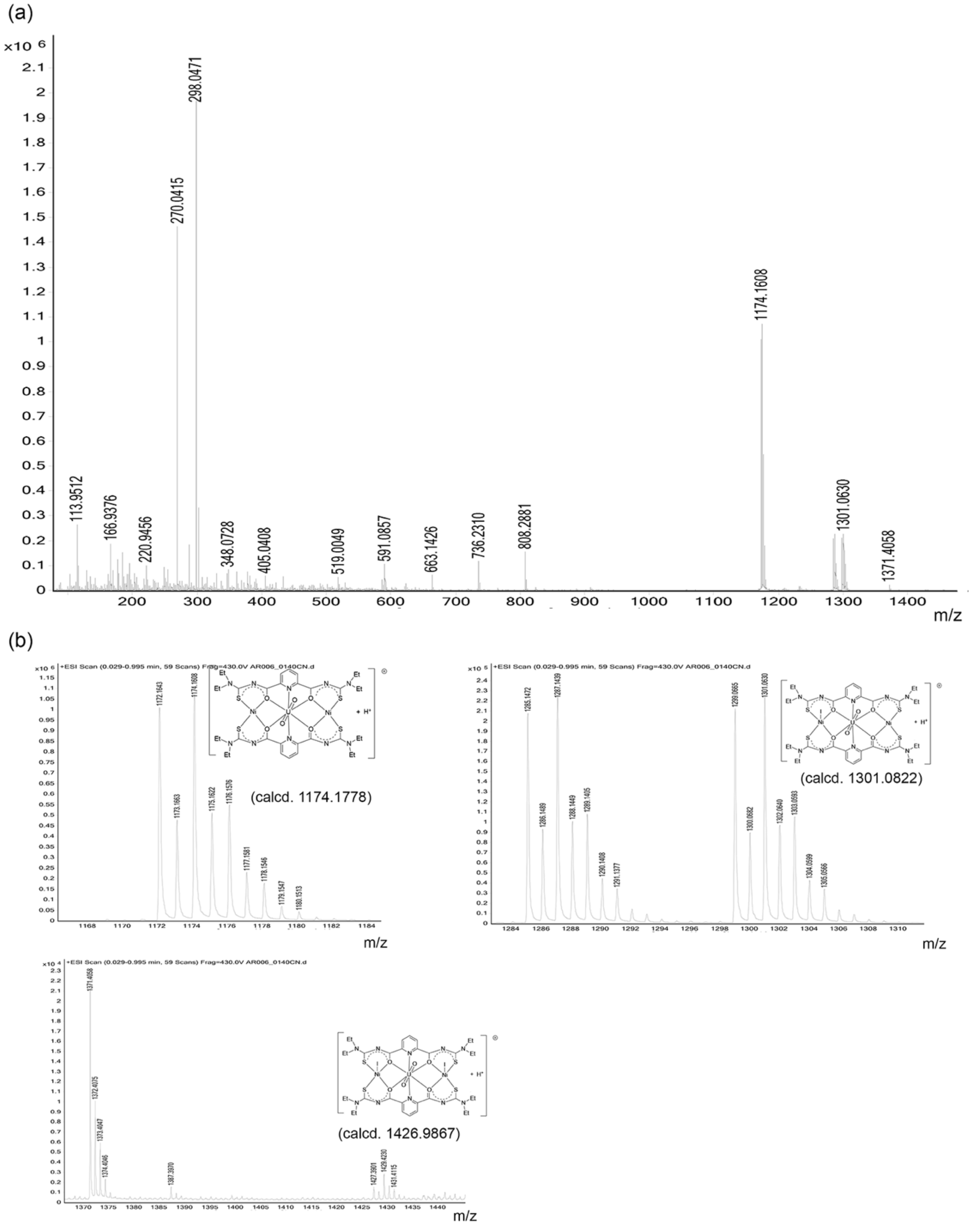

Figure S22: ESI(+) mass spectrum of $[(UO_2)(NiI)_2(L^{Et2})_2]$ (**7**): (a) overview spectrum, (b) high-mass region with assignment.

3. Computational Chemistry

DFT (Density Functional Theory) calculations were performed with the high-performance computing system of the ZEDAT [1] using the program package GAUSSIAN 16 [2]. The gas phase geometry optimizations were performed using coordinates derived from the X-ray crystal structures. The calculations for the ligand molecules H_2L^{Et2} and H_2L^{morph} were performed without any restrictions on the structures by using the hybrid density functional B3LYP [3–5] together with the 6-311G basis set for all atoms as implemented in Gaussian.[6,7] For initial optimization of uranium-containing compounds, the LANL2DZ basis set and the corresponding effective core potential (ECP) was used for uranium [8], while 6-311G was initially kept for the other atoms. The bigger 6-311++G** basis set was used for the other atoms to obtain more reliable geometries [6,7,9–11]. Relativistic, dispersion corrected, all-electron calculations on the PBE0-DKH2-GD3BJ/SARC-DKH2(uranium)+aug-cc-pVTZ-DK(others) level as suggested in ref. [12] were attempted but the system size proved prohibitive. For comparable accuracy as outlined in ref. [13], calculations using the PBE0 hybrid functional [14] with Grimme dispersion and Becke-Johnson damping [15] using the Stuttgart relativistic large core ECP for uranium [16,17], and the def2-TZVPPD basis set for all other atoms [18,19] were attempted. Although some SCF calculations for the *N*,*N*-diethyl derivatives did not converge (see main text), the energetic trends are reasonably close to those obtained at the B3LYP level. The same level of theory (PBE0-GD3BJ/def2-TZVPPD) was applied in single point calculations of the ligand isomers with geometries obtained at the B3LYP/6-311G to obtain more reliable relative energy difference estimated between the different conformers. Further details are given in the Supporting Information. All basis sets were obtained from the EMSL database or the Basis Set Exchange repository [16,17]. Frequency calculations after the optimizations confirmed the convergence through the absence of imaginary frequencies.

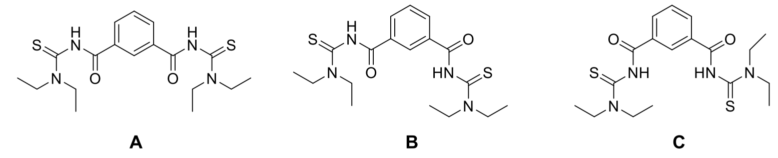

Figure S23: Considered (potential) conformers of H_2L^{Phthal}.

Table S33: DFT calculations of the different conformations of H_2L^{Phthal}. Optimization level: B3LYP/6-311G. Single-point level: PBE0-GD3BJ/def2-TZVPPD.

Isomer	*E*	Δ*E* [Hartree]	Δ*E* [kJ/mol]	Δ*E* [kcal/mol]
**A**	**-1866.4155**	**0.0000**	**0.0**	**0.0**
B	-1866.4073	0.0082	21.5	5.1
C	-1866.4059	0.0096	25.3	6.1

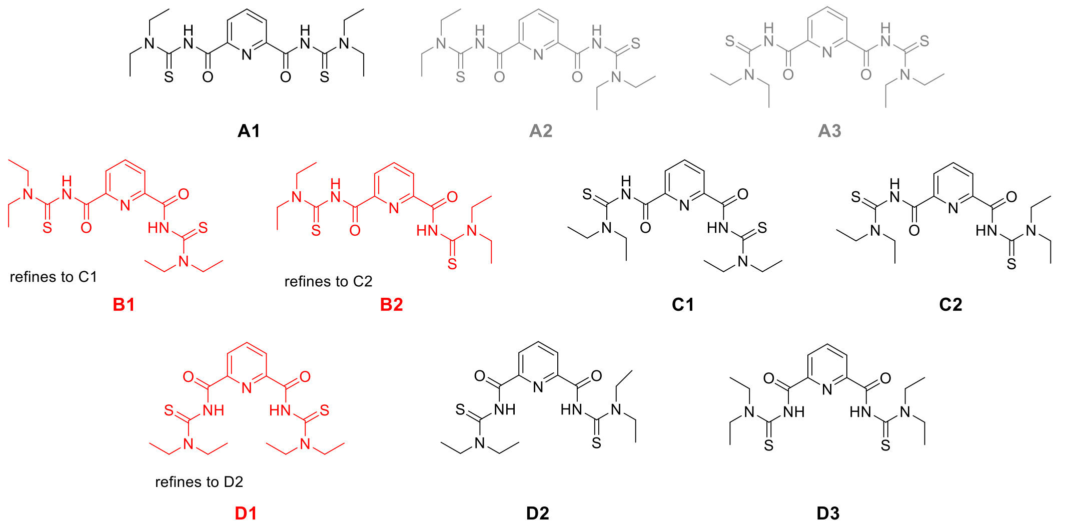

Figure S24: Considered (potential) conformers of H_2L^{Et2}. Stable conformers are black, while unstable conformers are given in red. Grey conformers have not been considered.

Table S34: DFT calculations of the different conformations of H_2L^{morph}. Optimization level: B3LYP/6-311G. Single-point level: PBE0-GD3BJ/def2-TZVPPD. The most stable isomer is bold.

Isomer	*E*	Δ*E* [Hartree]	Δ*E* [kJ/mol]	Δ*E* [kcal/mol]
A1	-2030.3743	0.0111	29.1	7.0
A2		not considered		
A3		not considered		
B1		refines to C1		
B2		refines to C2		
C1	-2030.3662	0.0192	50.4	12.1
C2	-2030.3743	0.0111	29.1	7.0
D1		refines to D2		
**D2**	**-2030.3854**	**0.0000**	**0.0**	**0.0**
D3	-2030.3838	0.0015	4.0	1.0

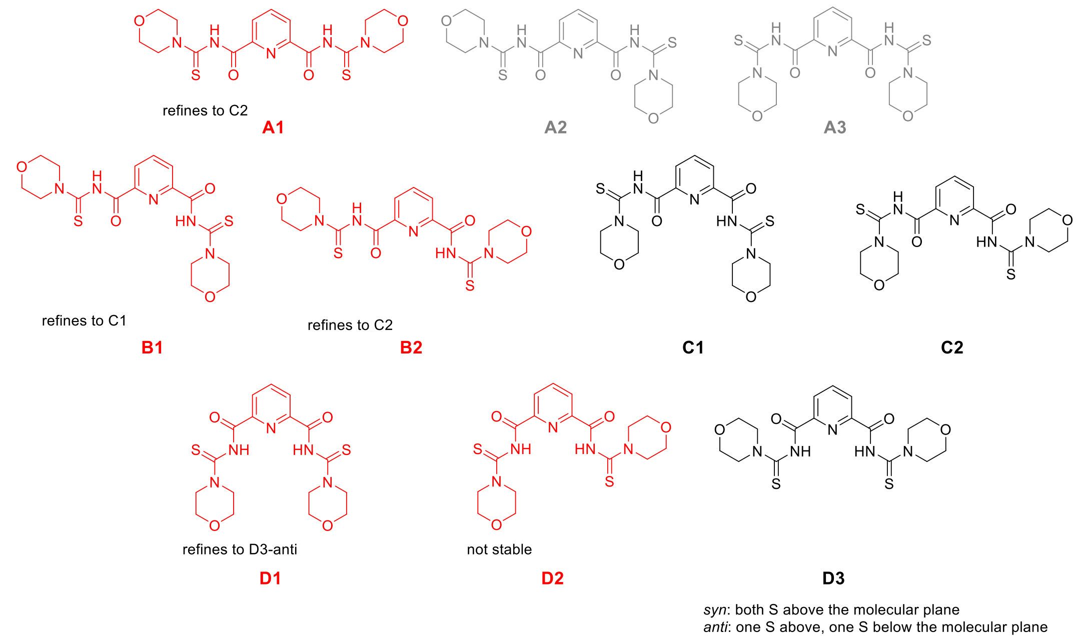

Figure S25: Considered (potential) conformers of H_2L^{morph}. Stable conformers are black, while unstable conformers are given in red. Grey conformers have not been considered.

Table S35: DFT calculations of the different conformations of H_2L^{morph}. Optimization level: B3LYP/6-311G. Single-point level: PBE0-GD3BJ/def2-TZVPPD. The most stable isomer is bold.

Isomer	*E*	Δ*E* [Hartree]	Δ*E* [kJ/mol]	Δ*E* [kcal/mol]
A1	-1882.4352	0.0213	55.9	13.4
A2		not considered		
A3		not considered		
B1		refines to C1		
B2		refines to C2		
C1	-1882.4422	0.0142	37.3	8.9
C2	-1882.4480	0.0084	22.2	5.3
D1		refines to D3-*anti*		
D2		refines to D1		
**D3-*syn***	**-1882.4565**	**0.0000**	**0.0**	**0.0**
D3-*anti*	-1882.4536	0.0028	7.4	1.8

DFT calculations conducted after optimization without any symmetry constraints at the B3LYP/LANL2DZ(uranium)+6-311G(others) level indicated very low energy differences between the conformers in the range of 5-10 kJ/mol at this level of theory. Further optimization of these geometries at the somewhat more accurate B3LYP/LANL2DZ(uranium)+6-311++G**(others) level indicated energy differences of up to 20 kJ/mol between the three isomers. The energetic trends observed for the B3LYP level were verified by even more accurate single point calculations at the PBE0/StuttgartRLC(uranium)+def2-TZVPPD(others) level on the geometries obtained at the B3LYP/LANL2DZ(uranium)+6-311++G**(others) level. These were corrected by Grimme dispersion and Becke-Johnson damping (GD3BJ). Relativistic all-electron calculations at the PBE0-DKH2-GD3BJ/SARC-DKH2(uranium)+aug-cc-pVTZ-DK(others) were attempted using the obtained geometries of the B3LYP/LANL2DZ(uranium)+6-311++G**(others) level but proved prohibitive due to the size of the system and due to the relatively poor structure convergence caused by the flat energy hypersurface regarding additional potential rotamers. The SCF calculation at the PBE0/StuttgartRLC(uranium)+def2-TZVPPD(others) level did not properly converge for the *N,N*-diethyl derivatives even across extremely narrow grids or using initial estimates from lower level calculations. Since the trends for the morpholine conformers were comparable between the B3LYP/LANL2DZ(uranium)+6-311++G**(others) level and the PBE0/StuttgartRLC(uranium)+def2-TZVPPD(others) level, even though the absolute energy differences were somewhat lower for the B3LYP calculation, the trends at the B3LYP should be informative about the relative conformer preference in the *N,N*-diethyl case, too. While a neglectable preference for the *anti,anti* conformation and the *syn,anti* conformation was found for the *N,N*-diethyl and morpholine derivatives on the B3LYP level respectively and the *syn,syn* structure is the least favored conformer in both cases, the *syn,anti* conformer of the morpholine derivative is the most stable by nearly 20 kJ/mol on the higher level calculation including dispersion effects. The individual values are displayed in Figure 1. Even structural differences resulting in energetic differences ranging around 20 kJ/mol as identified for the solid-state geometries can readily be overcome by packing effects in the solid state that become relevant during the crystallization processes.

Table S36: DFT calculations of the different conformations of H_2L^{morph}. Level: B3LYP/LANL2DZ(uranium)+6-311++G**(others). The most stable isomer is bold.

Ligand	Isomer	*E*	Δ*E* [Hartree]	Δ*E* [kJ/mol]	Δ*E* [kcal/mol]
	anti,anti	-4696.273	0.0026	6.8	1.6
H_2L^{morph}	***syn,anti***	**-4696.275**	**0.0000**	**0.0**	**0.0**
	syn,syn	-4696.268	0.0072	19.0	4.5
	anti,anti	**-4400.185**	**0.0000**	**0.0**	**0.0**
H_2L^{Et2}	*syn,anti*	-4400.182	0.0025	6.6	1.6
	syn,syn	-4400.178	0.0066	17.4	4.2

Table S37: DFT calculations of the different conformations of H_2L^{morph}. Optimization level: B3LYP/LANL2DZ(uranium)+6-311++G**(others). Single-point level: PBE0-GD3BJ/StuttgartRLC(uranium)+def2-TZVPPD(others). The most stable isomer is bold.

Ligand	Isomer	*E*	Δ*E* [Hartree]	Δ*E* [kJ/mol]	Δ*E* [kcal/mol]
	anti,anti	-4692.596	0.0060	15.8	3.8
H_2L^{morph}	***syn,anti***	**-4692.602**	**0.0000**	**0.0**	**0.0**
	syn,syn	-4692.593	0.0091	24.0	5.7
	anti,anti		SCF not converged		
H_2L^{Et2}	*syn,anti*	-4396.730			
	syn,syn		SCF not converged		

4. References

1 Bennett, L.; Melchers, B.; Proppe, B. High-Performance Computing at ZEDAT. Freie Universität, Berlin, Germany, 2020. Available online: https://refubium.fu-berlin.de/handle/fub188/26993 (accessed on 30 August 2024).

2 Frisch, M.J.; Trucks, G.W.; Schlegel, H.B.; Scuseria, G.E.; Robb, M.A.; Cheeseman, J.R.; Scalmani, G.; Barone, V.; Petersson, G.A.; Nakatsuji, H.; et al. *Gaussian 16*, Revision A.03; Gaussian, Inc.: Wallingford, CT, USA, 2016.

3 Vosko, S.H.; Wilk, L.; Nusair, M. Accurate spin-dependent electron liquid correlation energies for local spin density calculations: A critical analysis. *Can. J. Phys.* **1980**, *58*, 1200–1211.

4 Becke, A.D. Density-functional thermochemistry. III. The role of exact exchange. *J. Chem. Phys.* **1993**, *98*, 5648–5652.

5 Lee, C.; Yang, W.; Parr, R.G. Development of the Colle-Salvetti correlation-energy formula into a functional of the electron density. *Phys. Rev. B* **1988**, *37*, 785–789.

6 Krishnan, R., Binkley, J. S., Seeger, R., Pople, J. A. Self-consistent molecular orbital methods. XX. A basis set for correlated wave functions. *J. Chem. Phys.* **1980**, *72*, 650-654.

7 McLean, A. D., Chandler, G. S. Contracted Gaussian basis sets for molecular calculations. I. Second row atoms, Z=11-18. *J. Chem. Phys.* **1980**, *72*, 5639-5648.

8 Hay, P. J. Ab initio studies of excited states of polyatomic molecules including spin-orbit and multiplet effects: The electronic states of UF_6. *J. Chem. Phys.* **1983**, *79*, 5469-5482.

9 Spitznagel, G. W., Clark, T., Schleyer, P. von Ragué, Hehre, W. J. An evaluation of the performance of diffuse function-augmented basis sets for second row elements, Na-Cl. *J. Comput. Chem.* **1987**, *8*, 1109-1116.

10 Clark, T., Chandrasekhar, J., Spitznagel, G. W., Schleyer, P. Von Ragué. Efficient diffuse function-augmented basis sets for anion calculations. III. The 3-21+G basis set for first-row elements, Li-F. *J. Comput. Chem.* **1983**, *4*, 294-301.

11 Francl, M. M., Pietro, W. J., Hehre, W. J., Binkley, J. S., Gordon, M. S., DeFrees, D. J., Pople, J. A. Self-consistent molecular orbital methods. XXIII. A polarization-type basis set for second-row elements. *J. Chem. Phys.* **1982**, *77*, 3654-3665.

12 Pantazis, D.A.; Neese, F. All-Electron Scalar Relativistic Basis Sets for the Actinides. *J. Chem. Theory Comput.* **2011**, *7*, 677–684.

13 Shamov, G.A.; Schreckenbach, G.; Vo, T. N. A Comparative Relativistic DFT and AbInitio Study on the Structure and Thermodynamics of the Oxofluorides of Uranium(IV), (V) and (VI). *Chem. Eur. J.* **2007**, *13*, 4932–4947.

14 Adamo, C., Barone, V. Toward reliable density functional methods without adjustable parameters: The PBE0 model. *J. Chem. Phys.* **1999**, *110*, 6158–6170.

15 Grimme, S., Ehrlich, S., Goerigk, L. Effect of the damping function in dispersion corrected density functional theory, *J. Comp. Chem.* **2011**, *32*, 1456-1465.

16 Feller, D. The role of databases in support of computational chemistry calculations. *J. Comput. Chem.* **1996**, *17*, 1571–1586.

17 Schuchardt, K.L.; Didier, B.T.; Elsethagen, T.; Sun, L.; Gurumoorthi, V.; Chase, J.; Li, J.; Windus, T.L. Basis Set Exchange: A Community Database for Computational Sciences. *J. Chem. Inf. Model.* **2007**, *47*, 1045–1052.

18 Rappoport, D., Furche, F. Property-optimized Gaussian basis sets for molecular response calculations. *J. Chem. Phys.* **2010**, *133*, 134105.

19 Weigend, F., Ahlrichs, R. Balanced basis sets of split valence, triple zeta valence and quadruple zeta valence quality for H to Rn: Design and assessment of accuracy. *Phys. Chem. Chem. Phys.* **2005**, *7*, 3297.
